# Phyletic Distribution and Lineage-Specific Domain Architectures of Archaeal Two-Component Signal Transduction Systems

**DOI:** 10.1128/JB.00681-17

**Published:** 2018-03-12

**Authors:** Michael Y. Galperin, Kira S. Makarova, Yuri I. Wolf, Eugene V. Koonin

**Affiliations:** a National Center for Biotechnology Information, National Library of Medicine, National Institutes of Health, Bethesda, Maryland, USA; Rutgers University-Robert Wood Johnson Medical School

**Keywords:** archaeal genomes, signal transduction, arCOGs, membrane proteins, gene neighborhoods, protein-protein interactions, Archaea, halobacterium, genome analysis, genomics, histidine kinase, metagenomics, methanogens, two-component regulatory systems

## Abstract

The two-component signal transduction (TCS) machinery is a key mechanism of sensing environmental changes in the prokaryotic world. TCS systems have been characterized thoroughly in bacteria but to a much lesser extent in archaea. Here, we provide an updated census of more than 2,000 histidine kinases and response regulators encoded in 218 complete archaeal genomes, as well as unfinished genomes available from metagenomic data. We describe the domain architectures of the archaeal TCS components, including several novel output domains, and discuss the evolution of the archaeal TCS machinery. The distribution of TCS systems in archaea is strongly biased, with high levels of abundance in haloarchaea and thaumarchaea but none detected in the sequenced genomes from the phyla Crenarchaeota, Nanoarchaeota, and Korarchaeota. The archaeal sensor histidine kinases are generally similar to their well-studied bacterial counterparts but are often located in the cytoplasm and carry multiple PAS and/or GAF domains. In contrast, archaeal response regulators differ dramatically from the bacterial ones. Most archaeal genomes do not encode any of the major classes of bacterial response regulators, such as the DNA-binding transcriptional regulators of the OmpR/PhoB, NarL/FixJ, NtrC, AgrA/LytR, and ActR/PrrA families and the response regulators with GGDEF and/or EAL output domains. Instead, archaea encode multiple copies of response regulators containing either the stand-alone receiver (REC) domain or combinations of REC with PAS and/or GAF domains. Therefore, the prevailing mechanism of archaeal TCS signaling appears to be via a variety of protein-protein interactions, rather than direct transcriptional regulation.

**IMPORTANCE** Although the Archaea represent a separate domain of life, their signaling systems have been assumed to be closely similar to the bacterial ones. A study of the domain architectures of the archaeal two-component signal transduction (TCS) machinery revealed an overall similarity of archaeal and bacterial sensory modules but substantial differences in the signal output modules. The prevailing mechanism of archaeal TCS signaling appears to involve various protein-protein interactions rather than direct transcription regulation. The complete list of histidine kinases and response regulators encoded in the analyzed archaeal genomes is available online at http://www.ncbi.nlm.nih.gov/Complete_Genomes/TCSarchaea.html.

## INTRODUCTION

All living organisms possess certain means to monitor environmental conditions and react to changes in the environment by adjusting their behavior and/or metabolism. Two-component signal transduction (TCS) systems provide a key mechanism of environmental sensing and intracellular surveillance in most bacteria and some archaea (1–3). The components of the TCS, histidine kinases (HKs) and response regulators (RRs), carry highly conserved phosphotransfer modules, namely, the ATPase (listed as family HATPase_c [Pfam ID PF02518] in the Pfam database [4]) and dimerization (HisKA or DHp; Pfam ID PF00512) domains of the HKs and phosphoacceptor (receiver [REC]; Pfam ID PF00072) domains of the RRs. These conserved modules are combined with a variety of extracellular, integral membrane, or cytoplasmic sensory domains on the HKs and output domains on the RRs (2, 5–7). Such modular architecture accounts for the tremendous diversity of the signals sensed by the TCSs and the cellular responses triggered by them.

The TCS machinery is also found in many archaea and some eukaryotes, but its distribution is strongly biased. Early analyses of archaeal genomes revealed widespread and abundant TCS systems in the members of the archaeal phyla Euryarchaeota and Thaumarchaeota but not among members of Crenarchaeota, Korarchaeota, or Nanoarchaeota (5, 6, 8–11). This remarkable pattern led to the conclusion that TCSs originated in bacteria after the separation of bacterial and archaeal lineages and were subsequently acquired by archaea through multiple events of horizontal gene transfer (8, 12). In recent years, many more archaeal genomes have been sequenced and the initial observations of the biased distribution of TCSs have been confirmed: there are still no TCSs encoded in the finished genomes of any representatives of *Cren*-, *Kor*-, or Nanoarchaeota or “Candidatus Nanohaloarchaeota,” whereas members of Euryarchaeota and Thaumarchaeota encode a variety of HKs and RRs, often with complex domain architectures (5, 6, 9, 10, 13). However, archaeal signaling systems in general and, specifically, archaeal TCSs remain poorly understood.

In contrast to the bacteria, in which multiple HKs and RRs have been characterized both structurally and functionally (2, 7), only a few archaeal TCSs have been studied experimentally. In Halobacterium salinarum, the chemotaxis histidine kinase CheA and response regulators CheY and CheB have been shown to function essentially in the same manner they do in bacteria (14, 15). In addition, the halobacterial light- and redox-sensing transcriptional regulator Bat (bacterio-opsin activator of transcription) (16) contains at its N terminus a divergent REC domain, which, however, had not been recognized until now and whose role in Bat-mediated regulation (17, 18), if any, remains obscure. In Methanosaeta harundinacea, the regulation of methanogenesis involves a TCS consisting of an HK, FilI, and two RRs, FilR1, a transcriptional regulator that combines the C-terminal REC domain with an N-terminal winged helix-turn-helix (wHTH) domain and an uncharacterized DUF1724 (Pfam ID PF08350) domain in the middle, and FilR2, which consists of a stand-alone REC domain (19). Finally, a TCS responsible for temperature-dependent gene regulation in the psychrophilic archaeon Methanococcoides burtonii consists of a thermally unstable HK LtrK and an RR, LtrR, with an HTH-REC domain architecture (20).

Despite these findings, RRs containing a DNA-binding HTH (or wHTH) domain appear to be quite rare in archaea, and most putative archaeal output domains remain either functionally uncharacterized (e.g., the haloarchaeal HalX domain [Pfam ID PF08662] [5]) or not even properly described. General protein family databases, such as Pfam, InterPro, Conserved Domain Database (CDD), SMART, and STRING (4, 21–24), as well as specialized databases of microbial signal transduction (MiST) and prokaryotic two-component systems (P2CS; http://www.p2cs.org/) (13, 25), provide a treasure trove of data on archaeal TCS components, but most of these data remain to be carefully analyzed. As a step toward a better understanding of archaeal TCSs, we provide here a comprehensive census of the HKs and RRs encoded in archaeal genomes, define their common output domains, and discuss the caveats of annotating the archaeal TCSs and their functional features and possible routes of evolution.

## RESULTS

### Distribution of the TCSs in archaea.

**FIG 1:**
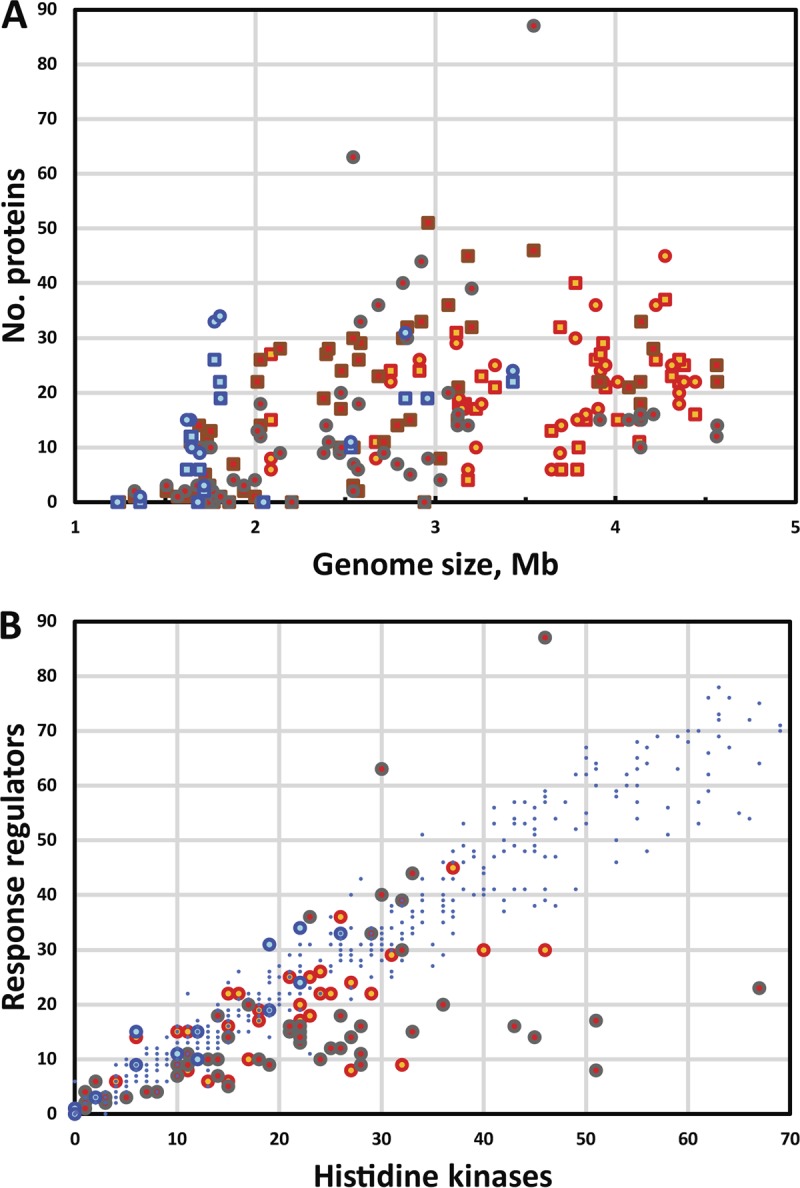
Census of the archaeal two-component signal transduction systems. (A) Total numbers of sensor histidine kinases (HKs) and response regulators (RRs) encoded in 218 archaeal genomes. (B) Ratios of histidine kinases and response regulators in various archaea. Squares represent HKs, and circles represent RRs; symbols representing data for halobacteria are in orange and red, those for methanogens are in brown, and those for thaumarchaea are in blue (9). The small blue dots indicate RR/HK ratios for individual bacterial genomes.

Due to the recent efforts in archaeal genome sequencing, the current collection includes completely sequenced genomes of 218 archaeal species that encode more than half a million protein sequences, of which over 4,000 could be classified as either histidine kinases (HKs) or response regulators (RRs) (Table 1; see Tables S1 and S2 in the supplemental material). Using this up-to-date set of genomes, this study confirmed the previous reports on the biased distribution of TCSs in archaea. We did not find a single HK or RR in any finished genome of the representatives of Crenarchaeota (44 genomes used in this study and 42 additional genomes of different strains from the same species). However, several TCSs have been detected in the unfinished genome sequences of putative crenarchaea obtained in metagenomics studies (see below and Table S3). The current selection of completely sequenced genomes is clearly biased. There are currently only single complete genomes from the phyla Korarchaeota and “*Ca*. Nanohaloarchaeota” and two genomes of the members of Nanoarchaeota; none of them encodes any TCSs. The recently sequenced genome of the first representative of the phylum “Candidatus Micrarchaeota” (26) encodes a single TCS (Table 1). Therefore, this study concentrated on the analysis of Euryarchaeota (149 genomes, 124 of which encode at least one HK and/or RR) and Thaumarchaeota (16 genomes, 12 of which encode TCSs) (Table 1). The total number of HKs and RRs per genome correlates poorly with genome size (Fig. 1) and ranges from zero in *Cren*-, *Kor*-, and Nanoarchaeota and certain members of other phyla to 133 in Methanospirillum hungatei strain JF-1. In the majority of archaeal genomes, HKs and RRs comprise less than 2% of all open reading frames (ORFs), which is comparable to their numbers in bacterial genomes (27). The diversity of TCSs generally correlates with their abundance. Thus, most Pyrococcus and Thermococcus spp., members of the euryarchaeal class Thermococci, encode a single chemotaxis HK, CheA, and two chemotaxis RRs, CheY and CheB, and some species even fewer than that (Table S2). In contrast, members of the classes Halobacteria and Methanomicrobia typically encode 10 or more TCSs, which can comprise up to 3 to 4% of the protein-coding genes.

**TABLE 1:** Distribution of TCSs among major archaeal taxa

Archaeal superphylum, phylum, or class	No. of complete genomes	Total no. of proteins	No. (%) of^*a*^:			
			Histidine kinases	Response regulators		
				All	Containing:	
					REC only	HTH
DPANN group	4					
“*Ca*. Micrarchaeota”	1	952	1	1	—	1
“*Ca*. Nanohaloarchaeota”	1	1,183	—	—	—	—
Nanoarchaeota	2	1,122	—	—	—	—
Euryarchaeota	149					
Archaeoglobi	7	15,162	37	42	32 (76)	
Halobacteria	35	123,786	745	695	163 (23)	93
Methanobacteria	17	33,023	140	150	35 (23)	—
Methanococci	12	19,966	19	23	15 (65)	—
Methanomicrobia	39	108,246	998	697	300 (43)	20
Methanopyri	1	1,687	—	—	—	—
Thermococci	25	51,618	17	31	17 (55)	—
Thermoplasmata	11	18,221	3	4	—	1
Unclassified euryarchaea	3	4,814	4	8	6 (75)	—
TACK group	61					
Thaumarchaeota	16	36,321	175	224	172 (77)	1
Crenarchaeota	44	88,281	—	—	—	—
Korarchaeota	1	1,602	—	—	—	—
Unclassified archaea	4	5,604	—	—	—	—
Total no.	218	511,588	2,139	1,875	740	116

a Among the proteins that combine the HisKA, HATPase, and REC domains, the 53 that contain REC domains at their C termini were counted as histidine kinases, whereas those (445 in total) that contain REC domains on their N termini were counted as response regulators. —, no proteins found.

In contrast to bacterial genomes, which generally encode highly similar numbers of HKs and RRs, both the absolute and the relative numbers of these two components in archaea vary widely, particularly in methanogens (Fig. 1A and B). In many cases, there is evidence of tandem gene duplication, with two or more nearly identical proteins encoded by adjacent genes (Table S2). It has to be noted that throughout this work, as in previous ones (5, 6), HKs that contain REC domains on their N termini were classified as RRs, whereas those HKs that contain REC domains at their C termini are referred to as hybrid HKs and counted as HKs.

### Distinctive features of archaeal histidine kinases. (i) Archaeal sensor domains.

In accordance with previous reports (1, 5, 8, 9, 28), archaeal HKs were generally similar to the bacterial ones in their domain organization but were more likely to be located in the cytoplasm: 62% of HKs were predicted to contain no transmembrane segments (see Fig. S1). More than 72% of HKs carried one or more PAS and/or GAF domains, and at least 330 (∼15%) had both. Many other HKs contained previously described sensor domains, such as MEDS (methanogen/methylotroph, DcmR sensory domain [Pfam ID PF14417]), PocR (Pfam ID PF10114), and HisKA_7TM (Pfam ID PF16927) (29–31). The recently redefined single (sCache) and double (dCache) CACHE domains (32, 33) were far less abundant in archaea than in bacteria. They were almost exclusively found in members of the Methanomicrobia, which is consistent with the suggestion that they had originated in the bacteria after their separation from archaea (33). Archaeal HKs often carried versions of these domains specific for archaea that were not always recognized by the standard domain models and, accordingly, were not properly annotated in the CDD, InterPro, or Pfam outputs. Nevertheless, iterative database searches clearly identified these domains, such as the MEDS domain in the Methanosarcina acetivorans protein MA_3962 (GenBank accession number AAM07313) and other methanogen HKs or the HisKA_7TM domain in the H. salinarum protein VNG_2180C (GenBank accession number AAG20315) and many other haloarchaeal HKs. In addition to these widespread sensor domains, there were some found only in specific archaeal lineages, often restricted to a single family or even a single genus. An example of such domains is the HisKA_4TM (Pfam ID PF16926) sensor domain, found exclusively among haloarchaea.

### (ii) Nonenzymatic HK-like proteins.

In addition to HKs typical of those in bacteria, some archaea encode HK-like proteins with unusual domain architectures that contain typical N-terminal PAS, GAF, and/or other sensor domains and a C-terminal HisKA-like dimerization domain with a conserved His residue but lack recognizable HATPase domains. Such domain architectures, referred to as “Possible incomplete histidine kinase” in the P2CS database (25) and as “HisKA, no HATPase” in Table S2, are found primarily in Archaeoglobi and Methanomicrobia (the genomes of Archaeoglobus fulgidus strain DSM 4304, Methanoculleus marisnigri strain JR1, and Methanolacinia petrolearia strain DSM 11571 each carry 7 such genes). Despite lacking the HATPase domain and therefore being devoid of the kinase activity, most of these proteins are currently misannotated as HKs. Although such annotation is misleading, the conserved His residues of these proteins could still have the phosphoacceptor (and even phosphotransfer) function, allowing participation in signal transduction. An intriguing possibility is that such proteins possess the phosphatase activity toward RRs that appears to be the property of the HisKA-type domains (34).

### Principal classes of archaeal response regulators. (i) Transcriptional regulators.

DNA-binding transcriptional regulators comprise more than two-thirds of all bacterial RRs (5, 6), and most experimentally characterized archaeal RRs are also transcriptional regulators (16, 19, 20). However, the number of archaeal RRs with (known or predicted) DNA-binding HTH domains is actually very small (Table 1). The majority of these are homologs of the Bat-type transcriptional regulator (16, 17, 35) that were detected in multiple copies in nearly all haloarchaeal genomes. In some Bat-like proteins, the N-terminal REC domain is highly diverged or replaced by other domains. The LtrR-like RRs with the HTH-REC (or wHTH-REC) domain architecture (20) were identified only in haloarchaea and Methanosarcina spp. Certain other output domains, including the previously described HalX domain, could potentially contain a helix-turn-helix motif, but their ability to bind DNA remains to be tested. The RR- and HTH-encoding genes are often located in the same genomic neighborhoods (see below), but the ability of their protein products to interact with each other remains to be demonstrated *in vitro* and/or *in vivo*.

### (ii) CheY-like RRs.

**FIG 2:**
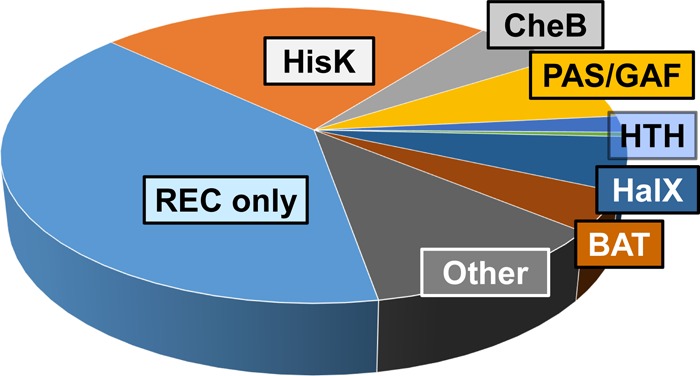
Principal classes of archaeal response regulators. The detailed data are available in Tables S1 and S2 and online at http://www.ncbi.nlm.nih.gov/Complete_Genomes/TCSarchaea.html.

Response regulators that, similarly to the bacterial CheY and Spo0F proteins, consist solely of stand-alone REC domains (36) are even more widespread in archaea than in bacteria. They comprise ∼40% of all archaeal RRs (Fig. 2) and ∼75% of all RRs in the members of Thaumarchaeota and Archaeoglobi (Table 1). In the marine ammonia-oxidizing archaea “Candidatus Nitrosoarchaeum limnia” strain SFB1 and “Candidatus Nitrosopumilus adriaticus,” 28 RRs of the total of 33 and 34, respectively, are such stand-alone REC domains. A relatively small fraction of these RRs (approximately 1 in 7) are encoded within the chemotaxis operons, often next to the CheA-type HKs (Table S2 and MiST database [13]). In thaumarchaea, the genes coding for REC-only RRs are occasionally found adjacent to, but are transcribed divergently from, the genes encoding predicted DNA-binding proteins of the ArsR (HTH_20) family (archaeal Cluster of Orthologous Groups [arCOG] arCOG03067 members), such as in the Nlim_0095-Nlim_0096, Nmar_0452-Nmar_0453, and NSED_08760-NSED_08765 gene pairs. However, most of these REC-only RRs are not encoded in any conserved gene neighborhoods, so their specific functions could not be inferred. Most of the stand-alone REC domains retain the conserved Asp residue that corresponds to the phosphoryl-accepting Asp57 in the CheY protein from E. coli (Fig. S2A), as well as the Mg^2+^-coordinating acidic residues Asp/Glu12 and Asp13 and the phosphoryl group-interacting Thr/Ser87 and Lys109 (37). All these RRs are expected to get phosphorylated and be involved into various protein-protein interactions, particularly those that contain C-terminal extensions with predicted disordered regions, such as Methanosarcina mazei proteins MM_2953 and MM_2954.

### (iii) CheB-type RRs.

Almost half of the analyzed archaeal genomes encode the chemotaxis response regulator CheB, a combination of the REC domain with the protein-glutamate methylesterase (Table 2), similar to the experimentally characterized RR from H. salinarum (14, 15). These RRs are typically encoded in a single copy per genome in the same operons as CheY-type RRs, often right next to them. This association can be used to identify those REC-only RRs that are actually involved in chemotaxis.

**TABLE 2:** Previously described REC-associated domains in archaeal response regulators

Domain^*a*^	Pfam ID	No. of proteins containing domain^*b*^	Accession no. of representative example in:		Typical domain architecture(s)	Phylogenetic distribution
			GenBank	UniProt		
CheB	PF01339	>450	AAG19394	P0DMI2	CheB, REC-CheB	All archaeal phyla
PAS	PF13426	>750	ADJ14551	D8JA29	REC-PAS, PEC-PAS-PAS, REC-PAS-GAF	All archaeal phyla
GAF	PF13492	>450	ADL57802	D9PUA4	REC-GAF, REC-PAS-GAF	All archaeal phyla
HisKA	PF00512	18	ADQ67009	E4NT16	REC-(PAS)*_n_*-HisKA	Most archaeal phyla
HATPase	PF02518		AAB85399	Q2FT91	REC-(PA)*_n_*-HisKA-HATPase	Most archaeal phyla
BAT	PF15915	>250	CAJ52858	Q18GN9	REC-PAS-GAF-BAT-HTH_10	Halobacteria
HTH_10	PF04967	>250	ADE04186	D4GY03	REC-PAS-GAF-BAT-HTH_10	Halobacteria
HalX	PF08663	>550	AAG19349	Q9HR09	HalX, REC-HalX, HxlR-REC-HalX	Halobacteria
Glyco_transf-2_3 (BcsA)	PF13641	13	BAM69725	T2GID5	REC-BcsA, REC-REC-BcsA	Methanobacteriaceae
iKaiC^*c*^	PF06745	9	ABE53050	Q12U26	iKaiC-REC	Methanosarcinaceae
DUF835 (iKaiC)	PF05763	36	AFV24814	K4MHW3	REC-DUF835, REC-PAS-PAS-DUF835	Methanomicrobia, Thermoplasmata
MCPsignal	PF00015	3	ACL17079	B8GK08	REC-PAS-PAS-HAMP-MCPsignal	Methanosphaerula, Methanospirillum
MEDS (iKaiC)	PF14417	2	AFU59108	K0IJ25	MEDS, MEDS-REC, REC-MEDS	“Candidatus Nitrososphaera,” “Candidatus Nitrocosmicus”
TPR-like	PF13414	6	AFS82335	K0BBH8	REC-PAS-TPR, TPR-TPR-TPR-REC	Methanolinea, Nitrosopumilales

a Abbreviated domain names that may represent a group of related Pfam (4) domains, e.g., PAS represents domains from PAS to PAS_11. The Pfam and GAF entries listed are for the versions that are most often found in archaeal RRs.

b In archaea, see the respective Pfam entries for the complete listings.

c iKaiC, inactivated KaiC-like ATPase domain (described in detail in reference 41).

### (iv) REC-HalX.

Response regulators containing the HalX output domain have been identified in multiple copies in many haloarchaeal genomes (5). During the initial analysis of the genome of H. salinarum strain NRC-1, the first sequenced representative of the haloarchaea, two of the three REC-HalX RRs were misannotated as “HoxA-like transcriptional regulator,” most likely because of the presence of the common REC domain. The same annotation has subsequently been assigned to many other RRs of this family, and some of these erroneous annotations remain in the current databases. Given that the HalX domain consists of three predicted α-helices with possible coiled-coil regions (Fig. S3), it might form an HTH (or helix-loop-helix) structure. However, DNA-binding capacity—or any other function—of this domain has not been documented. The current analysis revealed many RR sequences in which HalX domains did not appear in the standard CDD or Pfam/InterPro outputs but could be recognized in iterative searches with PSI-BLAST or jackHMMer. Accordingly, several additional sequences of the HalX domain (Table S4A) were submitted to Pfam, which should result in better recognition of this common haloarchaeal domain.

### (v) REC-(PAS)_*n*_ and REC-PAS-GAF domain combinations.

Some archaea (mostly methanogens) encode RRs with REC-PAS, REC-PAS-PAS, REC-PAS-GAF, and similar domain combinations that include ligand-binding PAS and/or GAF domains (38–40) but do not include any obvious output domains. Such proteins can be expected to dimerize upon phosphorylation of the REC domain and/or change their conformation upon ligand binding by the PAS or GAF domain (38–40). Dimerization is likely to affect functionally relevant protein-protein interactions of these RRs. However, no RRs of this class have been studied experimentally, and their interacting partners remain unknown.

### (vi) REC domains in histidine kinases.

Almost 20% of the archaeal REC-containing proteins (Fig. 2) are histidine kinases (marked as REC-HisK in Table S2), with domain architectures that include an N-terminal REC domain followed by one or more PAS and/or GAF domains and the C-terminal HK-specific dimerization (HisKA or DHp) and ATPase (HATPase) domains. This type of protein has been described previously (5), but none have been characterized experimentally. The phosphorylatable Asp residue (corresponding to Asp57 in CHEY_ECOLI) is conserved in the majority (albeit not in all) of the REC domains in these proteins (Fig. S2B), and some of these lack other conserved residues, so that as many as 40% of them might not get phosphorylated. The REC-HisK domain organization implies involvement of these proteins in signal transduction networks, either as intermediates in phosphorylation cascades or as dedicated intracellular sensors. Indeed, phosphorylation of the REC domains or ligand binding by the PAS/GAF domains could each lead to protein dimerization and activation of the downstream HK domains. In contrast, hybrid HKs like those in bacteria that contain the REC domains at the C termini and likely catalyze intramolecular phosphoryl transfer are rare in archaea. They are found exclusively in the members of the class Methanomicrobia and comprise ∼5% of their HKs (Table S2). However, in the aceticlastic methanogen Methanothrix soehngenii (also referred to as Methanosaeta concilii), all 8 HKs are of the hybrid type. Some of the hybrid HKs contain the second REC domain and/or the Hpt domain (Pfam ID PF01627) at their C termini; such domain architectures can be predicted to allow complex phosphorelays.

Several archaeal RRs, exemplified by the A. fulgidus protein AF_1472, combine the REC domain with PAS and/or GAF domains and the dimerization HisKA domain but lack the HATPase domain. Similarly to the REC- and PAS-/GAF-containing RRs mentioned above, such RRs likely form dimers (and potentially multimers) and participate in protein-protein interactions. Again, no RR of this class has been studied experimentally, and their interacting partners remain unknown.

### (vii) RRs with enzymatic output domains.

Studies on bacterial RRs revealed multiple instances where the REC domains were attached to standard metabolic enzymes, placing their activity under the environmental control (5, 6). The same trend was noticed in archaea, in which some RRs contain an RadA-like NTPase of the KaiC family, the recently described archaeal signal transduction hubs (41), and several other predicted enzymes. All these fusions show narrow phyletic distributions, typically within a certain family or even a single genus of archaea. Thus, the KaiC-REC fusion is represented solely in the members of 6 genera (Methanococcoides, Methanohalobium, Methanohalophilus, Methanolobus, Methanomethylovorans, and Methanosalsum) of the family Methanosarcinaceae (41). Similarly, the fusion of one or two REC domains with the cellulose synthase (BcsA)-like glycosyltransferase domain (Pfam accession number PF13641), previously described in the Methanobacterium thermoautotrophicum protein MTH_548 (5), was detected only in Methanobacterium spp., Methanobrevibacter spp., and Methanothermobacter spp., three genera in the family Methanobacteriaceae (Table 2). Several additional RRs containing predicted enzymatic domains (thioredoxin reductase and pyruvate phosphate dikinase) were identified in unfinished metagenomic samples (see below and Table S3).

### Novel predicted lineage-specific output domains in archaeal RRs.

Analysis of archaeal RRs revealed several types of proteins in which the REC domains were fused to previously unclassified sequences. By clustering these non-REC sequences, we defined several putative novel domains (Table 3). For simplicity, we refer to these REC-associated domains as output domains, although the examples of RRs with REC-PAS and REC-PAS-GAF domain architectures show that these domains could also serve as input (sensor) domains, with the signal output being simply dimerization of the respective RRs and/or their interaction with still-unidentified target proteins. Most of these REC-associated domains showed narrow phyletic distribution and were accordingly denoted “halobacterial output domain” (HalOD1 and HalOD2), “methanogen output domain” (MetOD1 to MetOD5), and so on (Table 3). These names are only provisional and are expected to be replaced by better, more specific ones after these domains are experimentally characterized. Some of these domains are briefly described below; more information is available in the supplemental material.

**TABLE 3:** Novel REC-associated domains in archaeal response regulators

Domain^*a*^	Pfam ID^*b*^	Length (aa)	No. of proteins containing domain^*c*^	Accession no. of representative example in:		Domain architecture(s)	Phylogenetic distribution
				GenBank	UniProt		
AcidOD1	NA	70	2	ADD08891	B5IH54	REC-AcidOD1	Aciduliprofundum
HalOD1	PF18545	80	>2,000	ADE02288	I3R6Z3	REC-HalOD1, HalOD1-PAS, HalOD1-iKaiC	Halobacteria, haloviruses
HalOD2	PF18547	130	14	ACV46423	C7NX58	REC-HalOD2	Halobacteria
MetOD1	PF18546	140	>100	AAM05831	Q8TN48	MetOD1, REC-MetOD1, REC-PAS-MetOD1	Methanobacteria, Methanomicrobia
MetOD2	PF18548	80	>70	ABD41349	Q2FRF9	REC-MetOD2, MetOD3-REC-MetOD2	Methanocellales, Methanomicrobiales
MetOD3	NA	180	4	ABS55483	A7I6X2	MetOD3-REC-MetOD2	Methanoregula
MetOD4	NA	80	3	ADZ10090	F0TAL8	MetOD4, REC-MetOD4	Methanobacterium
MetOD5	NA	300	3	ABS56126	A7I8R5	MetOD5, MetOD5-REC	Methanoregula
NitrOD1	PF18549	70	12	AFS82515	K0BBZ3	NitrOD1, REC-NitrOD1	Nitrosopumilus
NitrOD2	PF18550	90	13	AIF82253	A0A075MLY6	NitrOD2, NitrOD2-REC	Nitrososphaera
NitrOD3	NA	120	3	AIF83507	A0A075MRR1	NitrOD3-REC	Nitrososphaera
NitrOD4	NA	75	4	AFS80878	K0B452	NitrOD4, NitrOD4-REC	Nitrosopumilus
NitrOD5	PF11537	100	16	ABX12603	A9A4B4	NitrOD5, REC-NitrOD5	Nitrosopumilus, “Candidatus Nitrosotalea,” “Candidatus Nitrosotenuis”
TackOD1	PF18551	200	12	AFS80801	K0B8K6	TackOD1, REC-TackOD1, REC-wHTH-TackOD1	TACK group

a Tentative domain names, constructed by combining an abbreviated taxon name with “OD” (output domain). These names are expected to be replaced as soon as these domains are experimentally characterized. Details of the analyses of remote sequence similarities of these domains are presented in Table S5 in the supplemental material.

b NA, not available. The numbered domains are expected to be included in Pfam release 33 [2018]).

c The number of archaeal proteins containing the domain (with any domain architecture) in the NCBI protein database as of 1 July 2017.

### (i) HalOD1.

**FIG 3:**
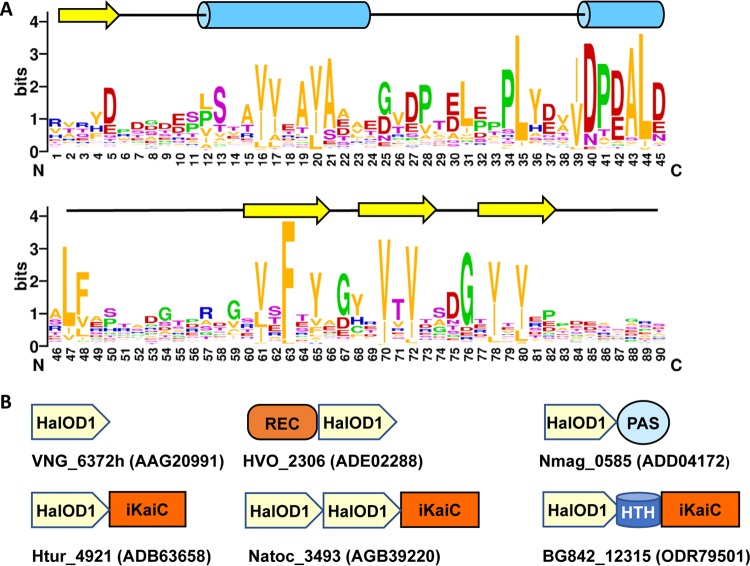
Sequence conservation and domain architectures of HalOD1s. (A) Sequence logo generated by the WebLogo program (77) from an alignment obtained by PSI-BLAST run using as query the sequence of halovirus HRTV-8 protein 1 (GenBank accession number AGM10749; UniProt ID R4T552). The first position of the logo corresponds to Arg21 of HRTV8-1 and to Glu161 of Haloferax volcanii response regulator HVO_2306 (GenBank accession number ADE02288; UniProt ID D4GWD4). Secondary structure prediction (cylinders indicate α-helices, and arrows indicate β-strands) was produced by JPred (84). (B) Domain architectures of selected HalOD1-containing proteins, listed under their locus names and GenBank accession numbers. iKaiC, divergent and possibly inactivated ATPase domain of the KaiC superfamily. The domain architectures for each sector are shown only for comparison and are not scaled to size.

Response regulators with the REC-HalOD1 domain architecture are found primarily in halobacteria of the genus Haloferax and related genera (Table S4B). However, short proteins consisting of the stand-alone HalOD1 (Fig. 3) are encoded in nearly all halobacterial genomes, from a single plasmid-borne gene, Vng_6372h (OE_6052R), in H. salinarum to 49 copies in the genome of Haloterrigena turkmenica strain DSM 5511, with a total of over 2,000 sequences in GenBank (Table S4B). Stand-alone HalOD1 proteins have previously been assigned to the arCOGs arCOG08103, arCOG08928, arCOG08980, arCOG08989, and arCOG09008 (42). In addition to haloarchaea, stand-alone HalOD1s are also encoded in the genomes of haloviruses HF1, HF2, HGTV-1, HRTV-5, HRTV-8, and HSTV-2. This protein domain was also found in combination with REC, PAS, and KaiC-like ATPase (arCOG02452) domains, indicating that it is a key member of the haloarchaeal signal transduction network. Based on the amino acid conservation pattern, which includes two adjacent (DE)hh(DEN) motifs (Fig. 3, positions 37 to 45) (“h” denotes a hydrophobic residue), found, for example, in the EAL-type cyclic di-GMP phosphodiesterases (43), this domain is likely to bind metal ions and might possess an enzymatic activity.

### (ii) HalOD2.

This output domain is found only in a few halobacteria (Table S3C). The predicted secondary structure of its N-terminal part is similar to that of the HalX domain. At the C terminus, it contains two highly conserved CXXC motifs, separated by 20 to 25 residues (Fig. S4). These cysteines form a predicted Zn finger and might be involved in DNA (or RNA) binding.

### (iii) MetOD1.

MetOD1 is found in a variety of methanogens, with slightly different variants in the members of the classes Methanobacteria and Methanomicrobia (Table S4D). Iterative database searches show that this domain is similar to the heme-NO-binding (HNOB), vinyl 4-reductase (V4R), and l-2-amino-thiazoline-4-carboxylic acid hydrolase (ATC hydrolase) domains (Pfam families PF07700, PF02830, and PF14196, respectively). Specifically, MetOD1 shares the predicted secondary structure with the HNOB domain and a cluster of 3 highly conserved Cys residues (Fig. S5) with the V4R domain (in particular, the phenol sensor domains of transcriptional regulators PoxR and MopR [44, 45]) and the ATC hydrolase domain. The ATC hydrolase cleaves the C-S bond in the thiazoline ring structure (46), suggesting that MetOD1 might have a related enzymatic activity.

### (iv) MetOD2 to MetOD5.

MetOD2 is found exclusively in Methanocellales and Methanomicrobiales (Table 3; Table S4E). It consists of four predicted α-helices (Fig. S6), indicating that this domain might represent a distinct version of a DNA- or RNA-binding HTH module. The membrane-anchored MetOD3 and other MetODs are found only within one or two genera of methanogens and include very few members (Table 3).

### (v) NitrOD1 to NitrOD5.

NitrOD1 to NitrOD5 are found almost exclusively in Thaumarchaeota and show a narrow phyletic distribution, typically within a single genus of Nitrosopumilis or “Candidatus Nitrososphaera” (Table 3). However, some of them display remote sequence similarity to domains present in other archaea and/or bacteria (Table S5). As with HalOD1, these domains are often found in a stand-alone form, in multiple copies per genome, and only a few of these are fused with the REC domain. Thus, “Candidatus Nitrososphaera evergladensis” encodes 8 copies of the stand-alone NitrOD2 and 2 more in the NitrOD2-REC combination. A stand-alone NitrOD5 is found in 7 copies in the genome of “Candidatus Nitrosotalea devanaterra” and in 4 copies in “Candidatus Nitrosotenuis cloacae,” whereas Nitrosopumilus maritimus encodes a single copy of this domain in a REC-NitrOD5 architecture. HHpred searches show that NitrOD1 may be a variant of the Lrp/AsnC ligand-binding domain (Pfam ID PF01037), whereas NitrOD3 is related to the Roadblock/LC7 (Pfam ID PF03259) domain (Table S5). NitrOD2, NitrOD4, and NitrOD5 belong to the archaeal “Death domain-like” family (41).

### (vi) TackOD1.

In contrast to all other novel output domains (Table 3), TackOD1 is found in members of several different phyla, primarily in Thaumarchaeota but also in Korarchaeota and unfinished genomes of “Candidatus Bathyarchaeota” and “*Ca*. Odinarchaeota,” as well as in some bacteria. Proteins containing this domain have previously been included in arCOG06883 and found within thaumarchaeal type IV pilus loci (47). It contains 11 highly conserved Cys residues, which form 5 CXXC motifs and an HXXC motif (Fig. S7). This arrangement is similar to the one in the Double Zinc Ribbon (DZR; Pfam ID PF12773) domain but includes three more cysteines. An insert domain with 8 similarly located cysteines is found in the DNA helicase PriA, where it binds two Zn ions and positions a β-hairpin that likely acts as a DNA-unwinding wedge (48). However, apart from the predicted metal binding, which might stabilize the protein structure, the functions of these cysteines and of TackOD1 domain as such remain obscure.

### (vii) Other output domains.

Several additional REC-associated domains (including AcidOD1; Table 3) were found only in one or two species of a single archaeal genus. The respective proteins are listed as “Other RRs” in Table S2. With continued archaeal genome sequencing, new instances of these domains are expected to be identified, which will allow a more informative analysis.

### Response regulators encoded in unfinished archaeal genomes.

In addition to the RRs encoded in complete archaeal genomes, we identified some interesting domain combinations that so far are detectable only in unfinished genomes (and therefore not listed in Table 3). Several deep-branching archaeal lineages have recently been inferred based on metagenomic sequencing data and are not represented by a single finished genome. These include the Asgard group with at least 4 predicted phyla, 6 of the 8 phyla in the putative DPANN superphylum, and 3 new phyla in the TACK superphylum (49–51) (https://www.ncbi.nlm.nih.gov/Taxonomy/Browser/wwwtax.cgi?name=Archaea). Although searching the metagenomic sequence data does not allow accurate accounting of the full gene complements of the respective species, these sequences provide some insight into the signaling mechanisms in the proverbial “microbial dark matter” (50). The list of HKs and RRs found in metagenomics sequences that are attributed to the new archaeal phyla is available as Table S3 and also at https://www.ncbi.nlm.nih.gov/Complete_Genomes/TCSarchaea-unfin.html. The main findings from this analysis are as follows.

In most new phyla, there are representatives encoding at least some TCSs. This is the case for all 4 phyla in the Asgard group, 5 of 8 phyla in the DPANN group, and 4 of 6 phyla the in TACK group. Notably, while the available finished genomes of Crenarchaeota do not encode any TCSs, there are several contigs assigned to crenarchaea that do encode some HKs and RRs. Most RRs encoded in these unfinished genomes are of typical archaeal varieties, as described above, with a clear predominance of CheY-type REC-only RRs, as well as REC-PAS and REC-(PAS)_*n*_-HisK domain combinations. There are also some bacterial-type RRs that have not yet been detected in finished archaeal genomes. These include, among others, the NtrC family RRs (REC-AAA-HTH domain architecture) in two crenarchaea and in “Candidatus Thorarchaeota archaeon SMTZ1-83,” a combination of REC with the protein phosphatase 2C (PP2C)-type protein serine phosphatase domain (RsbU/SpoIIE) in three Arc I group archaea, a WspR-like (fusion of REC with inactivated GGDEF domain) RR in two genomes of “Candidatus Micrarchaeum acidiphilum,” the Mycobacterium-like combination of the REC domain with thioredoxin reductase in two organisms from “Candidatus Heimdallarchaeota,” and the Bacteroides-like combination of REC with pyruvate phosphate dikinase in three members of “*Ca*. Heimdallarchaeota.” In addition, the genome of Lokiarchaeum sp. strain GC14_75 encodes four proteins with the same Arc-type ribbon-helix-helix-HisKA-REC domain architecture that is also encoded in the marine sediment metagenome. Given that the only GGDEF domain detected so far in any archaeon comes from the uncultured methanogenic archaeon Methanocella arvoryzae (GenBank accession number CAJ37382), it remains to be determined whether these bacterial-type RRs are genuine archaeal proteins or represent contamination of archaeal contigs with bacterial DNA.

## DISCUSSION

Since the discovery by Woese and Fox that methanogens and halobacteria form a distinct “archaebacteria” lineage (52), various aspects of archaeal biology have been thoroughly investigated. With the arrival of genome sequences, it became clear that most of the archaeal enzymes and structural proteins involved in DNA replication and repair, transcription, translation, and membrane ATP biosynthesis are distinct from bacterial ones and resemble eukaryotic homologs, whereas the enzymes catalyzing the reactions of central metabolism are largely shared with bacteria (53–55). This chimeric composition of the archaeal proteomes is manifested most clearly in transcription, where the binding of a eukaryotic-type RNA polymerase with eukaryotic-type basal transcription factors to eukaryotic-type promoters is controlled mostly by bacterial-type transcriptional regulators (56–61). The presence of two-component systems in some archaea but not others is in line with these observations and has led to the scenario in which the TCSs originated in bacteria after their separation from the last common cellular ancestor and radiated into archaea and eukaryotes through multiple horizontal gene transfer events (8, 12). This hypothesis is consistent with the widespread representation and diversity of TCS signaling in bacteria and its limited distribution in archaea and eukaryotes (8). Nevertheless, the genomes of certain euryarchaea (Haloarcula marismortui, Methanococcoides burtonii, and Methanospirillum hungatei), as well as the recently sequenced genomes of such thaumarchaea as “Candidatus Nitrososphaera gargensis,” encode numerous diverse TCSs, on par with any bacteria (see Table S1 in the supplemental material), which has been appropriately noted in the respective genome descriptions (62–65).

However, the present analysis revealed several important aspects in which archaeal two-component systems differ from the bacterial ones. These include the following: (i) the total contents of HKs and RRs encoded in archaea, which are typically somewhat smaller than in bacteria (Fig. 1A); (ii) the HK/RR ratio, which, especially in methanogens, varies within a much wider range than in bacteria (Fig. 1B); (iii) the much higher fraction of cytosol-located sensors (Fig. S1), which has also been observed for archaeal chemoreceptors (66); (iv) the abundance (predominance in some lineages) of stand-alone REC domains; (v) the absence of typical bacterial DNA-binding RRs of the OmpR/PhoB, NarL/FixJ, NtrC, AgrA/LytR, and ActR/PrrA families and RRs with GGDEF and/or EAL output domains (Fig. 2; Table S1); and (vi) the presence of domain combinations apparently specific for archaea, such as REC-(PAS/GAF)_*n*_ or REC-(PAS/GAF)_*n*_-HisKA, that include ligand-binding and protein-interacting domains but contain no obvious output domains. These features, viewed together with the biased distribution of HKs and RRs among archaeal phyla (Table 1), are generally compatible with the bacterial origin of the archaeal TCS machinery but suggest that these systems were acquired early in the archaeal evolution and their spread was not limited to the euryarchaeal and thaumarchaeal lineages.

This study also illuminates the poor state of the genome annotation of the archaeal TCS machinery. The difficulties with the annotation of multidomain proteins have been discussed previously (67, 68), but archaeal TCSs represent a special case. Due to the substantial differences between bacterial and archaeal systems, automatic transfer of the annotation of the better-characterized bacterial proteins often leads to errors. Such erroneous annotation often overlooks the presence of known but divergent domains (such as the REC domain in haloarchaeal RRs of the BAT family) and obscures the presence of previously unrecognized output domains specific for archaea. We hope that delineation of several such domains (Table 3) prompts their experimental characterization.

In this work, analysis of the complete archaeal genome sequences was supplemented with a limited analysis of unfinished genomes derived from metagenomic samples. This extension of our analysis allowed expansion of the coverage to 8 additional archaeal phyla, the detection of TCSs in members of Crenarchaeota and “*Ca*. Nanohaloarchaeota,” and the identification of several new and interesting domain architectures (Table S3). However, most HKs and RRs encoded in unfinished genomes were related to those identified in complete genomes. Thus, the trends outlined here are likely to remain valid even after the substantial expansion of the coverage of archaeal genome diversity that is expected in the near future (69).

Combining these observations with the recently proposed role of the KaiC ATPase superfamily as a central hub of the archaeon-specific signal transduction network specific for archaea (41), it appears that, analogously to the chimeric composition of the transcriptional machinery, the archaeal signal transduction system is a hybrid between bacterial components and the ancestral archaeal KaiC-based machinery. These findings underscore the remarkable ability of archaea to incorporate bacterial components into their native regulatory framework on multiple independent occasions and the fast evolution and turnover of these components in different archaeal lineages.

## MATERIALS AND METHODS

### Identification of archaeal HKs and RRs.

Sequence analysis of archaeal TCSs was performed essentially as described previously (5, 9, 70). The list of archaea with completely sequenced genomes, as well as their genome sizes and the numbers of proteins encoded, was extracted from the NCBI Genome website (https://www.ncbi.nlm.nih.gov/genome/) (71). This list was reconciled with the one on the EBI genome website (http://www.ebi.ac.uk/genomes/archaea.html) and trimmed to leave only a single representative per species, which resulted in a set of 218 genomes that were available by 1 July 2017 (see Table S1 in the supplemental material; four genomes that did not provide protein translations were excluded from consideration). The taxonomic assignments of the selected organisms were taken from the NCBI Taxonomy database (72). The lists of HKs encoded in each archaeal lineage were generated through PSI-BLAST searches (73) of the entries from the selected taxa in the NCBI protein database, using as the query the 200-amino-acid (aa) C-terminal fragment of the Archaeoglobus fulgidus HK with the locus tag AF_0770 (GenBank accession number AAB90464; UniProt identifier [ID] O29488_ARCFU). To accelerate the search by using the existing profiles of HisKA and HATPase domains, the first iteration of PSI-BLAST was run using the DELTA-BLAST tool (74). It was followed by several iterations of PSI-BLAST with default parameters and terminated when new iterations retrieved only sequences of other members of the GHKL superfamily, such as DNA gyrase, DNA topoisomerase VI, MutL, or heat shock protein 90 (HSP90)-like ATPase. These results were validated through additional database searches against selected families of Halobacteria and Methanomicrobia using taxon-specific queries. The lists of HKs retrieved by BLAST searches were compared with those obtained by extracting from the NCBI protein database those entries that contained annotated HisKA and/or HATPase c domains. The domain architectures of the proteins in the combined HK lists were manually checked against the Conserved Domain Database (CDD) and InterPro database. The presence of PAS and/or GAF domains was additionally evaluated using PSI-BLAST (73) and CD Search (75). Transmembrane segments were predicted using TMHMM (76).

The lists of REC domain-containing proteins encoded in each individual genome were obtained by PSI-BLAST searches of selected taxon entries in the NCBI protein database using the sequence of the H. salinarum CheY protein (GenBank accession number AAG19395; UniProt ID CHEY_HALS3) as the query (again, DELTA-BLAST was used for the first iteration of each search but subsequent PSI-BLAST iterations were run to convergence) and by extracting the REC domain assignments from the NCBI's CDD (22). These lists of RRs were combined and sorted according to their domain architectures. The HK and RR counts obtained were checked against those in the MiST and P2CS databases (13, 25), and most discrepancies (caused largely by different counting strategies) have been reconciled. No attempt was made to identify potentially missed and/or untranslated HKs or RRs (see the P2CS website for examples).

Sequence logos were generated using the WebLogo program (77) from the alignments obtained by running PSI-BLAST against the selected protein sets using CheY proteins from Escherichia coli (GenBank accession number AAA23577; UniProt ID CHEY_ECOLI) and H. salinarum as queries. The PSI-BLAST outputs were formatted using the “Query-anchored with letters for identities” option and edited to remove those residues that did not align with CHEY_ECOLI. Genomic neighborhoods were analyzed using the MicrobesOnline and SEED databases (78, 79) and by checking the association of archaeal Clusters of Orthologous Genes (arCOGs) (42), which are available at the NCBI FTP site (ftp://ftp.ncbi.nih.gov/pub/wolf/COGs/arCOG/).

The complete list of genomes analyzed, with the respective numbers of HKs and RRs, is presented in Table S1. An expanded HTML version of that table that includes a listing of all HKs and RRs encoded is provided as Table S2 and is also available online at https://www.ncbi.nlm.nih.gov/Complete_Genomes/TCSarchaea.html. As rationalized in the previous studies (5, 9, 70), HKs that contain REC domains on their N termini were counted as RRs, whereas HKs that contain REC domains at their C termini were referred to as hybrid HKs and counted as HKs. A list of HKs and RRs encoded in several unfinished archaeal genomes is provided as Table S3 and is available online at https://www.ncbi.nlm.nih.gov/Complete_Genomes/TCSarchaea-unfin.html. In addition to the bona fide HKs and RRs, this study identified a small number of proteins with highly divergent, truncated, and/or frameshifted REC domains; these were not included in the total count (marked by asterisks in Table S2).

### Sequence analysis of RR output domains.

The domain architectures of the archaeal RRs were manually checked against the CDD/SPARCLE and InterPro databases (21, 22). The RRs that contained, in addition to the REC domain, unassigned regions of ≥70 amino acid residues were marked as containing putative novel output domains (Table S4), and those regions were submitted for additional BLAST searches against the entire NCBI protein database and jackHMMer searches against UniProt (80, 81). For those regions that were found in multiple RRs, multiple alignments were generated from the BLAST outputs. These putative novel output domains were compared against arCOGs and further analyzed for remote sequence similarity using the CD Search (75) with relaxed cutoffs and the HHpred tool (82) of the MPI Bioinformatics Toolkit (83) (Table S5). Protein secondary structures were taken from the HHpred outputs and/or predicted using JPred (84). Putative novel output domains were assigned mnemonic names and submitted for inclusion in the Pfam database (4).

## Supplementary Material

Supplemental material
