# Supplementary material for: Phyletic Distribution and Lineage-Specific Domain Architectures of Archaeal Two-Component Signal Transduction Systems
Source: J Bacteriol. 2018 Mar 12;200(7):e00681-17. doi: 10.1128/JB.00681-17 (PMC5847659; doi:10.1128/JB.00681-17)
Supplement: Supplemental material [file supp_200_7_e00681-17__index.html]

Supplemental material 

# Phyletic Distribution and Lineage-Specific Domain Architectures of Archaeal Two-Component Signal Transduction Systems

## Supplemental material

- Supplemental file 1 -

  Tables S1 (Archaeal genomes used and a census of their TCSs) and S5 (Details of the sequence analysis of the newly described archaeal domains) and Fig. S1 (Transmembrane histidine kinase content in bacteria and archaea) and S2 to S7 (Sequence conservation in archaeal receiver domains [S2], the HalX domain [S3], the HalOD2 domain [S4], the MetOD1 domain and its domain combinations [S5], the MetOD2 domain and its domain combinations [S6], and the TackOD1 domain [S7])

  PDF, 1.8M
- Supplemental file 2 -

  Table S2 (Histidine kinases and response regulators encoded in complete genomes of archaea)

  PDF, 8.6M
- Supplemental file 3 -

  Table S3 (Histidine kinases and response regulators encoded in unfinished genomes of archaea)

  PDF, 306K
- Supplemental file 4 -

  Table S4 (Examples of the HalX, HalOD1, HalOD2, MetOD1, MetOD2, and TackOD1 domains)

  XLSX, 266K
