# Supplementary material for: Phyletic Distribution and Lineage-Specific Domain Architectures of Archaeal Two-Component Signal Transduction Systems
Source: J Bacteriol. 2018 Mar 12;200(7):e00681-17. doi: 10.1128/JB.00681-17 (PMC5847659; doi:10.1128/JB.00681-17)
Supplement: Supplemental material [file JB.00681-17_zjb999094653s1.pdf]

Table S1. Histidine kinases and response regulators encoded in complete archaeal genomes

Phylum (class), organism name	GenBank entry	Strain TaxID	Encoded proteins	All HisK	All RRs	Response regulators							
						REC only	CheB type	REC-HisK	REC-PAS	With HTH	REC-HalX	Bat	Other
**Candidatus Korarchaeota**													
Candidatus Korarchaeum cryptofilum OPF8	CP000968	374847	1,602	**0**	**0**	**0**	**0**	**0**	**0**	**0**	**0**	**0**	**0**
**Candidatus Micrarchaeota**													
Candidatus Micrarchaeota archaeon Mia14	CP019964	1920749	**952**	**1**	**1**	**0**	**0**	**0**	**0**	**1**	**0**	**0**	**0**
**Candidatus Nanohaloarchaeota**													
Nanohaloarchaea archaeon SG9	CP012986	1737403	1,183	**0**	**0**	**0**	**0**	**0**	**0**	**0**	**0**	**0**	**0**
**Crenarchaeota**			**88,281**	**0**	**0**	**0**	**0**	**0**	**0**	**0**	**0**	**0**	**0**
Acidianus hospitalis W1	CP002535	933801	2329	0	0	0	0	0	0	0	0	0	0
Acidianus manzaensis	CP020477	282676	2,644	0	0	0	0	0	0	0	0	0	0
Acidilobus saccharovorans 345-15	CP001742	666510	1,499	0	0	0	0	0	0	0	0	0	0
Aeropyrum camini SY1 = JCM 12091	AP012489	1198449	1,646	0	0	0	0	0	0	0	0	0	0
Aeropyrum pernix K1	BA000002	272557	1,841	0	0	0	0	0	0	0	0	0	0
Caldisphaera lagunensis DSM 15908	CP003378	1056495	1474	0	0	0	0	0	0	0	0	0	0
Caldivirga maquilingensis IC-167	CP000852	397948	1,963	0	0	0	0	0	0	0	0	0	0
Desulfurococcus amylolyticus DSM 16532	CP003321	768672	1,421	0	0	0	0	0	0	0	0	0	0
Desulfurococcus kamchatkensis 1221n	CP001140	490899	1,471	0	0	0	0	0	0	0	0	0	0
Desulfurococcus mucosus DSM 2162	CP002363	765177	1,345	0	0	0	0	0	0	0	0	0	0
Fervidicoccus fontis Kam940	CP003423	1163730	1,387	0	0	0	0	0	0	0	0	0	0
Hyperthermus butylicus DSM 5456	CP000493	415426	1,602	0	0	0	0	0	0	0	0	0	0
Ignicoccus hospitalis KIN4/I	CP000816	453591	1,434	0	0	0	0	0	0	0	0	0	0
Ignicoccus islandicus DSM 13165	CP00686	940295	1,511	0	0	0	0	0	0	0	0	0	0

Ignisphaera aggregans DSM 17230	CP002098	583356	1,930	0	0	0	0	0	0	0	0	0	0
Metallosphaera cuprina Ar-4	CP002656	1006006	2,029	0	0	0	0	0	0	0	0	0	0
Metallosphaera sedula DSM 5348	CP000682	399549	2,256	0	0	0	0	0	0	0	0	0	0
Pyrobaculum sp. WP30	CP012158	1227555	2,216	0	0	0	0	0	0	0	0	0	0
Pyrobaculum aerophilum str. IM2	AE009441	178306	2,605	0	0	0	0	0	0	0	0	0	0
Pyrobaculum arsenaticum DSM 13514	CP000660	340102	2,299	0	0	0	0	0	0	0	0	0	0
Pyrobaculum calidifontis JCM 11548	CP000561	410359	2,149	0	0	0	0	0	0	0	0	0	0
Pyrobaculum ferrireducens	CP003098	1104324	2,827	0	0	0	0	0	0	0	0	0	0
Pyrobaculum islandicum DSM 4184	CP000504	384616	1,978	0	0	0	0	0	0	0	0	0	0
Pyrobaculum neutrophilum V24Sta	CP001014	444157	2,125	0	0	0	0	0	0	0	0	0	0
Pyrobaculum oguniense TE7	CP003316	698757	2835	0	0	0	0	0	0	0	0	0	0
Pyrodictium delaneyi	CP013011	1273541	2,012	0	0	0	0	0	0	0	0	0	0
Pyrolobus fumarii 1A	CP002838	694429	1967	0	0	0	0	0	0	0	0	0	0
Staphylothermus hellenicus DSM 12710	CP002051	591019	1599	0	0	0	0	0	0	0	0	0	0
Staphylothermus marinus F1	CP000575	399550	1570	0	0	0	0	0	0	0	0	0	0
Sulfolobus sp. A20	CP017006	1891280	2497	0	0	0	0	0	0	0	0	0	0
Sulfolobus acidocaldarius DSM 639	CP000077	330779	2,223	0	0	0	0	0	0	0	0	0	0
Sulfolobus islandicus M.16.4	CP001402	426118	2,609	0	0	0	0	0	0	0	0	0	0
Sulfolobus solfataricus P2	AE006641	273057	2,977	0	0	0	0	0	0	0	0	0	0
Sulfolobus tokodaii str. 7	BA000023	273063	2,825	0	0	0	0	0	0	0	0	0	0
Thermofilum adornatus	CP006646	1365176	1,896	0	0	0	0	0	0	0	0	0	0
Thermofilum carboxyditrophus 1505	CP007493	697581	1,924	0	0	0	0	0	0	0	0	0	0
Thermofilum pendens Hrk 5	CP000505	368408	1,876	0	0	0	0	0	0	0	0	0	0
Thermofilum uzonense	CP009961	1550241	1641	0	0	0	0	0	0	0	0	0	0
Thermogladius cellulolyticus 1633	CP003531	1184251	1,414	0	0	0	0	0	0	0	0	0	0
Thermoproteus tenax Kra 1	FN869859	768679	2,049	0	0	0	0	0	0	0	0	0	0
Thermoproteus uzoniensis 768-20	CP002590	999630	2,186	0	0	0	0	0	0	0	0	0	0
Thermosphaera aggregans DSM 11486	CP001939	633148	1,387	0	0	0	0	0	0	0	0	0	0
Vulcanisaeta distributa DSM 14429	CP002100	572478	2,493	0	0	0	0	0	0	0	0	0	0
Vulcanisaeta moutnovskia 768-28	CP002529	985053	2,320	0	0	0	0	0	0	0	0	0	0

**Euryarchaeota**													
**Archaeoglobi**			**15,162**	**38**	**42**	**32**	**5**	**1**	**0**	**0**	**0**	**0**	**4**
Archaeoglobus fulgidus DSM 4304	AE000782	224325	2400	13	12	9	1	1	0	0	0	0	1
Archaeoglobus profundus DSM 5631	CP001857	572546	1818	3	7	4	1	0	0	0	0	0	2
Archaeoglobus sulfaticallidus PM70-1	CP005290	387631	2213	4	5	4	0	0	0	0	0	0	1
Archaeoglobus veneficus SNP6	CP002588	693661	2065	4	3	2	1	0	0	0	0	0	0
Ferroglobus placidus DSM 10642	CP001899	589924	2463	4	4	3	1	0	0	0	0	0	0
Geoglobus acetivorans SBH6	CP009552	565033	2,218	5	8	7	1	0	0	0	0	0	0
Geoglobus ahangari	CP011267	113653	1,985	4	3	3	0	0	0	0	0	0	0
**Halobacteria**			**123,786**	**745**	**695**	**163**	**35**	**230**	**51**	**11**	**110**	**81**	**14**
Halalkalicoccus jeotgali B3	CP002062	795797	3,873	6	14	4	0	2	2	1	2	3	0
Halanaeroarchaeum sulfurireducens HSR2	CP008874	1604004	2,228	27	8	1	1	3	1	0	2	0	0
Haloarcula hispanica ATCC 33960	CP002921	634497	3,865	26	36	3	1	18	6	0	6	2	0
Haloarcula marismortui ATCC 43049	AY596297	272569	4,240	37	45	7	1	21	7	1	6	2	0
Haloarcula sp. CBA1115	CP010529	1592728	3,916	26	36	3	1	19	5	0	6	2	0
Halobacterium hubeiense	LN831302	1407499	3,143	18	19	2	1	6	2	0	6	2	0
Halobacterium salinarum NRC-1	AE004437	64091	2,622	11	8	1	1	2	0	0	3	1	0
Halobacterium sp. DL1	CP007060	751944	3,280	18	17	5	1	4	1	0	4	1	1
Halobiforma lacisalsi AJ5	CP019285	358396	4,097	25	22	8	2	6	0	0	2	4	0
Halodesulfurarchaeum formicicum	CP016804	1873524	2079	15	6	1	1	2	0	0	1	1	0
Haloferax gibbonsii ATCC 33959	CP011947	1227459	3,744	27	24	3	1	10	3	0	2	4	1
Haloferax mediterranei ATCC 33500	CP001868	523841	3,752	22	17	3	1	4	3	0	1	4	1
Haloferax volcanii DS2	CP001956	309800	4,042	15	22	4	1	8	4	1	2	1	1
Halogeometricum borinquense DSM 11551	CP001690	469382	3,898	21	25	4	1	8	3	1	3	3	2
Halomicrobium mukohataei DSM 12286	CP001688	485914	3,349	21	25	3	1	8	1	0	9	1	2
Halopiger xanaduensis SH-6	CP002839	797210	4,221	26	18	10	2	2	0	0	2	2	0
Haloquadratum walsbyi DSM 16790	AM180088	768065	2,560	4	6	1	0	3	0	1	0	1	0
Halorhabdus tiamatea SARL4B	HF571520	1033806	3,023	18	17	4	1	7	0	0	4	1	0
Halorhabdus utahensis DSM 12940	CP001687	519442	2,998	31	29	4	1	11	4	0	7	2	0

Halorientalis sp. IM1011	CP019067	1932360	3,746	40	30	3	1	12	3	1	7	2	1
Halorubrum lacusprofundi ATCC 49239	CP001365	416348	3,564	32	9	2	1	2	0	0	3	1	0
Halostagnicola larsenii XH-48	CP007055	797299	4,171	11	15	7	1	2	0	0	1	3	1
Haloterrigena daqingensis	CP019330	588898	3,642	15	16	7	1	1	0	0	2	5	0
Haloterrigena turkmenica DSM 5511	CP001860	543526	5,113	46	30	18	1	3	0	0	4	4	0
Halovivax ruber XH-70	CP003050	797302	3,099	17	10	2	1	3	0	0	3	1	0
Natrialba magadii ATCC 43099	CP001932	547559	4,215	16	22	10	2	2	0	1	2	4	1
Natrialbaceae archaeon JW/NM-HA 15	CP019893	1902251	3,642	29	22	6	1	7	1	2	2	2	1
Natrinema pellirubrum DSM 15624	CP003372	797303	4,199	22	20	6	1	7	0	0	2	4	0
Natrinema sp. J7-2	CP003412	406552	3595	10	15	6	1	3	0	0	2	3	0
Natronobacterium gregoryi SP2	CP003377	797304	3,656	6	15	6	2	1	0	1	1	4	0
Natronococcus occultus SP4	CP003929	694430	4,154	23	25	9	0	9	0	0	2	5	0
Natronomonas moolapensis 8.8.11	HF582854	268739	2,749	24	26	3	1	10	2	1	4	4	1
Natronomonas pharaonis DSM 2160	CR936257	348780	2,822	24	22	4	1	12	2	0	2	1	0
Salinarchaeum sp. Harcht-Bsk1	CP005962	1333523	3,013	23	18	2	1	9	1	0	4	0	1
halophilic archaeon DL31[a]	CP002988	756883	3,476	13	6	1	0	3	0	0	1	1	0
**Methanobacteria**			**33,023**	**140**	**150**	**35**	**0**	**38**	**28**	**0**	**0**	**0**	**49**
Methanobacterium congolense	LT607756	118062	2,284	10	9	2	0	4	1	0	0	0	2
Methanobacterium formicicum BRM9	CP006933	2162	2,342	23	36	7	0	6	9	0	0	0	14
Methanobacterium lacus AL-21	CP002551	877455	2,451	29	33	8	0	9	9	0	0	0	7
Methanobacterium paludis SWAN-1	CP002772	868131	2,367	10	7	4	0	0	1	0	0	0	2
Methanobacterium sp. MB1	HG425166	1379702	2013	14	18	7	0	4	3	0	0	0	4
Methanobrevibacter millerae	CP011266	230361	2,270	3	2	1	0	0	0	0	0	0	1
Methanobrevibacter olleyae	CP014265	294671	1,834	0	0	0	0	0	0	0	0	0	0
Methanobrevibacter ruminantium M1	CP001719	634498	2,217	0	0	0	0	0	0	0	0	0	0
Methanobrevibacter smithii ATCC 35061	CP000678	420247	1,793	0	0	0	0	0	0	0	0	0	0
Methanobrevibacter sp. AbM4	CP004050	224719	1,671	1	4	2	0	0	0	0	0	0	2
Methanobrevibacter sp. YE315	CP010834	1609968	1,942	0	0	0	0	0	0	0	0	0	0
Methanosphaera stadtmanae DSM 3091	CP000102	339860	1,534	0	0	0	0	0	0	0	0	0	0

Methanothermobacter marburgensis str. Marburg	CP001710	79929	1,757	11	11	1	0	5	1	0	0	0	4
Methanothermobacter sp. CaT2	AP011952	866790	1,725	12	10	1	0	4	0	0	0	0	5
Methanothermobacter thermautotrophicus str. Delta H	AE000666	187420	1,873	13	10	1	0	3	2	0	0	0	4
Methanothermobacter wolfeii	LT608329	145261	1,665	14	10	1	0	3	2	0	0	0	4
Methanothermus fervidus DSM 2088	CP002278	523846	1,285	0	0	0	0	0	0	0	0	0	0
**Methanococci**			**19,966**	**19**	**23**	**15**	**8**	**0**	**0**	**0**	**0**	**0**	**0**
Methanocaldococcus bathoardescens	CP009149	1301915	1,637	1	2	1	1	0	0	0	0	0	0
Methanocaldococcus fervens AG86	CP001696	573064	1,581	2	3	2	1	0	0	0	0	0	0
Methanocaldococcus infernus ME	CP002009	573063	1,442	1	2	1	1	0	0	0	0	0	0
Methanocaldococcus jannaschii DSM 2661	L77117	243232	1,786	0	0	0	0	0	0	0	0	0	0
Methanocaldococcus sp. FS406-22	CP001901	644281	1,816	1	1	1	0	0	0	0	0	0	0
Methanocaldococcus vulcanius M7	CP001787	579137	1,742	1	2	1	1	0	0	0	0	0	0
Methanococcus aeolicus Nankai-3	CP000743	419665	1,490	1	1	1	0	0	0	0	0	0	0
Methanococcus maripaludis S2	BX950229	402880	1,722	3	3	2	1	0	0	0	0	0	0
Methanococcus vannielii SB	CP000742	406327	1,678	5	3	2	1	0	0	0	0	0	0
Methanococcus voltae A3	CP002057	456320	1,717	2	3	2	1	0	0	0	0	0	0
Methanothermococcus okinawensis IH1	CP002792	647113	1,576	2	3	2	1	0	0	0	0	0	0
Methanotorris igneus Kol 5	CP002737	880724	1,779	0	0	0	0	0	0	0	0	0	0
**Methanomicrobia**			**108,246**	**998**	**697**	**300**	**33**	**175**	**62**	**20**	**0**	**0**	**107**
Methanocella arvoryzae MRE50	AM114193	351160	3,103	45	14	9	1	0	1	0	0	0	3
Methanocella conradii HZ254	CP003243	1041930	2,456	19	9	8	0	0	0	0	0	0	1
Methanocella paludicola SANAE	AP011532	304371	3,004	51	8	6	0	1	0	0	0	0	1
Methanococcoides burtonii DSM 6242	CP000300	259564	2406	26	18	8	1	4	2	1	0	0	2
Methanococcoides methylutens MM1	CP009518	1434104	2,203	27	14	7	0	4	1	1	0	0	1
Methanocorpusculum labreanum Z	CP000559	410358	1,739	1	1	0	0	0	0	0	0	0	1
Methanoculleus bourgensis MS2	HE964772	1201294	2,538	14	7	1	1	4	0	0	0	0	1

Methanoculleus marisnigri JR1	CP000562	368407	2,489	24	10	3	1	4	0	0	0	0	2
Methanoculleus sp. MAB1	LT158599	86622	3,450	15	5	1	1	1	1	0	0	0	1
Methanofollis liminatans DSM 4140	CM001555	28892	2,396	17	20	4	1	9	4	0	0	0	2
Methanohalobium evestigatum Z-7303	CP002069	644295	2,254	28	11	7	1	0	0	0	0	0	3
Methanohalophilus halophilus Z-7982	CP017921	2177	1,987	26	12	7	0	0	1	1	0	0	3
Methanohalophilus mahii DSM 5219	CP001994	547558	1,987	22	13	7	0	2	1	1	0	0	2
Methanolacinia petrolearia DSM 11571	CP002117	679926	2,785	32	30	7	1	10	6	0	0	0	6
Methanolobus psychrophilus R15	CP003083	1094980	2,841	36	20	8	1	5	2	1	0	0	3
Methanomethylovorans hollandica DSM 15978	CP003362	867904	2,525	11	9	4	1	2	1	1	0	0	0
Methanoplanus limicola DSM 2279	CM001436	937775	2,967	32	39	6	1	18	6	0	0	0	8
Methanoregula boonei 6A8	CP000780	456442	2,450	30	63	15	1	22	6	0	0	0	19
Methanoregula formicica SMSP	CP003167	593750	2,816	30	40	5	1	20	8	1	0	0	5
Methanosaeta concilii GP6	CP002565	990316	2,858	8	4	3	0	0	0	0	0	0	1
Methanosaeta harundinacea 6Ac	CP003117	1110509	2,399	2	6	2	0	1	1	1	0	0	1
Methanosaeta thermophila PT	CP000477	349307	1,696	7	4	3	0	0	0	0	0	0	1
Methanosalsum zhilinae DSM 4017	CP002101	679901	1,976	28	9	5	1	0	2	0	0	0	1
Methanosarcina acetivorans C2A	AE010299	188937	4,540	51	17	12	2	2	0	0	0	0	1
Methanosarcina barkeri str. Fusaro	CP000099	269797	3,625	25	12	8	1	1	0	1	0	0	1
Methanosarcina horonobensis HB-1 = JCM 15518	CP009516	1434110	4,095	67	23	17	1	3	0	1	0	0	1
Methanosarcina lacustris Z-7289	CP009515	1434111	3,264	18	10	7	1	0	0	1	0	0	1
Methanosarcina mazei Go1	AE008384	192952	3,371	33	15	10	2	1	0	1	0	0	1
Methanosarcina siciliae C2J	CP009508	1434118	4,401	43	17	11	2	2	0	1	0	0	1
Methanosarcina sp. 795	CP011449	1653396	2,416	15	14	10	1	0	0	1	0	0	2
Methanosarcina sp. Kolksee	CP009524	1434099	3,476	21	15	11	1	1	0	1	0	0	1
Methanosarcina sp. MTP4	CP009505	1434100	3,250	28	16	13	1	1	0	0	0	0	1
Methanosarcina sp. WH1	CP009504	1434102	3,198	22	15	11	1	1	0	1	0	0	1
Methanosarcina sp. WWM596	CP009503	1434103	3,408	22	16	11	1	1	0	1	0	0	2
Methanosarcina thermophila CHTI-55	CP009502	1434121	2,545	21	16	11	1	1	0	2	0	0	1

Methanosarcina vacuolata Z-761	CP009520	1434123	3,538	22	14	11	0	1	0	1	0	0	1
Methanosphaerula palustris E1-9c	CP001338	521011	2,655	33	44	7	2	22	10	0	0	0	3
Methanospirillum hungatei JF-1	CP000254	323259	3,139	46	87	24	2	31	9	0	0	0	21
**Methanopyri**													
Methanopyrus kandleri AV19	AE009439	190192	1,687	0	0	0	0	0	0	0	0	0	0
**Thermococci**			**51,618**	**17**	**31**	**17**	**14**	**0**	**0**	**0**	**0**	**0**	**0**
Palaeococcus pacificus DY20341	CP006019	1343739	1,991	1	2	1	1	0	0	0	0	0	0
Pyrococcus abyssi GE5	AL096836	272844	1,896	1	2	1	1	0	0	0	0	0	0
Pyrococcus furiosus DSM 3638	AE009950	186497	2,125	0	0	0	0	0	0	0	0	0	0
Pyrococcus horikoshii OT3	BA000001	70601	1,955	1	2	1	1	0	0	0	0	0	0
Pyrococcus kukulkanii	CP010835	1609559	1,997	1	2	1	1	0	0	0	0	0	0
Pyrococcus sp. NA2	CP002670	342949	1,980	1	2	1	1	0	0	0	0	0	0
Pyrococcus sp. ST04	CP003534	1183377	1,748	1	2	1	1	0	0	0	0	0	0
Pyrococcus yayanosii CH1	CP002779	529709	1865	1	2	1	1	0	0	0	0	0	0
Thermococcus barophilus MP	CP002372	391623	2,207	1	2	1	1	0	0	0	0	0	0
Thermococcus chitonophagus	LN999010	54262	2,118	0	0	0	0	0	0	0	0	0	0
Thermococcus cleftensis	CP003651	163003	2,023	0	0	0	0	0	0	0	0	0	0
Thermococcus eurythermalis	CP008887	1505907	2,095	1	2	1	1	0	0	0	0	0	0
Thermococcus gammatolerans EJ3	CP001398	593117	2,157	1	1	1	0	0	0	0	0	0	0
Thermococcus guaymasensis DSM 11113	KC262637	110164	2,119	1	1	1	0	0	0	0	0	0	0
Thermococcus kodakarensis KOD1	AP006878	69014	2,306	1	2	1	1	0	0	0	0	0	0
Thermococcus litoralis DSM 5473	CP006670	523849	2506	1	2	1	1	0	0	0	0	0	0
Thermococcus nautili	CP007264	195522	2,284	1	2	1	1	0	0	0	0	0	0
Thermococcus onnurineus NA1	CP000855	523850	1,976	1	2	1	1	0	0	0	0	0	0
Thermococcus paralvinellae	CP006965	582419	2,023	0	0	0	0	0	0	0	0	0	0
Thermococcus peptonophilus	AF239996	523851	1,972	1	2	1	1	0	0	0	0	0	0
Thermococcus piezophilus	CP015520	1712654	1,867	0	0	0	0	0	0	0	0	0	0
Thermococcus sibiricus MM 739	CP001463	604354	2,036	0	0	0	0	0	0	0	0	0	0

Thermococcus sp. 2319x1	CP012200	1674923	2017	0	0	0	0	0	0	0	0	0	0
Thermococcus sp. 4557	CP002920	1042877	2133	0	0	0	0	0	0	0	0	0	0
Thermococcus sp. AM4	CP002952	246969	2,222	1	1	1	0	0	0	0	0	0	0
**Thermoplasmata**			**18,221**	**3**	**4**	**0**	**0**	**0**	**0**	**1**	**0**	**0**	**3**
Candidatus Methanomassiliicoccus intestinalis Issoire-Mx1	CP005934	1295009	1820	0	0	0	0	0	0	0	0	0	0
Candidatus Methanomethylophilus alvus Mx1201	CP004049	1236689	1643	0	1	0	0	0	0	0	0	0	1
Candidatus Methanoplasma termitum MpT1	CP010070	1577791	1,404	0	1	0	0	0	0	0	0	0	1
Cuniculiplasma divulgatum	LT719092	1673428	1802	0	0	0	0	0	0	0	0	0	0
Ferroplasma acidarmanus fer1	CP004145	333146	1927	0	0	0	0	0	0	0	0	0	0
Ferroplasma acidiphilum	CP015363	74969	1,764	0	0	0	0	0	0	0	0	0	0
Methanogenic archaeon ISO4-H5	CP014214	1495144	1,794	3	1	0	0	0	0	1	0	0	0
Picrophilus torridus DSM 9790	AE017261	263820	1,535	0	0	0	0	0	0	0	0	0	0
Thermoplasma acidophilum DSM 1728	AL139299	273075	1,481	0	0	0	0	0	0	0	0	0	0
Thermoplasma volcanium GSS1	BA000011	273116	1,523	0	0	0	0	0	0	0	0	0	0
Thermoplasmatales archaeon BRNA1	CP002916	1054217	1,528	0	1	0	0	0	0	0	0	0	1
**unclassified Euryarchaeota**			**4,814**	**4**	**8**	**6**	**0**	**0**	**0**	**0**	**0**	**0**	**2**
Aciduliprofundum boonei T469	CP001941	439481	1539	2	4	3	0	0	0	0	0	0	1
Aciduliprofundum sp. MAR08-339	CP003168	673860	1494	2	4	3	0	0	0	0	0	0	1
uncultured marine group II euryarchaeote	CM001443	274854	1,781	0	0	0	0	0	0	0	0	0	0
**Nanoarchaeota**			**1,122**										
Candidatus Nanopusillus acidilobi	CP010514	1577684	587	0	0	0	0	0	0	0	0	0	0
Nanoarchaeum equitans Kin4-M	AE017199	228908	535	0	0	0	0	0	0	0	0	0	0

**Thaumarchaeota**			36,321	175	224	172	7	1	0	1	0	0	43
Candidatus Caldiarchaeum subterraneum	BA000048	311458	1,730	0	0	0	0	0	0	0	0	0	0
Candidatus Nitrocosmicus oleophilus	CP012850	1353260	3,725	22	24	16	0	0	0	0	0	0	8
Candidatus Nitrosoarchaeum limnia SFB1	CM001158	1007084	2,038	26	33	28	1	1	0	0	0	0	3
Candidatus Nitrosomarinus catalina	CP021324	1898749	1,677	0	1	0	0	0	0	0	0	0	1
Candidatus Nitrosopelagicus brevis	CP007026	1410606	1,445	0	0	0	0	0	0	0	0	0	0
Candidatus Nitrosopumilus adriaticus	CP011070	1580092	2,184	22	34	28	1	0	0	0	0	0	5
Candidatus Nitrosopumilus koreensis AR1	CP003842	1229908	1,890	12	15	12	0	0	0	0	0	0	3
Candidatus Nitrosopumilus piranensis	CP010868	1582439	2,161	2	3	1	0	0	0	0	0	0	2
Candidatus Nitrosopumilus sediminis	CP003843	1229909	1,974	6	9	5	0	0	0	0	0	0	4
Candidatus Nitrososphaera evergladensis SR1	CP007174	1459636	3,499	19	19	12	1	0	0	0	0	0	6
Candidatus Nitrososphaera gargensis Ga9.2	CP002408	1237085	3,565	19	31	25	1	0	0	0	0	0	5
Candidatus Nitrosotalea devanaterra	LN890280	1078905	2103	19	19	16	1	0	0	0	0	0	2
Candidatus Nitrosotenuis cloacae	CP011097	1603555	1,717	6	15	12	1	0	0	0	0	0	2
Cenarchaeum symbiosum A	DP000238	414004	2,017	0	0	0	0	0	0	0	0	0	0
Nitrosopumilus maritimus SCM1	CP000866	436308	1,795	12	10	9	0	0	0	0	0	0	1
Nitrososphaera viennensis EN76	CP007536	926571	2801	10	11	8	1	0	0	1	0	0	1
**unclassified Archaea**			**5,604**	**0**	**0**	**0**	**0**	**0**	**0**	**0**	**0**	**0**	**0**
archaeon GW2011_AR10	CP010424	1579370	1,339	0	0	0	0	0	0	0	0	0	0
archaeon GW2011_AR15	CP010425	1579373	1,308	0	0	0	0	0	0	0	0	0	0
archaeon GW2011_AR20	CP010426	1579378	1,010	0	0	0	0	0	0	0	0	0	0
Uncultured archaeon clone ASS_A1	CP009479	115547	1,947	0	0	0	0	0	0	0	0	0	0
**TOTAL**	**218**		**511,588**	**2,139**	**1,875**	**740**	**102**	**445**	**141**	**33**	**110**	**81**	**222**

[a] – Based in its 16S rRNA sequence, halophilic archaeaon DL31 belongs to the family *Halorubraceae*, class *Halobacteria*.

Table S5. Details of the sequence analysis of the newly described archaeal domains

Domain name	Query ID (GenBank entry, aa boundaries)	Program used[a]	Hit ID and its annotation	HHpred probablity (Pr) or PSI-BLAST E-value (E)	Comment
AcidOD1	MSA of four sequences related to ADD08891.1 (122-194 aa)	HHpred	PDB: 4UN1_A; Putative transcrip-tional regulator, AsnC family (126-165 aa)	Pr= 71.79	Partial overlap with the ligand binding domain of the Lrp/AsnC family regulators
HalOD2	ACV46423.1 (114-195 aa)	HHpred	PF08663.9, HalX domain	Pr=85.79	A HalX-related domain, followed by a Zn-finger
	ACV46423.1 (196-253 aa)	HHpred	PF13240.5, Zinc-ribbon domain	Pr=96.38	
MetOD1	AAM05831.1 (133-292 aa)	HHpred	PDB: 5FRW_A; Phenol-responsive sensory domain of PoxR. TIGR02019; BchJ; Bacteriochlorophyll 4-vinyl reductase	Pr=98.52	Related to the V4R (PF02830) hydrocarbon-binding domain
MetOD3	ABS55483.1 (20-200 aa)	TMHMM	Transmembrane helix, at positions 174..196		Membrane-anchored Ig-like domain, possibly involved in sugar binding (lectin-like). Ig-like domains are often seen in membrane- and S-layer-associated proteins
		HHpred	PF10648.8, Gmad2; Immunoglobulin-like domain of bacterial spore germination	Pr=53.38	
NitrOD1	AFS82515.1 (114-198 aa)	PSI-BLAST	KEQ56625.1, AsnC family transcriptional regulator protein	E=0.67	Related to the ligand binding domain of the Lrp/AsnC family regulators
NitrOD2	AIC16890.1 (1-100 aa)	PSI-BLAST, HHPred	AFU59294.1, hypothetical protein Ngar_c23690	E=0.023	Second iteration of the PSI-BLAST (with E=0.1 inclusion treshold) returns a hit to the Ngar_c23690 protein. When Ngar_c23690 is used as query for an HHpred search, it reveals similarity with the DUF3227 domain (Pr= 88.03%), which was recently shown to belong to the archaeal Death domain-like family (1)
NitrOD3	AIF83507.1 (1-142 aa)	HHpred	PDB: 3KYE_A; Roadblock/LC7 domain	Pr= 68.3	Related to the Roadblock/LC7 domain, expansion in Thaumarchaea
NitrOD4	AFS80878.1 (1-96 aa)	PSI-BLAST	AFU59889.1, hypothetical protein Ngar_c29710 (194-263 aa)	E=0.003	Second iteration of the PSI-BLAST returns a hit to the Ngar_c29710 protein. When used as query for HHpred, this protein reveals similarity with the DUF3227 family (Pr=97%), which was recently shown to belong to the archaeal Death domain-like family (1)

NitrOD5	ABX12603.1 (127-216 aa)	HHpred	PF11537.7; Protein of unknown function (DUF3227)	Pr=95.52	DUF3227 family was shown to belong to archaeal Death domain-like family (1)
TackOD1	AFS80801.1 (126-318 aa)	HHPred	PDB: 2JNE_A; *E. coli* Yfgj bound to two Zn^{+2}. PF12773; Double zinc ribbon domain	Pr=97.56	Similar to the Double zinc ribbon (PF12773) domain, in bacteria often fused to the GGDEF (diguanylate cyclase) domains

[a] - PSI_BLAST searches were run against NCBI's non-redundant protein database with the following parameters: inclusion treshold E= 0.01; taxonomy restriction: "archaea" only; no low complexity filtering; composition based statistics: off, unless stated otherwise in comments. HHpredwas ru with default parameters with three HHblits interations, against PDB, Pfam and CDD database. TMHHM 2.0 was run with default parameters.

Reference

1. **Makarova KS, Galperin MY, Koonin EV.** 2017. Proposed role for KaiC-like ATPases as major signal transduction hubs in Archaea. mBio01959-17, in press.

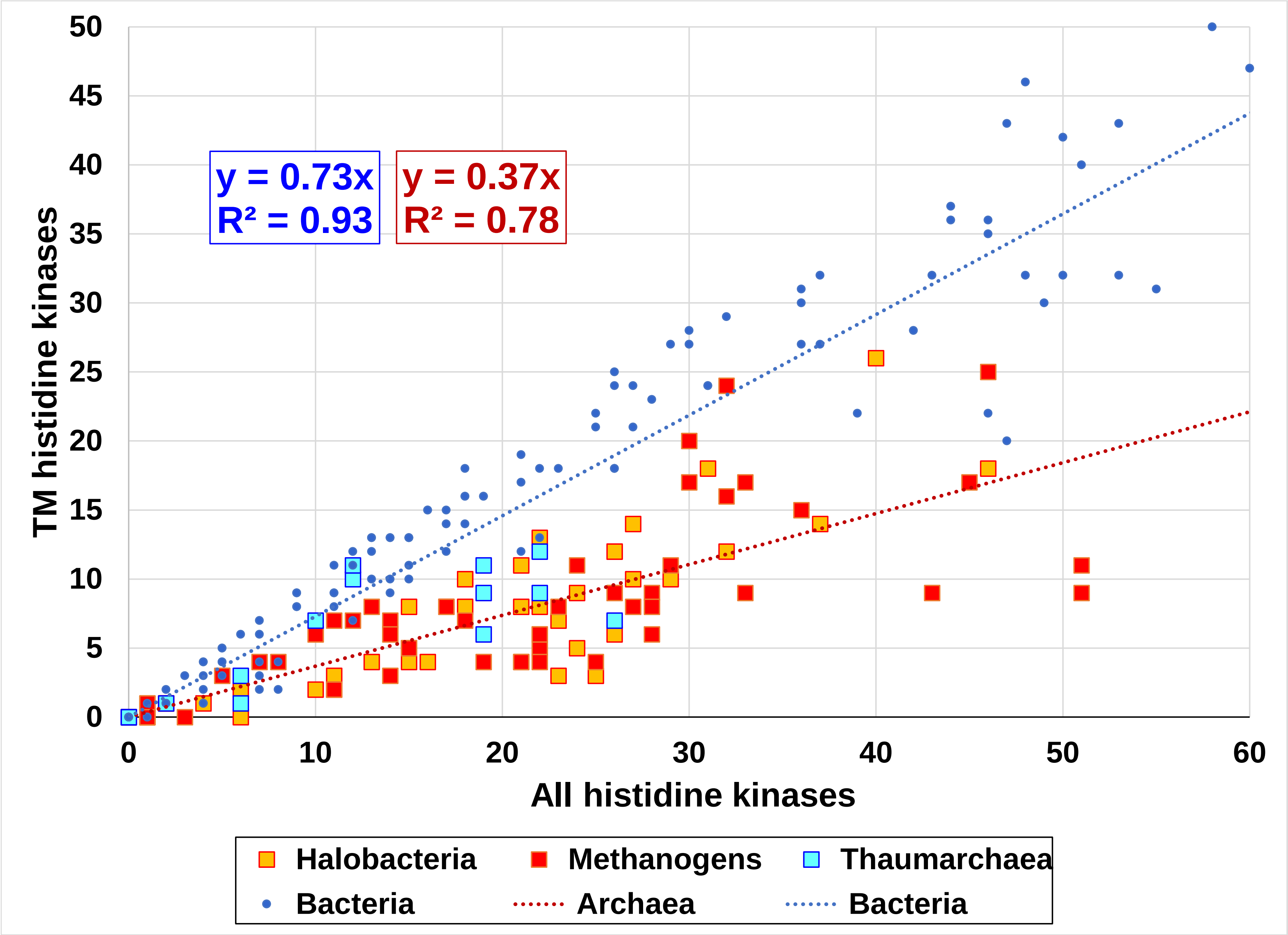

Figure S1. Transmembrane histidine kinase content in bacteria and archaea

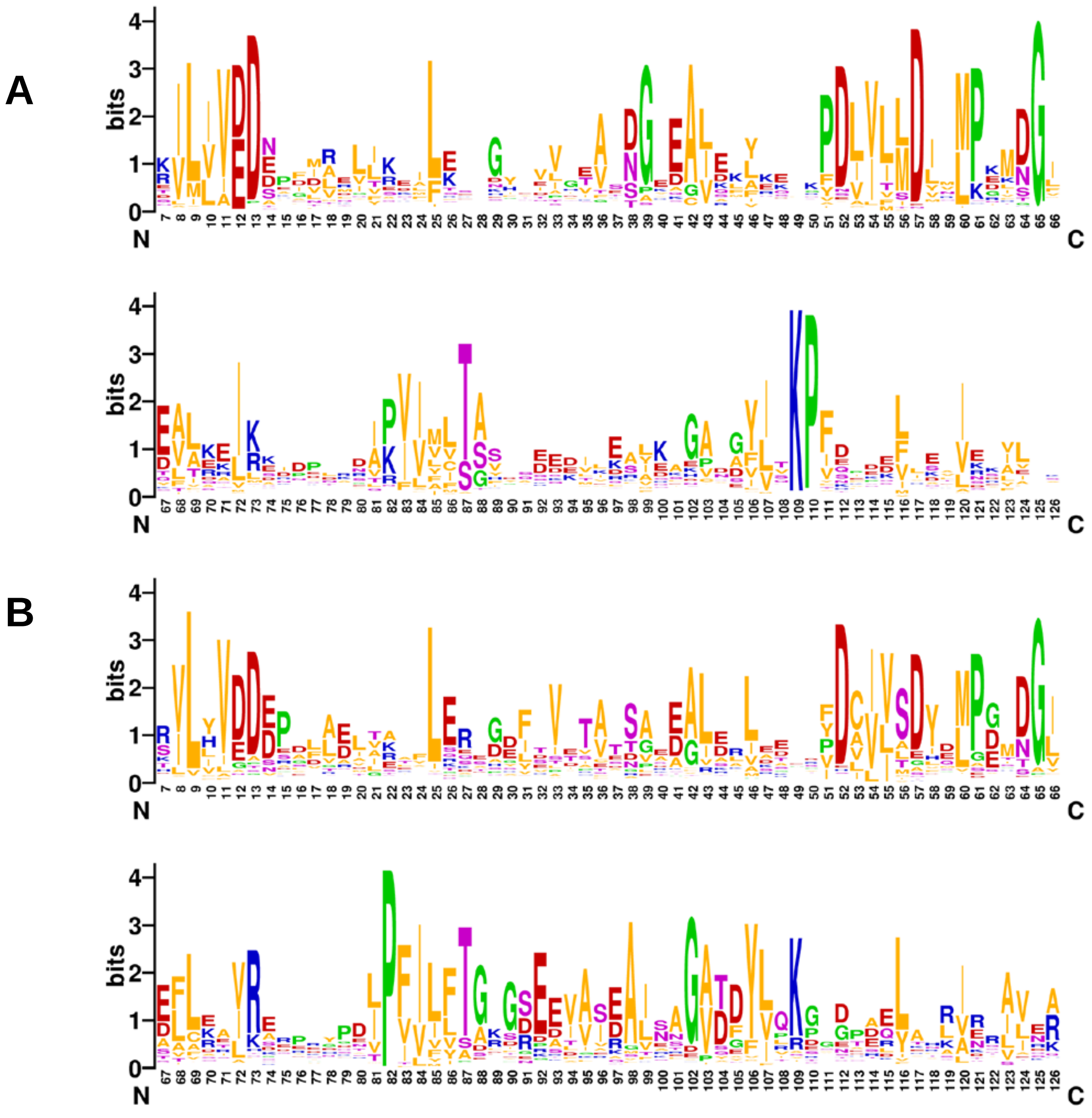

Figure S2. Sequence conservation in archaeal receiver domains.

A, response regulators with stand-alone REC domains;

B, REC domains of the response regulators with the REC-(PAS/GAF)$_n$-HisKA-HATPase domain architectures

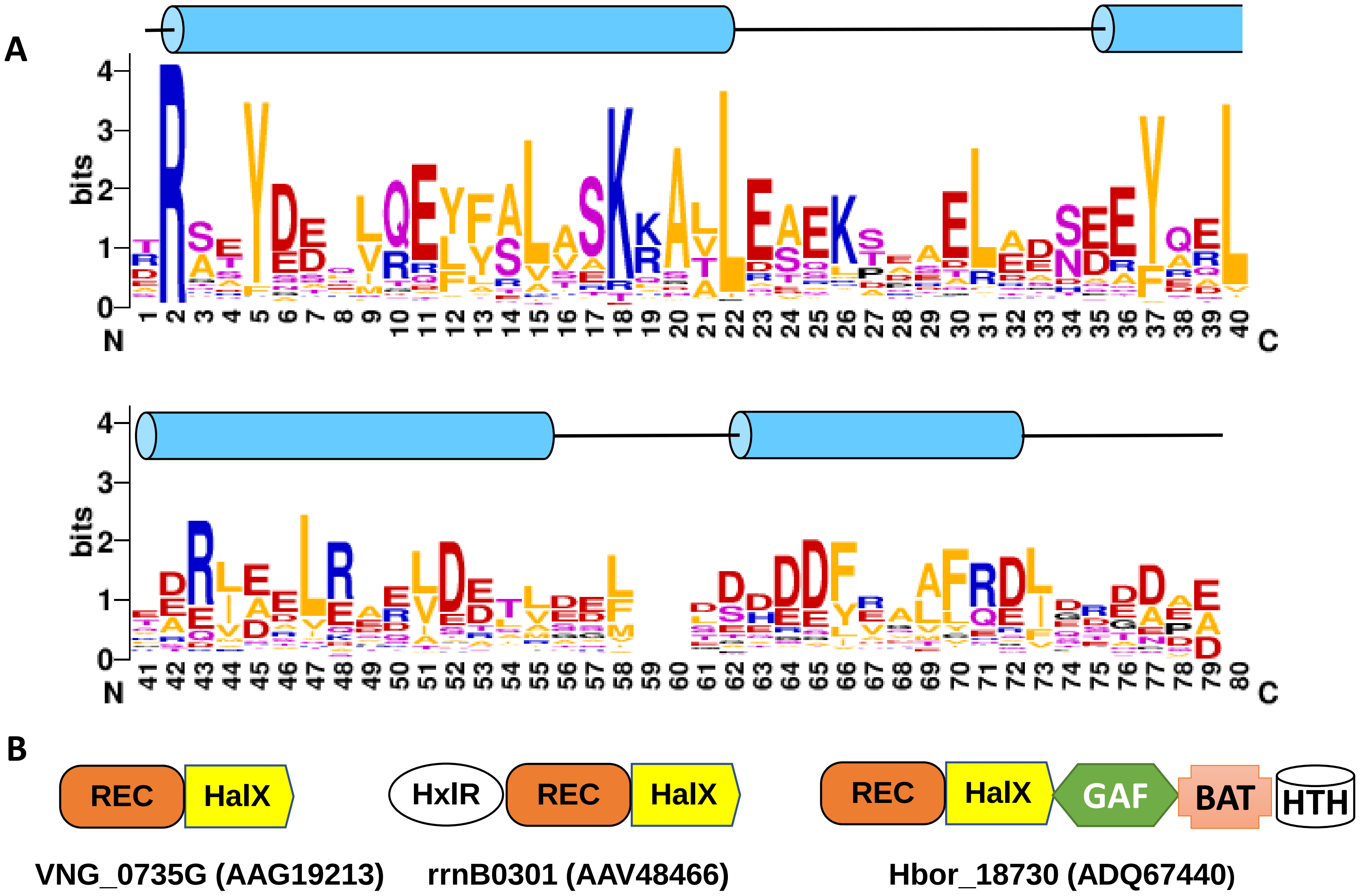

Figure S3. Sequence conservation and domain architectures of the HalX (PF08663) domain.
A. Sequence logo of HalX, blue cylinders on top indicate α-helices predicted by the JPred program.
B. Examples of HalX domain architectures

A

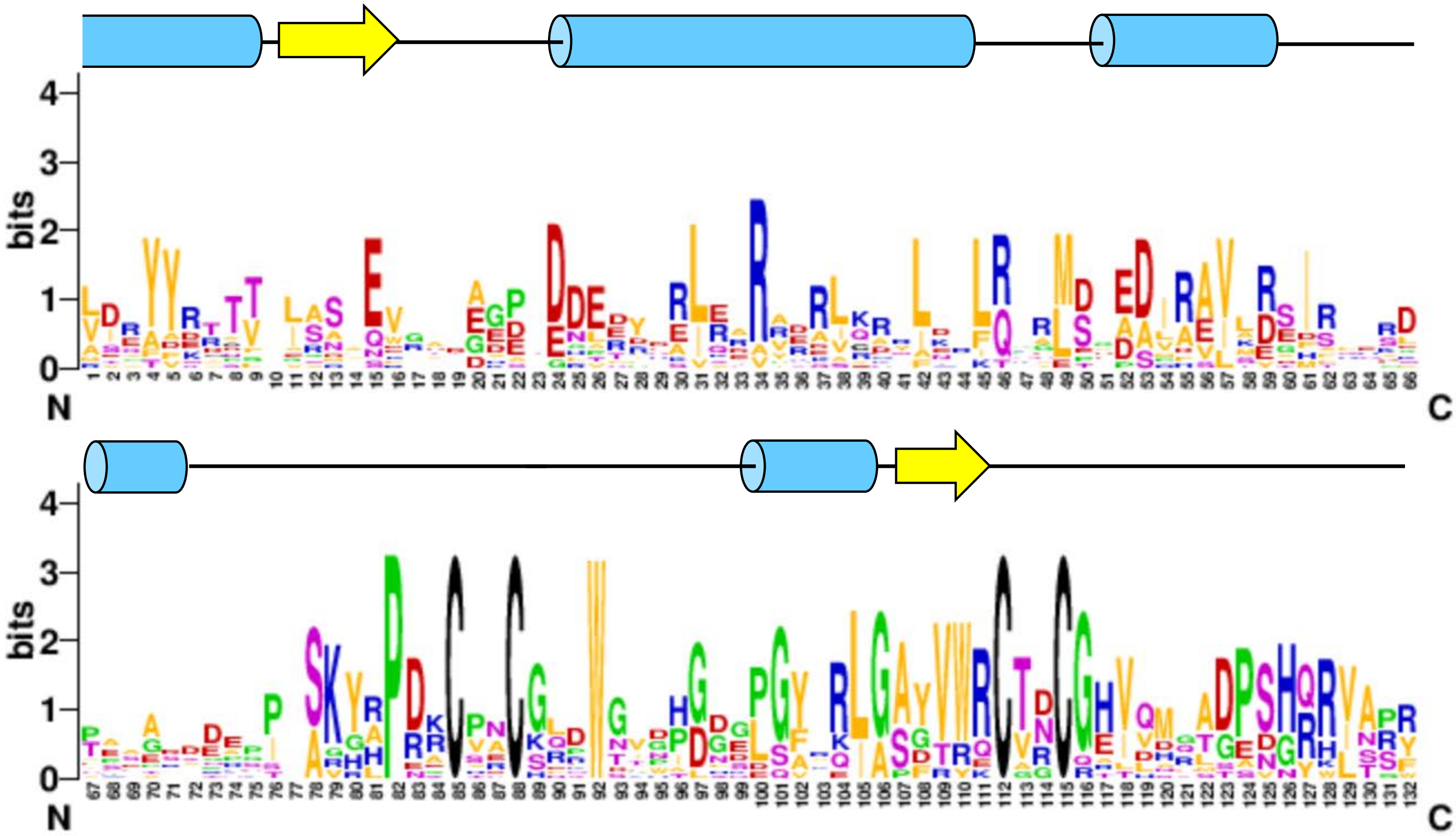

Figure S4. **Sequence conservation in the HalOD2 domain.** Residue numbering is based on the sequence of the response regulator Hmuk_0286 from *Halomicrobium mukohataei*. Logo position 1 corresponds to Leu122 of Hmuk_0286 (GenBank: ACV46423). Blue cylinders and yellow arrows indicate α-helices and β-strands, respectively, predicted by the JPred program.

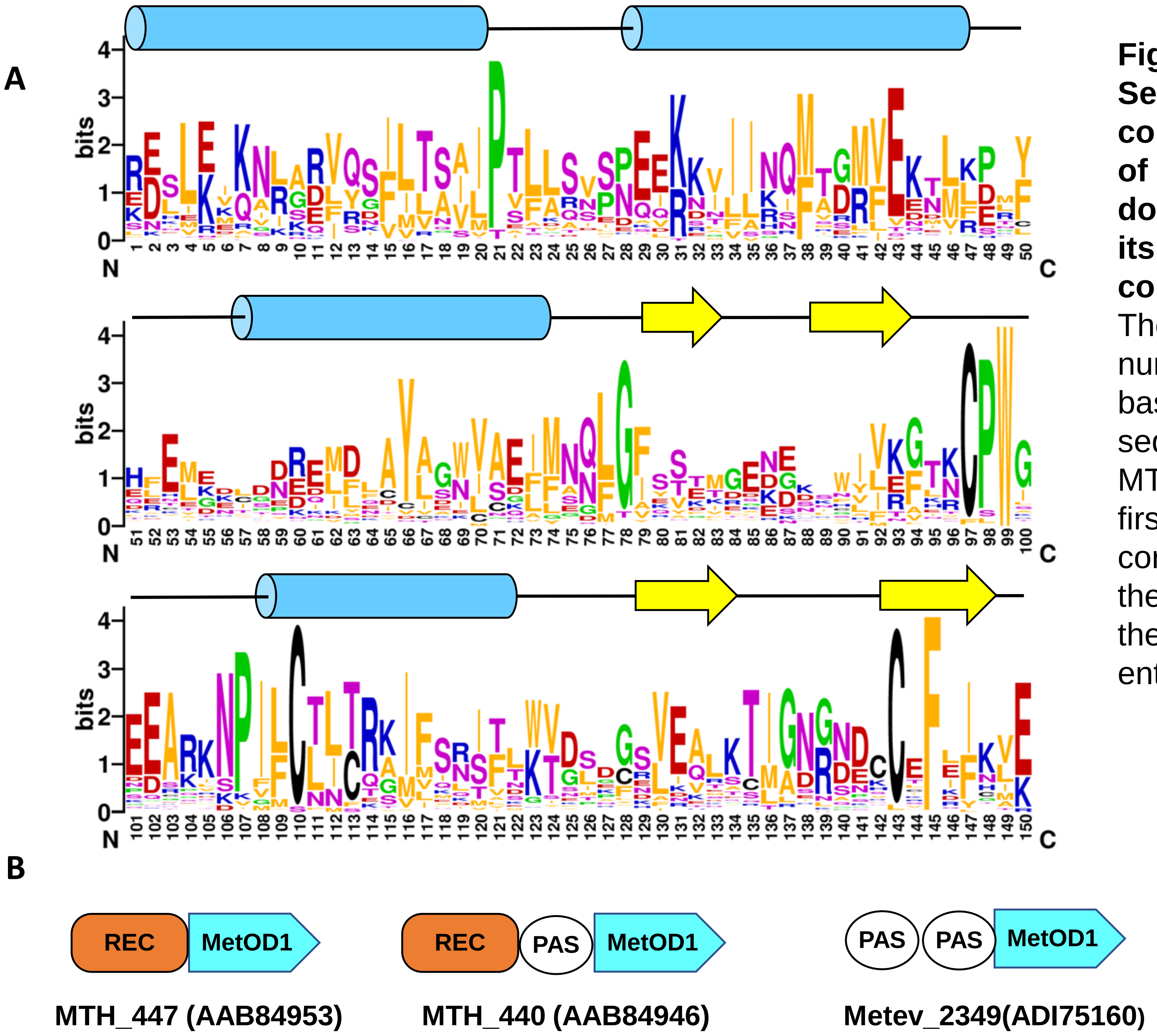

Figure S5. Sequence conservation of the MetOD1 domain and its domain combinations. The residue numbering is based on the sequence of MTH_447, the first position corresponds to the Lys128 of the GenBank entry AAB84953

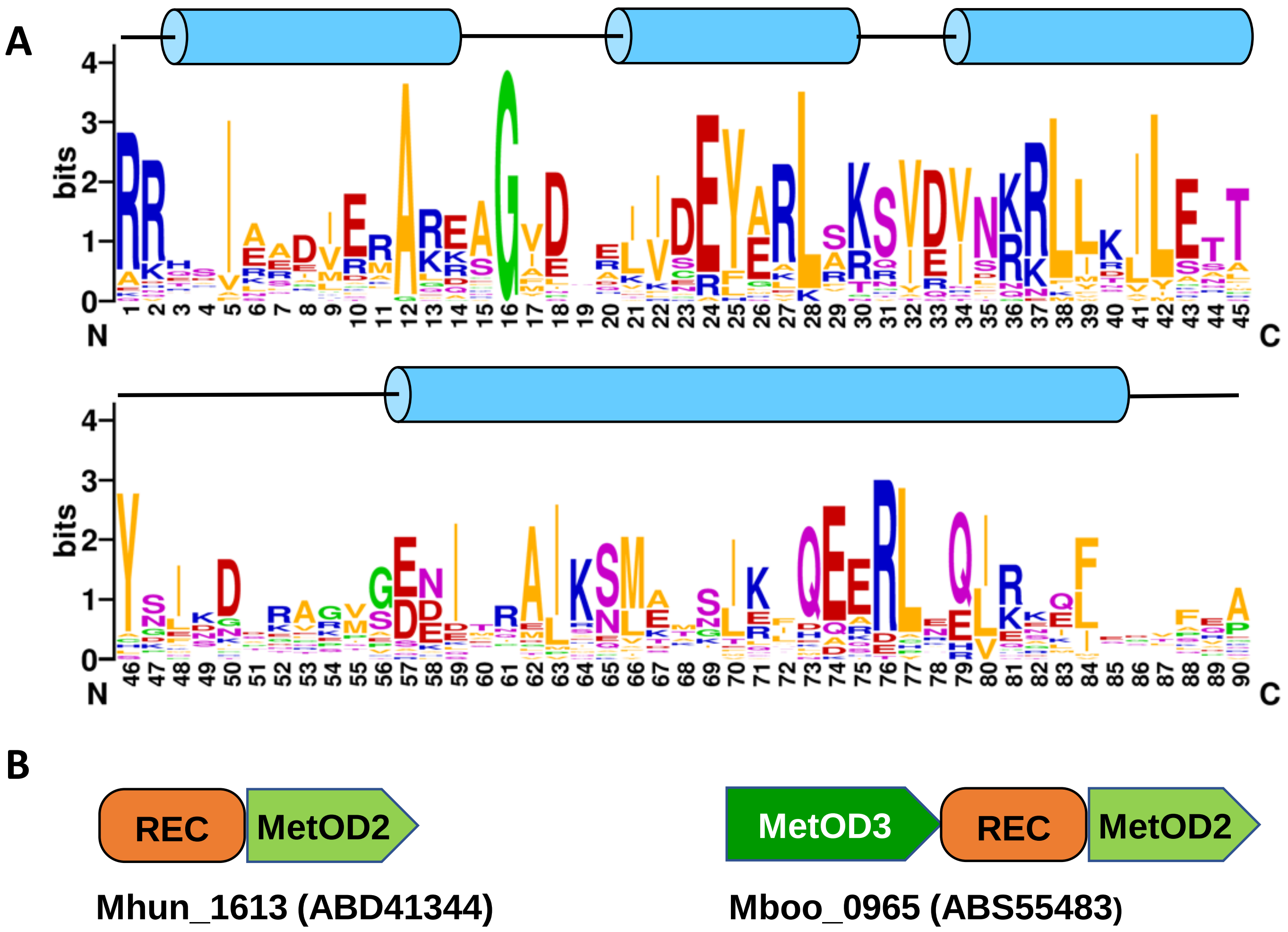

Figure S6. Sequence conservation of the MetOD2 domain and its domain combinations. The residue numbering is based on the sequence of Mhun_1613, the first position corresponds to Arg124 of the GenBank entry ABD41344. The cylinders on top indicate α-helices predicted by JPred.

A

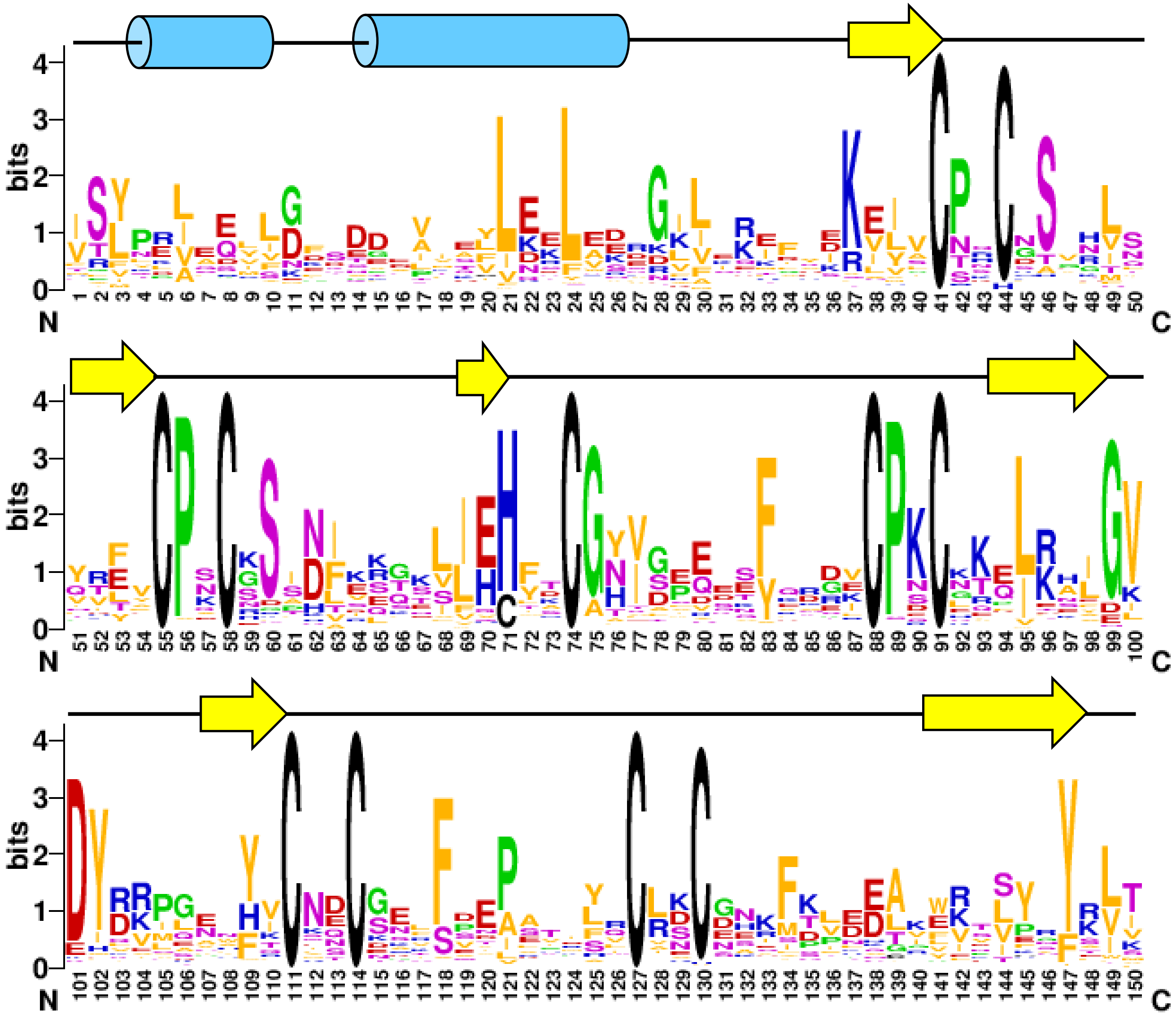

Figure S7. Sequence conservation of the TackOD1 domain. The residue numbering is based on the sequence of NKOR_04565 protein from *Candidatus* Nitrosopumilus koreensis AR1, the first position corresponds to Ile167 of the GenBank entry AFS80801
