# Supplementary material for: Phyletic Distribution and Lineage-Specific Domain Architectures of Archaeal Two-Component Signal Transduction Systems
Source: J Bacteriol. 2018 Mar 12;200(7):e00681-17. doi: 10.1128/JB.00681-17 (PMC5847659; doi:10.1128/JB.00681-17)
Supplement: Supplemental material [file JB.00681-17_zjb999094653s2.pdf]

Table S2. Histidine kinases and response regulators encoded in complete genomes of Archaea

**Archaea**	
**Candidatus Micrarchaeota**	
. Candidatus Micrarchaeota archaeon Mia14	1 HK, 1 RR
**Candidatus Nanohaloarchaeota**	
. Nanohaloarchaea archaeon SG9...........................	0 hits
**Candidatus Korarchaeota**	
. Candidatus Korarchaeum cryptofilum OPF8................	0 hits
**Crenarchaeota**	
44 completely sequenced genomes	0 hits
**Euryarchaeota**	
. Aciduliprofundum boonei T469	2 HKs, 4 RRs
. Aciduliprofundum sp. MAR08-339i........................	2 HKs, 4 RRs
**Archaeoglobi**	
. Archaeoglobus fulgidus DSM 4304	13 HKs, 12 RRs
. Archaeoglobus profundus DSM 5631	3 HKs, 7 RRs
. Archaeoglobus sulfaticallidus PM70-1i..................	4 HKs, 5 RRs
. Archaeoglobus veneficus SNP6i..........................	4 HKs, 3 RRs
. Ferroglobus placidus DSM 10642	4 HKs, 4 RRs
. Geoglobus acetivorans..................................	5 HKs, 8 RRs
. Geoglobus ahangari.....................................	4 HKs, 3 RRs
**Halobacteria**	
. Halalkalicoccus jeotgali B3	6 HKs, 14 RRs
. Halanaeroarchaeum sulfurireducens	27 HKs, 8 RRs
. Haloarcula hispanica ATCC 33960	26 HKs, 35 RRs
. Haloarcula marismortui ATCC 43049	37 HKs, 45 RRs
. Haloarcula sp. CBA1115	26 HKs, 36 RRs
. Halobacterium hubeiense	18 HKs, 19 RRs
. Halobacterium salinarum NRC-1	11 HKs, 9 RRs
. Halobacterium sp. DL1	18 HKs, 17 RRs
. Halobiforma lacisalsi AJ5	25 HKs, 22 RRs
. Halodesulfurarchaeum formicicum	15 HKs, 6 RRs
. Haloferax gibbonsii ARA6	27 HKs, 22 RRs
. Haloferax mediterranei ATCC 33500	22 HKs, 14 RRs
. Haloferax volcanii DS2	15 HKs, 22 RRs
. Halogeometricum borinquense DSM 11551	21 HKs, 25 RRs
. Halomicrobium mukohataei DSM 12286	21 HKs, 24 RRs
. Halopiger xanaduensis SH-6	26 HKs, 18 RRs
. Haloquadratum walsbyi DSM 16790	4 HKs, 5 RRs
. Halorhabdus tiamatea SARL4B	18 HKs, 17 RRs
. Halorhabdus utahensis DSM 12940	31 HKs, 29 RRs
. Halorientalis sp. IM1011	40 HKs, 29 RRs
. Halorubrum lacusprofundi ATCC 49239	32 HKs, 9 RRs
. Halostagnicola larsenii XH-48	11 HKs, 15 RRs
. Haloterrigena daqingensis	15 HKs, 16 RRs
. Haloterrigena turkmenica DSM 5511	46 HKs, 30 RRs
. Halovivax ruber XH-70	17 HKs, 10 RRs
. Natrialba magadii ATCC 43099	16 HKs, 23 RRs
. Natrialbaceae archaeon JW/NM-HA 15	29 HKs, 22 RRs
. Natrinema pellirubrum DSM 15624	22 HKs, 18 RRs
. Natrinema sp. J7-2	10 HKs, 15 RRs
. Natronobacterium gregoryi SP2	6 HKs, 15 RRs
. Natronococcus occultus SP4	23 HKs, 25 RRs
. Natronomonas moolapensis 8.8.11	24 HKs, 26 RRs
. Natronomonas pharaonis DSM 2160	24 HKs, 22 RRs
. Salinarchaeum sp. Harcht-Bsk1	23 HKs, 16 RRs
. halophilic archaeon DL31	13 HKs, 6 RRs
**Methanobacteria**	
. Methanobacterium congolense	10 HKs, 9 RRs
. Methanobacterium formicicum BRM9	23 HKs, 35 RRs
. Methanobacterium lacus AL-21...........................	29 HKs, 33 RRs
. Methanobacterium paludis SWAN1.........................	10 HKs, 7 RRs
. Methanobacterium sp. MB1	14 HKs, 18 RRs
. Methanobrevibacter millerae............................	3 HKs, 2 RRs
. Methanobrevibacter olleyae	0 hits
. Methanobrevibacter ruminantium M1	0 hits
. Methanobrevibacter smithii ATCC 35061..................	0 hits
. Methanobrevibacter sp. AbM4............................	1 HK, 4 RRs
. Methanobrevibacter sp. YE315	0 hits
. Methanosphaera stadtmanae DSM 3091.....................	0 hits
. Methanothermobacter marburgensis str. Marburg	11 HKs, 11 RRs
. Methanothermobacter thermautotrophicus str. Delta H ...	12 HKs, 8 RRs
. Methanothermobacter wolfeii............................	13 HKs, 10 RRs
. Methanothermobacter sp. CaT2...........................	14 HKs, 10 RRs
. Methanothermus fervidus DSM 2088.......................	0 hits
**Methanococci**	
. Methanocaldococcus bathoardescens......................	1 HK, 2 RRs

. Methanocaldococcus fervens AG86 .... 2 HKs, 3 RRs
. Methanocaldococcus infernus ME .... 1 HK, 2 RRs
. Methanocaldococcus jannaschii DSM 2661 .... 0 hits
. Methanocaldococcus sp. FS406-22 .... 1 HK, 1 RR
. Methanocaldococcus vulcanius M7 .... 1 HK, 2 RRs
. Methanococcus aeolicus Nankai-3 .... 1 HK, 1 RR
. Methanococcus maripaludis S2 .... 3 HKs, 3 RRs
. Methanococcus vannielii SB .... 5 HKs, 3 RRs
. Methanococcus voltae A3 .... 2 HKs, 3 RRs
. Methanothermococcus okinawensis......................... 2 HKs, 3 RRs
. Methanotorris igneus.................................... 0 hits

Methanomicrobia

. Methanocella arvoryzae MRE50 .... 45 HKs, 14 RRs
. Methanocella conradii HZ254............................. 19 HKs, 9 RRs
. Methanocella paludicola SANAE .... 51 HKs, 7 RRs
. Methanococcoides burtonii DSM 6242 .... 26 HKs, 18 RRs
. Methanococcoides methylutens MM1........................ 27 HKs, 12 RRs
. Methanocorpusculum labreanum Z .... 1 HK, 1 RR 1
. Methanoculleus bourgensis MS2........................... 14 HKs, 5 RRs
. Methanoculleus marisnigri JR1 .... 24 HKs, 7 RRs
. Methanoculleus sp. MAB1................................. 15 HKs, 4 RRs
. Methanofollis liminatans DSM 4140....................... 17 HKs, 18 RRs
. Methanohalobium evestigatum Z-7303 .... 28 HKs, 11 RRs
. Methanohalophilus halophilus strain Z-7982............. 26 HKs, 12 RRs
. Methanohalophilus mahii DSM 5219 .... 22 HKs, 11 RRs
. Methanolacinia petrolearia DSM 11571 .... 32 HKs, 19 RRs
. Methanolobus psychrophilus R15.......................... 36 HKs, 18 RRs
. Methanomethylovorans hollandica DSM 15978............... 11 HKs, 8 RRs
. Methanoplanus limicola DSM 2279......................... 32 HKs, 30 RRs
. Methanoregula boonei 6A8 .... 30 HKs, 63 RRs
. Methanoregula formicica................................. 30 HKs, 38 RRs
. Methanosaeta concilii GP6............................... 8 HKs, 4 RRs
. Methanosaeta harundinacea............................... 2 HKs, 6 RRs
. Methanosaeta thermophila PT .... 7 HKs, 4 RRs
. Methanosalsum zhilinae.................................. 28 HKs, 9 RRs
. Methanosarcina acetivorans C2A .... 51 HKs, 15 RRs
. Methanosarcina barkeri str. fusaro .... 25 HKs, 11 RRs
. Methanosarcina horonobensis............................. 67 HKs, 9 RRs
. Methanosarcina lacustris................................ 18 HKs, 16 RRs
. Methanosarcina mazei Go1 .... 33 HKs, 14 RRs
. Methanosarcina siciliae................................. 43 HKs, 16 RRs
. Methanosarcina sp. 795.................................. 15 HKs, 14 RRs
. Methanosarcina sp. Kolksee.............................. 21 HKs, 16 RRs
. Methanosarcina sp. MTP4................................. 28 HKs, 16 RRs
. Methanosarcina sp. WH1.................................. 22 HKs, 15 RRs
. Methanosarcina sp. WWM596............................... 22 HKs, 16 RRs
. Methanosarcina thermophila CHTI-55 .... 21 HKs, 16 RRs
. Methanosarcina vacuolata Z-761.......................... 22 HKs, 14 RRs
. Methanosphaerula palustris E1-9c .... 33 HKs, 44 RRs
. Methanospirillum hungatei JF-1 .... 46 HKs, 87 RRs

Methanopyri

. Methanopyrus kandleri AV19.............................. 0 hits

Thermococci

. Palaeococcus pacificus DY20341.......................... 1 HK, 2 RRs
. Pyrococcus abyssi GE5 .... 1 HK, 2 RRs
. Pyrococcus furiosus DSM 3638............................ 0 hits
. Pyrococcus horikoshii OT3 .... 1 HK, 2 RRs
. Pyrococcus kukulkanii................................... 1 HK, 2 RRs
. Pyrococcus sp. NA2...................................... 1 HK, 2 RRs
. Pyrococcus sp. ST04..................................... 1 HK, 2 RRs
. Pyrococcus yayanosii CH1................................ 1 HK, 2 RRs
. Thermococcus barophilus................................. 1 HK, 2 RRs
. Thermococcus chitonophagus.............................. 0 hits
. Thermococcus cleftensis................................. 0 hits
. Thermococcus eurythermalis.............................. 1 HK, 2 RRs
. Thermococcus gammatolerans EJ3 .... 1 HK, 1 RR
. Thermococcus guaymasensis DSM 11113..................... 1 HK, 1 RR
. Thermococcus kodakarensis KOD1 .... 1 HK, 2 RRs
. Thermococcus litoralis DSM 5473......................... 1 HK, 2 RRs
. Thermococcus nautili.................................... 1 HK, 2 RRs
. Thermococcus onnurineus NA1 .... 1 HK, 2 RRs
. Thermococcus paralvinellae.............................. 0 hits
. Thermococcus peptonophilus JCM 9653..................... 1 HK, 2 RRs
. Thermococcus piezophilus................................ 0 hits
. Thermococcus sibiricus MM 739........................... 0 hits
. Thermococcus sp. 2319x1................................. 0 hits
. Thermococcus sp. 4557................................... 0 hits
. Thermococcus sp. AM4.................................... 1 HK, 1 RR

Thermoplasmata

. Ca. Methanomassiliicoccus intestinalis Issoire-Mx1.....   0 hits
. Ca. Methanomethylophilus alvus Mx1201.................   0 HKs, 1 RR
. Ca. Methanoplasma termitum MpT1.......................   0 HKs, 1 RR
. Cuniculiplasma divulgatum.............................   0 hits
. Ferroplasma acidarmanus fer1..........................   0 hits
. Ferroplasma acidiphilum...............................   0 hits
. methanogenic archaeon ISO4-H5.........................   3 HKs, 1 RR
. Picrophilus torridus DSM 9790.........................   0 hits
. Thermoplasma acidophilum DSM 1728.....................   0 hits
. Thermoplasma volcanium GSS1...........................   0 hits
. Thermoplasmatales archaeon BRNA1......................   0 HKs, 1 RR

unclassified Euryarchaeota

. uncultured marine group II euryarchaeote..............   0 HKs, 3 RRs

Nanoarchaeota

. Ca. Nanopusillus acidilobi ....   0 hits
. Nanoarchaeum equitans Kin4-M ....   0 hits

Thaumarchaeota

. Ca. Caldiarchaeum subterraneum........................   0 hits
. Ca. Nitrocosmicus oleophilus..........................   22 HKs, 24 RRs
. Ca. Nitrosoarchaeum limnia SFB1.......................   26 HKs, 33 RRs
. Ca. Nitrosomarinus catalina...........................   0 hits
. Ca. Nitrosopelagicus brevis...........................   0 hits
. Ca. Nitrosopumilus adriaticus.........................   16 HKs, 34 RRs
. Ca. Nitrosopumilus koreensis AR1......................   12 HKs, 15 RRs
. Ca. Nitrosopumilus piranensis.........................   2 HKs, 3 RRs
. Ca. Nitrosopumilus sediminis..........................   6 HKs, 10 RRs
. Ca. Nitrososphaera evergladensis SR1..................   19 HKs, 19 RRs
. Ca. Nitrososphaera gargensis Ga9.2....................   19 HKs, 31 RRs
. Ca. Nitrosotalea devanaterra ....   18 HKs, 19 RRs
. Ca. Nitrosotenuis cloacae strain SAT1.................   6 HKs, 15 RRs
. Cenarchaeum symbiosum.................................   0 hits
. Nitrosopumilus maritimus SCM1 ....   11 HKs, 10 RRs
. Nitrososphaera viennensis EN76........................   10 HKs, 11 RRs

Unclassified archaea

. archaeon GW2011 AR10..................................   0 hits
. archaeon GW2011 AR15..................................   0 hits
. archaeon GW2011 AR20..................................   0 hits
. uncultured archaeon clone ASS A1......................   0 hits

Hybrid histidine kinases, containing the REC domain at their C-termini, are counted as HKs; REC-HisK combinations with REC domains on their N-termini (with or wihout central PAS and/or GAF domains) are counted as RRs.

Proteins marked with * indicate inactivated or truncated REC domains, not included in the total count

---

Candidatus Micrarchaeota

Candidatus Micrarchaeota archaeon Mia14

HisK, no REC

Mia14_0098 ASI13441 GAF nad PAS sensor-containing signal trans... CDD UniProt Pfam InterPro

wHTH-REC

Mia14_0100 ASI13443 response regulator receiver [Candidatus Mi... CDD UniProt Pfam InterPro (wHTH-REC-bGlyc)

Euryarchaeota

Aciduliprofundum boonei T469

HisKin

Aboo_0120 ADD07932 response regulator receiver protein [Acidul... CDD UniProt Pfam InterPro
Aboo_0497 ADD08308 response regulator receiver protein [Acidul... CDD UniProt Pfam InterPro

REC only

Aboo_0851 ADD08660 response regulator receiver protein [Acidul... CDD UniProt Pfam InterPro
Aboo_1193 ADD09002 response regulator receiver protein [Acidul... CDD UniProt Pfam InterPro
Aboo_1536 ADD09342 response regulator receiver protein [Acidul... CDD UniProt Pfam InterPro

Other RRs

Aboo_1082 ADD08891 response regulator receiver protein [Acidul... CDD UniProt Pfam InterPro (REC-AcidOD1)

Aciduliprofundum sp. MAR08-339

HisKin

AciM339_0928 AGB04801 response regulator receiver protein [Aci... CDD UniProt Pfam InterPro
AciM339_1357 AGB05216 response regulator receiver protein [Aci... CDD UniProt Pfam InterPro

REC only

AciM339_0598 AGB04482 response regulator with CheY-like receiv... CDD UniProt Pfam InterPro
AciM339_1529 AGB05382 response regulator containing CheY-like ... CDD UniProt Pfam InterPro
AciM339_1569 AGB05418 response regulator with CheY-like receiv... CDD UniProt Pfam InterPro
*AciM339_1015 AGB04887 response regulator with CheY-like receiv... CDD UniProt Pfam InterPro

Other RRs

AciM339_1390 AGB05246 response regulator with CheY-like receiv... CDD UniProt Pfam InterPro (REC-AcidOD1)

Archaeoglobus fulgidus DSM 4304

HisK, no REC

AF_0021	AAB91205	signal-transducing histidine kinase [Archa...	CDD	UniProt	Pfam	InterPro
AF_0208	AAB91025	signal-transducing histidine kinase [Archa...	CDD	UniProt	Pfam	InterPro
AF_0450	AAB90782	signal-transducing histidine kinase [Archa...	CDD	UniProt	Pfam	InterPro
AF_0770	AAB90464	signal-transducing histidine kinase [Archa...	CDD	UniProt	Pfam	InterPro
AF_0893	AAB90347	signal-transducing histidine kinase [Archa...	CDD	UniProt	Pfam	InterPro
AF_1040	AAB90200	chemotaxis histidine kinase (cheA) [Archae...	CDD	UniProt	Pfam	InterPro
AF_1184	AAB90059	signal-transducing histidine kinase [Archa...	CDD	UniProt	Pfam	InterPro
AF_1452	AAB89797	signal-transducing histidine kinase [Archa...	CDD	UniProt	Pfam	InterPro
AF_1515	AAB89730	signal-transducing histidine kinase [Archa...	CDD	UniProt	Pfam	InterPro
AF_1639	AAB89602	signal-transducing histidine kinase [Archa...	CDD	UniProt	Pfam	InterPro
AF_1721	AAB89530	signal-transducing histidine kinase [Archa...	CDD	UniProt	Pfam	InterPro
AF_2032	AAB89224	signal-transducing histidine kinase, putat...	CDD	UniProt	Pfam	InterPro
AF_2109	AAB89139	signal-transducing histidine kinase [Archa...	CDD	UniProt	Pfam	InterPro
*AF_0881	(not translated, marked as frameshifted)					

HisKA, no HATPase

AF_0277	AAB90955	signal-transducing histidine kinase, putat...	CDD	UniProt	Pfam	InterPro
AF_0410	AAB90824	signal-transducing histidine kinase, putat...	CDD	UniProt	Pfam	InterPro
AF_0448	AAB90784	signal-transducing histidine kinase, putat...	CDD	UniProt	Pfam	InterPro
AF_1035	AAB90205	chemotaxis histidine kinase, putative [Arc...	CDD	UniProt	Pfam	InterPro
AF_1046	AAB90196	predicted coding region AF_1046 [Archaeogl...	CDD	UniProt	Pfam	InterPro
AF_1766	AAB89485	amino-acid ABC transporter, periplasmic bi...	CDD	UniProt	Pfam	InterPro
AF_2420	AAB91242	signal-transducing histidine kinase, putat...	CDD	UniProt	Pfam	InterPro

REC only

AF_0449	AAB90783	response regulator [Archaeoglobus fulgidus...	CDD	UniProt	Pfam	InterPro
AF_1042	AAB90207	chemotaxis response regulator (cheY) [Arch...	CDD	UniProt	Pfam	InterPro
AF_1063	AAB90176	response regulator [Archaeoglobus fulgidus...	CDD	UniProt	Pfam	InterPro
AF_1256	AAB89986	response regulator [Archaeoglobus fulgidus...	CDD	UniProt	Pfam	InterPro
AF_1384	AAB89861	response regulator [Archaeoglobus fulgidus...	CDD	UniProt	Pfam	InterPro
AF_1473	AAB89775	response regulator [Archaeoglobus fulgidus...	CDD	UniProt	Pfam	InterPro
AF_1898	AAB89351	response regulator [Archaeoglobus fulgidus...	CDD	UniProt	Pfam	InterPro
AF_2249	AAB89010	response regulator [Archaeoglobus fulgidus...	CDD	UniProt	Pfam	InterPro
AF_2419	AAB91243	response regulator [Archaeoglobus fulgidus...	CDD	UniProt	Pfam	InterPro

REC-HisK

AF_1483	AAB89763	signal-transducing histidine kinase [Archa..	CDD	UniProt	Pfam	InterPro

REC-CheB

AF_1041	AAB90199	protein-glutamate methylesterase [Archaeog...	CDD	UniProt	Pfam	InterPro

Other RRs

AF_1472	AAB89776	signal-transducing histidine kinase [Archa...	CDD	UniProt	Pfam	InterPro	(REC-PAS-GAF-HisKA)

Archaeoglobus profundus DSM 5631

HisK, no REC

Arcpr_0538	ADB57604	PAS/PAC sensor signal transduction histidi...	CDD	UniProt	Pfam	InterPro
Arcpr_0624	ADB57689	PAS/PAC sensor signal transduction histidi...	CDD	UniProt	Pfam	InterPro
Arcpr_1374	ADB58423	CheA signal transduction histidine kinase ...	CDD	UniProt	Pfam	InterPro

REC only

Arcpr_0537	ADB57603	response regulator receiver protein [Archa...	CDD	UniProt	Pfam	InterPro
Arcpr_0540	ADB57606	response regulator receiver protein [Archa...	CDD	UniProt	Pfam	InterPro
Arcpr_0996	ADB58057	response regulator receiver protein [Archa...	CDD	UniProt	Pfam	InterPro
Arcpr_1376	ADB58425	response regulator receiver protein [Archa...	CDD	UniProt	Pfam	InterPro

REC-CheB

Arcpr_1375	ADB58424	response regulator receiver protein [Archa...	CDD	UniProt	Pfam	InterPro

Other RRs

Arcpr_0541	ADB57607	response regulator receiver modulated CheB...	CDD	UniProt	Pfam	InterPro	(REC-HisKA)
Arcpr_1453	ADB58501	putative PAS/PAC sensor protein [Archaeogl...	CDD	UniProt	Pfam	InterPro	(REC-HisKA)

Archaeoglobus sulfaticallidus PM70-1

HisK, no REC

Asulf_01122	AGK61122	PAS domain S-box [Archaeoglobus sulfatica...	CDD	UniProt	Pfam	InterPro
Asulf_01671	AGK61644	PAS domain S-box [Archaeoglobus sulfatica...	CDD	UniProt	Pfam	InterPro
Asulf_01711	AGK61682	PAS domain S-box [Archaeoglobus sulfatica...	CDD	UniProt	Pfam	InterPro
Asulf_01766	AGK61737	hypothetical protein Asulf_01766 [Archaeo...	CDD	UniProt	Pfam	InterPro

HisKA, no HATPase

Asulf_00181	AGK60216	PAS domain S-box [Archaeoglobus sulfatica...	CDD	UniProt	Pfam	InterPro
Asulf_01632	AGK61607	PAS domain S-box [Archaeoglobus sulfatica...	CDD	UniProt	Pfam	InterPro
Asulf_01672	AGK61645	PAS domain S-box [Archaeoglobus sulfatica...	CDD	UniProt	Pfam	InterPro
Asulf_01680	AGK61652	PAS domain S-box [Archaeoglobus sulfatica...	CDD	UniProt	Pfam	InterPro
Asulf_02212	AGK62165	PAS domain S-box [Archaeoglobus sulfatica...	CDD	UniProt	Pfam	InterPro

REC only

Asulf_01219	AGK61217	hypothetical protein Asulf_01219 [Archaeo...	CDD	UniProt	Pfam	InterPro
Asulf_01271	AGK61267	hypothetical protein Asulf_01271 [Archaeo...	CDD	UniProt	Pfam	InterPro
Asulf_01865	AGK61833	hypothetical protein Asulf_01865 [Archaeo...	CDD	UniProt	Pfam	InterPro
Asulf_02176	AGK62129	hypothetical protein Asulf_02176 [Archaeo...	CDD	UniProt	Pfam	InterPro
*Asulf_00749	AGK60762	hypothetical protein Asulf_00749 [Archaeo...	CDD	UniProt	Pfam	InterPro

Other RRs

Asulf_00750 AGK60763 PAS domain S-box [Archaeoglobus sulfatica... CDD UniProt Pfam InterPro (REC-PAS-PAS-PAS-GAF-HisKA)

Archaeoglobus veneficus SNP6

HisK, no REC

Arcve_0187 AEA46226 PAS/PAC sensor signal transduction histidi... CDD UniProt Pfam InterPro
Arcve_0189 AEA46228 PAS/PAC sensor signal transduction histidi... CDD UniProt Pfam InterPro
Arcve_0380 AEA46413 PAS/PAC sensor signal transduction histidi... CDD UniProt Pfam InterPro
Arcve_1463 AEA47466 CheA signal transduction histidine kinase ... CDD UniProt Pfam InterPro

HisKA, no HATPase

Arcve_0186 AEA46225 PAS sensor protein [Archaeoglobus veneficu... CDD UniProt Pfam InterPro
Arcve_0534 AEA46561 PAS sensor protein [Archaeoglobus veneficu... CDD UniProt Pfam InterPro

REC only

Arcve_0190 AEA46229 response regulator receiver protein [Archa... CDD UniProt Pfam InterPro
Arcve_1461 AEA47464 response regulator receiver protein [Archa... CDD UniProt Pfam InterPro

REC-CheB

Arcve_1462 AEA47465 response regulator receiver modulated CheB... CDD UniProt Pfam InterPro

---

Candidatus Methanomassiliicoccus intestinalis Issoire-Mx1

Other RRs

*MMINT_05120 AGN25892 PAS domain S-box [Candidatus Methanomassi... CDD UniProt Pfam InterPro

---

Candidatus Methanomethylophilus alvus Mx1201

Other RRs

MMALV_04780 AGI85220 hypothetical protein MMALV_04780 [Candida... CDD UniProt Pfam InterPro (REC-xDUF835)

Candidatus Methanoplasma termitum

Other RRs

Mpt1_c03930 AIZ56287 regulator of RpoS [Candidatus Methanoplas... CDD UniProt Pfam InterPro (REC-DUF835)

Ferroglobus placidus DSM 10642

HisK, no REC

Ferp_0280 ADC64461 integral membrane sensor signal transducti... CDD UniProt Pfam InterPro
Ferp_1075 ADC65234 CheA signal transduction histidine kinase ... CDD UniProt Pfam InterPro
Ferp_1359 ADC65510 PAS/PAC sensor signal transduction histidi... CDD UniProt Pfam InterPro
Ferp_1726 ADC65870 PAS/PAC sensor signal transduction histidi... CDD UniProt Pfam InterPro

HisKA, no HATPase

Ferp_1360 ADC65511 putative PAS/PAC sensor protein [Ferroglob... CDD UniProt Pfam InterPro

REC only

Ferp_0251 ADC64433 response regulator receiver protein [Ferrog... CDD UniProt Pfam InterPro
Ferp_1077 ADC65236 response regulator receiver protein [Ferrog... CDD UniProt Pfam InterPro
Ferp_1725 ADC65869 response regulator receiver protein [Ferrog... CDD UniProt Pfam InterPro

REC-CheB

Ferp_1076 ADC65235 response regulator protein-glutamate methyl... CDD UniProt Pfam InterPro

---

Geoglobus acetivorans

HisK, no REC

GACE_0482 AIY89536 signal-transducing histidine kinase [Geogl... CDD UniProt Pfam InterPro
GACE_0668 AIY89720 Signal transduction histidine kinase CheA ... CDD UniProt Pfam InterPro
GACE_1270 AIY90311 signal-transducing histidine kinase [Geogl... CDD UniProt Pfam InterPro
GACE_1553 AIY90588 signal-transducing histidine kinase [Geogl... CDD UniProt Pfam InterPro
GACE_1579 AIY90614 signal-transducing histidine kinase [Geogl... CDD UniProt Pfam InterPro

HisKA, no HATPase

GACE_0662 AIY89714 hypothetical protein GACE_0662 [Geoglobus ... CDD UniProt Pfam InterPro
GACE_0824 AIY89873 signal-transducing histidine kinase, putat... CDD UniProt Pfam InterPro
GACE_0973 AIY90019 signal-transducing histidine kinase, putat... CDD UniProt Pfam InterPro
GACE_1241 AIY90280 signal-transducing histidine kinase, putat... CDD UniProt Pfam InterPro
GACE_1965 AIY90990 histidine kinase [Geoglobus acetivorans] ... CDD UniProt Pfam InterPro

REC only

GACE_0483 AIY89537 Signal transduction response regulator [Geo... CDD UniProt Pfam InterPro
GACE_0624 AIY89676 Signal transduction response regulator [Geo... CDD UniProt Pfam InterPro
GACE_0666 AIY89718 Chemotaxis regulator [Geoglobus acetivorans... CDD UniProt Pfam InterPro
GACE_0823 AIY89872 Signal transduction response regulator [Geo... CDD UniProt Pfam InterPro
GACE_0970 AIY90016 Signal transduction response regulator [Geo... CDD UniProt Pfam InterPro
GACE_1067 AIY90111 Chemotaxis regulator [Geoglobus acetivorans... CDD UniProt Pfam InterPro
GACE_1240 AIY90279 Signal transduction response regulator [Geo... CDD UniProt Pfam InterPro

REC-CheB

GACE_0667 AIY89719 Chemotaxis response regulator protein-gluta... CDD UniProt Pfam InterPro

---

Geoglobus ahangari

HisK, no REC

GAH_00133 AKG92508 Signal transduction histidine kinase [Geog... CDD UniProt Pfam InterPro

GAH_00453 AKG92195 Signal transduction histidine kinase [Geog... CDD UniProt Pfam InterPro
GAH_01483 AKG91226 Signal transduction histidine kinase [Geog... CDD UniProt Pfam InterPro
GAH_01739 AKG90980 PAS domain S-box [Geoglobus ahangari] ... CDD UniProt Pfam InterPro

HisKA, no HATPase

GAH_00707 AKG91958 ABC-type amino acid transport [Geoglobus a... CDD UniProt Pfam InterPro

REC only

GAH_01741 AKG90978 Response regulator receiver domain [Geoglog... CDD UniProt Pfam InterPro
GAH_01904 AKG90820 Response regulator receiver domain [Geoglog... CDD UniProt Pfam InterPro
GAH_01962 AKG90765 Response regulator receiver domain [Geoglog... CDD UniProt Pfam InterPro

---

Halalkalicoccus jeotgali B3

HisK, no REC

HacjB3_01570 ADJ13707 sensory transduction histidine kinase [H... CDD UniProt Pfam InterPro
HacjB3_03485 ADJ14088 HTR-like protein [Halalkalicoccus jeotga... CDD UniProt Pfam InterPro
HacjB3_07670 ADJ14919 histidine kinase [Halalkalicoccus jeotga... CDD UniProt Pfam InterPro
HacjB3_07685 ADJ14922 HTR-like protein [Halalkalicoccus jeotga... CDD UniProt Pfam InterPro
HacjB3_09455 ADJ15274 signal-transducing histidine kinase-like... CDD UniProt Pfam InterPro
HacjB3_12375 ADJ15856 integral membrane sensor signal transduc... CDD UniProt Pfam InterPro

REC only

HacjB3_03480 ADJ14087 multi-sensor signal transduction histidi... CDD UniProt Pfam InterPro
HacjB3_06575 ADJ14706 response regulator receiver protein [Hal... CDD UniProt Pfam InterPro
HacjB3_08175 ADJ15018 response regulator receiver protein [Hal... CDD UniProt Pfam InterPro
HacjB3_12380 ADJ15857 response regulator receiver protein [Hal... CDD UniProt Pfam InterPro

REC-HisK

HacjB3_01630 ADJ13719 signal-transducing histidine kinase-like... CDD UniProt Pfam InterPro
HacjB3_06595 ADJ14708 HTR-like protein [Halalkalicoccus jeotga... CDD UniProt Pfam InterPro

REC-PAS

HacjB3_05800 ADJ14551 HTR-like protein [Halalkalicoccus jeotga... CDD UniProt Pfam InterPro
HacjB3_08460 ADJ15075 multi-sensor signal transduction histidi... CDD UniProt Pfam InterPro

REC-HalX

HacjB3_06600 ADJ14709 response regulator receiver protein [Hal... CDD UniProt Pfam InterPro
HacjB3_10100 ADJ15403 response regulator receiver protein [Hal... CDD UniProt Pfam InterPro

HxlR-REC-HalX

HacjB3_01620 ADJ13717 HoxA-like transcriptional regulator [Hal... CDD UniProt Pfam InterPro (wHTH-REC-HalX)

Bat

HacjB3_00310 ADJ13455 putative PAS/PAC sensor protein [Halalka... CDD UniProt Pfam InterPro
HacjB3_04505 ADJ14292 putative PAS/PAC sensor protein [Halalka... CDD UniProt Pfam InterPro
HacjB3_18828 ADJ17105 bacterio-opsin activator-like protein (p... CDD UniProt Pfam InterPro

---

Halanaeroarchaeum sulfurireducens

HisK, no REC

HLASF_0096 AKH96610 signal-transducing histidine kinase [Halan... CDD UniProt Pfam InterPro
HLASF_0153 AKH96666 PAS/PAC sensor signal transduction histidi... CDD UniProt Pfam InterPro
HLASF_0190 AKH96701 histidine kinase [Halanaeroarchaeum sulfur... CDD UniProt Pfam InterPro
HLASF_0252 AKH96761 signal-transducing histidine kinase/respon... CDD UniProt Pfam InterPro
HLASF_0293 AKH96800 PAS domain s-box [Halanaeroarchaeum sulfur... CDD UniProt Pfam InterPro
HLASF_0431 AKH96937 putative signal-transducing histidine kina... CDD UniProt Pfam InterPro
HLASF_0463 AKH96968 multi-sensor signal transduction histidine... CDD UniProt Pfam InterPro
HLASF_0784 AKH97279 signal-transducing histidine kinase [Halan... CDD UniProt Pfam InterPro
HLASF_0844 AKH97337 PAS domain S-box [Halanaeroarchaeum sulfur... CDD UniProt Pfam InterPro
HLASF_0887 AKH97379 signal-transducing histidine kinase/respon... CDD UniProt Pfam InterPro
HLASF_0982 AKH97472 PAS/PAC sensor signal transduction histidi... CDD UniProt Pfam InterPro
HLASF_0986 AKH97476 signal-transducing histidine kinase [Halan... CDD UniProt Pfam InterPro
HLASF_1036 AKH97525 multi-sensor signal transduction histidine... CDD UniProt Pfam InterPro
HLASF_1137 AKH97625 PAS-PAC-PAC sensing his kinase [Halanaeroa... CDD UniProt Pfam InterPro
HLASF_1151 AKH97639 histidine kinase [Halanaeroarchaeum sulfur... CDD UniProt Pfam InterPro
HLASF_1363 AKH97849 chemotaxis protein CheA [Halanaeroarchaeum... CDD UniProt Pfam InterPro
HLASF_1594 AKH98072 PAS/PAC sensor signal transduction histidi... CDD UniProt Pfam InterPro
HLASF_1667 AKH98143 PAS/PAC sensor signal transduction histidi... CDD UniProt Pfam InterPro
HLASF_1729 AKH98204 signal-transducing histidine kinase [Halan... CDD UniProt Pfam InterPro
HLASF_1768 AKH98240 signal-transducing histidine kinase [Halan... CDD UniProt Pfam InterPro
HLASF_1769 AKH98241 PAS/PAC sensor signal transduction histidi... CDD UniProt Pfam InterPro
HLASF_1890 AKH98361 signal-transducing histidine kinase [Halan... CDD UniProt Pfam InterPro
HLASF_1899 AKH98370 PAS/PAC sensor signal transduction histidi... CDD UniProt Pfam InterPro
HLASF_1937 AKH98407 GAF sensor signal transduction histidine k... CDD UniProt Pfam InterPro
HLASF_2065 AKH98527 histidine kinase [Halanaeroarchaeum sulfur... CDD UniProt Pfam InterPro
HLASF_3032 AKH98658 signal-transducing histidine kinase-like p... CDD UniProt Pfam InterPro
HLASF_3033 AKH98659 PAS/PAC sensor signal transduction histidi... CDD UniProt Pfam InterPro

REC only

HLASF_1365 AKH97851 chemotaxis protein CheY [Halanaeroarchaeum... CDD UniProt Pfam InterPro

REC-HisK

HLASF_0290 AKH96797 PAS/PAC sensor signal transduction histidi... CDD UniProt Pfam InterPro
HLASF_0294 AKH96801 multi-sensor signal transduction histidine... CDD UniProt Pfam InterPro
HLASF_3082 AKH98708 PAS/PAC sensor signal transduction histidi... CDD UniProt Pfam InterPro

REC-CheB

HLASF_1364 AKH97850 chemotaxis-specific methylesterase CheB [H... CDD UniProt Pfam InterPro

REC-PAS

HLASF_0292	AKH96799	PAS/PAC sensor signal transduction histidi...	CDD	UniProt	Pfam	InterPro
**REC-HalX**						
HLASF_0332	AKH96839	response regulator receiver protein [Halan...	CDD	UniProt	Pfam	InterPro
HLASF_1337	AKH97823	response regulator receiver protein [Halan...	CDD	UniProt	Pfam	InterPro

Haloarcula hispanica ATCC 33960

**HisK, no REC**						
HAH_0071	AEM55704	HTR-like protein [Haloarcula hispanica ATC...	CDD	UniProt	Pfam	InterPro
HAH_0206	AEM55839	signal-transducing histidine kinase [Haloa...	CDD	UniProt	Pfam	InterPro
HAH_0589	AEM56212	signal-transducing histidine kinase-like p...	CDD	UniProt	Pfam	InterPro
HAH_0606	AEM56229	signal-transducing histidine kinase [Haloa...	CDD	UniProt	Pfam	InterPro
HAH_0667	AEM56290	putative signal-transducing histidine kina...	CDD	UniProt	Pfam	InterPro
HAH_0703	AEM56326	putative signal-transducing histidine kina...	CDD	UniProt	Pfam	InterPro
HAH_0841	AEM56461	HTR-like protein [Haloarcula hispanica ATC...	CDD	UniProt	Pfam	InterPro
HAH_0880	AEM56498	signal-transducing histidine kinase-like p...	CDD	UniProt	Pfam	InterPro
HAH_0991	AEM56609	putative light-sensing histidine kinase [H...	CDD	UniProt	Pfam	InterPro
HAH_1163	AEM56778	membrane associated histidine kinase [Halo...	CDD	UniProt	Pfam	InterPro
HAH_1220	AEM56835	putative GAF-PAC signal-transducing histid...	CDD	UniProt	Pfam	InterPro
HAH_1223	AEM56838	sensory transduction histidine kinase [Hal...	CDD	UniProt	Pfam	InterPro
HAH_1592	AEM57199	putative signal-transducing histidine kina...	CDD	UniProt	Pfam	InterPro
HAH_1615	AEM57222	HTR-like protein [Haloarcula hispanica ATC...	CDD	UniProt	Pfam	InterPro
HAH_1618	AEM57225	HTR-like protein [Haloarcula hispanica ATC...	CDD	UniProt	Pfam	InterPro
HAH_1706	AEM57309	putative signal-transducing histidine kina...	CDD	UniProt	Pfam	InterPro
HAH_1721	AEM57323	HTR-like protein [Haloarcula hispanica ATC...	CDD	UniProt	Pfam	InterPro
HAH_1966	AEM57563	HTR-like protein [Haloarcula hispanica ATC...	CDD	UniProt	Pfam	InterPro
HAH_2073	AEM57667	PAS-PAC-PAC sensing histidine kinase [Halo...	CDD	UniProt	Pfam	InterPro
HAH_2228	AEM57817	membrane bound histidine kinase A [Haloarc...	CDD	UniProt	Pfam	InterPro
HAH_2681	AEM58266	chemotaxis protein CheA [Haloarcula hispan...	CDD	UniProt	Pfam	InterPro
HAH_2781	AEM58361	histidine kinase [Haloarcula hispanica ATC...	CDD	UniProt	Pfam	InterPro
HAH_2893	AEM58470	adaptive-response sensory-kinase [Haloarcu...	CDD	UniProt	Pfam	InterPro
HAH_2907	AEM58484	protein kinase-like protein [Haloarcula hi...	CDD	UniProt	Pfam	InterPro
HAH_3035	AEM58609	adaptive-response sensory-kinase [Haloarcu...	CDD	UniProt	Pfam	InterPro
HAH_5014	AEM59148	PAS domain S-box (plasmid) [Haloarcula his...	CDD	UniProt	Pfam	InterPro
**REC only**						
HAH_1222	AEM56837	response regulator receiver [Haloarcula hi...	CDD	UniProt	Pfam	InterPro
HAH_2067	AEM57661	HTR-like protein [Haloarcula hispanica ATC...	CDD	UniProt	Pfam	InterPro
HAH_2670	AEM58255	chemotaxis protein CheY [Haloarcula hispan...	CDD	UniProt	Pfam	InterPro
**REC-HisK**						
HAH_0600	AEM56223	HTR-like protein [Haloarcula hispanica ATC...	CDD	UniProt	Pfam	InterPro
HAH_0612	AEM56235	HTR-like protein [Haloarcula hispanica ATC...	CDD	UniProt	Pfam	InterPro
HAH_0833	AEM56453	HTR-like protein [Haloarcula hispanica ATC...	CDD	UniProt	Pfam	InterPro
HAH_1041	AEM56659	signal transducing histidine kinase [Haloa...	CDD	UniProt	Pfam	InterPro
HAH_1151	AEM56766	HTR-like protein [Haloarcula hispanica ATC...	CDD	UniProt	Pfam	InterPro
HAH_1165	AEM56780	signal transducing histidine kinase [Haloa...	CDD	UniProt	Pfam	InterPro
HAH_1221	AEM56836	signal-transducing histidine kinase [Haloa...	CDD	UniProt	Pfam	InterPro
HAH_1275	AEM56890	HTR-like protein [Haloarcula hispanica ATC...	CDD	UniProt	Pfam	InterPro
HAH_1439	AEM57049	HTR-like protein [Haloarcula hispanica ATC...	CDD	UniProt	Pfam	InterPro
HAH_1487	AEM57096	HTR-like protein [Haloarcula hispanica ATC...	CDD	UniProt	Pfam	InterPro
HAH_2068	AEM57662	putative light- and oxygen-sensing transcr...	CDD	UniProt	Pfam	InterPro
HAH_2167	AEM57757	HTR-like protein [Haloarcula hispanica ATC...	CDD	UniProt	Pfam	InterPro
HAH_2332	AEM57920	HTR-like protein [Haloarcula hispanica ATC...	CDD	UniProt	Pfam	InterPro
HAH_2810	AEM58390	HTR-like protein [Haloarcula hispanica ATC...	CDD	UniProt	Pfam	InterPro
HAH_2849	AEM58428	putative light and redox sensing histidine...	CDD	UniProt	Pfam	InterPro
HAH_2964	AEM58538	adaptive-response sensory-kinase [Haloarcu...	CDD	UniProt	Pfam	InterPro
HAH_5035	AEM59169	light and oxygen sensing histidine kinase ...	CDD	UniProt	Pfam	InterPro
HAH_5036	AEM59170	HTR-like protein (plasmid) [Haloarcula his...	CDD	UniProt	Pfam	InterPro
**REC-CheB**						
HAH_2680	AEM58265	chemotaxis response regulator protein-glut...	CDD	UniProt	Pfam	InterPro
**REC-PAS\GAF**						
HAH_0305	AEM55931	HTR-like protein [Haloarcula hispanica ATC...	CDD	UniProt	Pfam	InterPro
HAH_1327	AEM56941	HTR-like protein [Haloarcula hispanica ATC...	CDD	UniProt	Pfam	InterPro
HAH_1746	AEM57348	conserved hypothetical protein [Haloarcula...	CDD	UniProt	Pfam	InterPro
HAH_2599	AEM58185	HTR-like protein [Haloarcula hispanica ATC...	CDD	UniProt	Pfam	InterPro
HAH_3048	AEM58622	HTR-like protein [Haloarcula hispanica ATC...	CDD	UniProt	Pfam	InterPro
HAH_1089	AEM56707	HTR-like protein [Haloarcula hispanica ATC...	CDD	UniProt	Pfam	InterPro
**REC-HalX**						
HAH_0545	AEM56169	HoxA-like transcriptional regulator [Haloa...	CDD	UniProt	Pfam	InterPro
HAH_0617	AEM56240	signal receiver domain-containing protein ...	CDD	UniProt	Pfam	InterPro
HAH_1093	AEM56711	HoxA-like transcriptional regulator [Haloa...	CDD	UniProt	Pfam	InterPro
HAH_1107	AEM56724	HoxA-like transcriptional regulator [Haloa...	CDD	UniProt	Pfam	InterPro
HAH_1725	AEM57327	HoxA-like transcriptional regulator / resp...	CDD	UniProt	Pfam	InterPro
HAH_2172	AEM57761	HoxA-like transcriptional regulator [Haloa...	CDD	UniProt	Pfam	InterPro
**Bat**						
HAH_5032	AEM59166	bacterio-opsin activator-like protein (pla...	CDD	UniProt	Pfam	InterPro
HAH_5327	AEM59460	bacterio-opsin activator-like protein (pla...	CDD	UniProt	Pfam	InterPro

Haloarcula marismortui ATCC 43049

**HisK, no REC**						
rrnAC0086	AAV45164	HTR-like protein [Haloarcula marismortui A...	CDD	UniProt	Pfam	InterPro
rrnAC0247	AAV45304	putative light-sensing histidine kinase [H...	CDD	UniProt	Pfam	InterPro
rrnAC0413	AAV45448	gaf-pac signal-transducing histidine kinas...	CDD	UniProt	Pfam	InterPro
rrnAC0130	AAV45202	signal-transducing histidine kinase-like [...	CDD	UniProt	Pfam	InterPro
rrnAC0301	AAV45356	signal transducing his kinase [Haloarcula ...	CDD	UniProt	Pfam	InterPro
rrnAC0410	AAV45445	sensory transduction histidine kinase [Hal...	CDD	UniProt	Pfam	InterPro
rrnAC0477	AAV45502	membrane associated histidine kinase [Halo...	CDD	UniProt	Pfam	InterPro
rrnAC0789	AAV45776	putative PAS/PAC sensing his kinase [Haloa...	CDD	UniProt	Pfam	InterPro
rrnAC0964	AAV45935	signal-transducing histidine kinase-like [...	CDD	UniProt	Pfam	InterPro
rrnAC0988	AAV45956	HTR-like protein [Haloarcula marismortui A...	CDD	UniProt	Pfam	InterPro
rrnAC0991	AAV45959	HTR-like protein [Haloarcula marismortui A...	CDD	UniProt	Pfam	InterPro
rrnAC1096	AAV46056	signal-transducing histidine kinase-like [...	CDD	UniProt	Pfam	InterPro
rrnAC1114	AAV46071	HTR-like protein [Haloarcula marismortui A...	CDD	UniProt	Pfam	InterPro
rrnAC1346	AAV46277	signal-transducing histidine kinase-like [...	CDD	UniProt	Pfam	InterPro
rrnAC1391	AAV46320	HTR-like protein [Haloarcula marismortui A...	CDD	UniProt	Pfam	InterPro
rrnAC1486	AAV46411	signal-transducing histidine kinase-like [...	CDD	UniProt	Pfam	InterPro
rrnAC1501	AAV46423	pas-pac-pac sensing his kinase [Haloarcula...	CDD	UniProt	Pfam	InterPro
rrnAC1693	AAV46601	membrane bound his kinase A [Haloarcula ma...	CDD	UniProt	Pfam	InterPro
rrnAC2205	AAV47041	chemotaxis protein CheA [Haloarcula marism...	CDD	UniProt	Pfam	InterPro
rrnAC2219	AAV47052	PhoR protein-like [Haloarcula marismortui ...	CDD	UniProt	Pfam	InterPro
rrnAC2337	AAV47159	histidine kinase [Haloarcula marismortui A...	CDD	UniProt	Pfam	InterPro
rrnAC2461	AAV47268	adaptive-response sensory-kinase [Haloarcu...	CDD	UniProt	Pfam	InterPro
rrnAC2477	AAV47283	protein kinase-like [Haloarcula marismortu...	CDD	UniProt	Pfam	InterPro
rrnAC2616	AAV47403	adaptive-response sensory-kinase [Haloarcu...	CDD	UniProt	Pfam	InterPro
rrnAC2694	AAV47465	unknown [Haloarcula marismortui ATCC 43049...	CDD	UniProt	Pfam	InterPro
rrnAC2789	AAV47546	HTR-like protein [Haloarcula marismortui A...	CDD	UniProt	Pfam	InterPro
rrnAC2945	AAV47680	signal-transducing histidine kinase [Haloa...	CDD	UniProt	Pfam	InterPro
rrnAC2997	AAV47726	membrane bound his kinase A [Haloarcula ma...	CDD	UniProt	Pfam	InterPro
rrnAC3347	AAV48037	signal-transducing histidine kinase-like [...	CDD	UniProt	Pfam	InterPro
rrnAC3349	AAV48038	signal-transducing histidine kinase-like [...	CDD	UniProt	Pfam	InterPro
rrnAC3370	AAV48057	signal transducing histidine kinase [Haloa...	CDD	UniProt	Pfam	InterPro
rrnAC3482	AAV48156	putative signal-transducing histidine kina...	CDD	UniProt	Pfam	InterPro
rrnB0133	AAV48329	putative redox sensing protein [Haloarcula...	CDD	UniProt	Pfam	InterPro
rrnB0156	AAV48345	putative light and redox sensing histidine...	CDD	UniProt	Pfam	InterPro
rrnB0180	AAV48366	putative 22-domain light- and oxygen-sensi...	CDD	UniProt	Pfam	InterPro
rrnB0295	AAV48462	gaf only light sensing his kinase [Haloarc...	CDD	UniProt	Pfam	InterPro
rrnB0299	AAV48465	nineteen domain light and oxygen sensing h...	CDD	UniProt	Pfam	InterPro
*rrnB0178	AAV48365	unknown [Haloarcula marismortui ATCC 43049...	CDD	UniProt	Pfam	InterPro
**REC only**						
rrnAC0411	AAV45446	response regulator receiver [Haloarcula ma...	CDD	UniProt	Pfam	InterPro
rrnAC1494	AAV46417	HTR-like protein [Haloarcula marismortui A...	CDD	UniProt	Pfam	InterPro
rrnAC2168	AAV47011	unknown [Haloarcula marismortui ATCC 43049...	CDD	UniProt	Pfam	InterPro
rrnAC2194	AAV47032	chemotaxis protein CheY [Haloarcula marism...	CDD	UniProt	Pfam	InterPro
rrnB0297	AAV48464	two-component response regulator [Haloarcu...	CDD	UniProt	Pfam	InterPro
rrnB0302	AAV48467	response regulator [Haloarcula marismortui...	CDD	UniProt	Pfam	InterPro
pNG4019	AAV44395	HTR-type protein [Haloarcula marismortui A...	CDD	UniProt	Pfam	InterPro
**REC-HisK**						
pNG7155	AAV44863	HTR-type protein [Haloarcula marismortui A...	CDD	UniProt	Pfam	InterPro
pNG7156	AAV44864	11-domain light and oxygen sensing his kin...	CDD	UniProt	Pfam	InterPro
rrnAC0075	AAV45155	HTR-type protein [Haloarcula marismortui A...	CDD	UniProt	Pfam	InterPro
rrnAC0412	AAV45447	signal-transducing histidine kinase [Haloa...	CDD	UniProt	Pfam	InterPro
rrnAC0475	AAV45500	signal transducing his kinase [Haloarcula ...	CDD	UniProt	Pfam	InterPro
rrnAC0487	AAV45512	HTR-type protein [Haloarcula marismortui A...	CDD	UniProt	Pfam	InterPro
rrnAC0574	AAV45588	HTR-type protein [Haloarcula marismortui A...	CDD	UniProt	Pfam	InterPro
rrnAC0794	AAV45781	HTR-like protein [Haloarcula marismortui A...	CDD	UniProt	Pfam	InterPro
rrnAC0848	AAV45829	HTR-type protein [Haloarcula marismortui A...	CDD	UniProt	Pfam	InterPro
rrnAC1495	AAV46418	putative light- and oxygen-sensing transcr...	CDD	UniProt	Pfam	InterPro
rrnAC1626	AAV46539	HTR-type protein [Haloarcula marismortui A...	CDD	UniProt	Pfam	InterPro
rrnAC1806	AAV46695	HTR-type protein [Haloarcula marismortui A...	CDD	UniProt	Pfam	InterPro
rrnAC2044	AAV46901	HTR-type protein [Haloarcula marismortui A...	CDD	UniProt	Pfam	InterPro
rrnAC2372	AAV47188	HTR-type protein [Haloarcula marismortui A...	CDD	UniProt	Pfam	InterPro
rrnAC2416	AAV47229	putative light and redox sensing histidine...	CDD	UniProt	Pfam	InterPro
rrnAC2533	AAV47335	adaptive-response sensory-kinase [Haloarcu...	CDD	UniProt	Pfam	InterPro
rrnAC2692	AAV47464	oxygen and light sensing his kinase [Haloa...	CDD	UniProt	Pfam	InterPro
rrnAC3050	AAV47770	HTR-type protein [Haloarcula marismortui A...	CDD	UniProt	Pfam	InterPro
rrnAC3361	AAV48050	HTR-type protein [Haloarcula marismortui A...	CDD	UniProt	Pfam	InterPro
rrnAC3379	AAV48063	HTR-type protein [Haloarcula marismortui A...	CDD	UniProt	Pfam	InterPro
rrnB0296	AAV48463	signal-transducing histidine kinase-like [...	CDD	UniProt	Pfam	InterPro
**REC-CheB**						
rrnAC2204	AAV47040	protein-glutamate methylesterase [Haloarcu...	CDD	UniProt	Pfam	InterPro
**REC-PAS/GAF**						
rrnAC0339	AAV45387	HTR-type protein [Haloarcula marismortui A...	CDD	UniProt	Pfam	InterPro
rrnAC0356	AAV45402	HTR-type protein [Haloarcula marismortui A...	CDD	UniProt	Pfam	InterPro
rrnAC0674	AAV45676	HTR-type protein [Haloarcula marismortui A...	CDD	UniProt	Pfam	InterPro
rrnAC1142	AAV46094	unknown [Haloarcula marismortui ATCC 43049...	CDD	UniProt	Pfam	InterPro
rrnAC2109	AAV46960	HTR-type protein [Haloarcula marismortui A...	CDD	UniProt	Pfam	InterPro
rrnAC2211	AAV47045	HTR-type protein [Haloarcula marismortui A...	CDD	UniProt	Pfam	InterPro
rrnAC3271	AAV47967	small putative GAF containing sensor [Halo...	CDD	UniProt	Pfam	InterPro

REC-HalX

rrnAC0361 AAV45406 HoxA-type transcriptional regulator [Haloa... CDD UniProt Pfam InterPro
rrnAC0536 AAV45554 HoxA-type transcriptional regulator [Haloa... CDD UniProt Pfam InterPro
rrnAC1118 AAV46075 HoxA-type transcriptional regulator [Haloa... CDD UniProt Pfam InterPro
rrnAC1630 AAV46544 HoxA-type transcriptional regulator [Haloa... CDD UniProt Pfam InterPro
rrnAC3308 AAV47999 HoxA-type transcriptional regulator [Haloa... CDD UniProt Pfam InterPro
rrnAC3385 AAV48068 signal receiver domain protein [Haloarcula... CDD UniProt Pfam InterPro

Bat

pNG7159 AAV44867 bacterio-opsin activator-like protein (pla... CDD UniProt Pfam InterPro
pNG7223 AAV44926 bacterio-opsin activator-like protein (pla... CDD UniProt Pfam InterPro

HxlR-REC-HalX

rrnB0301 AAV48466 HoxA-type transcriptional regulator [Haloa... CDD UniProt Pfam InterPro (wHTH-REC-HalX)

Haloarcula sp. CBA1115

HisK, no REC

SG26_02335 AJF24630 chemotaxis protein CheY [Haloarcula sp. CB... CDD UniProt Pfam InterPro
SG26_02855 AJF24726 peptidylprolyl isomerase [Haloarcula sp. C... CDD UniProt Pfam InterPro
SG26_03720 AJF24880 histidine kinase [Haloarcula sp. CBA1115] ... CDD UniProt Pfam InterPro
SG26_05915 AJF25298 chemotaxis protein CheA [Haloarcula sp. CB... CDD UniProt Pfam InterPro
SG26_06410 AJF25385 histidine kinase [Haloarcula sp. CBA1115] ... CDD UniProt Pfam InterPro
SG26_06835 AJF25462 histidine kinase [Haloarcula sp. CBA1115] ... CDD UniProt Pfam InterPro
SG26_07545 AJF25594 histidine kinase [Haloarcula sp. CBA1115] ... CDD UniProt Pfam InterPro
SG26_08310 AJF25729 chemotaxis protein CheY [Haloarcula sp. CB... CDD UniProt Pfam InterPro
SG26_08955 AJF25848 histidine kinase [Haloarcula sp. CBA1115] ... CDD UniProt Pfam InterPro
SG26_10810 AJF26181 histidine kinase [Haloarcula sp. CBA1115] ... CDD UniProt Pfam InterPro
SG26_10895 AJF26197 signal transduction protein [Haloarcula sp... CDD UniProt Pfam InterPro
SG26_11375 AJF26288 histidine kinase [Haloarcula sp. CBA1115] ... CDD UniProt Pfam InterPro
SG26_12065 AJF26413 chemotaxis protein CheY [Haloarcula sp. CB... CDD UniProt Pfam InterPro
SG26_12245 AJF26445 histidine kinase [Haloarcula sp. CBA1115] ... CDD UniProt Pfam InterPro
SG26_12770 AJF26538 histidine kinase [Haloarcula sp. CBA1115] ... CDD UniProt Pfam InterPro
SG26_13610 AJF26696 histidine kinase [Haloarcula sp. CBA1115] ... CDD UniProt Pfam InterPro
SG26_13895 AJF26748 histidine kinase [Haloarcula sp. CBA1115] ... CDD UniProt Pfam InterPro
SG26_13910 AJF26750 histidine kinase [Haloarcula sp. CBA1115] ... CDD UniProt Pfam InterPro
SG26_16040 AJF27140 chemotaxis protein CheY [Haloarcula sp. CB... CDD UniProt Pfam InterPro
SG26_16050 AJF27142 chemotaxis protein CheY [Haloarcula sp. CB... CDD UniProt Pfam InterPro
SG26_16440 AJF27207 histidine kinase [Haloarcula sp. CBA1115] ... CDD UniProt Pfam InterPro
SG26_16520 AJF27221 chemotaxis protein CheY [Haloarcula sp. CB... CDD UniProt Pfam InterPro
SG26_06905 AJF27389 protein kinase [Haloarcula sp. CBA1115] ... CDD UniProt Pfam InterPro
SG26_11200 AJF27435 histidine kinase [Haloarcula sp. CBA1115] ... CDD UniProt Pfam InterPro
SG26_15945 AJF27479 histidine kinase [Haloarcula sp. CBA1115] ... CDD UniProt Pfam InterPro
SG26_17995 AJF27656 diguanylate cyclase (plasmid) [Haloarcula ... CDD UniProt Pfam InterPro

REC only

SG26_02825 AJF24721 chemotaxis protein CheY [Haloarcula sp. CB... CDD UniProt Pfam InterPro
SG26_05860 AJF25288 regulator [Haloarcula sp. CBA1115] .... CDD UniProt Pfam InterPro
SG26_13905 AJF27459 chemotaxis protein CheY [Haloarcula sp. CB... CDD UniProt Pfam InterPro

REC-HisK

SG26_02830 AJF24722 transcriptional regulator [Haloarcula sp. ... CDD UniProt Pfam InterPro
SG26_03360 AJF24813 hypothetical protein SG26_03360 [Haloarcul... CDD UniProt Pfam InterPro
SG26_03415 AJF24824 chemotaxis protein CheY [Haloarcula sp. CB... CDD UniProt Pfam InterPro
SG26_04210 AJF24974 chemotaxis protein CheY [Haloarcula sp. CB... CDD UniProt Pfam InterPro
SG26_06545 AJF25411 chemotaxis protein CheY [Haloarcula sp. CB... CDD UniProt Pfam InterPro
SG26_06625 AJF25424 histidine kinase [Haloarcula sp. CBA1115] ... CDD UniProt Pfam InterPro
SG26_07185 AJF25525 histidine kinase [Haloarcula sp. CBA1115] ... CDD UniProt Pfam InterPro
SG26_10865 AJF26191 chemotaxis protein CheY [Haloarcula sp. CB... CDD UniProt Pfam InterPro
SG26_10925 AJF26203 chemotaxis protein CheY [Haloarcula sp. CB... CDD UniProt Pfam InterPro
SG26_12025 AJF26406 chemotaxis protein CheY [Haloarcula sp. CB... CDD UniProt Pfam InterPro
SG26_13550 AJF26684 chemotaxis protein CheY [Haloarcula sp. CB... CDD UniProt Pfam InterPro
SG26_13620 AJF26698 signal transduction protein [Haloarcula sp... CDD UniProt Pfam InterPro
SG26_13900 AJF26749 histidine kinase [Haloarcula sp. CBA1115] ... CDD UniProt Pfam InterPro
SG26_15195 AJF26981 chemotaxis protein CheY [Haloarcula sp. CB... CDD UniProt Pfam InterPro
SG26_15430 AJF27026 chemotaxis protein CheY [Haloarcula sp. CB... CDD UniProt Pfam InterPro
SG26_13020 AJF27451 signal transduction protein [Haloarcula sp... CDD UniProt Pfam InterPro
SG26_14170 AJF27467 chemotaxis protein CheY [Haloarcula sp. CB... CDD UniProt Pfam InterPro
SG26_17900 AJF27638 histidine kinase (plasmid) [Haloarcula sp.... CDD UniProt Pfam InterPro
SG26_17895 AJF27814 chemotaxis protein CheY (plasmid) [Haloarc... CDD UniProt Pfam InterPro

REC-CheB

SG26_05910 AJF25297 chemotaxis protein [Haloarcula sp. CBA1115... CDD UniProt Pfam InterPro

REC-PAS\GAF

SG26_05495 AJF25221 chemotaxis protein CheY [Haloarcula sp. CB... CDD UniProt Pfam InterPro
SG26_07610 AJF25606 HTR-like protein [Haloarcula sp. CBA1115] ... CDD UniProt Pfam InterPro
SG26_14645 AJF26875 chemotaxis protein CheY [Haloarcula sp. CB... CDD UniProt Pfam InterPro
SG26_16640 AJF27244 hypothetical protein SG26_16640 [Haloarcul... CDD UniProt Pfam InterPro
SG26_13250 AJF26631 chemotaxis protein CheY [Haloarcula sp. CB... CDD UniProt Pfam InterPro

REC-HalX

SG26_03440 AJF24828 DNA-binding protein [Haloarcula sp. CBA111... CDD UniProt Pfam InterPro
SG26_10610 AJF26145 DNA-binding protein [Haloarcula sp. CBA111... CDD UniProt Pfam InterPro
SG26_10950 AJF26208 histidine kinase [Haloarcula sp. CBA1115] ... CDD UniProt Pfam InterPro
SG26_13270 AJF26633 HoxA transcriptional regulator [Haloarcula... CDD UniProt Pfam InterPro

SG26_13340 AJF26645 HoxA transcriptional regulator [Haloarcula... CDD UniProt Pfam InterPro
SG26_16540 AJF27225 DNA-binding protein [Haloarcula sp. CBA111... CDD UniProt Pfam InterPro
BAT
SG26_17910 AJF27640 bacterio-opsin activator (plasmid) [Haloar... CDD UniProt Pfam InterPro
SG26_18225 AJF27697 bacterio-opsin activator (plasmid) [Haloar... CDD UniProt Pfam InterPro

---

Halobacterium hubeiense
HisK, no REC
HHUB_1728 CQH51403 histidine kinase [Halobacterium hubeiense]... CDD UniProt Pfam InterPro
HHUB_1729 CQH51412 sensor box histidine kinase [Halobacterium... CDD UniProt Pfam InterPro
HHUB_1816 CQH52193 histidine kinase [Halobacterium hubeiense]... CDD UniProt Pfam InterPro
HHUB_1911 CQH52992 sensor box histidine kinase [Halobacterium... CDD UniProt Pfam InterPro
HHUB_2038 CQH54053 sensor box histidine kinase [Halobacterium... CDD UniProt Pfam InterPro
HHUB_2095 CQH54516 sensor box histidine kinase [Halobacterium... CDD UniProt Pfam InterPro
HHUB_2110 CQH54635 sensor box histidine kinase [Halobacterium... CDD UniProt Pfam InterPro
HHUB_2152 CQH54925 taxis sensor histidine kinase CheA [Haloba... CDD UniProt Pfam InterPro
HHUB_2376 CQH56406 sensor box histidine kinase [Halobacterium... CDD UniProt Pfam InterPro
HHUB_2520 CQH57351 sensor box histidine kinase [Halobacterium... CDD UniProt Pfam InterPro
HHUB_2546 CQH57478 sensor box histidine kinase [Halobacterium... CDD UniProt Pfam InterPro
HHUB_2629 CQH57920 histidine kinase [Halobacterium hubeiense]... CDD UniProt Pfam InterPro
HHUB_3249 CQH60575 sensor box histidine kinase [Halobacterium... CDD UniProt Pfam InterPro
HHUB_3290 CQH60769 histidine kinase [Halobacterium hubeiense]... CDD UniProt Pfam InterPro
HHUB_3476 CQH61531 sensor box histidine kinase [Halobacterium... CDD UniProt Pfam InterPro
HHUB_3576 CQH61886 MEDS domain histidine kinase [Halobacteriu... CDD UniProt Pfam InterPro
HHUB_4136 CQH63613 sensor box histidine kinase (plasmid) [Hal... CDD UniProt Pfam InterPro
HHUB_4197 CQH63826 MEDS domain histidine kinase (plasmid) [Ha... CDD UniProt Pfam InterPro
REC only
HHUB_2154 CQH54936 response regulator CheY [Halobacterium hub... CDD UniProt Pfam InterPro
HHUB_2521 CQH57358 receiver box response regulator [Halobacte... CDD UniProt Pfam InterPro
REC-HisK
HHUB_1789 CQH51931 receiver/sensor box histidine kinase [Halo... CDD UniProt Pfam InterPro
HHUB_1988 CQH53656 receiver/sensor box histidine kinase [Halo... CDD UniProt Pfam InterPro
HHUB_2058 CQH54205 receiver/sensor box histidine kinase [Halo... CDD UniProt Pfam InterPro
HHUB_2176 CQH55070 sensor box histidine kinase [Halobacterium... CDD UniProt Pfam InterPro
HHUB_2382 CQH56443 receiver/sensor box histidine kinase [Halo... CDD UniProt Pfam InterPro
HHUB_2407 CQH56616 receiver/sensor box histidine kinase [Halo... CDD UniProt Pfam InterPro
REC-CheB
HHUB_2153 CQH54930 protein-glutamate methylesterase CheB [Hal... CDD UniProt Pfam InterPro
REC-PAS\GAF
HHUB_2544 CQH57469 receiver/sensor box [Halobacterium hubeien... CDD UniProt Pfam InterPro
HHUB_1699 CQH51141 receiver box response regulator [Halobacte... CDD UniProt Pfam InterPro
REC-HalX
HHUB_1730 CQH51423 receiver box response regulator [Halobacte... CDD UniProt Pfam InterPro
HHUB_1910 CQH52985 receiver box response regulator [Halobacte... CDD UniProt Pfam InterPro
HHUB_1912 CQH53000 receiver box response regulator [Halobacte... CDD UniProt Pfam InterPro
HHUB_2096 CQH54524 receiver box response regulator [Halobacte... CDD UniProt Pfam InterPro
HHUB_2174 CQH55056 receiver box response regulator [Halobacte... CDD UniProt Pfam InterPro
HHUB_2545 CQH57473 receiver box response regulator [Halobacte... CDD UniProt Pfam InterPro
BAT
HHUB_2522 CQH57367 receiver/sensor/bat box HTH-10 family tran... CDD UniProt Pfam InterPro
HHUB_4212 CQH63873 receiver/sensor/bat box HTH-10 family tran... CDD UniProt Pfam InterPro

---

Halobacterium salinarum NRC-1
HisK, no REC
VNG_0716G AAG19197 sensory histidine protein kinase homolog [... CDD UniProt Pfam InterPro
VNG_0736G AAG19214 signal-transducing histidine kinase homolo... CDD UniProt Pfam InterPro
VNG_0916G AAG19348 adaptive-response sensory-kinase [Halobact... CDD UniProt Pfam InterPro
VNG_0971G AAG19393 chemotaxis protein [Halobacterium salinaru... CDD UniProt Pfam InterPro
VNG_1175G AAG19550 PhoR protein homolog [Halobacterium salina... CDD UniProt Pfam InterPro
VNG_1193C AAG19566 conserved hypothetical protein [Halobacter... CDD UniProt Pfam InterPro
VNG_1234C AAG19596 conserved hypothetical protein [Halobacter... CDD UniProt Pfam InterPro
VNG_1374G AAG19701 signal-transducing histidine kinase homolo... CDD UniProt Pfam InterPro
VNG_1375C AAG19702 conserved hypothetical protein [Halobacter... CDD UniProt Pfam InterPro
VNG_2037C AAG20198 conserved hypothetical protein [Halobacter... CDD UniProt Pfam InterPro
VNG_2180C AAG20315 conserved hypothetical protein [Halobacter... CDD UniProt Pfam InterPro
REC only
VNG_0974G AAG19395 **chemotaxis protein CheY [Halobacterium sal...** CDD UniProt Pfam InterPro
REC-HisK
VNG_2334C AAG20440 conserved hypothetical protein [Halobacter... CDD UniProt Pfam InterPro
VNG_6035G AAG20731 Htr-like protein (plasmid) [Halobacterium ... CDD UniProt Pfam InterPro
REC-CheB
VNG_0973G AAG19394 **chemotaxis protein CheB [Halobacterium sal...** CDD UniProt Pfam InterPro
REC-HalX
VNG_0735G AAG19213 HoxA-like transcriptional regulator [Halob... CDD UniProt Pfam InterPro
VNG_0917G AAG19349 transcriptional regulatory protein [Haloba... CDD UniProt Pfam InterPro
VNG_2036G AAG20197 HoxA-like transcriptional regulator [Halob... CDD UniProt Pfam InterPro
BAT

VNG_1464G AAG19769 **Bacterioopsin transcriptional activator Ba...** CDD UniProt Pfam InterPro

---

Halobacterium sp. DL1

HisK, no REC

HALDL1_01605 AHG02470 histidine kinase [Halobacterium sp. DL1]... CDD UniProt Pfam InterPro
HALDL1_07930 AHG03532 histidine kinase [Halobacterium sp. DL1]... CDD UniProt Pfam InterPro
HALDL1_08070 AHG03556 ATPase [Halobacterium sp. DL1] ... CDD UniProt Pfam InterPro
HALDL1_08930 AHG03710 chemotaxis protein CheY [Halobacterium s... CDD UniProt Pfam InterPro
HALDL1_09165 AHG03752 chemotaxis protein CheA [Halobacterium s... CDD UniProt Pfam InterPro
HALDL1_09575 AHG03823 histidine kinase [Halobacterium sp. DL1]... CDD UniProt Pfam InterPro
HALDL1_10925 AHG04054 histidine kinase [Halobacterium sp. DL1]... CDD UniProt Pfam InterPro
HALDL1_12055 AHG04244 ATPase [Halobacterium sp. DL1] ... CDD UniProt Pfam InterPro
HALDL1_12510 AHG04326 histidine kinase [Halobacterium sp. DL1]... CDD UniProt Pfam InterPro
HALDL1_13135 AHG04433 histidine kinase [Halobacterium sp. DL1]... CDD UniProt Pfam InterPro
HALDL1_13475 AHG04491 histidine kinase [Halobacterium sp. DL1]... CDD UniProt Pfam InterPro
HALDL1_13795 AHG04538 histidine kinase [Halobacterium sp. DL1]... CDD UniProt Pfam InterPro
HALDL1_14765 AHG04711 histidine kinase [Halobacterium sp. DL1]... CDD UniProt Pfam InterPro
HALDL1_14850 AHG04722 histidine kinase [Halobacterium sp. DL1]... CDD UniProt Pfam InterPro
HALDL1_15195 AHG04785 histidine kinase [Halobacterium sp. DL1]... CDD UniProt Pfam InterPro
HALDL1_16045 AHG04933 histidine kinase [Halobacterium sp. DL1]... CDD UniProt Pfam InterPro
HALDL1_16380 AHG05000 histidine kinase [Halobacterium sp. DL1]... CDD UniProt Pfam InterPro
HALDL1_14015 AHG05376 hypothetical protein HALDL1_14015 [Halob... CDD UniProt Pfam InterPro

REC only

HALDL1_09175 AHG03754 chemotaxis protein CheY [Halobacterium s... CDD UniProt Pfam InterPro
HALDL1_11895 AHG04219 chemotaxis protein CheY [Halobacterium s... CDD UniProt Pfam InterPro
HALDL1_15945 AHG04916 histidine kinase [Halobacterium sp. DL1]... CDD UniProt Pfam InterPro
HALDL1_15340 AHG04813 response regulator [Halobacterium sp. DL... CDD UniProt Pfam InterPro
HALDL1_16385 AHG05001 chemotaxis protein CheY [Halobacterium s... CDD UniProt Pfam InterPro

REC-HisK

HALDL1_02160 AHG05087 hypothetical protein HALDL1_02160 [Halob... CDD UniProt Pfam InterPro
HALDL1_05165 AHG05182 hypothetical protein HALDL1_05165 [Halob... CDD UniProt Pfam InterPro
HALDL1_13390 AHG05353 hypothetical protein HALDL1_13390 [Halob... CDD UniProt Pfam InterPro
HALDL1_15400 AHG05403 hypothetical protein HALDL1_15400 [Halob... CDD UniProt Pfam InterPro

REC-CheB

HALDL1_09170 AHG03753 chemotaxis protein [Halobacterium sp. DL... CDD UniProt Pfam InterPro

REC-PAS

HALDL1_08820 AHG05257 hypothetical protein HALDL1_08820 [Halob... CDD UniProt Pfam InterPro

REC-HalX

HALDL1_08065 AHG03555 DNA-binding protein [Halobacterium sp. D... CDD UniProt Pfam InterPro
HALDL1_08935 AHG03711 DNA-binding protein [Halobacterium sp. D... CDD UniProt Pfam InterPro
HALDL1_09570 AHG03822 DNA-binding protein [Halobacterium sp. D... CDD UniProt Pfam InterPro
HALDL1_13790 AHG04537 DNA-binding protein [Halobacterium sp. D... CDD UniProt Pfam InterPro

BAT

HALDL1_11890 AHG05321 hypothetical protein HALDL1_11890 [Halob... CDD UniProt Pfam InterPro

REC-HalOD1

HALDL1_15395 AHG04822 chemotaxis protein CheY [Halobacterium s... CDD UniProt Pfam InterPro (REC-HalOD1)

Halobiforma lacisalsi AJ5

HisK, no REC

C445_00475 EMA37876 signal transduction histidine kinase CheA ... CDD UniProt Pfam InterPro
C445_00500 EMA37881 signal-transducing histidine kinase [Halob... CDD UniProt Pfam InterPro
C445_00711 EMA37365 PAS/PAC sensor signal transduction histidi... CDD UniProt Pfam InterPro
C445_02616 EMA37097 HTR-like protein [Halobiforma lacisalsi AJ... CDD UniProt Pfam InterPro
C445_03238 EMA36412 signal-transducing histidine kinase [Halob... CDD UniProt Pfam InterPro
C445_03768 EMA35943 signal-transducing histidine kinase [Halob... CDD UniProt Pfam InterPro
C445_04353 EMA36058 PAS/PAC sensor signal transduction histidi... CDD UniProt Pfam InterPro
C445_04493 EMA36086 multi-sensor signal transduction histidine... CDD UniProt Pfam InterPro
C445_04793 EMA36146 chemotaxis protein CheA [Halobiforma lacis... CDD UniProt Pfam InterPro
C445_05838 EMA35216 11-domain light and oxygen sensing his kin... CDD UniProt Pfam InterPro
C445_05878 EMA35224 signal-transducing histidine kinase [Halob... CDD UniProt Pfam InterPro
C445_06568 EMA34890 GAF sensor signal transduction histidine k... CDD UniProt Pfam InterPro
C445_06900 EMA34630 multi-sensor signal transduction histidine... CDD UniProt Pfam InterPro
C445_08994 EMA33813 GAF sensor signal transduction histidine k... CDD UniProt Pfam InterPro
C445_10797 EMA32599 multi-sensor signal transduction histidine... CDD UniProt Pfam InterPro
C445_12901 EMA32213 multi-sensor signal transduction histidine... CDD UniProt Pfam InterPro
C445_13570 EMA31847 signal-transducing histidine kinase [Halob... CDD UniProt Pfam InterPro
C445_14287 EMA31658 multi-sensor signal transduction histidine... CDD UniProt Pfam InterPro
C445_14317 EMA31664 multi-sensor signal transduction histidine... CDD UniProt Pfam InterPro
C445_14874 EMA31200 PAS sensor protein [Halobiforma lacisalsi ... CDD UniProt Pfam InterPro
C445_16569 EMA30442 histidine kinase, partial [Halobiforma lac... CDD UniProt Pfam InterPro
C445_16579 EMA30177 PAS sensor protein [Halobiforma lacisalsi ... CDD UniProt Pfam InterPro
C445_18181 EMA28579 PAS/PAC sensor signal transduction histidi... CDD UniProt Pfam InterPro
C445_20027 EMA27973 PAS/PAC sensor signal transduction histidi... CDD UniProt Pfam InterPro
C445_20052 EMA27978 PAS/PAC sensor signal transduction histidi... CDD UniProt Pfam InterPro

REC only

C445_00470 EMA37875 response regulator receiver protein [Halob... CDD UniProt Pfam InterPro
C445_02181 EMA37012 response regulator receiver protein [Halob... CDD UniProt Pfam InterPro

C445_04498	EMA36087	response regulator receiver protein [Halob...	CDD	UniProt	Pfam	InterPro
C445_04818	EMA35526	response regulator receiver protein [Halob...	CDD	UniProt	Pfam	InterPro
C445_12896	EMA32212	response regulator receiver protein [Halob...	CDD	UniProt	Pfam	InterPro
C445_14202	EMA31641	response regulator receiver protein [Halob...	CDD	UniProt	Pfam	InterPro
C445_14322	EMA31665	response regulator receiver protein [Halob...	CDD	UniProt	Pfam	InterPro
C445_20037	EMA27975	response regulator receiver protein [Halob...	CDD	UniProt	Pfam	InterPro
**REC-HisK**						
C445_03228	EMA36410	multi-sensor signal transduction histidine...	CDD	UniProt	Pfam	InterPro
C445_04538	EMA36095	putative signal-transducing histidine kina...	CDD	UniProt	Pfam	InterPro
C445_09720	EMA33056	HTR-like protein [Halobiforma lacisalsi AJ...	CDD	UniProt	Pfam	InterPro
C445_12756	EMA32184	multi-sensor signal transduction histidine...	CDD	UniProt	Pfam	InterPro
C445_18196	EMA28582	signal-transducing histidine kinase [Halob...	CDD	UniProt	Pfam	InterPro
C445_20390	EMA27376	pas domain s-box [Halobiforma lacisalsi AJ...	CDD	UniProt	Pfam	InterPro
**REC-CheB**						
C445_00480	EMA37877	chemotaxis-specific methylesterase [Halobi...	CDD	UniProt	Pfam	InterPro
C445_04813	EMA35525	chemotaxis-specific methylesterase [Halobi...	CDD	UniProt	Pfam	InterPro
**REC-HalX**						
C445_04553	EMA36098	response regulator receiver protein [Halob...	CDD	UniProt	Pfam	InterPro
C445_14869	EMA31199	response regulator receiver protein [Halob...	CDD	UniProt	Pfam	InterPro
**BAT**						
C445_00941	EMA37411	PAS/PAC sensor protein [Halobiforma lacisa...	CDD	UniProt	Pfam	InterPro
C445_06400	EMA35105	response regulator receiver modulated GAF ...	CDD	UniProt	Pfam	InterPro
C445_12891	EMA32211	response regulator receiver modulated GAF ...	CDD	UniProt	Pfam	InterPro
C445_16011	EMA30827	PAS/PAC sensor protein [Halobiforma lacisa...	CDD	UniProt	Pfam	InterPro

---

Halodesulfurarchaeum formicicum

**HisK, no REC**						
HTSR_0110	AOW79319	PAS/PAC sensor signal transduction histidi...	CDD	UniProt	Pfam	InterPro
HTSR_0391	AOW79591	signal-transducing histidine kinase [Halod...	CDD	UniProt	Pfam	InterPro
HTSR_0452	AOW79652	PAS/PAC sensor signal transduction histidi...	CDD	UniProt	Pfam	InterPro
HTSR_0469	AOW79668	signal transduction histidine kinase [Halo...	CDD	UniProt	Pfam	InterPro
HTSR_0473	AOW79672	signal-transducing histidine kinase [Halod...	CDD	UniProt	Pfam	InterPro
HTSR_0485	AOW79682	multi-sensor signal transduction histidine...	CDD	UniProt	Pfam	InterPro
HTSR_0621	AOW79814	integral membrane sensor signal transducti...	CDD	UniProt	Pfam	InterPro
HTSR_0688	AOW79880	signal-transducing histidine kinase [Halod...	CDD	UniProt	Pfam	InterPro
HTSR_0987	AOW80171	signal transduction histidine kinase [Halo...	CDD	UniProt	Pfam	InterPro
HTSR_1224	AOW80402	PAS domain-containing sensor histidine kin...	CDD	UniProt	Pfam	InterPro
HTSR_1520	AOW80694	two-component sensor histidine kinase [Hal...	CDD	UniProt	Pfam	InterPro
HTSR_1655	AOW80825	PAS domain-containing sensor histidine kin...	CDD	UniProt	Pfam	InterPro
HTSR_1685	AOW80855	PAS sensor histidine kinase [Halodesulfura...	CDD	UniProt	Pfam	InterPro
HTSR_1904	AOW81068	histidine kinase [Halodesulfurarchaeum for...	CDD	UniProt	Pfam	InterPro
HTSR_2060	AOW81220	two-component sensor histidine kinase [Hal...	CDD	UniProt	Pfam	InterPro
**REC only**						
HTSR_1368	AOW80544	chemotaxis protein CheY [Halodesulfurarchae...	CDD	UniProt	Pfam	InterPro
**REC-HisK**						
HTSR_1116	AOW80296	multi-sensor signal transduction histidine ...	CDD	UniProt	Pfam	InterPro
HTSR_1800	AOW80966	multi-sensor signal transduction histidine ...	CDD	UniProt	Pfam	InterPro
**REC-CheB**						
HTSR_1369	AOW80545	chemotaxis-specific methylesterase CheB [Ha...	CDD	UniProt	Pfam	InterPro
**REC-HalX**						
HTSR_1559	AOW80732	response regulator receiver protein [Halode...	CDD	UniProt	Pfam	InterPro
**BAT**						
HTSR_0470	AOW79669	PAS sensor histidine kinase [Halodesulfurar...	CDD	UniProt	Pfam	InterPro

---

Haloferax gibbonsii ATCC 33959

**HisK, no REC**						
ABY42_00525	AKU06301	HTR-like protein [Haloferax gibbonsii] ...	CDD	UniProt	Pfam	InterPro
ABY42_00840	AKU06356	HTR-like protein [Haloferax gibbonsii] ...	CDD	UniProt	Pfam	InterPro
ABY42_00970	AKU06382	histidine kinase [Haloferax gibbonsii] ...	CDD	UniProt	Pfam	InterPro
ABY42_02650	AKU06692	histidine kinase [Haloferax gibbonsii] ...	CDD	UniProt	Pfam	InterPro
ABY42_02935	AKU06745	histidine kinase [Haloferax gibbonsii] ...	CDD	UniProt	Pfam	InterPro
ABY42_03010	AKU06760	histidine kinase [Haloferax gibbonsii] ...	CDD	UniProt	Pfam	InterPro
ABY42_05880	AKU07293	histidine kinase [Haloferax gibbonsii] ...	CDD	UniProt	Pfam	InterPro
ABY42_05985	AKU07312	chemotaxis protein CheA [Haloferax gibbon...	CDD	UniProt	Pfam	InterPro
ABY42_06650	AKU07434	bacterio-opsin activator [Haloferax gibbo...	CDD	UniProt	Pfam	InterPro
ABY42_06845	AKU07471	histidine kinase [Haloferax gibbonsii] ...	CDD	UniProt	Pfam	InterPro
ABY42_06950	AKU07489	histidine kinase [Haloferax gibbonsii] ...	CDD	UniProt	Pfam	InterPro
ABY42_08095	AKU07712	histidine kinase [Haloferax gibbonsii] ...	CDD	UniProt	Pfam	InterPro
ABY42_08810	AKU07839	chemotaxis protein CheY [Haloferax gibbon...	CDD	UniProt	Pfam	InterPro
ABY42_08880	AKU07851	histidine kinase [Haloferax gibbonsii] ...	CDD	UniProt	Pfam	InterPro
ABY42_09550	AKU07973	histidine kinase [Haloferax gibbonsii] ...	CDD	UniProt	Pfam	InterPro
ABY42_10615	AKU08166	histidine kinase [Haloferax gibbonsii] ...	CDD	UniProt	Pfam	InterPro
ABY42_11660	AKU08355	chemotaxis protein CheY [Haloferax gibbon...	CDD	UniProt	Pfam	InterPro
ABY42_12560	AKU08523	transcriptional regulator [Haloferax gibb...	CDD	UniProt	Pfam	InterPro
ABY42_14485	AKU08884	chemotaxis protein [Haloferax gibbonsii] ...	CDD	UniProt	Pfam	InterPro
ABY42_06335	AKU09049	histidine kinase [Haloferax gibbonsii] ...	CDD	UniProt	Pfam	InterPro
ABY42_16170	AKU09336	histidine kinase (plasmid) [Haloferax gib...	CDD	UniProt	Pfam	InterPro

ABY42_16475	AKU09389	hypothetical protein ABY42_16475 (plasmid...	CDD	UniProt	Pfam	InterPro	
ABY42_16585	AKU09408	histidine kinase (plasmid) [Haloferax gib...	CDD	UniProt	Pfam	InterPro	
ABY42_16950	AKU09468	histidine kinase (plasmid) [Haloferax gib...	CDD	UniProt	Pfam	InterPro	
ABY42_16975	AKU09473	HTR-like protein (plasmid) [Haloferax gib...	CDD	UniProt	Pfam	InterPro	
ABY42_18000	AKU09698	histidine kinase (plasmid) [Haloferax gib...	CDD	UniProt	Pfam	InterPro	
ABY42_18255	AKU09807	HTR-like protein (plasmid) [Haloferax gib...	CDD	UniProt	Pfam	InterPro	
**REC only**							
ABY42_05905	AKU07298	regulator [Haloferax gibbonsii]	CDD	UniProt	Pfam	InterPro	
ABY42_06660	AKU07436	response regulator [Haloferax gibbonsii] ...	CDD	UniProt	Pfam	InterPro	
ABY42_09885	AKU08035	histidine kinase [Haloferax gibbonsii]	CDD	UniProt	Pfam	InterPro	
**REC-HisK**							
ABY42_01350	AKU06453	histidine kinase [Haloferax gibbonsii]	CDD	UniProt	Pfam	InterPro	
ABY42_02495	AKU06663	histidine kinase [Haloferax gibbonsii]	CDD	UniProt	Pfam	InterPro	
ABY42_07090	AKU07514	chemotaxis protein CheY [Haloferax gibbon...	CDD	UniProt	Pfam	InterPro	
ABY42_08375	AKU07764	chemotaxis protein CheY [Haloferax gibbon...	CDD	UniProt	Pfam	InterPro	
ABY42_10725	AKU08181	HTR-like protein [Haloferax gibbonsii]......	CDD	UniProt	Pfam	InterPro	
ABY42_10995	AKU08230	histidine kinase [Haloferax gibbonsii] ...	CDD	UniProt	Pfam	InterPro	
ABY42_13320	AKU08667	histidine kinase [Haloferax gibbonsii]......	CDD	UniProt	Pfam	InterPro	
ABY42_16180	AKU09338	HTR-like protein (plasmid) [Haloferax gib...	CDD	UniProt	Pfam	InterPro	
ABY42_17520	AKU09606	PAS sensor protein (plasmid) [Haloferax g...	CDD	UniProt	Pfam	InterPro	
ABY42_17580	AKU09618	histidine kinase (plasmid) [Haloferax gib...	CDD	UniProt	Pfam	InterPro	
**REC-CheB**							
ABY42_05990	AKU09043	chemotaxis protein [Haloferax gibbonsii] ...	CDD	UniProt	Pfam	InterPro	
**REC-PAS\GAF**							
ABY42_10740	AKU08183	HTR-like protein [Haloferax gibbonsii]......	CDD	UniProt	Pfam	InterPro	
ABY42_11995	AKU08419	diguanylate cyclase [Haloferax gibbonsii]...	CDD	UniProt	Pfam	InterPro	
ABY42_17740	AKU09648	HTR-like protein (plasmid) [Haloferax gib...	CDD	UniProt	Pfam	InterPro	
**REC-HalX**							
ABY42_06210	AKU07352	DNA-binding protein [Haloferax gibbonsii]...	CDD	UniProt	Pfam	InterPro	
ABY42_14490	AKU08885	transcriptional regulator [Haloferax gibb...	CDD	UniProt	Pfam	InterPro	
**BAT**							
ABY42_06655	AKU07435	chemotaxis protein CheY [Haloferax gibbon...	CDD	UniProt	Pfam	InterPro	
ABY42_09520	AKU09080	bacterio-opsin activator [Haloferax gibbo...	CDD	UniProt	Pfam	InterPro	
ABY42_16590	AKU09409	bacterio-opsin activator (plasmid) [Halof...	CDD	UniProt	Pfam	InterPro	
ABY42_16945	AKU09467	PAS/PAC sensor protein (plasmid) [Halofer...	CDD	UniProt	Pfam	InterPro	
**REC-HalOD1**							
ABY42_11475	AKU08318	chemotaxis protein CheY [Haloferax gibbon...	CDD	UniProt	Pfam	InterPro	(REC-HalOD1)

Haloferax mediterranei ATCC 33500

**HisK, no REC**						
HFX_0175	AFK17916	HTR-like protein [Haloferax mediterranei A...	CDD	UniProt	Pfam	InterPro
HFX_0206	AFK17947	PAS domain S-box [Haloferax mediterranei A...	CDD	UniProt	Pfam	InterPro
HFX_0587	AFK18312	putative signal-transducing histidine kina...	CDD	UniProt	Pfam	InterPro
HFX_1133	AFK18849	HTR-like protein [Haloferax mediterranei A...	CDD	UniProt	Pfam	InterPro
HFX_1211	AFK18924	PAS domain S-box [Haloferax mediterranei A...	CDD	UniProt	Pfam	InterPro
HFX_1232	AFK18945	taxis sensor histidine kinase cheA / two-c...	CDD	UniProt	Pfam	InterPro
HFX_1346	AFK19057	putative redox sensing protein [Haloferax ...	CDD	UniProt	Pfam	InterPro
HFX_1436	AFK19145	PAS domain S-box [Haloferax mediterranei A...	CDD	UniProt	Pfam	InterPro
HFX_1472	AFK19180	putative signal-transducing histidine kina...	CDD	UniProt	Pfam	InterPro
HFX_1751	AFK19457	PAS domain S-box [Haloferax mediterranei A...	CDD	UniProt	Pfam	InterPro
HFX_1895	AFK19592	HTR-like protein [Haloferax mediterranei A...	CDD	UniProt	Pfam	InterPro
HFX_2153	AFK19842	oxygen and light sensing his kinase [Halof...	CDD	UniProt	Pfam	InterPro
HFX_2348	AFK20035	HTR-like protein [Haloferax mediterranei A...	CDD	UniProt	Pfam	InterPro
HFX_2522	AFK20204	putative light- and oxygen-sensing transcr...	CDD	UniProt	Pfam	InterPro
HFX_5121	AFK20956	histidine kinase (plasmid) [Haloferax medi...	CDD	UniProt	Pfam	InterPro
HFX_5211	AFK21044	signal transduction histidine kinase / two...	CDD	UniProt	Pfam	InterPro
HFX_6029	AFK21156	PAS domain S-box (plasmid) [Haloferax medi...	CDD	UniProt	Pfam	InterPro
HFX_6124	AFK21249	multi-sensor signal transduction histidine...	CDD	UniProt	Pfam	InterPro
HFX_6198	AFK21321	light and oxygen sensing histidine kinase ...	CDD	UniProt	Pfam	InterPro
HFX_6272	AFK21395	signal-transducing histidine kinase-like p...	CDD	UniProt	Pfam	InterPro
HFX_0600	AFK18325	putative PAS/PAC sensing his kinase [Halof...	CDD	UniProt	Pfam	InterPro
HFX_1909	AFK19606	putative signal-transducing histidine kina...	CDD	UniProt	Pfam	InterPro
HFX_2053	AFK19745	histidine kinase [Haloferax mediterranei A...	CDD	UniProt	Pfam	InterPro
**REC only**						
HFX_1216	AFK18929	response regulator cheY [Haloferax mediter...	CDD	UniProt	Pfam	InterPro
HFX_1438	AFK19147	response regulator / transcription regulat...	CDD	UniProt	Pfam	InterPro
HFX_2988	AFK20652	response regulator receiver [Haloferax med...	CDD	UniProt	Pfam	InterPro
**REC-HisK**						
HFX_1367	AFK19077	HTR-like protein [Haloferax mediterranei A...	CDD	UniProt	Pfam	InterPro
HFX_1521	AFK19228	adaptive-response sensory-kinase [Halofera...	CDD	UniProt	Pfam	InterPro
HFX_1557	AFK19264	light and oxygen sensing histidine kinase ...	CDD	UniProt	Pfam	InterPro
HFX_1805	AFK19509	PAS domain S-box [Haloferax mediterranei A...	CDD	UniProt	Pfam	InterPro
**REC-CheB**						
HFX_1233	AFK18946	chemotaxis response regulator containing a...	CDD	UniProt	Pfam	InterPro
**REC-PAS\GAF**						
HFX_0350	AFK18086	HTR-like protein [Haloferax mediterranei A...	CDD	UniProt	Pfam	InterPro
HFX_2989	AFK20653	response regulator with CheY-like receiver...	CDD	UniProt	Pfam	InterPro
HFX_6428	AFK21547	response regulator with CheY-like receiver...	CDD	UniProt	Pfam	InterPro

REC-HalX
HFX_1279 AFK18992 HoxA-like transcriptional regulator [Halof... CDD UniProt Pfam InterPro
BAT
HFX_1437 AFK19146 bacterio-opsin activator [Haloferax medite... CDD UniProt Pfam InterPro
HFX_2047 AFK19739 bacterio-opsin activator-like protein [Hal... CDD UniProt Pfam InterPro
HFX_2176 AFK19865 bacterio-opsin activator-like protein [Hal... CDD UniProt Pfam InterPro
HFX_5179 AFK21013 putative DNA binding protein (plasmid) [Ha... CDD UniProt Pfam InterPro
REC-HalOD1
HFX_2316 AFK20003 HTR-like protein [Haloferax mediterranei A... CDD UniProt Pfam InterPro (REC-HalOD1)

Haloferax volcanii DS2

HisK, no REC
HVO_0110 ADE02962 sensor box histidine kinase [Haloferax vol... CDD UniProt Pfam InterPro
HVO_0632 ADE03168 sensor box histidine kinase [Haloferax vol... CDD UniProt Pfam InterPro
HVO_1201 ADE04627 sensor box histidine kinase [Haloferax vol... CDD UniProt Pfam InterPro
HVO_1223 ADE04296 taxis sensor histidine kinase CheA [Halofe... CDD UniProt Pfam InterPro
HVO_1298 ADE05096 sensor box histidine kinase [Haloferax vol... CDD UniProt Pfam InterPro
HVO_1356 ADE05126 sensor box histidine kinase [Haloferax vol... CDD UniProt Pfam InterPro
HVO_1397 ADE03313 histidine kinase [Haloferax volcanii DS2] ... CDD UniProt Pfam InterPro
HVO_1414 ADE03864 sensor box histidine kinase [Haloferax vol... CDD UniProt Pfam InterPro
HVO_1811 ADE03851 sensor box histidine kinase [Haloferax vol... CDD UniProt Pfam InterPro
HVO_2339 ADE02601 sensor box histidine kinase [Haloferax vol... CDD UniProt Pfam InterPro
HVO_2840 ADE04018 sensor box histidine kinase [Haloferax vol... CDD UniProt Pfam InterPro
HVO_A0160 ADE01815 sensor box histidine kinase (plasmid) [Hal... CDD UniProt Pfam InterPro
HVO_B0175 ADE01614 histidine kinase (plasmid) [Haloferax volc... CDD UniProt Pfam InterPro
HVO_B0196 ADE01294 histidine kinase (plasmid) [Haloferax volc... CDD UniProt Pfam InterPro
HVO_B0273 ADE01372 sensor box histidine kinase (plasmid) [Hal... CDD UniProt Pfam InterPro
REC only
HVO_1207 ADE02829 response regulator CheY [Haloferax volcani... CDD UniProt Pfam InterPro
HVO_1358 ADE03521 receiver box response regulator [Haloferax... CDD UniProt Pfam InterPro
HVO_2012 ADE02718 receiver box response regulator [Haloferax... CDD UniProt Pfam InterPro
HVO_B0153B AOP12795 receiver box response regulator (plasmid) ... CDD UniProt Pfam InterPro
REC-HisK
HVO_0301 ADE03966 receiver/sensor box histidine kinase [Halo... CDD UniProt Pfam InterPro
HVO_1456 ADE04984 receiver/sensor box histidine kinase [Halo... CDD UniProt Pfam InterPro
HVO_1713 ADE04135 receiver/sensor box histidine kinase [Halo... CDD UniProt Pfam InterPro
HVO_2030 ADE02405 HTR-like protein [Haloferax volcanii DS2] ... CDD UniProt Pfam InterPro
HVO_2660 ADE03121 receiver/sensor box histidine kinase [Halo... CDD UniProt Pfam InterPro
HVO_A0550 ADE02147 receiver/sensor box histidine kinase (plas... CDD UniProt Pfam InterPro
HVO_B0143 ADE01433 receiver/sensor box histidine kinase (plas... CDD UniProt Pfam InterPro
HVO_B0154 ADE01517 receiver/sensor box histidine kinase (plas... CDD UniProt Pfam InterPro
REC-CheB
HVO_1224 ADE03699 protein-glutamate methylesterase CheB [Hal... CDD UniProt Pfam InterPro
REC-PAS\GAF
HVO_1953 ADE04799 11-domain light and oxygen sensing his kin... CDD UniProt Pfam InterPro
HVO_2025 ADE03702 receiver/sensor box protein [Haloferax vol... CDD UniProt Pfam InterPro
HVO_A0540 ADE01917 homolog to receiver/sensor box histidine k... CDD UniProt Pfam InterPro
HVO_A0587 ADE01998 receiver/sensor box protein (plasmid) [Hal... CDD UniProt Pfam InterPro
REC-HalX
HVO_1271 ADE05178 receiver box response regulator [Haloferax... CDD UniProt Pfam InterPro
HVO_2838 ADE04759 receiver box response regulator [Haloferax... CDD UniProt Pfam InterPro
REC-HTH_10
HVO_B0272 ADE01630 receiver box HTH-10 family transcription r... CDD UniProt Pfam InterPro
BAT
HVO_1357 ADE03360 receiver/bat box HTH-10 family transcripti... CDD UniProt Pfam InterPro
REC-HalOD1
HVO_2306 ADE02288 receiver box response regulator [Haloferax... CDD UniProt Pfam InterPro (REC-HalOD1)

Halogeometricum borinquense DSM 11551

HisK, no REC
Hbor_00580 ADQ65671 PAS domain S-box [Halogeometricum borinque... CDD UniProt Pfam InterPro
Hbor_05520 ADQ66153 PAS domain S-box [Halogeometricum borinque... CDD UniProt Pfam InterPro
Hbor_06340 ADQ66234 PAS domain S-box [Halogeometricum borinque... CDD UniProt Pfam InterPro
Hbor_07060 ADQ66304 signal transduction histidine kinase [Halo... CDD UniProt Pfam InterPro
Hbor_08410 ADQ66437 signal transduction histidine kinase [Halo... CDD UniProt Pfam InterPro
Hbor_09050 ADQ66500 histidine kinase with PAS domain [Halogeom... CDD UniProt Pfam InterPro
Hbor_10620 ADQ66654 PAS domain S-box [Halogeometricum borinque... CDD UniProt Pfam InterPro
Hbor_11910 ADQ66780 PAS domain S-box [Halogeometricum borinque... CDD UniProt Pfam InterPro
Hbor_15540 ADQ67124 PAS domain S-box [Halogeometricum borinque... CDD UniProt Pfam InterPro
Hbor_17680 ADQ67336 PAS domain S-box [Halogeometricum borinque... CDD UniProt Pfam InterPro
Hbor_18740 ADQ67441 PAS domain S-box [Halogeometricum borinque... CDD UniProt Pfam InterPro
Hbor_20180 ADQ67584 chemotaxis protein histidine kinase-like p... CDD UniProt Pfam InterPro
Hbor_20420 ADQ67608 PAS domain S-box [Halogeometricum borinque... CDD UniProt Pfam InterPro
Hbor_23440 ADQ67904 PAS domain S-box [Halogeometricum borinque... CDD UniProt Pfam InterPro
Hbor_25510 ADQ68105 PAS domain S-box [Halogeometricum borinque... CDD UniProt Pfam InterPro
Hbor_26640 ADQ68216 histidine kinase [Halogeometricum borinque... CDD UniProt Pfam InterPro
Hbor_30190 ADQ68556 PAS domain S-box (plasmid) [Halogeometricu... CDD UniProt Pfam InterPro

Hbor_31860	ADQ68721	PAS domain S-box (plasmid) [Halogeometricu...	CDD	UniProt	Pfam	InterPro	
Hbor_32000	ADQ68735	PAS domain S-box (plasmid) [Halogeometricu...	CDD	UniProt	Pfam	InterPro	
Hbor_32650	ADQ68796	signal transduction histidine kinase (plas...	CDD	UniProt	Pfam	InterPro	
Hbor_33990	ADQ68921	PAS domain S-box (plasmid) [Halogeometricu...	CDD	UniProt	Pfam	InterPro	
**REC only**							
Hbor_18720	ADQ67439	response regulator with CheY-like receiver...	CDD	UniProt	Pfam	InterPro	
Hbor_20350	ADQ67601	response regulator containing CheY-like re...	CDD	UniProt	Pfam	InterPro	
Hbor_28770	ADQ68418	CheY-like receiver domain-containing prote...	CDD	UniProt	Pfam	InterPro	
Hbor_31840	ADQ68719	response regulator with CheY-like receiver...	CDD	UniProt	Pfam	InterPro	
**REC-HisK**							
Hbor_00810	ADQ65694	PAS domain S-box [Halogeometricum borinque...	CDD	UniProt	Pfam	InterPro	
Hbor_10850	ADQ66675	fused histidine kinase with GAF domain/res...	CDD	UniProt	Pfam	InterPro	
Hbor_14940	ADQ67068	PAS domain S-box [Halogeometricum borinque...	CDD	UniProt	Pfam	InterPro	
Hbor_26680	ADQ68220	signal transduction histidine kinase [Halo...	CDD	UniProt	Pfam	InterPro	
Hbor_31450	ADQ68680	PAS domain S-box (plasmid) [Halogeometricu...	CDD	UniProt	Pfam	InterPro	
Hbor_32190	ADQ68751	PAS domain S-box (plasmid) [Halogeometricu...	CDD	UniProt	Pfam	InterPro	
Hbor_34840	ADQ69004	PAS domain S-box (plasmid) [Halogeometricu...	CDD	UniProt	Pfam	InterPro	
Hbor_37050	ADQ69411	PAS domain S-box (plasmid) [Halogeometricu...	CDD	UniProt	Pfam	InterPro	
**REC-CheB**							
Hbor_20170	ADQ67583	chemotaxis response regulator containing a...	CDD	UniProt	Pfam	InterPro	
**REC-PAS\GAF**							
Hbor_28780	ADQ68419	response regulator with CheY-like receiver...	CDD	UniProt	Pfam	InterPro	
Hbor_32010	ADQ68736	PAS domain S-box (plasmid) [Halogeometricu...	CDD	UniProt	Pfam	InterPro	
Hbor_32660	ADQ68797	PAS domain S-box (plasmid) [Halogeometricu...	CDD	UniProt	Pfam	InterPro	
**REC-HalX**							
Hbor_14340	ADQ67014	response regulator with CheY-like receiver...	CDD	UniProt	Pfam	InterPro	
Hbor_19590	ADQ67525	response regulator with CheY-like receiver...	CDD	UniProt	Pfam	InterPro	
Hbor_31650	ADQ68700	response regulator with CheY-like receiver...	CDD	UniProt	Pfam	InterPro	
**HxlR-REC**							
Hbor_26690	ADQ68221	predicted transcriptional regulator [Halog...	CDD	UniProt	Pfam	InterPro	(wHTH-REC)
**BAT**							
Hbor_18730	ADQ67440	predicted DNA binding protein [Halogeometr...	CDD	UniProt	Pfam	InterPro	
Hbor_32330	ADQ68765	predicted DNA binding protein (plasmid) [H...	CDD	UniProt	Pfam	InterPro	
Hbor_09190	ADQ66514	PAS domain S-box [Halogeometricum borinque...	CDD	UniProt	Pfam	InterPro	
**REC-HalOD1**							
Hbor_07210	ADQ66319	response regulator with CheY-like receiver...	CDD	UniProt	Pfam	InterPro	(REC-HalOD1))
**Other RRs**							
Hbor_14290	ADQ67009	PAS domain S-box [Halogeometricum borinque...	CDD	UniProt	Pfam	InterPro	(REC-PAS-HisKA)

Halomicrobium mukohataei DSM 12286

**HisK, no REC**						
Hmuk_0028	ACV46179	histidine kinase [Halomicrobium mukohataei...	CDD	UniProt	Pfam	InterPro
Hmuk_0178	ACV46315	histidine kinase [Halomicrobium mukohataei...	CDD	UniProt	Pfam	InterPro
Hmuk_0249	ACV46386	CheA signal transduction histidine kinase ...	CDD	UniProt	Pfam	InterPro
Hmuk_0501	ACV46635	histidine kinase [Halomicrobium mukohataei...	CDD	UniProt	Pfam	InterPro
Hmuk_0502	ACV46636	PAS/PAC sensor signal transduction histidi...	CDD	UniProt	Pfam	InterPro
Hmuk_0626	ACV46758	PAS/PAC sensor signal transduction histidi...	CDD	UniProt	Pfam	InterPro
Hmuk_0715	ACV46846	PAS/PAC sensor signal transduction histidi...	CDD	UniProt	Pfam	InterPro
Hmuk_0851	ACV46982	PAS/PAC sensor signal transduction histidi...	CDD	UniProt	Pfam	InterPro
Hmuk_0999	ACV47127	histidine kinase [Halomicrobium mukohataei...	CDD	UniProt	Pfam	InterPro
Hmuk_1333	ACV47454	histidine kinase [Halomicrobium mukohataei...	CDD	UniProt	Pfam	InterPro
Hmuk_1512	ACV47627	PAS/PAC sensor signal transduction histidi...	CDD	UniProt	Pfam	InterPro
Hmuk_1570	ACV47684	PAS/PAC sensor signal transduction histidi...	CDD	UniProt	Pfam	InterPro
Hmuk_1643	ACV47757	GAF sensor signal transduction histidine k...	CDD	UniProt	Pfam	InterPro
Hmuk_2066	ACV48179	PAS/PAC sensor signal transduction histidi...	CDD	UniProt	Pfam	InterPro
Hmuk_2120	ACV48233	histidine kinase [Halomicrobium mukohataei...	CDD	UniProt	Pfam	InterPro
Hmuk_2229	ACV48342	PAS/PAC sensor signal transduction histidi...	CDD	UniProt	Pfam	InterPro
Hmuk_2243	ACV48356	histidine kinase [Halomicrobium mukohataei...	CDD	UniProt	Pfam	InterPro
Hmuk_2385	ACV48495	histidine kinase [Halomicrobium mukohataei...	CDD	UniProt	Pfam	InterPro
Hmuk_2491	ACV48599	multi-sensor signal transduction histidine...	CDD	UniProt	Pfam	InterPro
Hmuk_2916	ACV49021	multi-sensor signal transduction histidine...	CDD	UniProt	Pfam	InterPro
Hmuk_2976	ACV49081	PAS/PAC sensor signal transduction histidi...	CDD	UniProt	Pfam	InterPro
**REC only**						
Hmuk_0237	ACV46374	response regulator receiver protein [Halom...	CDD	UniProt	Pfam	InterPro
Hmuk_0731	ACV46862	response regulator receiver protein [Halom...	CDD	UniProt	Pfam	InterPro
Hmuk_1899	ACV48012	response regulator receiver protein [Halom...	CDD	UniProt	Pfam	InterPro
**REC-HisK**						
Hmuk_0184	ACV46321	histidine kinase [Halomicrobium mukohataei...	CDD	UniProt	Pfam	InterPro
Hmuk_0418	ACV46552	multi-sensor signal transduction histidine...	CDD	UniProt	Pfam	InterPro
Hmuk_1670	ACV47784	multi-sensor signal transduction histidine...	CDD	UniProt	Pfam	InterPro
Hmuk_1762	ACV47876	multi-sensor signal transduction histidine...	CDD	UniProt	Pfam	InterPro
Hmuk_1898	ACV48011	multi-sensor signal transduction histidine...	CDD	UniProt	Pfam	InterPro
Hmuk_2699	ACV48805	multi-sensor signal transduction histidine...	CDD	UniProt	Pfam	InterPro
Hmuk_2813	ACV48918	multi-sensor signal transduction histidine...	CDD	UniProt	Pfam	InterPro
Hmuk_3311	ACV49406	multi-sensor signal transduction histidine...	CDD	UniProt	Pfam	InterPro
**REC-CheB**						
Hmuk_0248	ACV46385	response regulator receiver modulated CheB...	CDD	UniProt	Pfam	InterPro
**REC-PAS**						

Hmuk_3154	ACV49259	putative PAS/PAC sensor protein [Halomicro...	CDD	UniProt	Pfam	InterPro	
**REC-HalX**							
Hmuk_0173	ACV46310	response regulator receiver protein [Halom...	CDD	UniProt	Pfam	InterPro	
Hmuk_1584	ACV47698	response regulator receiver protein [Halom...	CDD	UniProt	Pfam	InterPro	
Hmuk_1664	ACV47778	response regulator receiver protein [Halom...	CDD	UniProt	Pfam	InterPro	
Hmuk_1750	ACV47864	response regulator receiver protein [Halom...	CDD	UniProt	Pfam	InterPro	
Hmuk_1820	ACV47934	response regulator receiver protein [Halom...	CDD	UniProt	Pfam	InterPro	
Hmuk_1875	ACV47989	response regulator receiver protein [Halom...	CDD	UniProt	Pfam	InterPro	
Hmuk_2371	ACV48483	response regulator receiver protein [Halom...	CDD	UniProt	Pfam	InterPro	
Hmuk_2545	ACV48653	response regulator receiver protein [Halom...	CDD	UniProt	Pfam	InterPro	
Hmuk_2559	ACV48665	response regulator receiver protein [Halom...	CDD	UniProt	Pfam	InterPro	
**BAT**							
Hmuk_3382	ACV49467	putative PAS/PAC sensor protein (plasmid) ...	CDD	UniProt	Pfam	InterPro	
**REC-HalOD2**							
Hmuk_0286	ACV46423	response regulator receiver protein [Halom...	CDD	UniProt	Pfam	InterPro	(REC-HalOD2)
**Other RRs**							
Hmuk_0285	ACV46422	response regulator receiver protein [Halom...	CDD	UniProt	Pfam	InterPro	(REC-PAS-PAS-HisKA)

Halopiger xanaduensis SH-6

**HisK, no REC**						
Halxa_0710	AEH35349	multi-sensor signal transduction histidine...	CDD	UniProt	Pfam	InterPro
Halxa_0853	AEH35492	multi-sensor signal transduction histidine...	CDD	UniProt	Pfam	InterPro
Halxa_0871	AEH35509	GAF sensor signal transduction histidine k...	CDD	UniProt	Pfam	InterPro
Halxa_1160	AEH35793	GAF sensor signal transduction histidine k...	CDD	UniProt	Pfam	InterPro
Halxa_1205	AEH35838	PAS/PAC sensor signal transduction histidi...	CDD	UniProt	Pfam	InterPro
Halxa_1517	AEH36149	integral membrane sensor signal transducti...	CDD	UniProt	Pfam	InterPro
Halxa_1872	AEH36500	multi-sensor signal transduction histidine...	CDD	UniProt	Pfam	InterPro
Halxa_2070	AEH36695	PAS/PAC sensor signal transduction histidi...	CDD	UniProt	Pfam	InterPro
Halxa_2205	AEH36830	GAF sensor signal transduction histidine k...	CDD	UniProt	Pfam	InterPro
Halxa_2555	AEH37173	CheA signal transduction histidine kinase ...	CDD	UniProt	Pfam	InterPro
Halxa_2598	AEH37216	integral membrane sensor signal transducti...	CDD	UniProt	Pfam	InterPro
Halxa_2606	AEH37224	multi-sensor signal transduction histidine...	CDD	UniProt	Pfam	InterPro
Halxa_2975	AEH37591	multi-sensor signal transduction histidine...	CDD	UniProt	Pfam	InterPro
Halxa_3261	AEH37873	PAS sensor protein [Halopiger xanaduensis ...	CDD	UniProt	Pfam	InterPro
Halxa_3340	AEH37952	histidine kinase [Halopiger xanaduensis SH...	CDD	UniProt	Pfam	InterPro
Halxa_3341	AEH37953	PAS/PAC sensor signal transduction histidi...	CDD	UniProt	Pfam	InterPro
Halxa_3515	AEH38126	CheA signal transduction histidine kinase ...	CDD	UniProt	Pfam	InterPro
Halxa_3601	AEH38210	multi-sensor signal transduction histidine...	CDD	UniProt	Pfam	InterPro
Halxa_3718	AEH38325	multi-sensor signal transduction histidine...	CDD	UniProt	Pfam	InterPro
Halxa_0502	AEH39102	multi-sensor signal transduction histidine...	CDD	UniProt	Pfam	InterPro
Halxa_0520	AEH39119	PAS/PAC sensor signal transduction histidi...	CDD	UniProt	Pfam	InterPro
Halxa_0578	AEH39168	PAS/PAC sensor signal transduction histidi...	CDD	UniProt	Pfam	InterPro
Halxa_0654	AEH39233	PAS/PAC sensor signal transduction histidi...	CDD	UniProt	Pfam	InterPro
Halxa_0655	AEH39234	PAS/PAC sensor signal transduction histidi...	CDD	UniProt	Pfam	InterPro
Halxa_0677	AEH39250	PAS/PAC sensor signal transduction histidi...	CDD	UniProt	Pfam	InterPro
Halxa_0056	AEH39308	multi-sensor signal transduction histidine...	CDD	UniProt	Pfam	InterPro
**REC only**						
Halxa_0167	AEH39410	response regulator receiver (plasmid) [Hal...	CDD	UniProt	Pfam	InterPro
Halxa_2553	AEH37171	response regulator receiver protein [Halop...	CDD	UniProt	Pfam	InterPro
Halxa_2712	AEH37329	response regulator receiver protein [Halop...	CDD	UniProt	Pfam	InterPro
Halxa_2962	AEH37578	response regulator receiver protein [Halop...	CDD	UniProt	Pfam	InterPro
Halxa_2976	AEH37592	response regulator receiver [Halopiger xan...	CDD	UniProt	Pfam	InterPro
Halxa_3434	AEH38045	response regulator receiver protein [Halop...	CDD	UniProt	Pfam	InterPro
Halxa_3516	AEH38127	response regulator receiver protein [Halop...	CDD	UniProt	Pfam	InterPro
Halxa_3600	AEH38209	response regulator receiver protein [Halop...	CDD	UniProt	Pfam	InterPro
Halxa_4206	AEH38808	response regulator receiver protein [Halop...	CDD	UniProt	Pfam	InterPro
Halxa_4207	AEH38809	response regulator receiver protein [Halop...	CDD	UniProt	Pfam	InterPro
**REC-HisK**						
Halxa_1706	AEH36337	multi-sensor signal transduction histidine...	CDD	UniProt	Pfam	InterPro
Halxa_3980	AEH38585	multi-sensor signal transduction histidine...	CDD	UniProt	Pfam	InterPro
**REC-CheB**						
Halxa_2554	AEH37172	response regulator receiver modulated CheB...	CDD	UniProt	Pfam	InterPro
Halxa_3514	AEH38125	response regulator receiver modulated CheB...	CDD	UniProt	Pfam	InterPro
**REC-HalX**						
Halxa_2594	AEH37212	response regulator receiver protein [Halop...	CDD	UniProt	Pfam	InterPro
Halxa_3262	AEH37874	HalX domain protein [Halopiger xanaduensis...	CDD	UniProt	Pfam	InterPro
**BAT**						
Halxa_1652	AEH36284	response regulator receiver modulated GAF ...	CDD	UniProt	Pfam	InterPro
Halxa_3599	AEH38208	response regulator receiver modulated GAF ...	CDD	UniProt	Pfam	InterPro

---

Haloquadratum walsbyi DSM 16790

**HisK, no REC**						
HQ_1280A	CAJ51409	sensor box histidine kinase [Haloquadratum...	CDD	UniProt	Pfam	InterPro
HQ_1910A	CAJ52038	sensor box histidine kinase [Haloquadratum...	CDD	UniProt	Pfam	InterPro
HQ_2749A	CAJ52857	sensor box histidine kinase [Haloquadratum...	CDD	UniProt	Pfam	InterPro
HQ_2814A	CAJ52921	sensor box histidine kinase [Haloquadratum...	CDD	UniProt	Pfam	InterPro

REC only

HQ_2751A	CAJ52859	receiver box response regulator [Haloquadr...	CDD	UniProt	Pfam	InterPro	

REC-HisK

HQ_1073A	CAJ51203	receiver/sensor box histidine kinase [Halo...	CDD	UniProt	Pfam	InterPro	
HQ_2141A	CAJ52268	receiver/sensor box histidine kinase [Halo...	CDD	UniProt	Pfam	InterPro	
HQ_4020A	CAJ51110	receiver/sensor box histidine kinase [Halo...	CDD	UniProt	Pfam	InterPro	

HxlR-REC

HQ_1946A	CAJ52074	HxlR family transcription regulator / rece...	CDD	UniProt	Pfam	InterPro	(wHTH-REC)

BAT

HQ_2750A	CAJ52858	receiver/bat box HTH-10 family transcripti...	CDD	UniProt	Pfam	InterPro	

Halorhabdus tiamatea SARL4B

HisK, no REC

HTIA_0018	CCQ32170	PAS/PAC sensor signal transduction histidi...	CDD	UniProt	Pfam	InterPro
HTIA_0112	CCQ32263	signal transduction histidine kinase [Halo...	CDD	UniProt	Pfam	InterPro
HTIA_0322	CCQ32472	PAS/PAC sensor signal transduction histidi...	CDD	UniProt	Pfam	InterPro
HTIA_0434	CCQ32580	signal-transducing histidine kinase [Halor...	CDD	UniProt	Pfam	InterPro
HTIA_0795	CCQ32935	signal transduction histidine kinase CheA ...	CDD	UniProt	Pfam	InterPro
HTIA_0884	CCQ33024	PAS/PAC sensor signal transduction histidi...	CDD	UniProt	Pfam	InterPro
HTIA_1402	CCQ33530	histidine kinase [Halorhabdus tiamatea SAR...	CDD	UniProt	Pfam	InterPro
HTIA_1479	CCQ33606	signal-transducing histidine kinase [Halor...	CDD	UniProt	Pfam	InterPro
HTIA_1523	CCQ33650	histidine kinase [Halorhabdus tiamatea SAR...	CDD	UniProt	Pfam	InterPro
HTIA_1528	CCQ33655	integral membrane sensor signal transducti...	CDD	UniProt	Pfam	InterPro
HTIA_1575	CCQ33702	PAS/PAC sensor signal transduction histidi...	CDD	UniProt	Pfam	InterPro
HTIA_1926	CCQ34044	PAS/PAC sensor signal transduction histidi...	CDD	UniProt	Pfam	InterPro
HTIA_2045	CCQ34159	PAS/PAC sensor signal transduction histidi...	CDD	UniProt	Pfam	InterPro
HTIA_2055	CCQ34169	signal-transducing histidine kinase [Halor...	CDD	UniProt	Pfam	InterPro
HTIA_2153	CCQ34265	membrane bound his kinase A [Halorhabdus t...	CDD	UniProt	Pfam	InterPro
HTIA_2580	CCQ34686	signal-transducing histidine kinase-like p...	CDD	UniProt	Pfam	InterPro
HTIA_2756	CCQ34858	signal-transducing histidine kinase [Halor...	CDD	UniProt	Pfam	InterPro
HTIA_p2847	CCQ34949	histidine kinase (plasmid) [Halorhabdus ti...	CDD	UniProt	Pfam	InterPro

REC only

HTIA_0228	CCQ32379	signal transduction response regulator [Ha...	CDD	UniProt	Pfam	InterPro
HTIA_0805	CCQ32945	chemotaxis regulator-transmits chemorecept...	CDD	UniProt	Pfam	InterPro
HTIA_1483	CCQ33610	response regulator receiver protein [Halor...	CDD	UniProt	Pfam	InterPro
HTIA_2678	CCQ34783	signal transduction histidine kinase [Halo...	CDD	UniProt	Pfam	InterPro

REC-HisK

HTIA_0110	CCQ32261	multi-sensor signal transduction histidine...	CDD	UniProt	Pfam	InterPro
HTIA_0113	CCQ32264	PAS/PAC sensor signal transduction histidi...	CDD	UniProt	Pfam	InterPro
HTIA_0716	CCQ32856	multi-sensor signal transduction histidine...	CDD	UniProt	Pfam	InterPro
HTIA_1478	CCQ33605	11-domain light and oxygen sensing his kin...	CDD	UniProt	Pfam	InterPro
HTIA_1530	CCQ33657	multi-sensor signal transduction histidine...	CDD	UniProt	Pfam	InterPro
HTIA_1900	CCQ34019	multi-sensor signal transduction histidine...	CDD	UniProt	Pfam	InterPro
HTIA_2537	CCQ34643	multi-sensor signal transduction histidine...	CDD	UniProt	Pfam	InterPro

REC-CheB

HTIA_0796	CCQ32936	chemotaxis response regulator protein-glut...	CDD	UniProt	Pfam	InterPro

REC-HalX

HTIA_0537	CCQ32682	HoxA-like transcriptional regulator [Halor...	CDD	UniProt	Pfam	InterPro
HTIA_0664	CCQ32807	HoxA-like transcriptional regulator [Halor...	CDD	UniProt	Pfam	InterPro
HTIA_1808	CCQ33932	response regulator receiver protein [Halor...	CDD	UniProt	Pfam	InterPro
HTIA_2784	CCQ34886	HalX-type transcriptional regulator [Halor...	CDD	UniProt	Pfam	InterPro

BAT

HTIA_0508	CCQ32653	bacterio-opsin activator HTH domain protei...	CDD	UniProt	Pfam	InterPro

---

Halorhabdus utahensis DSM 12940

HisK, no REC

Huta_0059	ACV10248	PAS/PAC sensor signal transduction histidi...	CDD	UniProt	Pfam	InterPro
Huta_0175	ACV10363	histidine kinase [Halorhabdus utahensis DS...	CDD	UniProt	Pfam	InterPro
Huta_0199	ACV10387	PAS/PAC sensor signal transduction histidi...	CDD	UniProt	Pfam	InterPro
Huta_0253	ACV10441	histidine kinase [Halorhabdus utahensis DS...	CDD	UniProt	Pfam	InterPro
Huta_0313	ACV10500	PAS/PAC sensor signal transduction histidi...	CDD	UniProt	Pfam	InterPro
Huta_0421	ACV10608	PAS/PAC sensor signal transduction histidi...	CDD	UniProt	Pfam	InterPro
Huta_0929	ACV11112	histidine kinase [Halorhabdus utahensis DS...	CDD	UniProt	Pfam	InterPro
Huta_0942	ACV11125	CheA signal transduction histidine kinase ...	CDD	UniProt	Pfam	InterPro
Huta_0978	ACV11161	PAS/PAC sensor signal transduction histidi...	CDD	UniProt	Pfam	InterPro
Huta_1026	ACV11209	PAS/PAC sensor signal transduction histidi...	CDD	UniProt	Pfam	InterPro
Huta_1464	ACV11640	PAS/PAC sensor signal transduction histidi...	CDD	UniProt	Pfam	InterPro
Huta_1519	ACV11694	histidine kinase [Halorhabdus utahensis DS...	CDD	UniProt	Pfam	InterPro
Huta_1570	ACV11745	GAF sensor signal transduction histidine k...	CDD	UniProt	Pfam	InterPro
Huta_1591	ACV11766	PAS/PAC sensor signal transduction histidi...	CDD	UniProt	Pfam	InterPro
Huta_1646	ACV11820	histidine kinase [Halorhabdus utahensis DS...	CDD	UniProt	Pfam	InterPro
Huta_1743	ACV11915	histidine kinase [Halorhabdus utahensis DS...	CDD	UniProt	Pfam	InterPro
Huta_1749	ACV11921	histidine kinase [Halorhabdus utahensis DS...	CDD	UniProt	Pfam	InterPro
Huta_1771	ACV11943	PAS/PAC sensor signal transduction histidi...	CDD	UniProt	Pfam	InterPro
Huta_1802	ACV11974	signal transduction histidine kinase regul...	CDD	UniProt	Pfam	InterPro
Huta_1868	ACV12038	PAS/PAC sensor signal transduction histidi...	CDD	UniProt	Pfam	InterPro
Huta_2078	ACV12245	PAS/PAC sensor signal transduction histidi...	CDD	UniProt	Pfam	InterPro

Huta_2186	ACV12353	histidine kinase [Halorhabdus utahensis DS...	CDD	UniProt	Pfam	InterPro
Huta_2195	ACV12362	multi-sensor signal transduction histidine...	CDD	UniProt	Pfam	InterPro
Huta_2204	ACV12371	PAS/PAC sensor signal transduction histidi...	CDD	UniProt	Pfam	InterPro
Huta_2266	ACV12433	PAS/PAC sensor signal transduction histidi...	CDD	UniProt	Pfam	InterPro
Huta_2318	ACV12485	PAS/PAC sensor signal transduction histidi...	CDD	UniProt	Pfam	InterPro
Huta_2352	ACV12518	histidine kinase [Halorhabdus utahensis DS...	CDD	UniProt	Pfam	InterPro
Huta_2425	ACV12591	membrane bound his kinase A [Halorhabdus u...	CDD	UniProt	Pfam	InterPro
Huta_2850	ACV13011	histidine kinase [Halorhabdus utahensis DS...	CDD	UniProt	Pfam	InterPro
Huta_2881	ACV13042	PAS/PAC sensor signal transduction histidi...	CDD	UniProt	Pfam	InterPro
Huta_2923	ACV13084	histidine kinase [Halorhabdus utahensis DS...	CDD	UniProt	Pfam	InterPro
**REC only**						
Huta_0953	ACV11136	response regulator receiver protein [Halor...	CDD	UniProt	Pfam	InterPro
Huta_1567	ACV11742	response regulator receiver protein [Halor...	CDD	UniProt	Pfam	InterPro
Huta_1869	ACV12039	response regulator receiver protein [Halor...	CDD	UniProt	Pfam	InterPro
Huta_3023	ACV13184	response regulator receiver protein [Halor...	CDD	UniProt	Pfam	InterPro
**REC-HisK**						
Huta_0261	ACV10449	multi-sensor signal transduction histidine...	CDD	UniProt	Pfam	InterPro
Huta_0330	ACV10517	multi-sensor signal transduction histidine...	CDD	UniProt	Pfam	InterPro
Huta_0496	ACV10683	multi-sensor signal transduction histidine...	CDD	UniProt	Pfam	InterPro
Huta_0888	ACV11071	multi-sensor signal transduction histidine...	CDD	UniProt	Pfam	InterPro
Huta_1511	ACV11686	multi-sensor signal transduction histidine...	CDD	UniProt	Pfam	InterPro
Huta_1571	ACV11746	multi-sensor signal transduction histidine...	CDD	UniProt	Pfam	InterPro
Huta_1870	ACV12040	response regulator receiver sensor signal ...	CDD	UniProt	Pfam	InterPro
Huta_2351	ACV12517	multi-sensor signal transduction histidine...	CDD	UniProt	Pfam	InterPro
Huta_2409	ACV12575	multi-sensor signal transduction histidine...	CDD	UniProt	Pfam	InterPro
Huta_2488	ACV12652	multi-sensor signal transduction histidine...	CDD	UniProt	Pfam	InterPro
Huta_3020	ACV13181	multi-sensor signal transduction histidine...	CDD	UniProt	Pfam	InterPro
**REC-CheB**						
Huta_0943	ACV11126	response regulator receiver modulated CheB...	CDD	UniProt	Pfam	InterPro
**REC-PAS/GAF**						
Huta_0510	ACV10697	putative PAS/PAC sensor protein [Halorhabd...	CDD	UniProt	Pfam	InterPro
Huta_1892	ACV12061	putative PAS/PAC sensor protein [Halorhabd...	CDD	UniProt	Pfam	InterPro
Huta_2350	ACV12516	putative GAF sensor protein [Halorhabdus u...	CDD	UniProt	Pfam	InterPro
Huta_2566	ACV12730	response regulator receiver modulated GAF ...	CDD	UniProt	Pfam	InterPro
**REC-HalX**						
Huta_0359	ACV10546	response regulator receiver protein [Halor...	CDD	UniProt	Pfam	InterPro
Huta_0772	ACV10957	response regulator receiver protein [Halor...	CDD	UniProt	Pfam	InterPro
Huta_1740	ACV11912	response regulator receiver protein [Halor...	CDD	UniProt	Pfam	InterPro
Huta_1770	ACV11942	response regulator receiver protein [Halor...	CDD	UniProt	Pfam	InterPro
Huta_1773	ACV11945	response regulator receiver protein [Halor...	CDD	UniProt	Pfam	InterPro
Huta_1858	ACV12028	response regulator receiver protein [Halor...	CDD	UniProt	Pfam	InterPro
Huta_2885	ACV13046	response regulator receiver protein [Halor...	CDD	UniProt	Pfam	InterPro
**BAT**						
Huta_0317	ACV10504	putative PAS/PAC sensor protein [Halorhabd...	CDD	UniProt	Pfam	InterPro
Huta_2079	ACV12246	putative PAS/PAC sensor protein [Halorhabd...	CDD	UniProt	Pfam	InterPro

---

Halorientalis sp. IM1011

**HisK, no REC**						
BV210_01020	AQL41381	hypothetical protein BV210_01020 [Halorie...	CDD	UniProt	Pfam	InterPro
BV210_01525	AQL41472	histidine kinase [Halorientalis sp. IM101...	CDD	UniProt	Pfam	InterPro
BV210_02185	AQL41594	hypothetical protein BV210_02185 [Halorie...	CDD	UniProt	Pfam	InterPro
BV210_02225	AQL41602	hypothetical protein BV210_02225 [Halorie...	CDD	UniProt	Pfam	InterPro
BV210_03255	AQL41792	hypothetical protein BV210_03255 [Halorie...	CDD	UniProt	Pfam	InterPro
BV210_03425	AQL41822	histidine kinase [Halorientalis sp. IM101...	CDD	UniProt	Pfam	InterPro
BV210_03465	AQL41829	hypothetical protein BV210_03465 [Halorie...	CDD	UniProt	Pfam	InterPro
BV210_03490	AQL41834	hypothetical protein BV210_03490 [Halorie...	CDD	UniProt	Pfam	InterPro
BV210_04000	AQL41928	hypothetical protein BV210_04000 [Halorie...	CDD	UniProt	Pfam	InterPro
BV210_05560	AQL44485	diguanylate cyclase [Halorientalis sp. IM...	CDD	UniProt	Pfam	InterPro
BV210_05580	AQL42215	hypothetical protein BV210_05580 [Halorie...	CDD	UniProt	Pfam	InterPro
BV210_05725	AQL42242	hypothetical protein BV210_05725 [Halorie...	CDD	UniProt	Pfam	InterPro
BV210_07575	AQL42576	hypothetical protein BV210_07575 [Halorie...	CDD	UniProt	Pfam	InterPro
BV210_08000	AQL42656	hypothetical protein BV210_08000 [Halorie...	CDD	UniProt	Pfam	InterPro
BV210_08470	AQL42742	hypothetical protein BV210_08470 [Halorie...	CDD	UniProt	Pfam	InterPro
BV210_08685	AQL42782	hypothetical protein BV210_08685 [Halorie...	CDD	UniProt	Pfam	InterPro
BV210_08815	AQL42805	hypothetical protein BV210_08815 [Halorie...	CDD	UniProt	Pfam	InterPro
BV210_08875	AQL42817	hypothetical protein BV210_08875 [Halorie...	CDD	UniProt	Pfam	InterPro
BV210_09240	AQL42886	hypothetical protein BV210_09240 [Halorie...	CDD	UniProt	Pfam	InterPro
BV210_09745	AQL42979	hypothetical protein BV210_09745 [Halorie...	CDD	UniProt	Pfam	InterPro
BV210_10135	AQL43053	hypothetical protein BV210_10135 [Halorie...	CDD	UniProt	Pfam	InterPro
BV210_10340	AQL43087	chemotaxis protein CheA [Halorientalis sp...	CDD	UniProt	Pfam	InterPro
BV210_10560	AQL43129	hypothetical protein BV210_10560 [Halorie...	CDD	UniProt	Pfam	InterPro
BV210_10935	AQL43201	histidine kinase [Halorientalis sp. IM101...	CDD	UniProt	Pfam	InterPro
BV210_11115	AQL43234	hypothetical protein BV210_11115 [Halorie...	CDD	UniProt	Pfam	InterPro
BV210_12030	AQL43373	hypothetical protein BV210_12030 [Halorie...	CDD	UniProt	Pfam	InterPro
BV210_12210	AQL43408	hypothetical protein BV210_12210 [Halorie...	CDD	UniProt	Pfam	InterPro
BV210_12340	AQL43432	hypothetical protein BV210_12340 [Halorie...	CDD	UniProt	Pfam	InterPro
BV210_12765	AQL43513	hypothetical protein BV210_12765 [Halorie...	CDD	UniProt	Pfam	InterPro
BV210_13050	AQL43566	histidine kinase [Halorientalis sp. IM101...	CDD	UniProt	Pfam	InterPro

BV210_13185	AQL43591	hypothetical protein BV210_13185 [Halorie...	CDD	UniProt	Pfam	InterPro	
BV210_13315	AQL43616	hypothetical protein BV210_13315 [Halorie...	CDD	UniProt	Pfam	InterPro	
BV210_13825	AQL43716	hypothetical protein BV210_13825 [Halorie...	CDD	UniProt	Pfam	InterPro	
BV210_14240	AQL43791	hypothetical protein BV210_14240 [Halorie...	CDD	UniProt	Pfam	InterPro	
BV210_14565	AQL43852	hypothetical protein BV210_14565 [Halorie...	CDD	UniProt	Pfam	InterPro	
BV210_14660	AQL43870	hypothetical protein BV210_14660 [Halorie...	CDD	UniProt	Pfam	InterPro	
BV210_14670	AQL43872	hypothetical protein BV210_14670 [Halorie...	CDD	UniProt	Pfam	InterPro	
BV210_15625	AQL44584	hypothetical protein BV210_15625 [Halorie...	CDD	UniProt	Pfam	InterPro	
BV210_17915	AQL44642	hypothetical protein BV210_17915 (plasmid...	CDD	UniProt	Pfam	InterPro	
BV210_18265	AQL44701	hypothetical protein BV210_18265 (plasmid...	CDD	UniProt	Pfam	InterPro	
**REC only**							
BV210_10385	AQL43096	response regulator [Halorientalis sp. IM1...	CDD	UniProt	Pfam	InterPro	
BV210_17890	AQL44639	hypothetical protein BV210_17890 (plasmid...	CDD	UniProt	Pfam	InterPro	
BV210_01025	AQL41382	two-component system response regulator [...	CDD	UniProt	Pfam	InterPro	
**REC-HisK**							
BV210_00825	AQL41345	hybrid sensor histidine kinase/response r...	CDD	UniProt	Pfam	InterPro	
BV210_01030	AQL41383	hypothetical protein BV210_01030 [Halorie...	CDD	UniProt	Pfam	InterPro	
BV210_01800	AQL41523	hypothetical protein BV210_01800 [Halorie...	CDD	UniProt	Pfam	InterPro	
BV210_03535	AQL41843	hypothetical protein BV210_03535 [Halorie...	CDD	UniProt	Pfam	InterPro	
BV210_08710	AQL42785	hypothetical protein BV210_08710 [Halorie...	CDD	UniProt	Pfam	InterPro	
BV210_13280	AQL43610	hypothetical protein BV210_13280 [Halorie...	CDD	UniProt	Pfam	InterPro	
BV210_13800	AQL43711	hypothetical protein BV210_13800 [Halorie...	CDD	UniProt	Pfam	InterPro	
BV210_14335	AQL43809	hypothetical protein BV210_14335 [Halorie...	CDD	UniProt	Pfam	InterPro	
BV210_15870	AQL44090	hypothetical protein BV210_15870 [Halorie...	CDD	UniProt	Pfam	InterPro	
BV210_16005	AQL44117	hypothetical protein BV210_16005 [Halorie...	CDD	UniProt	Pfam	InterPro	
BV210_17885	AQL44638	histidine kinase (plasmid) [Halorientalis...	CDD	UniProt	Pfam	InterPro	
BV210_17970	AQL44653	hypothetical protein BV210_17970 (plasmid...	CDD	UniProt	Pfam	InterPro	
**REC-CheB**							
BV210_10345	AQL43088	chemotaxis response regulator protein-glu...	CDD	UniProt	Pfam	InterPro	
**REC-PAS**							
BV210_03535	AQL41843	hypothetical protein BV210_03535 [Halorie...	CDD	UniProt	Pfam	InterPro	
BV210_09375	AQL42912	hypothetical protein BV210_09375 [Halorie...	CDD	UniProt	Pfam	InterPro	
BV210_14340	AQL43810	hypothetical protein BV210_14340 [Halorie...	CDD	UniProt	Pfam	InterPro	
**REC-HalX**							
BV210_00205	AQL44418	HoxA transcriptional regulator [Halorient...	CDD	UniProt	Pfam	InterPro	
BV210_01670	AQL41501	hypothetical protein BV210_01670 [Halorie...	CDD	UniProt	Pfam	InterPro	
BV210_12200	AQL43406	DNA-binding protein [Halorientalis sp. IM...	CDD	UniProt	Pfam	InterPro	
BV210_12205	AQL43407	hypothetical protein BV210_12205 [Halorie...	CDD	UniProt	Pfam	InterPro	
BV210_12410	AQL43442	response regulator [Halorientalis sp. IM1...	CDD	UniProt	Pfam	InterPro	
BV210_13180	AQL43590	response regulator [Halorientalis sp. IM1...	CDD	UniProt	Pfam	InterPro	
BV210_14620	AQL43862	DNA-binding protein [Halorientalis sp. IM...	CDD	UniProt	Pfam	InterPro	
**BAT**							
BV210_00260	AQL41236	hypothetical protein BV210_00260 [Halorie...	CDD	UniProt	Pfam	InterPro	
BV210_01770	AQL41517	bacterio-opsin activator [Halorientalis s...	CDD	UniProt	Pfam	InterPro	
**HxlR-REC-HalX**							
BV210_17895	AQL44640	HoxA transcriptional regulator (plasmid) ...	CDD	UniProt	Pfam	InterPro	(wHTH-REC-HalX)
**REC-HalOD2**							
BV210_03540	AQL41844	hypothetical protein BV210_03540 [Halorie...	CDD	UniProt	Pfam	InterPro	(REC-HalOD2)

Halorubrum lacusprofundi ATCC 49239

**HisK, no REC**						
Hlac_0258	ACM55863	PAS/PAC sensor signal transduction histidi...	CDD	UniProt	Pfam	InterPro
Hlac_0318	ACM55922	putative PAS/PAC sensor protein [Halorubru...	CDD	UniProt	Pfam	InterPro
Hlac_0469	ACM56071	PAS/PAC sensor signal transduction histidi...	CDD	UniProt	Pfam	InterPro
Hlac_0718	ACM56320	PAS/PAC sensor signal transduction histidi...	CDD	UniProt	Pfam	InterPro
Hlac_0919	ACM56517	GAF sensor signal transduction histidine k...	CDD	UniProt	Pfam	InterPro
Hlac_0920	ACM56518	multi-sensor signal transduction histidine...	CDD	UniProt	Pfam	InterPro
Hlac_1000	ACM56597	histidine kinase [Halorubrum lacusprofundi...	CDD	UniProt	Pfam	InterPro
Hlac_1231	ACM56823	histidine kinase [Halorubrum lacusprofundi...	CDD	UniProt	Pfam	InterPro
Hlac_1440	ACM57028	PAS/PAC sensor signal transduction histidi...	CDD	UniProt	Pfam	InterPro
Hlac_1519	ACM57107	histidine kinase [Halorubrum lacusprofundi...	CDD	UniProt	Pfam	InterPro
Hlac_1704	ACM57288	histidine kinase [Halorubrum lacusprofundi...	CDD	UniProt	Pfam	InterPro
Hlac_1800	ACM57379	histidine kinase [Halorubrum lacusprofundi...	CDD	UniProt	Pfam	InterPro
Hlac_1809	ACM57388	histidine kinase [Halorubrum lacusprofundi...	CDD	UniProt	Pfam	InterPro
Hlac_1908	ACM57486	multi-sensor signal transduction histidine...	CDD	UniProt	Pfam	InterPro
Hlac_1946	ACM57524	PAS/PAC sensor signal transduction histidi...	CDD	UniProt	Pfam	InterPro
Hlac_2051	ACM57629	PAS/PAC sensor signal transduction histidi...	CDD	UniProt	Pfam	InterPro
Hlac_2126	ACM57703	PAS/PAC sensor signal transduction histidi...	CDD	UniProt	Pfam	InterPro
Hlac_2158	ACM57735	histidine kinase [Halorubrum lacusprofundi...	CDD	UniProt	Pfam	InterPro
Hlac_2263	ACM57839	CheA signal transduction histidine kinase ...	CDD	UniProt	Pfam	InterPro
Hlac_2389	ACM57964	histidine kinase [Halorubrum lacusprofundi...	CDD	UniProt	Pfam	InterPro
Hlac_2596	ACM58168	PAS/PAC sensor signal transduction histidi...	CDD	UniProt	Pfam	InterPro
Hlac_2652	ACM58223	signal transduction histidine kinase regul...	CDD	UniProt	Pfam	InterPro
Hlac_2684	ACM58255	PAS/PAC sensor signal transduction histidi...	CDD	UniProt	Pfam	InterPro
Hlac_2889	ACM58447	histidine kinase [Halorubrum lacusprofundi...	CDD	UniProt	Pfam	InterPro
Hlac_3302	ACM58826	histidine kinase (plasmid) [Halorubrum lac...	CDD	UniProt	Pfam	InterPro
Hlac_3597	ACM59104	PAS/PAC sensor signal transduction histidi...	CDD	UniProt	Pfam	InterPro
Hlac_3605	ACM59111	PAS/PAC sensor signal transduction histidi...	CDD	UniProt	Pfam	InterPro

Hlac_3606	ACM59112	histidine kinase (plasmid) [Halorubrum lac...	CDD	UniProt	Pfam	InterPro
Hlac_3654	ACM59156	PAS/PAC sensor signal transduction histidi...	CDD	UniProt	Pfam	InterPro
Hlac_3655	ACM59157	PAS/PAC sensor signal transduction histidi...	CDD	UniProt	Pfam	InterPro
Hlac_3657	ACM59159	PAS/PAC sensor signal transduction histidi...	CDD	UniProt	Pfam	InterPro
Hlac_3664	ACM59164	PAS/PAC sensor signal transduction histidi...	CDD	UniProt	Pfam	InterPro
**REC only**						
Hlac_0694	ACM56296	response regulator receiver protein [Halor...	CDD	UniProt	Pfam	InterPro
Hlac_2561	ACM58133	response regulator receiver protein [Halor...	CDD	UniProt	Pfam	InterPro
**REC-HisK**						
Hlac_1004	ACM56601	multi-sensor signal transduction histidine...	CDD	UniProt	Pfam	InterPro
Hlac_1349	ACM56941	multi-sensor signal transduction histidine...	CDD	UniProt	Pfam	InterPro
**REC-CheB**						
Hlac_2262	ACM57838	response regulator receiver modulated CheB...	CDD	UniProt	Pfam	InterPro
**REC-HalX**						
Hlac_1208	ACM56802	response regulator receiver protein [Halor...	CDD	UniProt	Pfam	InterPro
Hlac_2012	ACM57590	response regulator receiver protein [Halor...	CDD	UniProt	Pfam	InterPro
Hlac_2553	ACM58125	response regulator receiver protein [Halor...	CDD	UniProt	Pfam	InterPro
**BAT**						
Hlac_3274	ACM58800	response regulator receiver protein (plasm...	CDD	UniProt	Pfam	InterPro

---

Halostagnicola larsenii XH-48

**HisK, no REC**							
HALLA_00410	AHG01785	hypothetical protein HALLA_00410 (plasmid...	CDD	UniProt	Pfam	InterPro	
HALLA_01675	AHG02037	hypothetical protein HALLA_01675 (plasmid...	CDD	UniProt	Pfam	InterPro	
HALLA_07140	AHF98659	histidine kinase [Halostagnicola larsenii...	CDD	UniProt	Pfam	InterPro	
HALLA_08845	AHF98957	histidine kinase [Halostagnicola larsenii...	CDD	UniProt	Pfam	InterPro	
HALLA_10825	AHF99276	histidine kinase [Halostagnicola larsenii...	CDD	UniProt	Pfam	InterPro	
HALLA_11420	AHF99383	histidine kinase [Halostagnicola larsenii...	CDD	UniProt	Pfam	InterPro	
HALLA_13025	AHF99575	histidine kinase [Halostagnicola larsenii...	CDD	UniProt	Pfam	InterPro	
HALLA_13485	AHF99643	histidine kinase [Halostagnicola larsenii...	CDD	UniProt	Pfam	InterPro	
HALLA_14880	AHF99883	histidine kinase [Halostagnicola larsenii...	CDD	UniProt	Pfam	InterPro	
HALLA_16575	AHG00173	chemotaxis protein CheA [Halostagnicola l...	CDD	UniProt	Pfam	InterPro	
HALLA_19755	AHG00692	histidine kinase [Halostagnicola larsenii...	CDD	UniProt	Pfam	InterPro	
**REC only**							
HALLA_00975	AHG01898	chemotaxis protein CheY (plasmid) [Halost...	CDD	UniProt	Pfam	InterPro	
HALLA_07145	AHF98660	chemotaxis protein CheY [Halostagnicola l...	CDD	UniProt	Pfam	InterPro	
HALLA_09815	AHF99097	DNA-binding protein [Halostagnicola larse...	CDD	UniProt	Pfam	InterPro	
HALLA_13235	AHF99612	chemotaxis protein CheY [Halostagnicola l...	CDD	UniProt	Pfam	InterPro	
HALLA_16190	AHG00109	chemotaxis protein CheY [Halostagnicola l...	CDD	UniProt	Pfam	InterPro	
HALLA_18040	AHG00415	chemotaxis protein CheY [Halostagnicola l...	CDD	UniProt	Pfam	InterPro	
HALLA_20920	AHG02369	histidine kinase (plasmid) [Halostagnicol...	CDD	UniProt	Pfam	InterPro	
**REC-HisK**							
HALLA_15625	AHG01075	hypothetical protein HALLA_15625 [Halosta...	CDD	UniProt	Pfam	InterPro	
HALLA_20480	AHG02281	hypothetical protein HALLA_20480 (plasmid...	CDD	UniProt	Pfam	InterPro	
**REC-CheB**							
HALLA_16560	AHG00171	chemotaxis protein [Halostagnicola larsen...	CDD	UniProt	Pfam	InterPro	
**REC-HalX**							
HALLA_02410	AHG01250	hypothetical protein HALLA_02410 (plasmid...	CDD	UniProt	Pfam	InterPro	
**BAT**							
HALLA_00405	AHG01784	hypothetical protein HALLA_00405 (plasmid...	CDD	UniProt	Pfam	InterPro	
HALLA_07150	AHF98661	diguanylate cyclase [Halostagnicola larse...	CDD	UniProt	Pfam	InterPro	
HALLA_15955	AHG00073	bacterio-opsin activator [Halostagnicola ...	CDD	UniProt	Pfam	InterPro	
**Other RRs**							
HALLA_02415	AHG01251	hypothetical protein HALLA_02415 (plasmid...	CDD	UniProt	Pfam	InterPro	(REC-KaiC)

Haloterrigena daqingensis

**HisK, no REC**						
BB347_00640	APX95228	histidine kinase [Haloterrigena daqingens...	CDD	UniProt	Pfam	InterPro
BB347_01435	APX95380	histidine kinase [Haloterrigena daqingens...	CDD	UniProt	Pfam	InterPro
BB347_02130	APX95506	histidine kinase [Haloterrigena daqingens...	CDD	UniProt	Pfam	InterPro
BB347_02240	APX95526	histidine kinase [Haloterrigena daqingens...	CDD	UniProt	Pfam	InterPro
BB347_02910	APX95646	two-component sensor histidine kinase [Ha...	CDD	UniProt	Pfam	InterPro
BB347_03165	APX95695	histidine kinase [Haloterrigena daqingens...	CDD	UniProt	Pfam	InterPro
BB347_03520	APX95757	histidine kinase [Haloterrigena daqingens...	CDD	UniProt	Pfam	InterPro
BB347_03870	APX95824	histidine kinase [Haloterrigena daqingens...	CDD	UniProt	Pfam	InterPro
BB347_04065	APX95859	histidine kinase [Haloterrigena daqingens...	CDD	UniProt	Pfam	InterPro
BB347_09065	APX96754	histidine kinase [Haloterrigena daqingens...	CDD	UniProt	Pfam	InterPro
BB347_14305	APX97690	histidine kinase [Haloterrigena daqingens...	CDD	UniProt	Pfam	InterPro
BB347_16020	APX97995	two-component sensor histidine kinase [Ha...	CDD	UniProt	Pfam	InterPro
BB347_16315	APX98051	PAS domain-containing sensor histidine ki...	CDD	UniProt	Pfam	InterPro
BB347_06115	APX98154	PAS domain-containing sensor histidine ki...	CDD	UniProt	Pfam	InterPro
BB347_16855	APX98378	histidine kinase (plasmid) [Haloterrigena...	CDD	UniProt	Pfam	InterPro
**REC only**						
BB347_00645	APX95229	two-component system response regulator [...	CDD	UniProt	Pfam	InterPro
BB347_00805	APX95260	response regulator [Haloterrigena daqinge...	CDD	UniProt	Pfam	InterPro
BB347_01860	APX95458	response regulator [Haloterrigena daqinge...	CDD	UniProt	Pfam	InterPro
BB347_04595	APX95954	response regulator [Haloterrigena daqinge...	CDD	UniProt	Pfam	InterPro

BB347_05765	APX98152	response regulator [Haloterrigena daqinge...	CDD	UniProt	Pfam	InterPro
BB347_06110	APX96233	response regulator [Haloterrigena daqinge...	CDD	UniProt	Pfam	InterPro
BB347_16815	APX98370	response regulator (plasmid) [Haloterrige...	CDD	UniProt	Pfam	InterPro
**REC-HisK**						
BB347_00005	APX95111	hybrid sensor histidine kinase/response r...	CDD	UniProt	Pfam	InterPro
**REC-CheB**						
BB347_01855	APX95457	chemotaxis response regulator protein-glu...	CDD	UniProt	Pfam	InterPro
**REC-HalX**						
BB347_03875	APX95825	DNA-binding protein [Haloterrigena daqing...	CDD	UniProt	Pfam	InterPro
BB347_00960	APX95290	HoxA transcriptional regulator [Haloterri...	CDD	UniProt	Pfam	InterPro
**BAT**						
BB347_00650	APX95230	diguanylate cyclase [Haloterrigena daqing...	CDD	UniProt	Pfam	InterPro
BB347_09670	APX96866	bacterio-opsin activator [Haloterrigena d...	CDD	UniProt	Pfam	InterPro
BB347_12125	APX97299	diguanylate cyclase [Haloterrigena daqing...	CDD	UniProt	Pfam	InterPro
BB347_16770	APX98361	histidine kinase (plasmid) [Haloterrigena...	CDD	UniProt	Pfam	InterPro
BB347_17085	APX98421	hypothetical protein BB347_17085 (plasmid...	CDD	UniProt	Pfam	InterPro

Haloterrigena turkmenica DSM 5511

**HisK, no REC**						
Htur_0183	ADB59084	integral membrane sensor signal transducti...	CDD	UniProt	Pfam	InterPro
Htur_0209	ADB59110	multi-sensor signal transduction histidine...	CDD	UniProt	Pfam	InterPro
Htur_0255	ADB59156	multi-sensor signal transduction histidine...	CDD	UniProt	Pfam	InterPro
Htur_0270	ADB59171	PAS/PAC sensor signal transduction histidi...	CDD	UniProt	Pfam	InterPro
Htur_0375	ADB59275	integral membrane sensor signal transducti...	CDD	UniProt	Pfam	InterPro
Htur_0519	ADB59417	multi-sensor signal transduction histidine...	CDD	UniProt	Pfam	InterPro
Htur_0546	ADB59444	integral membrane sensor signal transducti...	CDD	UniProt	Pfam	InterPro
Htur_0906	ADB59801	multi-sensor signal transduction histidine...	CDD	UniProt	Pfam	InterPro
Htur_0958	ADB59852	CheA signal transduction histidine kinase ...	CDD	UniProt	Pfam	InterPro
Htur_0970	ADB59864	multi-sensor signal transduction histidine...	CDD	UniProt	Pfam	InterPro
Htur_1021	ADB59913	multi-sensor signal transduction histidine...	CDD	UniProt	Pfam	InterPro
Htur_1080	ADB59972	GAF sensor signal transduction histidine k...	CDD	UniProt	Pfam	InterPro
Htur_1083	ADB59975	GAF sensor signal transduction histidine k...	CDD	UniProt	Pfam	InterPro
Htur_1263	ADB60153	histidine kinase [Haloterrigena turkmenica...	CDD	UniProt	Pfam	InterPro
Htur_1265	ADB60155	integral membrane sensor signal transducti...	CDD	UniProt	Pfam	InterPro
Htur_1537	ADB60423	putative PAS/PAC sensor protein [Haloterri...	CDD	UniProt	Pfam	InterPro
Htur_1725	ADB60610	integral membrane sensor signal transducti...	CDD	UniProt	Pfam	InterPro
Htur_1881	ADB60766	multi-sensor signal transduction histidine...	CDD	UniProt	Pfam	InterPro
Htur_1974	ADB60858	multi-sensor signal transduction histidine...	CDD	UniProt	Pfam	InterPro
Htur_2049	ADB60933	histidine kinase [Haloterrigena turkmenica...	CDD	UniProt	Pfam	InterPro
Htur_2215	ADB61095	PAS/PAC sensor signal transduction histidi...	CDD	UniProt	Pfam	InterPro
Htur_2280	ADB61160	PAS/PAC sensor signal transduction histidi...	CDD	UniProt	Pfam	InterPro
Htur_2292	ADB61172	multi-sensor signal transduction histidine...	CDD	UniProt	Pfam	InterPro
Htur_2359	ADB61237	PAS/PAC sensor signal transduction histidi...	CDD	UniProt	Pfam	InterPro
Htur_2509	ADB61386	integral membrane sensor signal transducti...	CDD	UniProt	Pfam	InterPro
Htur_2519	ADB61396	multi-sensor signal transduction histidine...	CDD	UniProt	Pfam	InterPro
Htur_2594	ADB61471	multi-sensor signal transduction histidine...	CDD	UniProt	Pfam	InterPro
Htur_2774	ADB61647	multi-sensor signal transduction histidine...	CDD	UniProt	Pfam	InterPro
Htur_2820	ADB61691	histidine kinase [Haloterrigena turkmenica...	CDD	UniProt	Pfam	InterPro
Htur_2827	ADB61698	multi-sensor signal transduction histidine...	CDD	UniProt	Pfam	InterPro
Htur_2907	ADB61776	multi-sensor signal transduction histidine...	CDD	UniProt	Pfam	InterPro
Htur_2911	ADB61780	multi-sensor signal transduction histidine...	CDD	UniProt	Pfam	InterPro
Htur_2936	ADB61805	multi-sensor signal transduction histidine...	CDD	UniProt	Pfam	InterPro
Htur_2998	ADB61866	integral membrane sensor signal transducti...	CDD	UniProt	Pfam	InterPro
Htur_3059	ADB61925	multi-sensor signal transduction histidine...	CDD	UniProt	Pfam	InterPro
Htur_3150	ADB62015	integral membrane sensor signal transducti...	CDD	UniProt	Pfam	InterPro
Htur_3223	ADB62087	multi-sensor signal transduction histidine...	CDD	UniProt	Pfam	InterPro
Htur_3424	ADB62287	integral membrane sensor signal transducti...	CDD	UniProt	Pfam	InterPro
Htur_3436	ADB62299	histidine kinase [Haloterrigena turkmenica...	CDD	UniProt	Pfam	InterPro
Htur_3488	ADB62350	integral membrane sensor signal transducti...	CDD	UniProt	Pfam	InterPro
Htur_3658	ADB62520	histidine kinase [Haloterrigena turkmenica...	CDD	UniProt	Pfam	InterPro
Htur_3966	ADB62820	PAS/PAC sensor signal transduction histidi...	CDD	UniProt	Pfam	InterPro
Htur_4049	ADB62889	PAS/PAC sensor signal transduction histidi...	CDD	UniProt	Pfam	InterPro
Htur_4329	ADB63144	multi-sensor signal transduction histidine...	CDD	UniProt	Pfam	InterPro
Htur_4970	ADB63704	PAS/PAC sensor signal transduction histidi...	CDD	UniProt	Pfam	InterPro
Htur_5232	ADB63863	multi-sensor signal transduction histidine...	CDD	UniProt	Pfam	InterPro
**REC only**						
Htur_0362	ADB59262	response regulator receiver protein [Halot...	CDD	UniProt	Pfam	InterPro
Htur_0517	ADB59415	response regulator receiver protein [Halot...	CDD	UniProt	Pfam	InterPro
Htur_0524	ADB59422	response regulator receiver protein [Halot...	CDD	UniProt	Pfam	InterPro
Htur_0708	ADB59605	response regulator receiver protein [Halot...	CDD	UniProt	Pfam	InterPro
Htur_0749	ADB59646	response regulator receiver protein [Halot...	CDD	UniProt	Pfam	InterPro
Htur_0960	ADB59854	response regulator receiver protein [Halot...	CDD	UniProt	Pfam	InterPro
Htur_1247	ADB60137	response regulator receiver protein [Halot...	CDD	UniProt	Pfam	InterPro
Htur_1743	ADB60628	response regulator receiver protein [Halot...	CDD	UniProt	Pfam	InterPro
Htur_1954	ADB60839	response regulator receiver protein [Halot...	CDD	UniProt	Pfam	InterPro
Htur_2026	ADB60910	response regulator receiver protein [Halot...	CDD	UniProt	Pfam	InterPro
Htur_2120	ADB61003	response regulator receiver protein [Halot...	CDD	UniProt	Pfam	InterPro
Htur_2303	ADB61183	response regulator receiver protein [Halot...	CDD	UniProt	Pfam	InterPro

Htur_3139	ADB62004	response regulator receiver protein [Halot...	CDD	UniProt	Pfam	InterPro
Htur_3985	ADB62834	response regulator receiver protein [Halot...	CDD	UniProt	Pfam	InterPro
Htur_4516	ADB63324	response regulator receiver protein [Halot...	CDD	UniProt	Pfam	InterPro
Htur_4530	ADB63335	response regulator receiver protein [Halot...	CDD	UniProt	Pfam	InterPro
Htur_4797	ADB63567	response regulator receiver protein [Halot...	CDD	UniProt	Pfam	InterPro
Htur_5237	ADB63868	response regulator receiver protein [Halot...	CDD	UniProt	Pfam	InterPro
*Htur_4286	ADB63103	response regulator receiver protein [Halot...	CDD	UniProt	Pfam	InterPro
**REC-HisK**						
Htur_0657	ADB59554	multi-sensor signal transduction histidine...	CDD	UniProt	Pfam	InterPro
Htur_3297	ADB62161	multi-sensor signal transduction histidine...	CDD	UniProt	Pfam	InterPro
Htur_3337	ADB62201	multi-sensor signal transduction histidine...	CDD	UniProt	Pfam	InterPro
**REC-CheB**						
Htur_0959	ADB59853	response regulator receiver modulated CheB...	CDD	UniProt	Pfam	InterPro
**REC-HalX**						
Htur_0917	ADB59811	response regulator receiver protein [Halot...	CDD	UniProt	Pfam	InterPro
Htur_1264	ADB60154	response regulator receiver protein [Halot...	CDD	UniProt	Pfam	InterPro
Htur_1538	ADB60424	response regulator receiver protein [Halot...	CDD	UniProt	Pfam	InterPro
Htur_2134	ADB61017	response regulator receiver protein [Halot...	CDD	UniProt	Pfam	InterPro
**BAT**						
Htur_0128	ADB59029	Response regulator receiver modulated GAF ...	CDD	UniProt	Pfam	InterPro
Htur_0348	ADB59249	Response regulator receiver modulated GAF ...	CDD	UniProt	Pfam	InterPro
Htur_0709	ADB59606	Response regulator receiver modulated GAF ...	CDD	UniProt	Pfam	InterPro
Htur_2813	ADB61685	putative PAS/PAC sensor protein [Haloterri...	CDD	UniProt	Pfam	InterPro

---

Halovivax ruber XH-70

**HisK, no REC**						
Halru_0043	AGB14697	PAS domain S-box [Halovivax ruber XH-70] ...	CDD	UniProt	Pfam	InterPro
Halru_0110	AGB14762	signal transduction histidine kinase [Halo...	CDD	UniProt	Pfam	InterPro
Halru_0140	AGB14792	PAS domain S-box [Halovivax ruber XH-70] ...	CDD	UniProt	Pfam	InterPro
Halru_0212	AGB14858	signal transduction histidine kinase [Halo...	CDD	UniProt	Pfam	InterPro
Halru_0313	AGB14957	PAS domain S-box [Halovivax ruber XH-70] ...	CDD	UniProt	Pfam	InterPro
Halru_0412	AGB15055	PAS domain S-box [Halovivax ruber XH-70] ...	CDD	UniProt	Pfam	InterPro
Halru_0509	AGB15144	signal transduction histidine kinase [Halo...	CDD	UniProt	Pfam	InterPro
Halru_0510	AGB15145	PAS domain S-box [Halovivax ruber XH-70] ...	CDD	UniProt	Pfam	InterPro
Halru_0750	AGB15377	bacteriophytochrome (light-regulated signa...	CDD	UniProt	Pfam	InterPro
Halru_0813	AGB15439	PAS domain S-box [Halovivax ruber XH-70] ...	CDD	UniProt	Pfam	InterPro
Halru_1039	AGB15657	histidine kinase [Halovivax ruber XH-70] ...	CDD	UniProt	Pfam	InterPro
Halru_1142	AGB15759	PAS domain S-box [Halovivax ruber XH-70] ...	CDD	UniProt	Pfam	InterPro
Halru_1267	AGB15881	histidine kinase with PAS domain [Haloviva...	CDD	UniProt	Pfam	InterPro
Halru_1286	AGB15900	chemotaxis protein histidine kinase-like p...	CDD	UniProt	Pfam	InterPro
Halru_2424	AGB17006	PAS domain S-box [Halovivax ruber XH-70] ...	CDD	UniProt	Pfam	InterPro
Halru_2446	AGB17028	PAS domain S-box [Halovivax ruber XH-70] ...	CDD	UniProt	Pfam	InterPro
Halru_2589	AGB17170	PAS domain S-box [Halovivax ruber XH-70] ...	CDD	UniProt	Pfam	InterPro
**REC only**						
Halru_1284	AGB15898	response regulator containing CheY-like re...	CDD	UniProt	Pfam	InterPro
Halru_2111	AGB16702	response regulator with CheY-like receiver...	CDD	UniProt	Pfam	InterPro
**REC-HisK**						
Halru_0583	AGB15216	PAS domain S-box [Halovivax ruber XH-70].....	CDD	UniProt	Pfam	InterPro
Halru_2261	AGB16847	PAS domain S-box [Halovivax ruber XH-70].....	CDD	UniProt	Pfam	InterPro
Halru_2318	AGB16903	PAS domain S-box [Halovivax ruber XH-70].....	CDD	UniProt	Pfam	InterPro
**REC-CheB**						
Halru_1285	AGB15899	chemotaxis response regulator containing a...	CDD	UniProt	Pfam	InterPro
**REC-HalX**						
Halru_1141	AGB15758	response regulator with CheY-like receiver...	CDD	UniProt	Pfam	InterPro
Halru_1332	AGB15945	response regulator with CheY-like receiver...	CDD	UniProt	Pfam	InterPro
Halru_0139	AGB14791	response regulator with CheY-like receiver...	CDD	UniProt	Pfam	InterPro
**BAT**						
Halru_0454	AGB15092	response regulator with CheY-like receiver...	CDD	UniProt	Pfam	InterPro

---

halophilic archaeon DL31

**HisK, no REC**						
Halar_0348	AEN07604	PAS/PAC sensor signal transduction histidi...	CDD	UniProt	Pfam	InterPro
Halar_0535	AEN07772	PAS/PAC sensor signal transduction histidi...	CDD	UniProt	Pfam	InterPro
Halar_1252	AEN05001	multi-sensor signal transduction histidine...	CDD	UniProt	Pfam	InterPro
Halar_1860	AEN05570	integral membrane sensor signal transducti...	CDD	UniProt	Pfam	InterPro
Halar_1910	AEN05619	PAS/PAC sensor signal transduction histidi...	CDD	UniProt	Pfam	InterPro
Halar_2090	AEN05774	integral membrane sensor signal transducti...	CDD	UniProt	Pfam	InterPro
Halar_2135	AEN05817	histidine kinase [halophilic archaeon DL31...	CDD	UniProt	Pfam	InterPro
Halar_2293	AEN05965	histidine kinase [halophilic archaeon DL31...	CDD	UniProt	Pfam	InterPro
Halar_2384	AEN06047	PAS/PAC sensor signal transduction histidi...	CDD	UniProt	Pfam	InterPro
Halar_2680	AEN06325	multi-sensor signal transduction histidine...	CDD	UniProt	Pfam	InterPro
Halar_2797	AEN06432	multi-sensor signal transduction histidine...	CDD	UniProt	Pfam	InterPro
Halar_3242	AEN06855	multi-sensor signal transduction histidine...	CDD	UniProt	Pfam	InterPro
Halar_3393	AEN06996	multi-sensor signal transduction histidine...	CDD	UniProt	Pfam	InterPro
**REC only**						
Halar_0350	AEN07606	response regulator receiver protein (plasm...	CDD	UniProt	Pfam	InterPro
**REC-HisK**						

Halar_0299	AEN07558	multi-sensor signal transduction histidine...	CDD	UniProt	Pfam	InterPro	
Halar_1537	AEN05269	multi-sensor signal transduction histidine...	CDD	UniProt	Pfam	InterPro	
Halar_3160	AEN06776	multi-sensor signal transduction histidine...	CDD	UniProt	Pfam	InterPro	
**REC-HalX**							
Halar_1479	AEN05212	response regulator receiver protein [halop...	CDD	UniProt	Pfam	InterPro	
**BAT**							
Halar_2454	AEN06108	putative PAS/PAC sensor protein [halophili...	CDD	UniProt	Pfam	InterPro	

Methanobacterium congolense isolate Buetzberg

**HisK, no REC**							
MCBB_0068	SCG84656	putative sensor histidine kinase pdtaS [Me...	CDD	UniProt	Pfam	InterPro	
MCBB_0614	SCG85187	putative sensor histidine kinase pdtaS [Me...	CDD	UniProt	Pfam	InterPro	
MCBB_0628	SCG85201	Signal transduction histidine kinase [Meth...	CDD	UniProt	Pfam	InterPro	
MCBB_0815	SCG85380	putative sensor histidine kinase pdtaS [Me...	CDD	UniProt	Pfam	InterPro	
MCBB_1261	SCG85819	putative sensor histidine kinase pdtaS [Me...	CDD	UniProt	Pfam	InterPro	
MCBB_1296	SCG85854	Methyl sulfide methyltransferase-associate...	CDD	UniProt	Pfam	InterPro	
MCBB_1518	SCG86073	putative sensor histidine kinase pdtaS [Me...	CDD	UniProt	Pfam	InterPro	
MCBB_1772	SCG86323	putative sensor histidine kinase pdtaS [Me...	CDD	UniProt	Pfam	InterPro	
MCBB_1872	SCG86421	putative sensor histidine kinase pdtaS [Me...	CDD	UniProt	Pfam	InterPro	
MCBB_2314	SCG86852	putative sensor histidine kinase pdtaS [Me...	CDD	UniProt	Pfam	InterPro	
**REC only**							
MCBB_1548	SCG86103	putative histidine kinase 1 [Methanobacter...	CDD	UniProt	Pfam	InterPro	
MCBB_1688	SCG86243	Phyllosphere-induced regulator PhyR [Metha...	CDD	UniProt	Pfam	InterPro	
**REC-HisK**							
MCBB_0649	SCG85222	putative sensor histidine kinase pdtaS [Me...	CDD	UniProt	Pfam	InterPro	
MCBB_0916	SCG85476	Signal transduction histidine kinase [Meth...	CDD	UniProt	Pfam	InterPro	
MCBB_2021	SCG86569	putative transcriptional regulatory protei...	CDD	UniProt	Pfam	InterPro	
MCBB_2060	SCG86603	putative sensor histidine kinase pdtaS [Me...	CDD	UniProt	Pfam	InterPro	
**REC-PAS**							
MCBB_0027	SCG84616	putative transcriptional regulatory protei...	CDD	UniProt	Pfam	InterPro	
**REC-MetOD1**							
MCBB_1096	SCG85655	putative transcriptional regulatory protei...	CDD	UniProt	Pfam	InterPro	(REC-PAS-MetOD1)
MCBB_1432	SCG85988	putative transcriptional regulatory protei...	CDD	UniProt	Pfam	InterPro	(REC-MetOD1)

Methanobacterium formicicum BRM9

**HisK, no REC**						
BRM9_0417	AIS31244	signal transduction histidine kinase [Meth...	CDD	UniProt	Pfam	InterPro
BRM9_0432	AIS31257	histidine kinase/PAS domain-containing pro...	CDD	UniProt	Pfam	InterPro
BRM9_0435	AIS31260	histidine kinase/PAS domain-containing pro...	CDD	UniProt	Pfam	InterPro
BRM9_0748	AIS31568	signal transduction histidine kinase [Meth...	CDD	UniProt	Pfam	InterPro
BRM9_0978	AIS31795	two component system histidine kinase [Met...	CDD	UniProt	Pfam	InterPro
BRM9_1002	AIS31818	histidine kinase/PAS domain-containing pro...	CDD	UniProt	Pfam	InterPro
BRM9_1005	AIS31821	histidine kinase/PAS domain-containing pro...	CDD	UniProt	Pfam	InterPro
BRM9_1036	AIS31852	histidine kinase/PAS domain-containing pro...	CDD	UniProt	Pfam	InterPro
BRM9_1139	AIS31955	histidine kinase/PAS domain-containing pro...	CDD	UniProt	Pfam	InterPro
BRM9_1209	AIS32024	histidine kinase/PAS domain-containing pro...	CDD	UniProt	Pfam	InterPro
BRM9_1224	AIS32039	histidine kinase/PAS domain-containing pro...	CDD	UniProt	Pfam	InterPro
BRM9_1237	AIS32052	histidine kinase/PAS domain-containing pro...	CDD	UniProt	Pfam	InterPro
BRM9_1350	AIS32165	signal transduction histidine kinase [Meth...	CDD	UniProt	Pfam	InterPro
BRM9_1404	AIS32218	two component sytem histidine kinase [Meth...	CDD	UniProt	Pfam	InterPro
BRM9_1491	AIS32305	histidine kinase/PAS domain-containing pro...	CDD	UniProt	Pfam	InterPro
BRM9_1636	AIS32448	signal transduction histidine kinase [Meth...	CDD	UniProt	Pfam	InterPro
BRM9_1659	AIS32471	histidine kinase/PAS domain-containing pro...	CDD	UniProt	Pfam	InterPro
BRM9_1746	AIS32556	histidine kinase/PAS domain-containing pro...	CDD	UniProt	Pfam	InterPro
BRM9_1755	AIS32565	histidine kinase/PAS domain-containing pro...	CDD	UniProt	Pfam	InterPro
BRM9_1925	AIS32729	histidine kinase/PAS domain-containing pro...	CDD	UniProt	Pfam	InterPro
BRM9_1948	AIS32752	signal transduction histidine kinase [Meth...	CDD	UniProt	Pfam	InterPro
BRM9_2038	AIS32840	signal transduction histidine kinase [Meth...	CDD	UniProt	Pfam	InterPro
BRM9_2109	AIS32911	histidine kinase/PAS domain-containing pro...	CDD	UniProt	Pfam	InterPro
**REC only**						
BRM9_0637	AIS31460	response regulator domain containing prote...	CDD	UniProt	Pfam	InterPro
BRM9_0976	AIS31793	response regulator domain-containing prote...	CDD	UniProt	Pfam	InterPro
BRM9_1035	AIS31851	response regulator domain-containing prote...	CDD	UniProt	Pfam	InterPro
BRM9_1148	AIS31964	response regulator domain-containing prote...	CDD	UniProt	Pfam	InterPro
BRM9_1234	AIS32049	response regulator domain-containing prote...	CDD	UniProt	Pfam	InterPro
BRM9_1405	AIS32219	two component system response regulator [M...	CDD	UniProt	Pfam	InterPro
BRM9_1846	AIS32654	response regulator domain-containing prote...	CDD	UniProt	Pfam	InterPro
**REC-HisK**						
BRM9_0252	AIS31081	histidine kinase/PAS domain-containing pro...	CDD	UniProt	Pfam	InterPro
BRM9_0860	AIS31677	histidine kinase/PAS domain-containing pro...	CDD	UniProt	Pfam	InterPro
BRM9_0975	AIS31792	two component system histidine kinase/resp...	CDD	UniProt	Pfam	InterPro
BRM9_1144	AIS31960	histidine kinase/GAF/PAS domain-containin ...	CDD	UniProt	Pfam	InterPro
BRM9_1406	AIS32220	histidine kinase/PAS domain-containing pro...	CDD	UniProt	Pfam	InterPro
BRM9_2138	AIS32940	histidine kinase/response regulator hybrid...	CDD	UniProt	Pfam	InterPro
*BRM9_0748	AIS31568	signal transduction histidine kinase [Meth...	CDD	UniProt	Pfam	InterPro
**REC-PAS**						
BRM9_0068	AIS30901	response regulator/PAS domain-containing p...	CDD	UniProt	Pfam	InterPro

BRM9_0069	AIS30902	response regulator/PAS domain-containing p...	CDD	UniProt	Pfam	InterPro	
BRM9_0349	AIS31176	response regulator/PAS domain-containing p...	CDD	UniProt	Pfam	InterPro	
BRM9_0433	AIS31258	PAS domain-containing protein [Methanobact...	CDD	UniProt	Pfam	InterPro	
BRM9_0977	AIS31794	two component system response regulator [M...	CDD	UniProt	Pfam	InterPro	
BRM9_1034	AIS31850	response regulator/PAS domain-containing p...	CDD	UniProt	Pfam	InterPro	
BRM9_1049	AIS31865	PAS domain-containing protein [Methanobact...	CDD	UniProt	Pfam	InterPro	
BRM9_1233	AIS32048	response regulator/PAS domain-containing p...	CDD	UniProt	Pfam	InterPro	
BRM9_1414	AIS32228	PAS domain-containing protein [Methanobact...	CDD	UniProt	Pfam	InterPro	
**REC-GAF-PAS**							
BRM9_1144	AIS31960	histidine kinase/GAF/PAS domain-containing...	CDD	UniProt	Pfam	InterPro	
**REC-BcsA**							
BRM9_1475	AIS32289	glycosyl transferase GT2 family with respo...	CDD	UniProt	Pfam	InterPro	(REC-BcsA)
**REC-MetOD1**							
BRM9_1223	AIS32038	response regulator domain-containing prote...	CDD	UniProt	Pfam	InterPro	(REC-MetOD1)
BRM9_1398	AIS32213	response regulator/PAS domain-containing p...	CDD	UniProt	Pfam	InterPro	(REC-PAS-MetOD1)
BRM9_1407	AIS32221	response regulator domain-containing prote...	CDD	UniProt	Pfam	InterPro	(REC-MetOD1)

Methanobacterium lacus AL-21

**HisK, no REC**						
Metbo_0409	ADZ08661	signal transduction histidine kinase [Meth...	CDD	UniProt	Pfam	InterPro
Metbo_0448	ADZ08700	signal transduction histidine kinase [Meth...	CDD	UniProt	Pfam	InterPro
Metbo_0507	ADZ08759	signal transduction histidine kinase [Meth...	CDD	UniProt	Pfam	InterPro
Metbo_0937	ADZ09184	PAS/PAC sensor signal transduction histidi...	CDD	UniProt	Pfam	InterPro
Metbo_1039	ADZ09286	signal transduction histidine kinase [Meth...	CDD	UniProt	Pfam	InterPro
Metbo_1049	ADZ09296	PAS/PAC sensor signal transduction histidi...	CDD	UniProt	Pfam	InterPro
Metbo_1102	ADZ09347	histidine kinase [Methanobacterium lacus] ...	CDD	UniProt	Pfam	InterPro
Metbo_1235	ADZ09477	PAS/PAC sensor signal transduction histidi...	CDD	UniProt	Pfam	InterPro
Metbo_1328	ADZ09569	PAS/PAC sensor signal transduction histidi...	CDD	UniProt	Pfam	InterPro
Metbo_1329	ADZ09570	signal transduction histidine kinase [Meth...	CDD	UniProt	Pfam	InterPro
Metbo_1353	ADZ09593	signal transduction histidine kinase [Meth...	CDD	UniProt	Pfam	InterPro
Metbo_1417	ADZ09656	signal transduction histidine kinase [Meth...	CDD	UniProt	Pfam	InterPro
Metbo_1430	ADZ09668	signal transduction histidine kinase [Meth...	CDD	UniProt	Pfam	InterPro
Metbo_1445	ADZ09683	PAS/PAC sensor signal transduction histidi...	CDD	UniProt	Pfam	InterPro
Metbo_1514	ADZ09745	PAS/PAC sensor signal transduction histidi...	CDD	UniProt	Pfam	InterPro
Metbo_1550	ADZ09778	signal transduction histidine kinase [Meth...	CDD	UniProt	Pfam	InterPro
Metbo_1593	ADZ09820	signal transduction histidine kinase [Meth...	CDD	UniProt	Pfam	InterPro
Metbo_1622	ADZ09849	multi-sensor signal transduction histidine...	CDD	UniProt	Pfam	InterPro
Metbo_1624	ADZ09851	multi-sensor signal transduction histidine...	CDD	UniProt	Pfam	InterPro
Metbo_1625	ADZ09852	PAS/PAC sensor signal transduction histidi...	CDD	UniProt	Pfam	InterPro
Metbo_1666	ADZ09892	integral membrane sensor signal transducti...	CDD	UniProt	Pfam	InterPro
Metbo_1676	ADZ09902	signal transduction histidine kinase [Meth...	CDD	UniProt	Pfam	InterPro
Metbo_1775	ADZ09998	PAS/PAC sensor signal transduction histidi...	CDD	UniProt	Pfam	InterPro
Metbo_1783	ADZ10005	PAS/PAC sensor signal transduction histidi...	CDD	UniProt	Pfam	InterPro
Metbo_1823	ADZ10045	signal transduction histidine kinase [Meth...	CDD	UniProt	Pfam	InterPro
Metbo_1846	ADZ10068	signal transduction histidine kinase [Meth...	CDD	UniProt	Pfam	InterPro
Metbo_1865	ADZ10086	signal transduction histidine kinase [Meth...	CDD	UniProt	Pfam	InterPro
Metbo_2284	ADZ10498	multi-sensor signal transduction histidine...	CDD	UniProt	Pfam	InterPro
Metbo_2320	ADZ10534	signal transduction histidine kinase [Meth...	CDD	UniProt	Pfam	InterPro
**REC only**						
Metbo_1044	ADZ09291	response regulator receiver protein [Metha...	CDD	UniProt	Pfam	InterPro
Metbo_1079	ADZ09324	response regulator receiver [Methanobacter...	CDD	UniProt	Pfam	InterPro
Metbo_1223	ADZ09465	response regulator receiver protein [Metha...	CDD	UniProt	Pfam	InterPro
Metbo_1236	ADZ09478	response regulator receiver protein [Metha...	CDD	UniProt	Pfam	InterPro
Metbo_1101	ADZ09346	response regulator receiver protein [Metha...	CDD	UniProt	Pfam	InterPro
Metbo_1592	ADZ09819	response regulator receiver [Methanobacter...	CDD	UniProt	Pfam	InterPro
Metbo_1635	ADZ09861	hypothetical protein Metbo_1635 [Methanoba...	CDD	UniProt	Pfam	InterPro
Metbo_1742	ADZ09965	response regulator receiver protein [Metha...	CDD	UniProt	Pfam	InterPro
**REC-HisK**						
Metbo_0326	ADZ08578	signal transduction histidine kinase [Meth...	CDD	UniProt	Pfam	InterPro
Metbo_0757	ADZ09008	signal transduction histidine kinase [Meth...	CDD	UniProt	Pfam	InterPro
Metbo_1100	ADZ09345	signal transduction histidine kinase [Meth...	CDD	UniProt	Pfam	InterPro
Metbo_1412	ADZ09651	signal transduction histidine kinase [Meth...	CDD	UniProt	Pfam	InterPro
Metbo_1429	ADZ09667	signal transduction histidine kinase [Meth...	CDD	UniProt	Pfam	InterPro
Metbo_1591	ADZ09818	signal transduction histidine kinase [Meth...	CDD	UniProt	Pfam	InterPro
Metbo_1755	ADZ09978	signal transduction histidine kinase [Meth...	CDD	UniProt	Pfam	InterPro
Metbo_1895	ADZ10115	signal transduction histidine kinase [Meth...	CDD	UniProt	Pfam	InterPro
Metbo_1896	ADZ10116	signal transduction histidine kinase [Meth...	CDD	UniProt	Pfam	InterPro
**REC-PAS**						
Metbo_0064	ADZ08317	putative PAS/PAC sensor protein [Methanoba...	CDD	UniProt	Pfam	InterPro
Metbo_0065	ADZ08318	putative PAS/PAC sensor protein [Methanoba...	CDD	UniProt	Pfam	InterPro
Metbo_1237	ADZ09479	putative PAS/PAC sensor protein [Methanoba...	CDD	UniProt	Pfam	InterPro
Metbo_1351	ADZ09591	putative PAS/PAC sensor protein [Methanoba...	CDD	UniProt	Pfam	InterPro
Metbo_1352	ADZ09592	putative PAS/PAC sensor protein [Methanoba...	CDD	UniProt	Pfam	InterPro
Metbo_1828	ADZ10050	putative PAS/PAC sensor protein [Methanoba...	CDD	UniProt	Pfam	InterPro
Metbo_1872	ADZ10093	putative PAS/PAC sensor protein [Methanoba...	CDD	UniProt	Pfam	InterPro
Metbo_2061	ADZ10280	putative PAS/PAC sensor protein [Methanoba...	CDD	UniProt	Pfam	InterPro
Metbo_2319	ADZ10533	PAS sensor protein [Methanobacterium lacus...	CDD	UniProt	Pfam	InterPro
**REC-BcsA**						

Metbo_0838 ADZ09088 response regulator receiver protein [Metha... CDD UniProt Pfam InterPro (REC-BcsA)
REC-MetOD1
Metbo_0747 ADZ08998 response regulator receiver protein [Metha... CDD UniProt Pfam InterPro (REC-MetOD1)
Metbo_1099 ADZ09344 response regulator receiver protein [Metha... CDD UniProt Pfam InterPro (REC-MetOD1)
Metbo_1144 ADZ09388 putative PAS/PAC sensor protein [Methanoba... CDD UniProt Pfam InterPro (REC-PAS-MetOD1)
Other RRs
Metbo_1354 ADZ09594 response regulator receiver [Methanobacter... CDD UniProt Pfam InterPro (REC-Smc)
Metbo_1869 ADZ10090 response regulator receiver protein [Metha... CDD UniProt Pfam InterPro (REC-MetOD4)
Metbo_1895 ADZ10115 signal transduction histidine kinase [Meth... CDD UniProt Pfam InterPro (REC-HisK-PAS)

Methanobacterium paludis SWAN1
HisK, no REC
MSWAN_0174 AEG17220 signal transduction histidine kinase [Meth... CDD UniProt Pfam InterPro
MSWAN_0184 AEG17230 signal transduction histidine kinase [Meth... CDD UniProt Pfam InterPro
MSWAN_0338 AEG17382 signal transduction histidine kinase [Meth... CDD UniProt Pfam InterPro
MSWAN_1140 AEG18158 signal transduction histidine kinase [Meth... CDD UniProt Pfam InterPro
MSWAN_1308 AEG18325 signal transduction histidine kinase [Meth... CDD UniProt Pfam InterPro
MSWAN_1334 AEG18349 signal transduction histidine kinase [Meth... CDD UniProt Pfam InterPro
MSWAN_1380 AEG18394 signal transduction histidine kinase [Meth... CDD UniProt Pfam InterPro
MSWAN_1544 AEG18558 signal transduction histidine kinase [Meth... CDD UniProt Pfam InterPro
MSWAN_1581 AEG18594 signal transduction histidine kinase [Meth... CDD UniProt Pfam InterPro
MSWAN_1892 AEG18901 signal transduction histidine kinase [Meth... CDD UniProt Pfam InterPro
REC only
MSWAN_0629 AEG17665 response regulator receiver [Methanobacter... CDD UniProt Pfam InterPro
MSWAN_0852 AEG17879 response regulator receiver [Methanobacter... CDD UniProt Pfam InterPro
MSWAN_1141 AEG18159 response regulator receiver protein [Metha... CDD UniProt Pfam InterPro
MSWAN_1161 AEG18179 response regulator receiver [Methanobacter... CDD UniProt Pfam InterPro
REC-PAS
MSWAN_0023 AEG17071 putative PAS/PAC sensor protein [Methanoba... CDD UniProt Pfam InterPro
REC-MetOD1
MSWAN_1059 AEG18080 putative PAS/PAC sensor protein [Methanoba... CDD UniProt Pfam InterPro (REC-PAS-MetOD1)
MSWAN_1497 AEG18511 response regulator receiver protein [Metha... CDD UniProt Pfam InterPro (REC-MetOD1)

Methanobacterium sp. MB1
HisK, no REC
*MBMB1_0321 CDG64431 hypothetical protein MBMB1_0321 [Methanoba... CDD UniProt Pfam InterPro
MBMB1_0322 CDG64432 hypothetical protein MBMB1_0322 [Methanoba... CDD UniProt Pfam InterPro
MBMB1_0533 CDG64640 hypothetical protein MBMB1_0533 [Methanoba... CDD UniProt Pfam InterPro
MBMB1_0598 CDG64705 signal transduction histidine kinase [Meth... CDD UniProt Pfam InterPro
MBMB1_0705 CDG64809 hypothetical protein MBMB1_0705 [Methanoba... CDD UniProt Pfam InterPro
MBMB1_0843 CDG64946 hypothetical protein MBMB1_0843 [Methanoba... CDD UniProt Pfam InterPro
MBMB1_0850 CDG64953 hypothetical protein MBMB1_0850 [Methanoba... CDD UniProt Pfam InterPro
MBMB1_0873 CDG64975 hypothetical protein MBMB1_0873 [Methanoba... CDD UniProt Pfam InterPro
MBMB1_0981 CDG65083 hypothetical protein MBMB1_0981 [Methanoba... CDD UniProt Pfam InterPro
MBMB1_1027 CDG65128 hypothetical protein MBMB1_1027 [Methanoba... CDD UniProt Pfam InterPro
MBMB1_1035 CDG65136 hypothetical protein MBMB1_1035 [Methanoba... CDD UniProt Pfam InterPro
MBMB1_1140 CDG65241 hypothetical protein MBMB1_1140 [Methanoba... CDD UniProt Pfam InterPro
MBMB1_1198 CDG65298 sensory transduction histidine kinase [Met... CDD UniProt Pfam InterPro
MBMB1_1523 CDG65617 hypothetical protein MBMB1_1523 [Methanoba... CDD UniProt Pfam InterPro
MBMB1_1793 CDG65872 hypothetical protein MBMB1_1793 [Methanoba... CDD UniProt Pfam InterPro
REC only
MBMB1_0017 CDG64137 hypothetical protein MBMB1_0017 [Methanoba... CDD UniProt Pfam InterPro
MBMB1_0416 CDG64526 putative PAS/PAC sensor protein [Methanoba... CDD UniProt Pfam InterPro
MBMB1_0721 CDG64825 hypothetical protein MBMB1_0721 [Methanoba... CDD UniProt Pfam InterPro
MBMB1_0849 CDG64952 hypothetical protein MBMB1_0849 [Methanoba... CDD UniProt Pfam InterPro
MBMB1_0872 CDG64974 response regulator receiver protein [Metha... CDD UniProt Pfam InterPro
MBMB1_1199 CDG65299 response regulator receiver protein [Metha... CDD UniProt Pfam InterPro
MBMB1_1401 CDG65499 hypothetical protein MBMB1_1401 [Methanoba... CDD UniProt Pfam InterPro
*MBMB1_1402 CDG65500 hypothetical protein MBMB1_1402 [Methanoba... CDD UniProt Pfam InterPro
REC-HisK
MBMB1_0158 CDG64277 signal transduction histidine kinase [Meth... CDD UniProt Pfam InterPro
MBMB1_0726 CDG64830 hypothetical protein MBMB1_0726 [Methanoba... CDD UniProt Pfam InterPro
MBMB1_1200 CDG65300 sensory transduction regulatory protein [M... CDD UniProt Pfam InterPro
MBMB1_2041 CDG66115 hypothetical protein MBMB1_2041 [Methanoba... CDD UniProt Pfam InterPro
REC-PAS
MBMB1_0016 CDG64136 PAS/PAC sensor protein [Methanobacterium s... CDD UniProt Pfam InterPro
MBMB1_0871 CDG64973 putative PAS/PAC sensor protein [Methanoba... CDD UniProt Pfam InterPro
MBMB1_1794 CDG65873 hypothetical protein MBMB1_1794 [Methanoba... CDD UniProt Pfam InterPro
REC-BcsA
MBMB1_0720 CDG64824 sensory transduction regulatory protein [M... CDD UniProt Pfam InterPro (REC-BcsA)
REC-GAF-PAS
MBMB1_0980 CDG65082 hypothetical protein MBMB1_0980 [Methanoba... CDD UniProt Pfam InterPro
REC-MetOD1
MBMB1_1193 CDG65293 PAS/PAC sensor protein [Methanobacterium s... CDD UniProt Pfam InterPro (REC-PAS-MetOD1)
MBMB1_1201 CDG65301 response regulator receiver protein [Metha... CDD UniProt Pfam InterPro (REC-MetOD1)

Methanobrevibacter millerae

HisK, no REC

sm9_0230	ALT68035	signal transduction histidine kinase [Meth...	CDD	UniProt	Pfam	InterPro	
sm9_1617	ALT69385	signal transduction histidine kinase [Meth...	CDD	UniProt	Pfam	InterPro	
sm9_1618	ALT69386	signal transduction histidine kinase [Meth...	CDD	UniProt	Pfam	InterPro	
**REC only**							
sm9_1619	ALT69387	response regulator receiver domain-contain...	CDD	UniProt	Pfam	InterPro	
**REC-BcsA**							
sm9_1620	ALT69388	glycosyl transferase GT2 family with respo...	CDD	UniProt	Pfam	InterPro	(REC-BcsA)

Methanobrevibacter sp. AbM4

HisK, no REC

Abm4_0570	AGN16467	two-component system histidine kinase [Met...	CDD	UniProt	Pfam	InterPro	
**REC only**							
Abm4_0571	AGN16468	two-component system response regulator [M...	CDD	UniProt	Pfam	InterPro	
Abm4_0572	AGN16469	response regulator domain-containing prote...	CDD	UniProt	Pfam	InterPro	
**REC-BcsA**							
Abm4_0589	AGN16484	response regulator/glycosyl transferase GT...	CDD	UniProt	Pfam	InterPro	(REC-BcsA)
Abm4_0573	AGN16470	response regulator/glycosyl transferase GT...	CDD	UniProt	Pfam	InterPro	(REC-BcsA)

Methanocaldococcus bathoardescens

HisK, no REC

JH146_0930	AIJ05775	CheA signal transduction histidine kinase ...	CDD	UniProt	Pfam	InterPro
**REC only**						
JH146_0940	AIJ05785	response regulator receiver protein [Metha...	CDD	UniProt	Pfam	InterPro
**REC-CheB**						
JH146_0932	AIJ05777	response regulator receiver modulated CheB...	CDD	UniProt	Pfam	InterPro

---

Methanocaldococcus fervens AG86

HisK, no REC

Mefer_0640	ACV24459	CheA signal transduction histidine kinase ...	CDD	UniProt	Pfam	InterPro
Mefer_0648	ACV24467	integral membrane sensor signal transducti...	CDD	UniProt	Pfam	InterPro
**REC only**						
Mefer_0647	ACV24466	response regulator receiver protein [Metha...	CDD	UniProt	Pfam	InterPro
Mefer_1110	ACV24921	response regulator receiver protein [Metha...	CDD	UniProt	Pfam	InterPro
**REC-CheB**						
Mefer_0641	ACV24460	response regulator receiver modulated CheB...	CDD	UniProt	Pfam	InterPro

---

Methanocaldococcus infernus ME

HisK, no REC

Metin_0774	ADG13440	CheA signal transduction histidine kinase ...	CDD	UniProt	Pfam	InterPro
**REC only**						
Metin_0247	ADG12917	response regulator receiver protein [Metha...	CDD	UniProt	Pfam	InterPro
**REC-CheB**						
Metin_0773	ADG13439	response regulator receiver protein [Metha...	CDD	UniProt	Pfam	InterPro

---

Methanocaldococcus sp. FS406-22

HisK, no REC

MFS40622_0241	ADC68937	integral membrane sensor signal transdu...	CDD	UniProt	Pfam	InterPro
**REC only**						
MFS40622_0242	ADC68938	response regulator receiver protein [Me...	CDD	UniProt	Pfam	InterPro

---

Methanocaldococcus vulcanius M7

HisK, no REC

Metvu_0258	ACX72125	CheA signal transduction histidine kinase ...	CDD	UniProt	Pfam	InterPro
**REC only**						
Metvu_1497	ACX73350	response regulator receiver protein [Metha...	CDD	UniProt	Pfam	InterPro
**REC-CheB**						
Metvu_0257	ACX72124	chemotaxis response regulator protein-glut...	CDD	UniProt	Pfam	InterPro

---

Methanocella arvoryzae MRE50

HisK, no REC

LRC113	CAJ35133	predicted signal transduction histidine ki...	CDD	UniProt	Pfam	InterPro
LRC120	CAJ35139	predicted signal transduction histidine ki...	CDD	UniProt	Pfam	InterPro
LRC135	CAJ35150	predicted signal transduction histidine ki...	CDD	UniProt	Pfam	InterPro
LRC318	CAJ35292	putative signal transduction histidine kin...	CDD	UniProt	Pfam	InterPro
LRC339	CAJ35311	putative signal transduction histidine kin...	CDD	UniProt	Pfam	InterPro
LRC393	CAJ35355	predicted signal transduction histidine ki...	CDD	UniProt	Pfam	InterPro
LRC522	CAJ35461	putative signal transduction histidine kin...	CDD	UniProt	Pfam	InterPro
LRC571	CAJ35501	chemotaxis histidine kinase [Methanocella ...	CDD	UniProt	Pfam	InterPro
LRC596	CAJ35520	predicted signal transduction histidine ki...	CDD	UniProt	Pfam	InterPro
RCIX2	CAJ35524	predicted signal transduction histidine ki...	CDD	UniProt	Pfam	InterPro

RCIX121	CAJ35620	predicted signal transduction histidine ki...	CDD	UniProt	Pfam	InterPro
RCIX156	CAJ35647	predicted signal transduction histidine ki...	CDD	UniProt	Pfam	InterPro
RCIX160	CAJ35650	predicted signal transduction histidine ki...	CDD	UniProt	Pfam	InterPro
RCIX170	CAJ35658	predicted signal transduction histidine ki...	CDD	UniProt	Pfam	InterPro
RCIX420	CAJ35860	predicted signal transduction histidine ki...	CDD	UniProt	Pfam	InterPro
RCIX484	CAJ35913	putative signal transduction histidine kin...	CDD	UniProt	Pfam	InterPro
RCIX485	CAJ35914	putative signal transduction histidine kin...	CDD	UniProt	Pfam	InterPro
RCIX512	CAJ35938	putative signal transduction histidine kin...	CDD	UniProt	Pfam	InterPro
RCIX604	CAJ36011	putative signal transduction histidine kin...	CDD	UniProt	Pfam	InterPro
RCIX850	CAJ36211	putative signal transduction histidine kin...	CDD	UniProt	Pfam	InterPro
RCIX980	CAJ36316	putative signal transduction histidine kin...	CDD	UniProt	Pfam	InterPro
RCIX1081	CAJ36399	predicted signal transduction histidine ki...	CDD	UniProt	Pfam	InterPro
RCIX1082	CAJ36400	putative signal transduction histidine kin...	CDD	UniProt	Pfam	InterPro
RCIX1084	CAJ36401	putative signal transduction histidine kin...	CDD	UniProt	Pfam	InterPro
RCIX1160	CAJ36480	putative signal transduction histidine kin...	CDD	UniProt	Pfam	InterPro
RCIX1237	CAJ36539	putative signal transduction histidine kin...	CDD	UniProt	Pfam	InterPro
RCIX1277	CAJ36574	predicted signal transduction histidine ki...	CDD	UniProt	Pfam	InterPro
RCIX1290	CAJ36583	predicted signal transduction histidine ki...	CDD	UniProt	Pfam	InterPro
RCIX1688	CAJ36909	putative signal transduction histidine kin...	CDD	UniProt	Pfam	InterPro
RCIX1713	CAJ36932	putative signal transduction histidine kin...	CDD	UniProt	Pfam	InterPro
RCIX1753	CAJ36961	putative signal transduction histidine kin...	CDD	UniProt	Pfam	InterPro
RCIX1976	CAJ37138	signal transduction serine/threonine kinas...	CDD	UniProt	Pfam	InterPro
RCIX2013	CAJ37166	putative signal transduction histidine kin...	CDD	UniProt	Pfam	InterPro
RCIX2191	CAJ37312	putative signal transduction histidine kin...	CDD	UniProt	Pfam	InterPro
RCIX2208	CAJ37327	putative signal transduction histidine kin...	CDD	UniProt	Pfam	InterPro
RCIX2210	CAJ37329	putative signal transduction histidine kin...	CDD	UniProt	Pfam	InterPro
RCIX2211	CAJ37330	putative signal transduction histidine kin...	CDD	UniProt	Pfam	InterPro
RRC13	CAJ37807	putative signal transduction histidine kin...	CDD	UniProt	Pfam	InterPro
RRC48	CAJ37831	putative signal transduction histidine kin...	CDD	UniProt	Pfam	InterPro
RRC186	CAJ37936	putative signal transduction histidine kin...	CDD	UniProt	Pfam	InterPro
RRC192	CAJ37939	putative signal transduction histidine kin...	CDD	UniProt	Pfam	InterPro
RRC193	CAJ37940	putative signal transduction histidine kin...	CDD	UniProt	Pfam	InterPro
RRC194	CAJ37941	putative signal transduction histidine kin...	CDD	UniProt	Pfam	InterPro
RRC287	CAJ38021	predicted signal transduction receptor [Me...	CDD	UniProt	Pfam	InterPro
RRC477	CAJ38171	putative signal transduction histidine kin...	CDD	UniProt	Pfam	InterPro

REC only

LRC569	CAJ35499	chemotaxis response regulator [Methanocell...	CDD	UniProt	Pfam	InterPro
LRC597	CAJ35521	hypothetical protein LRC597 [Methanocella ...	CDD	UniProt	Pfam	InterPro
RRC476	CAJ38170	putative response regulator (CheY-like) [M...	CDD	UniProt	Pfam	InterPro
RRC490	CAJ38182	two-component system response regulator [M...	CDD	UniProt	Pfam	InterPro
RCIX443	CAJ35876	putative response regulator (CheY-like) [M...	CDD	UniProt	Pfam	InterPro
RCIX1840	CAJ37025	putative response regulator (CheY-like) [M...	CDD	UniProt	Pfam	InterPro
RCIX2162	CAJ37286	putative response regulator (CheY-like) [M...	CDD	UniProt	Pfam	InterPro
RCIX2509	CAJ37575	predicted response regulator [Methanocella...	CDD	UniProt	Pfam	InterPro
RCIX2510	CAJ37576	predicted response regulator [Methanocella...	CDD	UniProt	Pfam	InterPro

REC-CheB

LRC570	CAJ35500	chemotaxis methylesterase [Methanocella ar...	CDD	UniProt	Pfam	InterPro

REC-PAS/GAF

RRC407	CAJ38113	putative signal transduction protein [Meth...	CDD	UniProt	Pfam	InterPro

Other RRs

RCIX981	CAJ36317	putative signal transduction protein [Meth...	CDD	UniProt	Pfam	InterPro	(REC-PAS-GAF-DUF835)
RCIX1890	CAJ37065	putative response regulator [Methanocella ...	CDD	UniProt	Pfam	InterPro	(REC-MetOD2)
RCIX2113	CAJ37246	putative signal transduction protein [Meth...	CDD	UniProt	Pfam	InterPro	(REC-PAS-GAF-DUF835)

Methanocella conradii HZ254

HisK, no REC

Mtc_0427	AFC99194	putative PAS sensor signal transduction hi...	CDD	UniProt	Pfam	InterPro
Mtc_0845	AFC99606	GAF sensor signal transduction histidine k...	CDD	UniProt	Pfam	InterPro
Mtc_1041	AFC99797	Putative signal transduction histidine kin...	CDD	UniProt	Pfam	InterPro
Mtc_1115	AFC99871	Putative signal transduction histidine kin...	CDD	UniProt	Pfam	InterPro
Mtc_1316	AFD00070	putative PAS sensor signal transduction hi...	CDD	UniProt	Pfam	InterPro
Mtc_1320	AFD00074	putative PAS sensor signal transduction hi...	CDD	UniProt	Pfam	InterPro
Mtc_1498	AFD00250	Putative signal transduction histidine kin...	CDD	UniProt	Pfam	InterPro
Mtc_1573	AFD00325	putative PAS sensor signal transduction hi...	CDD	UniProt	Pfam	InterPro
Mtc_1616	AFD00366	Putative signal transduction histidine kin...	CDD	UniProt	Pfam	InterPro
Mtc_1630	AFD00379	putative PAS sensor signal transduction hi...	CDD	UniProt	Pfam	InterPro
Mtc_1919	AFD00659	Putative signal transduction histidine kin...	CDD	UniProt	Pfam	InterPro
Mtc_1951	AFD00691	putative PAS sensor signal transduction hi...	CDD	UniProt	Pfam	InterPro
Mtc_2005	AFD00745	putative PAS sensor signal transduction hi...	CDD	UniProt	Pfam	InterPro
Mtc_2045	AFD00784	Putative signal transduction histidine kin...	CDD	UniProt	Pfam	InterPro
Mtc_2055	AFD00794	putative PAS sensor signal transduction hi...	CDD	UniProt	Pfam	InterPro
Mtc_2109	AFD00847	putative PAS sensor signal transduction hi...	CDD	UniProt	Pfam	InterPro
Mtc_2288	AFD01023	Putative signal transduction histidine kin...	CDD	UniProt	Pfam	InterPro
Mtc_2385	AFD01116	putative PAS sensor signal transduction hi...	CDD	UniProt	Pfam	InterPro
Mtc_2386	AFD01117	putative PAS sensor signal transduction hi...	CDD	UniProt	Pfam	InterPro

REC only

Mtc_0021	AFC98796	Response regulator [Methanocella conradii ...	CDD	UniProt	Pfam	InterPro	
Mtc_0391	AFC99161	Response regulator [Methanocella conradii ...	CDD	UniProt	Pfam	InterPro	
Mtc_0429	AFC99196	Response regulator [Methanocella conradii ...	CDD	UniProt	Pfam	InterPro	
Mtc_0831	AFC99592	Response regulator [Methanocella conradii ...	CDD	UniProt	Pfam	InterPro	
Mtc_1156	AFC99912	Response regulator [Methanocella conradii ...	CDD	UniProt	Pfam	InterPro	
Mtc_1960	AFD00700	Response regulator [Methanocella conradii ...	CDD	UniProt	Pfam	InterPro	
Mtc_2141	AFD00879	Response regulator [Methanocella conradii ...	CDD	UniProt	Pfam	InterPro	
Mtc_2363	AFD01096	Response regulator [Methanocella conradii ...	CDD	UniProt	Pfam	InterPro	
**Other RRs**							
Mtc_0800	AFC99561	Response regulator [Methanocella conradii ...	CDD	UniProt	Pfam	InterPro	(REC-MetOD2)

Methanocella paludicola SANAE

HisK, no REC

MCP_0034	BAI60106	putative histidine kinase [Methanocella pa...	CDD	UniProt	Pfam	InterPro
MCP_0052	BAI60124	putative histidine kinase [Methanocella pa...	CDD	UniProt	Pfam	InterPro
MCP_0054	BAI60126	putative histidine kinase [Methanocella pa...	CDD	UniProt	Pfam	InterPro
MCP_0354	BAI60426	putative histidine kinase [Methanocella pa...	CDD	UniProt	Pfam	InterPro
MCP_0357	BAI60429	putative histidine kinase [Methanocella pa...	CDD	UniProt	Pfam	InterPro
MCP_0535	BAI60607	putative histidine kinase [Methanocella pa...	CDD	UniProt	Pfam	InterPro
MCP_0536	BAI60608	putative histidine kinase [Methanocella pa...	CDD	UniProt	Pfam	InterPro
MCP_0578	BAI60650	putative histidine kinase [Methanocella pa...	CDD	UniProt	Pfam	InterPro
MCP_0602	BAI60674	putative histidine kinase [Methanocella pa...	CDD	UniProt	Pfam	InterPro
MCP_0751	BAI60823	putative histidine kinase [Methanocella pa...	CDD	UniProt	Pfam	InterPro
MCP_0753	BAI60825	putative histidine kinase [Methanocella pa...	CDD	UniProt	Pfam	InterPro
MCP_0834	BAI60906	putative histidine kinase [Methanocella pa...	CDD	UniProt	Pfam	InterPro
MCP_1032	BAI61104	putative histidine kinase [Methanocella pa...	CDD	UniProt	Pfam	InterPro
MCP_1179	BAI61251	putative histidine kinase [Methanocella pa...	CDD	UniProt	Pfam	InterPro
MCP_1198	BAI61270	putative histidine kinase [Methanocella pa...	CDD	UniProt	Pfam	InterPro
MCP_1286	BAI61358	putative histidine kinase [Methanocella pa...	CDD	UniProt	Pfam	InterPro
MCP_1287	BAI61359	putative histidine kinase [Methanocella pa...	CDD	UniProt	Pfam	InterPro
MCP_1288	BAI61360	putative histidine kinase [Methanocella pa...	CDD	UniProt	Pfam	InterPro
MCP_1307	BAI61379	putative histidine kinase [Methanocella pa...	CDD	UniProt	Pfam	InterPro
MCP_1342	BAI61414	putative histidine kinase [Methanocella pa...	CDD	UniProt	Pfam	InterPro
MCP_1350	BAI61422	putative histidine kinase [Methanocella pa...	CDD	UniProt	Pfam	InterPro
MCP_1351	BAI61423	putative histidine kinase [Methanocella pa...	CDD	UniProt	Pfam	InterPro
MCP_1387	BAI61459	putative histidine kinase [Methanocella pa...	CDD	UniProt	Pfam	InterPro
MCP_1388	BAI61460	putative histidine kinase [Methanocella pa...	CDD	UniProt	Pfam	InterPro
MCP_1413	BAI61485	putative histidine kinase [Methanocella pa...	CDD	UniProt	Pfam	InterPro
MCP_1426	BAI61498	putative histidine kinase [Methanocella pa...	CDD	UniProt	Pfam	InterPro
MCP_1443	BAI61515	putative histidine kinase [Methanocella pa...	CDD	UniProt	Pfam	InterPro
MCP_1543	BAI61615	putative histidine kinase [Methanocella pa...	CDD	UniProt	Pfam	InterPro
MCP_1544	BAI61616	putative histidine kinase [Methanocella pa...	CDD	UniProt	Pfam	InterPro
MCP_1546	BAI61618	putative histidine kinase [Methanocella pa...	CDD	UniProt	Pfam	InterPro
MCP_1547	BAI61619	putative histidine kinase [Methanocella pa...	CDD	UniProt	Pfam	InterPro
MCP_1614	BAI61686	putative histidine kinase [Methanocella pa...	CDD	UniProt	Pfam	InterPro
MCP_1615	BAI61687	putative histidine kinase [Methanocella pa...	CDD	UniProt	Pfam	InterPro
MCP_1624	BAI61696	putative histidine kinase [Methanocella pa...	CDD	UniProt	Pfam	InterPro
MCP_1665	BAI61737	putative histidine kinase [Methanocella pa...	CDD	UniProt	Pfam	InterPro
MCP_1757	BAI61829	putative histidine kinase [Methanocella pa...	CDD	UniProt	Pfam	InterPro
MCP_1783	BAI61855	putative histidine kinase [Methanocella pa...	CDD	UniProt	Pfam	InterPro
MCP_1809	BAI61881	putative histidine kinase [Methanocella pa...	CDD	UniProt	Pfam	InterPro
MCP_1856	BAI61928	putative histidine kinase [Methanocella pa...	CDD	UniProt	Pfam	InterPro
MCP_1858	BAI61930	putative histidine kinase [Methanocella pa...	CDD	UniProt	Pfam	InterPro
MCP_1908	BAI61980	putative histidine kinase [Methanocella pa...	CDD	UniProt	Pfam	InterPro
MCP_2068	BAI62140	putative histidine kinase [Methanocella pa...	CDD	UniProt	Pfam	InterPro
MCP_2192	BAI62264	putative histidine kinase [Methanocella pa...	CDD	UniProt	Pfam	InterPro
MCP_2193	BAI62265	putative histidine kinase [Methanocella pa...	CDD	UniProt	Pfam	InterPro
MCP_2197	BAI62269	putative histidine kinase [Methanocella pa...	CDD	UniProt	Pfam	InterPro
MCP_2198	BAI62270	putative histidine kinase [Methanocella pa...	CDD	UniProt	Pfam	InterPro
MCP_2199	BAI62271	putative histidine kinase [Methanocella pa...	CDD	UniProt	Pfam	InterPro
MCP_2202	BAI62274	putative histidine kinase [Methanocella pa...	CDD	UniProt	Pfam	InterPro
MCP_2254	BAI62326	putative histidine kinase [Methanocella pa...	CDD	UniProt	Pfam	InterPro
MCP_2616	BAI62688	putative histidine kinase [Methanocella pa...	CDD	UniProt	Pfam	InterPro
MCP_2784	BAI62856	putative histidine kinase [Methanocella pa...	CDD	UniProt	Pfam	InterPro

REC only

MCP_1156	BAI61228	putative response regulator [Methanocella ...	CDD	UniProt	Pfam	InterPro
MCP_1545	BAI61617	putative response regulator [Methanocella ...	CDD	UniProt	Pfam	InterPro
MCP_1650	BAI61722	putative response regulator [Methanocella ...	CDD	UniProt	Pfam	InterPro
MCP_1750	BAI61822	putative response regulator [Methanocella ...	CDD	UniProt	Pfam	InterPro
MCP_2023	BAI62095	putative response regulator [Methanocella ...	CDD	UniProt	Pfam	InterPro
MCP_2581	BAI62653	putative response regulator [Methanocella ...	CDD	UniProt	Pfam	InterPro
*MCP_2605	BAI62677	putative response regulator [Methanocella ...	CDD	UniProt	Pfam	InterPro

REC-HisK

MCP_2132	BAI62204	putative hybrid sensor and regulator [Meth...	CDD	UniProt	Pfam	InterPro	
**Other RRs**							
MCP_0475	BAI60547	putative response regulator [Methanocella ...	CDD	UniProt	Pfam	InterPro	(REC-MetOD2)

Methanococcoides burtonii DSM 6242

HisK, no REC

Mbur_0083	ABE51103	HATPase domain-containing multisensor sign...	CDD	UniProt	Pfam	InterPro	
Mbur_0262	ABE51269	PAS sensor signal transduction histidine k...	CDD	UniProt	Pfam	InterPro	
Mbur_0312	ABE51314	Signal transduction histidine kinase [Meth...	CDD	UniProt	Pfam	InterPro	
Mbur_0361	ABE51356	Chemotaxis protein CheA [Methanococcoides ...	CDD	UniProt	Pfam	InterPro	
Mbur_0380	ABE51375	Histidine kinase domain protein [Methanoco...	CDD	UniProt	Pfam	InterPro	
Mbur_0400	ABE51392	Signal transduction histidine kinase [Meth...	CDD	UniProt	Pfam	InterPro	
Mbur_0510	ABE51492	HATPase domain-containing external sensor ...	CDD	UniProt	Pfam	InterPro	
Mbur_0639	ABE51610	pleC (non-motile and phage-resistance prot...	CDD	UniProt	Pfam	InterPro	
Mbur_0694	ABE51659	Signal transduction histidine kinase [Meth...	CDD	UniProt	Pfam	InterPro	
Mbur_0768	ABE51727	pleC-like signal transduction histidine ki...	CDD	UniProt	Pfam	InterPro	
Mbur_0820	ABE51775	pleC (Non-motile and phage-resistance prot...	CDD	UniProt	Pfam	InterPro	
Mbur_0842	ABE51795	Multisensor signal transduction histidine ...	CDD	UniProt	Pfam	InterPro	
Mbur_0913	ABE51858	GAF-containing Multisensor signal transduc...	CDD	UniProt	Pfam	InterPro	
Mbur_0950	ABE51892	fixL-like, HATPase domain-containing PAS s...	CDD	UniProt	Pfam	InterPro	
Mbur_0955	ABE51897	Multisensor signal transduction histidine ...	CDD	UniProt	Pfam	InterPro	
Mbur_0958	ABE51900	ATP-binding region, ATPase-like protein [M...	CDD	UniProt	Pfam	InterPro	
Mbur_1023	ABE51958	signal transduction histidine kinase, with...	CDD	UniProt	Pfam	InterPro	
Mbur_1024	ABE51959	HATPase domain-containing multisensor sign...	CDD	UniProt	Pfam	InterPro	
Mbur_1166	ABE52089	pleC-like multisensor signal transduction ...	CDD	UniProt	Pfam	InterPro	
Mbur_1247	ABE52169	Multisensor signal transduction histidine ...	CDD	UniProt	Pfam	InterPro	
Mbur_1264	ABE52185	Sensor protein yyG-like multisensor signal...	CDD	UniProt	Pfam	InterPro	
Mbur_2119	ABE52992	HATPase domain-containing multisensor sign...	CDD	UniProt	Pfam	InterPro	
Mbur_2120	ABE52993	bvgS precursor-like multisensor signal tra...	CDD	UniProt	Pfam	InterPro	
Mbur_2201	ABE53066	Multisensor signal transduction histidine ...	CDD	UniProt	Pfam	InterPro	
Mbur_2286	ABE53146	Multisensor signal transduction histidine ...	CDD	UniProt	Pfam	InterPro	
Mbur_2403	ABE53254	luxQ-like PAS sensor signal transduction h...	CDD	UniProt	Pfam	InterPro	
**REC only**							
Mbur_0359	ABE51354	CheY like protein [Methanococcoides burton...	CDD	UniProt	Pfam	InterPro	
Mbur_0834	ABE51788	response regulator receiver domain [Methan...	CDD	UniProt	Pfam	InterPro	
Mbur_0841	ABE51794	response regulator receiver domain [Methan...	CDD	UniProt	Pfam	InterPro	
Mbur_1557	ABE52463	response regulator receiver domain [Methan...	CDD	UniProt	Pfam	InterPro	
Mbur_2107	ABE52981	response regulator receiver domain [Methan...	CDD	UniProt	Pfam	InterPro	
Mbur_2285	ABE53145	response regulator receiver domain [Methan...	CDD	UniProt	Pfam	InterPro	
Mbur_2294	ABE53151	response regulator receiver domain [Methan...	CDD	UniProt	Pfam	InterPro	
Mbur_2445	ABE53296	response regulator receiver domain [Methan...	CDD	UniProt	Pfam	InterPro	
**REC-HisK**							
Mbur_1775	ABE52664	multisensor signal transduction histidine ...	CDD	UniProt	Pfam	InterPro	
Mbur_1899	ABE52781	multisensor signal transduction histidine ...	CDD	UniProt	Pfam	InterPro	
Mbur_2108	ABE52982	multisensor signal transduction histidine ...	CDD	UniProt	Pfam	InterPro	
Mbur_2414	ABE53265	multisensor signal transduction histidine ...	CDD	UniProt	Pfam	InterPro	
**REC-CheB**							
Mbur_0360	ABE51355	Protein-glutamate methylesterase CheB [Met...	CDD	UniProt	Pfam	InterPro	
**REC-PAS**							
Mbur_0825	ABE51779	response regulator receiver domain-contain...	CDD	UniProt	Pfam	InterPro	
Mbur_0883	ABE51832	response regulator receiver domain-contain...	CDD	UniProt	Pfam	InterPro	
**HTH-REC**							
Mbur_0695	ABE51660	**LtrR, low temperature responsive response ...**	CDD	UniProt	Pfam	InterPro	(HTH_11-REC)
**REC-MetOD1**							
Mbur_0878	ABE51827	response regulator receiver domain-contain...	CDD	UniProt	Pfam	InterPro	(REC-MetOD1)
**Other RRs**							
Mbur_2185	ABE53050	response regulator receiver domain-contain...	CDD	UniProt	Pfam	InterPro	(KaiC-REC)

Methanococcoides methylutens MM1

HisK, no REC

MCMEM_0377	AKB84430	Sensory transduction histidine kinase [Met...	CDD	UniProt	Pfam	InterPro
MCMEM_0435	AKB84488	Sensory transduction histidine kinase [Met...	CDD	UniProt	Pfam	InterPro
MCMEM_0824	AKB84877	Sensory transduction histidine kinase [Met...	CDD	UniProt	Pfam	InterPro
MCMEM_0922	AKB84975	Sensory transduction histidine kinase [Met...	CDD	UniProt	Pfam	InterPro
MCMEM_0972	AKB85025	hypothetical protein MCMEM_0972 [Methanoco...	CDD	UniProt	Pfam	InterPro
MCMEM_1047	AKB85100	Adenylate cyclase [Methanococcoides methyl...	CDD	UniProt	Pfam	InterPro
MCMEM_1159	AKB85212	hypothetical protein MCMEM_1159 [Methanoco...	CDD	UniProt	Pfam	InterPro
MCMEM_1168	AKB85221	hypothetical protein MCMEM_1168 [Methanoco...	CDD	UniProt	Pfam	InterPro
MCMEM_1188	AKB85241	Sensory transduction histidine kinase [Met...	CDD	UniProt	Pfam	InterPro
MCMEM_1247	AKB85300	sensory transduction histidine kinase [Met...	CDD	UniProt	Pfam	InterPro
MCMEM_1299	AKB85352	Sensory transduction histidine kinase [Met...	CDD	UniProt	Pfam	InterPro
MCMEM_1317	AKB85370	hypothetical protein MCMEM_1317 [Methanoco...	CDD	UniProt	Pfam	InterPro
MCMEM_1411	AKB85464	hypothetical protein MCMEM_1411 [Methanoco...	CDD	UniProt	Pfam	InterPro
MCMEM_1415	AKB85468	hypothetical protein MCMEM_1415 [Methanoco...	CDD	UniProt	Pfam	InterPro
MCMEM_1453	AKB85506	sensory transduction histidine kinase [Met...	CDD	UniProt	Pfam	InterPro
MCMEM_1462	AKB85515	sensory transduction histidine kinase [Met...	CDD	UniProt	Pfam	InterPro
MCMEM_1504	AKB85557	Sensory transduction histidine kinase [Met...	CDD	UniProt	Pfam	InterPro
MCMEM_1505	AKB85558	hypothetical protein MCMEM_1505 [Methanoco...	CDD	UniProt	Pfam	InterPro
MCMEM_1624	AKB85677	sensory transduction histidine kinase [Met...	CDD	UniProt	Pfam	InterPro
MCMEM_1635	AKB85688	hypothetical protein MCMEM_1635 [Methanoco...	CDD	UniProt	Pfam	InterPro
MCMEM_1950	AKB86003	hypothetical protein MCMEM_1950 [Methanoco...	CDD	UniProt	Pfam	InterPro
MCMEM_1951	AKB86004	hypothetical protein MCMEM_1951 [Methanoco...	CDD	UniProt	Pfam	InterPro

MCMEM_2043 AKB86096 Sensory transduction histidine kinase [Met... CDD UniProt Pfam InterPro
MCMEM_2044 AKB86097 Sensory transduction histidine kinase [Met... CDD UniProt Pfam InterPro
MCMEM_2046 AKB86099 hypothetical protein MCMEM_2046 [Methanoco... CDD UniProt Pfam InterPro
MCMEM_2069 AKB86122 hypothetical protein MCMEM_2069 [Methanoco... CDD UniProt Pfam InterPro
Hybrid HisK
MCMEM_1707 AKB85760 hypothetical protein MCMEM_1707 [Methanoco... CDD UniProt Pfam InterPro
REC only
MCMEM_0434 AKB84487 response regulator receiver [Methanococcoi... CDD UniProt Pfam InterPro
MCMEM_0441 AKB84494 sensory transduction regulatory protein [M... CDD UniProt Pfam InterPro
MCMEM_0964 AKB85017 Response regulator receiver [Methanococcoi... CDD UniProt Pfam InterPro
MCMEM_0971 AKB85024 response regulator receiver [Methanococcoi... CDD UniProt Pfam InterPro
MCMEM_0304 AKB84357 Two-component response regulator [Methanoc... CDD UniProt Pfam InterPro
MCMEM_2068 AKB86121 response regulator receiver [Methanococcoi... CDD UniProt Pfam InterPro
MCMEM_0583 AKB84636 DNA-binding response regulator KdpE [Metha... CDD UniProt Pfam InterPro
REC-HisK
MCMEM_0032 AKB84085 Sensory transduction histidine kinase [Met... CDD UniProt Pfam InterPro
MCMEM_0128 AKB84181 Pole remodelling regulatory diguanylate cy... CDD UniProt Pfam InterPro
MCMEM_0305 AKB84358 hypothetical protein MCMEM_0305 [Methanoco... CDD UniProt Pfam InterPro
MCMEM_0556 AKB84609 Sensory transduction histidine kinase [Met... CDD UniProt Pfam InterPro
REC-PAS
MCMEM_1276 AKB85329 response regulator receiver [Methanococcoi... CDD UniProt Pfam InterPro
HTH-REC
MCMEM_1410 AKB85463 Phosphate regulon transcriptional regulato... CDD UniProt Pfam InterPro
Other RRs
MCMEM_0362 AKB84415 Phosphate regulon transcriptional regulato... CDD UniProt Pfam InterPro (KaiC-REC)

Methanococcus aeolicus Nankai-3
HisK, no REC
Maeo_1437 ABR57013 integral membrane sensor signal transducti... CDD UniProt Pfam InterPro
REC only
Maeo_1436 ABR57012 response regulator receiver protein [Metha... CDD UniProt Pfam InterPro

---

Methanococcus maripaludis S2
HisK, no REC
MMP0927 CAF30483 Chemotaxis protein CheA [Methanococcus mar... CDD UniProt Pfam InterPro
MMP1120 CAF30676 ATP-binding protein, ATPase-like:Histidine... CDD UniProt Pfam InterPro
MMP1303 CAF30859 Sensory transduction histidine kinase [Met... CDD UniProt Pfam InterPro
REC only
MMP1304 CAF30860 Response regulator receiver [Methanococcus... CDD UniProt Pfam InterPro
MMP0933 CAF30489 chemotaxis protein CheY [Methanococcus mar... CDD UniProt Pfam InterPro
REC-CheB
MMP0926 CAF30482 chemotaxis protein CheB [Methanococcus mar... CDD UniProt Pfam InterPro

---

Methanococcus vannielii SB
HisK, no REC
Mevan_0140 ABR54051 integral membrane sensor signal transducti... CDD UniProt Pfam InterPro
Mevan_0142 ABR54053 PAS/PAC sensor signal transduction histidi... CDD UniProt Pfam InterPro
Mevan_0220 ABR54130 CheA signal transduction histidine kinase ... CDD UniProt Pfam InterPro
Mevan_0444 ABR54351 integral membrane sensor signal transducti... CDD UniProt Pfam InterPro
Mevan_0613 ABR54519 integral membrane sensor signal transducti... CDD UniProt Pfam InterPro
REC only
Mevan_0256 ABR54165 response regulator receiver protein [Metha... CDD UniProt Pfam InterPro
Mevan_0614 ABR54520 response regulator receiver protein [Metha... CDD UniProt Pfam InterPro
REC-CheB
Mevan_0219 ABR54129 response regulator receiver modulated CheB... CDD UniProt Pfam InterPro

---

Methanococcus voltae A3
HisK, no REC
Mvol_0817 ADI36476 integral membrane sensor signal transducti... CDD UniProt Pfam InterPro
Mvol_0949 ADI36606 CheA signal transduction histidine kinase ... CDD UniProt Pfam InterPro
REC only
Mvol_0816 ADI36475 response regulator receiver protein [Metha... CDD UniProt Pfam InterPro
Mvol_0954 ADI36611 response regulator receiver protein [Metha... CDD UniProt Pfam InterPro
REC-CheB
Mvol_0948 ADI36605 response regulator receiver modulated CheB... CDD UniProt Pfam InterPro

---

Methanocorpusculum labreanum Z
HisK, no REC
Mlab_1181 ABN07350 histidine kinase [Methanocorpusculum labre... CDD UniProt Pfam InterPro
Other RRs
Mlab_1182 ABN07351 response regulator receiver protein [Metha... CDD UniProt Pfam InterPro (no hits in archaea)

Methanoculleus bourgensis MS2

HisK, no REC

BN140_0081	CCJ35004	multi-sensor signal transduction histidine...	CDD	UniProt	Pfam	InterPro
BN140_0242	CCJ35165	sensor histidine kinase [Methanoculleus bo...	CDD	UniProt	Pfam	InterPro
BN140_0345	CCJ35268	multi-sensor signal transduction histidine...	CDD	UniProt	Pfam	InterPro
BN140_0614	CCJ35537	Signal transduction histidine kinase [Meth...	CDD	UniProt	Pfam	InterPro
BN140_0740	CCJ35663	multi-sensor signal transduction histidine...	CDD	UniProt	Pfam	InterPro
BN140_1222	CCJ36145	multi-sensor signal transduction histidine...	CDD	UniProt	Pfam	InterPro
BN140_1315	CCJ36238	multi-sensor signal transduction histidine...	CDD	UniProt	Pfam	InterPro
BN140_1385	CCJ36308	multi-sensor signal transduction histidine...	CDD	UniProt	Pfam	InterPro
BN140_1881	CCJ36804	two-component system, chemotaxis family, s...	CDD	UniProt	Pfam	InterPro
BN140_2059	CCJ36982	multi-sensor signal transduction histidine...	CDD	UniProt	Pfam	InterPro
BN140_2065	CCJ36988	multi-sensor signal transduction histidine...	CDD	UniProt	Pfam	InterPro
BN140_2169	CCJ37092	putative PAS/PAC sensor protein [Methanocu...	CDD	UniProt	Pfam	InterPro
BN140_2482	CCJ37405	integral membrane sensor signal transducti...	CDD	UniProt	Pfam	InterPro
BN140_2483	CCJ37406	Osmosensitive K+ channel histidine kinase ...	CDD	UniProt	Pfam	InterPro

HisKA, no HATPase

BN140_0148	CCJ35071	putative PAS/PAC sensor protein [Methanocu...	CDD	UniProt	Pfam	InterPro
BN140_0356	CCJ35279	putative PAS/PAC sensor protein [Methanocu...	CDD	UniProt	Pfam	InterPro
BN140_2063	CCJ36986	putative PAS/PAC sensor protein [Methanocu...	CDD	UniProt	Pfam	InterPro
BN140_2404	CCJ37327	Signal transduction histidine kinase [Meth...	CDD	UniProt	Pfam	InterPro

REC only

BN140_1883	CCJ36806	two-component system, chemotaxis family, r...	CDD	UniProt	Pfam	InterPro

REC-HisK

BN140_0479	CCJ35402	multi-sensor signal transduction histidine...	CDD	UniProt	Pfam	InterPro
BN140_0971	CCJ35894	multi-sensor signal transduction histidine...	CDD	UniProt	Pfam	InterPro
BN140_2006	CCJ36929	multi-sensor signal transduction histidine...	CDD	UniProt	Pfam	InterPro
BN140_2153	CCJ37076	multi-sensor signal transduction histidine...	CDD	UniProt	Pfam	InterPro

REC-CheB

BN140_1882	CCJ36805	two-component system, chemotaxis family, r...	CDD	UniProt	Pfam	InterPro

Other RRs

BN140_0178	CCJ35101	response regulator receiver protein [Metha...	CDD	UniProt	Pfam	InterPro	(REC-MetOD2)

Methanoculleus marisnigri JR1

HisK, no REC

Memar_0157	ABN56092	multi-sensor signal transduction histidine...	CDD	UniProt	Pfam	InterPro
Memar_0236	ABN56170	putative PAS/PAC sensor protein [Methanocu...	CDD	UniProt	Pfam	InterPro
Memar_0324	ABN56257	multi-sensor signal transduction histidine...	CDD	UniProt	Pfam	InterPro
Memar_0519	ABN56452	PAS/PAC sensor signal transduction histidi...	CDD	UniProt	Pfam	InterPro
Memar_0864	ABN56797	multi-sensor signal transduction histidine...	CDD	UniProt	Pfam	InterPro
Memar_0884	ABN56817	PAS/PAC sensor signal transduction histidi...	CDD	UniProt	Pfam	InterPro
Memar_0912	ABN56845	PAS/PAC sensor signal transduction histidi...	CDD	UniProt	Pfam	InterPro
Memar_0943	ABN56876	CheA signal transduction histidine kinases...	CDD	UniProt	Pfam	InterPro
Memar_1136	ABN57068	multi-sensor signal transduction histidine...	CDD	UniProt	Pfam	InterPro
Memar_1347	ABN57277	multi-sensor signal transduction histidine...	CDD	UniProt	Pfam	InterPro
Memar_1351	ABN57281	multi-sensor signal transduction histidine...	CDD	UniProt	Pfam	InterPro
Memar_1434	ABN57363	multi-sensor signal transduction histidine...	CDD	UniProt	Pfam	InterPro
Memar_1528	ABN57457	PAS/PAC sensor signal transduction histidi...	CDD	UniProt	Pfam	InterPro
Memar_1707	ABN57634	GAF sensor signal transduction histidine k...	CDD	UniProt	Pfam	InterPro
Memar_1777	ABN57703	PAS/PAC sensor signal transduction histidi...	CDD	UniProt	Pfam	InterPro
Memar_1981	ABN57907	PAS/PAC sensor signal transduction histidi...	CDD	UniProt	Pfam	InterPro
Memar_2020	ABN57946	multi-sensor signal transduction histidine...	CDD	UniProt	Pfam	InterPro
Memar_2025	ABN57951	multi-sensor signal transduction histidine...	CDD	UniProt	Pfam	InterPro
Memar_2127	ABN58050	multi-sensor signal transduction histidine...	CDD	UniProt	Pfam	InterPro
Memar_2182	ABN58105	multi-sensor signal transduction histidine...	CDD	UniProt	Pfam	InterPro
Memar_2343	ABN58266	integral membrane sensor signal transducti...	CDD	UniProt	Pfam	InterPro
Memar_2344	ABN58267	integral membrane sensor signal transducti...	CDD	UniProt	Pfam	InterPro
Memar_2345	ABN58268	integral membrane sensor signal transducti...	CDD	UniProt	Pfam	InterPro

Hybrid HisK

Memar_0238	ABN56172	CheA signal transduction histidine kinase ...	CDD	UniProt	Pfam	InterPro

HisKA, no HATPase

Memar_0270	ABN56204	putative PAS/PAC sensor protein [Methanocu...	CDD	UniProt	Pfam	InterPro
Memar_0421	ABN56354	putative PAS/PAC sensor protein [Methanocu...	CDD	UniProt	Pfam	InterPro
Memar_1139	ABN57071	putative PAS/PAC sensor protein [Methanocu...	CDD	UniProt	Pfam	InterPro
Memar_1158	ABN57089	putative PAS/PAC sensor protein [Methanocu...	CDD	UniProt	Pfam	InterPro
Memar_1348	ABN57278	putative PAS/PAC sensor protein [Methanocu...	CDD	UniProt	Pfam	InterPro
Memar_1355	ABN57285	signal transduction histidine kinase, nitr...	CDD	UniProt	Pfam	InterPro
Memar_2294	ABN58217	signal transduction histidine kinase, nitr...	CDD	UniProt	Pfam	InterPro

REC only

Memar_0945	ABN56878	response regulator receiver protein [Metha...	CDD	UniProt	Pfam	InterPro
Memar_2024	ABN57950	response regulator receiver protein [Metha...	CDD	UniProt	Pfam	InterPro
Memar_2126	ABN58049	response regulator receiver protein [Metha...	CDD	UniProt	Pfam	InterPro

REC-HisK

Memar_0237	ABN56171	response regulator receiver protein [Metha...	CDD	UniProt	Pfam	InterPro
Memar_0872	ABN56805	multi-sensor signal transduction histidine...	CDD	UniProt	Pfam	InterPro
Memar_1339	ABN57269	multi-sensor signal transduction histidine...	CDD	UniProt	Pfam	InterPro
Memar_1906	ABN57832	multi-sensor signal transduction histidine...	CDD	UniProt	Pfam	InterPro

REC-CheB

Memar_0944	ABN56877	response regulator receiver protein [Metha...	CDD	UniProt	Pfam	InterPro	

Other RRs

Memar_0295	ABN56229	response regulator receiver protein [Metha...	CDD	UniProt	Pfam	InterPro	(REC-MetOD2)
Memar_0309	ABN56242	response regulator receiver protein [Metha...	CDD	UniProt	Pfam	InterPro	(REC-MetOD2)

Methanoculleus sp. MAB1

HisK, no REC

MMAB1_0115	CVK31332	Multi-sensor signal transduction histidine...	CDD	UniProt	Pfam	InterPro
MMAB1_0302	CVK31519	Sensor histidine kinase ResE [Methanoculle...	CDD	UniProt	Pfam	InterPro
MMAB1_0432	CVK31649	Multi-sensor signal transduction histidine...	CDD	UniProt	Pfam	InterPro
MMAB1_0448	CVK31665	Putative PAS/PAC sensor protein (fragment)...	CDD	UniProt	Pfam	InterPro
MMAB1_0595	CVK31812	membrane protein of unknown function [Meth...	CDD	UniProt	Pfam	InterPro
MMAB1_0797	CVK32011	Aerobic respiration control sensor protein...	CDD	UniProt	Pfam	InterPro
MMAB1_0945	CVK32159	Sensor protein kdpD [Methanoculleus sp. MA...	CDD	UniProt	Pfam	InterPro
MMAB1_1547	CVK32760	Multi-sensor signal transduction histidine...	CDD	UniProt	Pfam	InterPro
MMAB1_1671	CVK32884	Alginate biosynthesis sensor protein kinB ...	CDD	UniProt	Pfam	InterPro
MMAB1_1773	CVK32986	Two-component system protein A [Methanocul...	CDD	UniProt	Pfam	InterPro
MMAB1_2418	CVK33631	Two-component system, chemotaxis family, s...	CDD	UniProt	Pfam	InterPro
MMAB1_2671	CVK33884	Sporulation kinase A [Methanoculleus sp. M...	CDD	UniProt	Pfam	InterPro
MMAB1_2680	CVK33893	Multi-sensor signal transduction histidine...	CDD	UniProt	Pfam	InterPro
MMAB1_2853	CVK34066	Signal transduction histidine-protein kina...	CDD	UniProt	Pfam	InterPro
MMAB1_3343	CVK34556	Signal transduction histidine-protein kina...	CDD	UniProt	Pfam	InterPro
MMAB1_3344	CVK34557	Sensor protein kdpD [Methanoculleus sp. MA...	CDD	UniProt	Pfam	InterPro

HisKA, no HATPase

MMAB1_0192	CVK31409	putative PAS/PAC sensor protein [Methanocu...	CDD	UniProt	Pfam	InterPro
MMAB1_2678	CVK33891	putative PAS/PAC sensor protein [Methanocu...	CDD	UniProt	Pfam	InterPro
MMAB1_2873	CVK34086	Sporulation kinase A [Methanoculleus sp. M...	CDD	UniProt	Pfam	InterPro
MMAB1_2961	CVK34174	putative PAS/PAC sensor protein [Methanocu...	CDD	UniProt	Pfam	InterPro
MMAB1_3199	CVK34412	putative PAS/PAC sensor protein [Methanocu...	CDD	UniProt	Pfam	InterPro

REC only

MMAB1_2420	CVK33633	regulator of chemotaxis and motility [Meth...	CDD	UniProt	Pfam	InterPro

REC-HisK

MMAB1_2597	CVK33810	Sensor protein kdpD [Methanoculleus sp. MA...	CDD	UniProt	Pfam	InterPro

REC-CheB

MMAB1_2419	CVK33632	Chemotaxis response regulator protein-glut...	CDD	UniProt	Pfam	InterPro

REC-PAS

MMAB1_2854	CVK34067	protein of unknown function [Methanoculleu...	CDD	UniProt	Pfam	InterPro	(REC-PAS)

Other RRs

MMAB1_0227	CVK31444	Protein pilH [Methanoculleus sp. MAB1] ...	CDD	UniProt	Pfam	InterPro	(REC-MetOD2)

Methanofollis liminatans DSM 4140

HisK, no REC

Metli_0189	EJG06164	histidine kinase [Methanofollis liminatans...	CDD	UniProt	Pfam	InterPro
Metli_0420	EJG06388	PAS/PAC sensor signal transduction histidi...	CDD	UniProt	Pfam	InterPro
Metli_0960	EJG06918	PAS/PAC sensor signal transduction histidi...	CDD	UniProt	Pfam	InterPro
Metli_1172	EJG07128	histidine kinase [Methanofollis liminatans...	CDD	UniProt	Pfam	InterPro
Metli_1245	EJG07201	CheA signal transduction histidine kinase ...	CDD	UniProt	Pfam	InterPro
Metli_1399	EJG07351	Cache sensor signal transduction histidine...	CDD	UniProt	Pfam	InterPro
Metli_1751	EJG07695	histidine kinase [Methanofollis liminatans...	CDD	UniProt	Pfam	InterPro
Metli_1822	EJG07766	histidine kinase [Methanofollis liminatans...	CDD	UniProt	Pfam	InterPro
Metli_2158	EJG08099	GAF sensor signal transduction histidine k...	CDD	UniProt	Pfam	InterPro
Metli_2218	EJG08159	multi-sensor signal transduction histidine...	CDD	UniProt	Pfam	InterPro
Metli_2328	EJG08266	PAS/PAC sensor signal transduction histidi...	CDD	UniProt	Pfam	InterPro
Metli_2453	EJG08390	PAS/PAC sensor signal transduction histidi...	CDD	UniProt	Pfam	InterPro
Metli_2464	EJG08401	multi-sensor signal transduction histidine...	CDD	UniProt	Pfam	InterPro

Hybrid HisK

Metli_0113	EJG06091	PAS/PAC sensor hybrid histidine kinase [Me...	CDD	UniProt	Pfam	InterPro
Metli_1831	EJG07775	PAS/PAC sensor hybrid histidine kinase [Me...	CDD	UniProt	Pfam	InterPro
Metli_1888	EJG07832	PAS/PAC sensor hybrid histidine kinase [Me...	CDD	UniProt	Pfam	InterPro
Metli_2455	EJG08392	CheA signal transduction histidine kinase ...	CDD	UniProt	Pfam	InterPro

HisKA, no HATPase

Metli_0101	EJG06079	putative PAS/PAC sensor protein [Methanofo...	CDD	UniProt	Pfam	InterPro

REC only

Metli_0541	EJG06509	response regulator receiver protein [Metha...	CDD	UniProt	Pfam	InterPro
Metli_1243	EJG07199	response regulator receiver protein [Metha...	CDD	UniProt	Pfam	InterPro
Metli_1398	EJG07350	response regulator receiver protein [Metha...	CDD	UniProt	Pfam	InterPro
Metli_2274	EJG08213	response regulator receiver protein [Metha...	CDD	UniProt	Pfam	InterPro

REC-HisK

Metli_0125	EJG06103	multi-sensor signal transduction histidine...	CDD	UniProt	Pfam	InterPro
Metli_1069	EJG07026	multi-sensor signal transduction histidine...	CDD	UniProt	Pfam	InterPro
Metli_1175	EJG07131	multi-sensor signal transduction histidine...	CDD	UniProt	Pfam	InterPro
Metli_1388	EJG07340	multi-sensor signal transduction histidine...	CDD	UniProt	Pfam	InterPro
Metli_1592	EJG07540	multi-sensor signal transduction histidine...	CDD	UniProt	Pfam	InterPro
Metli_2000	EJG07942	multi-sensor signal transduction histidine...	CDD	UniProt	Pfam	InterPro
Metli_2114	EJG08055	multi-sensor signal transduction histidine...	CDD	UniProt	Pfam	InterPro
Metli_2375	EJG08313	multi-sensor signal transduction histidine...	CDD	UniProt	Pfam	InterPro
Metli_2454	EJG08391	response regulator receiver sensor signal ...	CDD	UniProt	Pfam	InterPro

REC-CheB

Metli_1244 EJG07200 multi-sensor signal transduction histidine... CDD UniProt Pfam InterPro

REC-PAS

Metli_0300 EJG06272 putative PAS/PAC sensor protein [Methanofo... CDD UniProt Pfam InterPro
Metli_1397 EJG07349 putative PAS/PAC sensor protein [Methanofo... CDD UniProt Pfam InterPro
Metli_1585 EJG07535 putative PAS/PAC sensor protein [Methanofo... CDD UniProt Pfam InterPro
Metli_1993 EJG07935 putative PAS/PAC sensor protein [Methanofo... CDD UniProt Pfam InterPro

REC-PAS-GAF

Metli_1723 EJG07667 putative PAS/PAC sensor protein [Methanofo... CDD UniProt Pfam InterPro

Other RRs

Metli_1973 EJG07916 response regulator receiver protein [Metha... CDD UniProt Pfam InterPro (REC-MetOD2)

---

methanogenic archaeon ISO4-H5

HisKin

AR505_0788 AMH94509 signal transduction histidine kinase [meth... CDD UniProt Pfam InterPro
AR505_0791 AMH94512 transcriptional regulator LytS family [met... CDD UniProt Pfam InterPro
AR505_1170 AMH94885 signal transduction histidine kinase [meth... CDD UniProt Pfam InterPro

OmpR family (REC-wHTH)

AR505_0790 AMH94511 transcriptional regulator LytR family [met... CDD UniProt Pfam InterPro (REC-wHTH)

Methanohalobium evestigatum Z-7303

HisK, no REC

Metev_0371 ADI73293 integral membrane sensor signal transducti... CDD UniProt Pfam InterPro
Metev_0374 ADI73296 histidine kinase [Methanohalobium evestiga... CDD UniProt Pfam InterPro
Metev_0376 ADI73298 multi-sensor signal transduction histidine... CDD UniProt Pfam InterPro
Metev_0453 ADI73368 signal transduction histidine kinase [Meth... CDD UniProt Pfam InterPro
Metev_0761 ADI73662 signal transduction histidine kinase (plas... CDD UniProt Pfam InterPro
Metev_0867 ADI73765 CheA signal transduction histidine kinase ... CDD UniProt Pfam InterPro
Metev_0983 ADI73874 signal transduction histidine kinase [Meth... CDD UniProt Pfam InterPro
Metev_1007 ADI73896 signal transduction histidine kinase [Meth... CDD UniProt Pfam InterPro
Metev_1009 ADI73898 signal transduction histidine kinase [Meth... CDD UniProt Pfam InterPro
Metev_1091 ADI73975 PAS/PAC sensor signal transduction histidi... CDD UniProt Pfam InterPro
Metev_1139 ADI74021 PAS/PAC sensor signal transduction histidi... CDD UniProt Pfam InterPro
Metev_1140 ADI74022 PAS/PAC sensor signal transduction histidi... CDD UniProt Pfam InterPro
Metev_1154 ADI74035 multi-sensor signal transduction histidine... CDD UniProt Pfam InterPro
Metev_1155 ADI74036 PAS/PAC sensor signal transduction histidi... CDD UniProt Pfam InterPro
Metev_1156 ADI74037 PAS/PAC sensor signal transduction histidi... CDD UniProt Pfam InterPro
Metev_1164 ADI74045 PAS/PAC sensor signal transduction histidi... CDD UniProt Pfam InterPro
Metev_1165 ADI74046 signal transduction histidine kinase [Meth... CDD UniProt Pfam InterPro
Metev_1291 ADI74154 signal transduction histidine kinase [Meth... CDD UniProt Pfam InterPro
Metev_1454 ADI74308 signal transduction histidine kinase [Meth... CDD UniProt Pfam InterPro
Metev_1536 ADI74386 multi-sensor signal transduction histidine... CDD UniProt Pfam InterPro
Metev_1589 ADI74434 PAS/PAC sensor signal transduction histidi... CDD UniProt Pfam InterPro
Metev_1590 ADI74435 PAS/PAC sensor signal transduction histidi... CDD UniProt Pfam InterPro
Metev_1701 ADI74540 signal transduction histidine kinase [Meth... CDD UniProt Pfam InterPro
Metev_1711 ADI74550 PAS/PAC sensor signal transduction histidi... CDD UniProt Pfam InterPro
Metev_1743 ADI74581 signal transduction histidine kinase [Meth... CDD UniProt Pfam InterPro
Metev_1934 ADI74764 multi-sensor signal transduction histidine... CDD UniProt Pfam InterPro
Metev_2106 ADI74933 signal transduction histidine kinase [Meth... CDD UniProt Pfam InterPro
Metev_2266 ADI75085 multi-sensor signal transduction histidine... CDD UniProt Pfam InterPro
Metev_2315 ADI75127 signal transduction histidine kinase (plas... CDD UniProt Pfam InterPro

REC only

Metev_0049 ADI72981 response regulator receiver protein [Metha... CDD UniProt Pfam InterPro
Metev_0865 ADI73763 response regulator receiver protein [Metha... CDD UniProt Pfam InterPro
Metev_1008 ADI73897 response regulator receiver protein [Metha... CDD UniProt Pfam InterPro
Metev_1010 ADI73899 response regulator receiver protein [Metha... CDD UniProt Pfam InterPro
Metev_1867 ADI74700 response regulator receiver protein [Metha... CDD UniProt Pfam InterPro
Metev_2220 ADI75046 response regulator receiver protein [Metha... CDD UniProt Pfam InterPro
Metev_2337 ADI75149 response regulator receiver protein [Metha... CDD UniProt Pfam InterPro

REC-CheB

Metev_0866 ADI73764 response regulator receiver modulated CheB... CDD UniProt Pfam InterPro

REC-MetOD1

Metev_1159 ADI74040 response regulator receiver protein [Metha... CDD UniProt Pfam InterPro (REC-MetOD1)
Metev_1574 ADI74421 response regulator receiver protein [Metha... CDD UniProt Pfam InterPro (REC-MetOD1)

Other RRs

Metev_1321 ADI74181 response regulator receiver protein [Metha... CDD UniProt Pfam InterPro (KaiC-REC)

Methanohalophilus halophilus Z-7982

HisK, no REC

BHR79_00425 APH38092 hypothetical protein BHR79_00425 [Methano... CDD UniProt Pfam InterPro
BHR79_00710 APH38146 hypothetical protein BHR79_00710 [Methano... CDD UniProt Pfam InterPro
BHR79_00945 APH38186 Two-component sensor histidine kinase, co... CDD UniProt Pfam InterPro
BHR79_01420 APH38273 hypothetical protein BHR79_01420 [Methano... CDD UniProt Pfam InterPro
BHR79_01795 APH38341 hypothetical protein BHR79_01795 [Methano... CDD UniProt Pfam InterPro
BHR79_02095 APH38395 hypothetical protein BHR79_02095 [Methano... CDD UniProt Pfam InterPro
BHR79_03265 APH38600 hypothetical protein BHR79_03265 [Methano... CDD UniProt Pfam InterPro

BHR79_03370 APH38620 hypothetical protein BHR79_03370 [Methano... CDD UniProt Pfam InterPro
BHR79_03665 APH38671 hypothetical protein BHR79_03665 [Methano... CDD UniProt Pfam InterPro
BHR79_06215 APH39117 histidine kinase [Methanohalophilus halop... CDD UniProt Pfam InterPro
BHR79_06570 APH39180 hypothetical protein BHR79_06570 [Methano... CDD UniProt Pfam InterPro
BHR79_06695 APH39204 hypothetical protein BHR79_06695 [Methano... CDD UniProt Pfam InterPro
BHR79_07285 APH39303 hypothetical protein BHR79_07285 [Methano... CDD UniProt Pfam InterPro
BHR79_07290 APH39304 histidine kinase [Methanohalophilus halop... CDD UniProt Pfam InterPro
BHR79_07785 APH39390 histidine kinase [Methanohalophilus halop... CDD UniProt Pfam InterPro
BHR79_08165 APH39458 C50 carotenoid epsilon cyclase [Methanoha... CDD UniProt Pfam InterPro
BHR79_08245 APH39471 hypothetical protein BHR79_08245 [Methano... CDD UniProt Pfam InterPro
BHR79_08250 APH39472 hypothetical protein BHR79_08250 [Methano... CDD UniProt Pfam InterPro
BHR79_08590 APH39531 Two-component sensor histidine kinase, co... CDD UniProt Pfam InterPro
BHR79_08905 APH39586 hypothetical protein BHR79_08905 [Methano... CDD UniProt Pfam InterPro
BHR79_09380 APH39670 hypothetical protein BHR79_09380 [Methano... CDD UniProt Pfam InterPro
BHR79_09430 APH39680 histidine kinase [Methanohalophilus halop... CDD UniProt Pfam InterPro
BHR79_10040 APH39782 hypothetical protein BHR79_10040 [Methano... CDD UniProt Pfam InterPro
BHR79_10050 APH39783 hypothetical protein BHR79_10050 [Methano... CDD UniProt Pfam InterPro

Hybrid HisK

BHR79_02040 APH38385 hypothetical protein BHR79_02040 [Methano... CDD UniProt Pfam InterPro
BHR79_07310 APH39307 hypothetical protein BHR79_07310 [Methano... CDD UniProt Pfam InterPro

REC only

BHR79_00950 APH38187 two-component system response regulator [... CDD UniProt Pfam InterPro
BHR79_02035 APH38384 two-component system response regulator [... CDD UniProt Pfam InterPro
BHR79_04435 APH38809 two-component system response regulator [... CDD UniProt Pfam InterPro
BHR79_06225 APH39119 hypothetical protein BHR79_06225 [Methano... CDD UniProt Pfam InterPro
BHR79_06820 APH39226 two-component system response regulator [... CDD UniProt Pfam InterPro
BHR79_07790 APH39391 two-component system response regulator [... CDD UniProt Pfam InterPro
BHR79_07890 APH39407 hypothetical protein BHR79_07890 [Methano... CDD UniProt Pfam InterPro

REC-HisK

BHR79_00325 APH38076 hypothetical protein BHR79_00325 [Methano... CDD UniProt Pfam InterPro
BHR79_03895 APH38715 hypothetical protein BHR79_03895 [Methano... CDD UniProt Pfam InterPro

REC-PAS

BHR79_08490 APH39513 response regulator receiver protein [Meth... CDD UniProt Pfam InterPro

HTH-REC

BHR79_09425 APH39679 response regulator receiver protein [Meth... CDD UniProt Pfam InterPro

Other RRs

BHR79_01660 APH38317 response regulator receiver protein [Meth... CDD UniProt Pfam InterPro (KaiC-REC)
BHR79_08240 APH39470 hypothetical protein BHR79_08240 [Methano... CDD UniProt Pfam InterPro (REC-PAS-DUF835)
BHR79_08465 APH39508 response regulator [Methanohalophilus hal... CDD UniProt Pfam InterPro (REC-MetOD1)

Methanohalophilus mahii DSM 5219

HisK, no REC

Mmah_0019 ADE35557 histidine kinase [Methanohalophilus mahii ... CDD UniProt Pfam InterPro
Mmah_0110 ADE35646 PAS/PAC sensor signal transduction histidi... CDD UniProt Pfam InterPro
Mmah_0194 ADE35729 GAF sensor signal transduction histidine k... CDD UniProt Pfam InterPro
Mmah_0285 ADE35817 PAS/PAC sensor signal transduction histidi... CDD UniProt Pfam InterPro
Mmah_0343 ADE35875 PAS/PAC sensor signal transduction histidi... CDD UniProt Pfam InterPro
Mmah_0428 ADE35959 multi-sensor signal transduction histidine... CDD UniProt Pfam InterPro
Mmah_0432 ADE35962 PAS/PAC sensor signal transduction histidi... CDD UniProt Pfam InterPro
Mmah_0496 ADE36024 signal transduction histidine kinase [Meth... CDD UniProt Pfam InterPro
Mmah_0593 ADE36119 multi-sensor signal transduction histidine... CDD UniProt Pfam InterPro
Mmah_0821 ADE36344 integral membrane sensor signal transducti... CDD UniProt Pfam InterPro
Mmah_0831 ADE36354 PAS/PAC sensor signal transduction histidi... CDD UniProt Pfam InterPro
Mmah_0908 ADE36429 PAS/PAC sensor signal transduction histidi... CDD UniProt Pfam InterPro
Mmah_0912 ADE36433 multi-sensor signal transduction histidine... CDD UniProt Pfam InterPro
Mmah_0996 ADE36515 signal transduction histidine kinase [Meth... CDD UniProt Pfam InterPro
Mmah_1063 ADE36574 multi-sensor signal transduction histidine... CDD UniProt Pfam InterPro
Mmah_1151 ADE36657 signal transduction histidine kinase [Meth... CDD UniProt Pfam InterPro
Mmah_1249 ADE36753 PAS/PAC sensor signal transduction histidi... CDD UniProt Pfam InterPro
Mmah_1250 ADE36754 PAS/PAC sensor signal transduction histidi... CDD UniProt Pfam InterPro
Mmah_1394 ADE36893 PAS/PAC sensor signal transduction histidi... CDD UniProt Pfam InterPro
Mmah_1503 ADE36999 multi-sensor signal transduction histidine... CDD UniProt Pfam InterPro
Mmah_1558 ADE37054 multi-sensor signal transduction histidine... CDD UniProt Pfam InterPro
Mmah_1998 ADE37491 PAS/PAC sensor signal transduction histidi... CDD UniProt Pfam InterPro

REC only

Mmah_0297 ADE35829 response regulator receiver protein [Metha... CDD UniProt Pfam InterPro
Mmah_0495 ADE36023 response regulator receiver protein [Metha... CDD UniProt Pfam InterPro
Mmah_1130 ADE36637 response regulator receiver protein [Metha... CDD UniProt Pfam InterPro
Mmah_1150 ADE36656 response regulator receiver protein [Metha... CDD UniProt Pfam InterPro
Mmah_1368 ADE36867 response regulator receiver protein [Metha... CDD UniProt Pfam InterPro
Mmah_1501 ADE36997 response regulator receiver protein [Metha... CDD UniProt Pfam InterPro
Mmah_1843 ADE37338 response regulator receiver protein [Metha... CDD UniProt Pfam InterPro

REC-HisK

Mmah_0609 ADE36134 multi-sensor signal transduction protein [... CDD UniProt Pfam InterPro
Mmah_1948 ADE37443 multi-sensor signal transduction protein [... CDD UniProt Pfam InterPro

REC-PAS

Mmah_1016 ADE36534 putative PAS/PAC sensor protein [Methanoha... CDD UniProt Pfam InterPro

wHTH-REC

Mmah_0822	ADE36345	response regulator receiver protein [Metha...	CDD	UniProt	Pfam	InterPro	(wHTH-REC)

REC-MetOD1

Mmah_1021	ADE36539	response regulator receiver protein [Metha...	CDD	UniProt	Pfam	InterPro	(REC-MetOD1)

Other RRs

Mmah_0369	ADE35901	response regulator receiver protein [Metha...	CDD	UniProt	Pfam	InterPro	(KaiC-REC)

Methanolacinia petrolearia DSM 11571

HisK, no REC

Mpet_0012	ADN34793	signal transduction histidine kinase [Meth...	CDD	UniProt	Pfam	InterPro
Mpet_0033	ADN34814	multi-sensor signal transduction histidine...	CDD	UniProt	Pfam	InterPro
Mpet_0077	ADN34858	signal transduction histidine kinase [Meth...	CDD	UniProt	Pfam	InterPro
Mpet_0078	ADN34859	integral membrane sensor signal transducti...	CDD	UniProt	Pfam	InterPro
Mpet_0115	ADN34895	signal transduction histidine kinase regul...	CDD	UniProt	Pfam	InterPro
Mpet_0121	ADN34901	multi-sensor signal transduction histidine...	CDD	UniProt	Pfam	InterPro
Mpet_0171	ADN34949	signal transduction histidine kinase [Meth...	CDD	UniProt	Pfam	InterPro
Mpet_0181	ADN34959	signal transduction histidine kinase [Meth...	CDD	UniProt	Pfam	InterPro
Mpet_0299	ADN35075	signal transduction histidine kinase [Meth...	CDD	UniProt	Pfam	InterPro
Mpet_0442	ADN35216	multi-sensor signal transduction histidine...	CDD	UniProt	Pfam	InterPro
Mpet_0585	ADN35359	multi-sensor signal transduction histidine...	CDD	UniProt	Pfam	InterPro
Mpet_0596	ADN35370	PAS/PAC sensor signal transduction histidi...	CDD	UniProt	Pfam	InterPro
Mpet_0645	ADN35419	multi-sensor signal transduction histidine...	CDD	UniProt	Pfam	InterPro
Mpet_0678	ADN35452	multi-sensor signal transduction histidine...	CDD	UniProt	Pfam	InterPro
Mpet_0829	ADN35600	integral membrane sensor signal transducti...	CDD	UniProt	Pfam	InterPro
Mpet_1108	ADN35874	multi-sensor signal transduction histidine...	CDD	UniProt	Pfam	InterPro
Mpet_1128	ADN35894	PAS/PAC sensor signal transduction histidi...	CDD	UniProt	Pfam	InterPro
Mpet_1138	ADN35904	multi-sensor signal transduction histidine...	CDD	UniProt	Pfam	InterPro
Mpet_1153	ADN35919	signal transduction histidine kinase [Meth...	CDD	UniProt	Pfam	InterPro
Mpet_1205	ADN35966	histidine kinase [Methanolacinia petrolear...	CDD	UniProt	Pfam	InterPro
Mpet_1492	ADN36250	PAS/PAC sensor signal transduction histidi...	CDD	UniProt	Pfam	InterPro
Mpet_1802	ADN36555	multi-sensor signal transduction histidine...	CDD	UniProt	Pfam	InterPro
Mpet_2066	ADN36814	CheA signal transduction histidine kinase ...	CDD	UniProt	Pfam	InterPro
Mpet_2523	ADN37267	PAS/PAC sensor signal transduction histidi...	CDD	UniProt	Pfam	InterPro
Mpet_2600	ADN37343	integral membrane sensor signal transducti...	CDD	UniProt	Pfam	InterPro
Mpet_2671	ADN37414	integral membrane sensor signal transducti...	CDD	UniProt	Pfam	InterPro
Mpet_2707	ADN37450	signal transduction histidine kinase [Meth...	CDD	UniProt	Pfam	InterPro
Mpet_2735	ADN37478	signal transduction histidine kinase [Meth...	CDD	UniProt	Pfam	InterPro
Mpet_2799	ADN37542	putative PAS/PAC sensor protein [Methanola...	CDD	UniProt	Pfam	InterPro
Mpet_2810	ADN37553	histidine kinase [Methanolacinia petrolear...	CDD	UniProt	Pfam	InterPro
Mpet_2819	ADN37561	multi-sensor signal transduction histidine...	CDD	UniProt	Pfam	InterPro

Hybrid HisK

Mpet_0061	ADN34842	multi-sensor hybrid histidine kinase [Meth...	CDD	UniProt	Pfam	InterPro

HisKA, no HATPase

Mpet_1496	ADN36253	putative PAS/PAC sensor protein [Methanola...	CDD	UniProt	Pfam	InterPro
Mpet_1966	ADN36716	putative methyl-accepting chemotaxis senso...	CDD	UniProt	Pfam	InterPro
Mpet_1967	ADN36717	putative sensor with HAMP domain [Methanol...	CDD	UniProt	Pfam	InterPro
Mpet_1968	ADN36718	putative methyl-accepting chemotaxis senso...	CDD	UniProt	Pfam	InterPro
Mpet_2120	ADN36868	putative PAS/PAC sensor protein [Methanola...	CDD	UniProt	Pfam	InterPro
Mpet_2690	ADN37433	putative PAS/PAC sensor protein [Methanola...	CDD	UniProt	Pfam	InterPro
Mpet_2746	ADN37489	putative PAS/PAC sensor protein [Methanola...	CDD	UniProt	Pfam	InterPro

REC only

Mpet_0013	ADN34794	response regulator receiver protein [Metha...	CDD	UniProt	Pfam	InterPro
Mpet_0182	ADN34960	response regulator receiver protein [Metha...	CDD	UniProt	Pfam	InterPro
Mpet_0586	ADN35360	response regulator receiver protein [Metha...	CDD	UniProt	Pfam	InterPro
Mpet_0594	ADN35368	response regulator receiver protein [Metha...	CDD	UniProt	Pfam	InterPro
Mpet_1207	ADN35968	response regulator receiver protein [Metha...	CDD	UniProt	Pfam	InterPro
Mpet_2064	ADN36812	response regulator receiver protein [Metha...	CDD	UniProt	Pfam	InterPro
Mpet_2121	ADN36869	response regulator receiver protein [Metha...	CDD	UniProt	Pfam	InterPro

REC-HisK

Mpet_0180	ADN34958	signal transduction histidine kinase [Meth...	CDD	UniProt	Pfam	InterPro
Mpet_0288	ADN35065	putative PAS/PAC sensor protein [Methanola...	CDD	UniProt	Pfam	InterPro
Mpet_0399	ADN35173	multi-sensor signal transduction histidine...	CDD	UniProt	Pfam	InterPro
Mpet_0602	ADN35376	multi-sensor signal transduction histidine...	CDD	UniProt	Pfam	InterPro
Mpet_1059	ADN35826	signal transduction histidine kinase [Meth...	CDD	UniProt	Pfam	InterPro
Mpet_1150	ADN35916	multi-sensor signal transduction histidine...	CDD	UniProt	Pfam	InterPro
Mpet_1963	ADN36713	multi-sensor signal transduction histidine...	CDD	UniProt	Pfam	InterPro
Mpet_2338	ADN37085	multi-sensor signal transduction histidine...	CDD	UniProt	Pfam	InterPro
Mpet_2562	ADN37306	multi-sensor signal transduction histidine...	CDD	UniProt	Pfam	InterPro
Mpet_2802	ADN37545	multi-sensor signal transduction histidine...	CDD	UniProt	Pfam	InterPro

REC-CheB

Mpet_2065	ADN36813	response regulator receiver modulated CheB...	CDD	UniProt	Pfam	InterPro

REC-PAS

Mpet_0059	ADN34840	putative PAS/PAC sensor protein [Methanola...	CDD	UniProt	Pfam	InterPro
Mpet_2605	ADN37348	putative PAS/PAC sensor protein [Methanola...	CDD	UniProt	Pfam	InterPro
Mpet_0266	ADN35044	response regulator receiver protein [Metha...	CDD	UniProt	Pfam	InterPro
Mpet_2524	ADN37268	putative PAS/PAC sensor protein [Methanola...	CDD	UniProt	Pfam	InterPro
Mpet_2767	ADN37510	putative PAS/PAC sensor protein [Methanola...	CDD	UniProt	Pfam	InterPro
Mpet_2772	ADN37515	response regulator receiver protein [Metha...	CDD	UniProt	Pfam	InterPro

Other RRs

Mpet_0172	ADN34950	putative PAS/PAC sensor protein [Methanola...	CDD	UniProt	Pfam	InterPro	(REC-PAS-PAS-HisKA)
Mpet_0183	ADN34961	response regulator receiver protein [Metha...	CDD	UniProt	Pfam	InterPro	(REC-PAS-HisKA)
Mpet_1022	ADN35789	putative PAS/PAC sensor protein [Methanola...	CDD	UniProt	Pfam	InterPro	(REC-PAS-PAS-HisKA)
Mpet_1107	ADN35873	putative PAS/PAC sensor protein [Methanola...	CDD	UniProt	Pfam	InterPro	(REC-PAS-PAS-GAF-PAS-HisKA)
Mpet_1172	ADN35935	putative PAS/PAC sensor protein [Methanola...	CDD	UniProt	Pfam	InterPro	(REC-PAS-PAS-GAF-PAS-HisKA)
Mpet_1739	ADN36492	response regulator receiver protein [Metha...	CDD	UniProt	Pfam	InterPro	(REC-MetOD2)

Methanolobus psychrophilus R15

**HisK, no REC**							
Mpsy_0054	AFV22267	multisensor signal transduction histidine ...	CDD	UniProt	Pfam	InterPro	
Mpsy_0073	AFV22286	multisensor signal transduction histidine ...	CDD	UniProt	Pfam	InterPro	
Mpsy_0208	AFV22421	multi-sensor hybrid histidine kinase [Meth...	CDD	UniProt	Pfam	InterPro	
Mpsy_0241	AFV22454	two-component hybrid sensor and regulator ...	CDD	UniProt	Pfam	InterPro	
Mpsy_0354	AFV22565	PAS/PAC sensor signal transduction histidi...	CDD	UniProt	Pfam	InterPro	
Mpsy_0363	AFV22574	PAS/PAC sensor signal transduction histidi...	CDD	UniProt	Pfam	InterPro	
Mpsy_0517	AFV22728	multi-sensor signal transduction histidine...	CDD	UniProt	Pfam	InterPro	
Mpsy_0812	AFV23021	signal transduction histidine kinase [Meth...	CDD	UniProt	Pfam	InterPro	
Mpsy_0825	AFV23034	sensory transduction histidine kinase [Met...	CDD	UniProt	Pfam	InterPro	
Mpsy_0872	AFV23081	PAS/PAC sensor signal transduction histidi...	CDD	UniProt	Pfam	InterPro	
Mpsy_1040	AFV23249	hypothetical protein Mpsy_1040 [Methanolob...	CDD	UniProt	Pfam	InterPro	
Mpsy_1174	AFV23382	PAS/PAC sensor signal transduction histidi...	CDD	UniProt	Pfam	InterPro	
Mpsy_1192	AFV23400	PAS/PAC sensor signal transduction histidi...	CDD	UniProt	Pfam	InterPro	
Mpsy_1204	AFV23412	PAS/PAC sensor signal transduction histidi...	CDD	UniProt	Pfam	InterPro	
Mpsy_1239	AFV23447	PAS/PAC sensor signal transduction histidi...	CDD	UniProt	Pfam	InterPro	
Mpsy_1251	AFV23459	integral membrane sensor signal transducti...	CDD	UniProt	Pfam	InterPro	
Mpsy_1297	AFV23505	PAS/PAC sensor signal transduction histidi...	CDD	UniProt	Pfam	InterPro	
Mpsy_1547	AFV23754	multi-sensor signal transduction histidine...	CDD	UniProt	Pfam	InterPro	
Mpsy_1696	AFV23903	signal transduction histidine kinase [Meth...	CDD	UniProt	Pfam	InterPro	
Mpsy_1718	AFV23925	sensory transduction histidine kinase [Met...	CDD	UniProt	Pfam	InterPro	
Mpsy_1737	AFV23944	PAS/PAC sensor signal transduction histidi...	CDD	UniProt	Pfam	InterPro	
Mpsy_2042	AFV24248	multi-sensor signal transduction histidine...	CDD	UniProt	Pfam	InterPro	
Mpsy_2220	AFV24424	PAS/PAC sensor signal transduction histidi...	CDD	UniProt	Pfam	InterPro	
Mpsy_2256	AFV24460	sensory transduction histidine kinase [Met...	CDD	UniProt	Pfam	InterPro	
Mpsy_2598	AFV24801	signal transduction histidine kinase CheA ...	CDD	UniProt	Pfam	InterPro	
Mpsy_2638	AFV24841	sensory transduction histidine kinase [Met...	CDD	UniProt	Pfam	InterPro	
Mpsy_2783	AFV24984	PAS/PAC sensor signal transduction histidi...	CDD	UniProt	Pfam	InterPro	
Mpsy_2808	AFV25009	PAS/PAC sensor signal transduction histidi...	CDD	UniProt	Pfam	InterPro	
Mpsy_2981	AFV25180	putative Histidine kinase [Methanolobus ps...	CDD	UniProt	Pfam	InterPro	
Mpsy_3073	AFV25272	PAS/PAC sensor signal transduction histidi...	CDD	UniProt	Pfam	InterPro	
**Hybrid HisK**							
Mpsy_0031	AFV22244	PAS domain protein [Methanolobus psychroph...	CDD	UniProt	Pfam	InterPro	
Mpsy_0087	AFV22300	multi-sensor hybrid histidine kinase [Meth...	CDD	UniProt	Pfam	InterPro	
Mpsy_1288	AFV23496	two component system histidine kinase [Met...	CDD	UniProt	Pfam	InterPro	
Mpsy_2824	AFV25023	multi-sensor hybrid histidine kinase [Meth...	CDD	UniProt	Pfam	InterPro	
Mpsy_2980	AFV25179	multi-sensor hybrid histidine kinase [Meth...	CDD	UniProt	Pfam	InterPro	
Mpsy_3068	AFV25267	PAS domain protein [Methanolobus psychroph...	CDD	UniProt	Pfam	InterPro	
**REC only**							
Mpsy_0893	AFV23102	response regulator receiver protein [Metha...	CDD	UniProt	Pfam	InterPro	
Mpsy_1286	AFV23494	response regulator receiver protein [Metha...	CDD	UniProt	Pfam	InterPro	
Mpsy_1697	AFV23904	response regulator receiver protein [Metha...	CDD	UniProt	Pfam	InterPro	
Mpsy_1719	AFV23926	response regulator receiver [Methanolobus ...	CDD	UniProt	Pfam	InterPro	
Mpsy_2043	AFV24249	response regulator receiver protein [Metha...	CDD	UniProt	Pfam	InterPro	
Mpsy_2344	AFV24548	response regulator receiver [Methanolobus ...	CDD	UniProt	Pfam	InterPro	
Mpsy_2600	AFV24803	response regulator receiver protein [Metha...	CDD	UniProt	Pfam	InterPro	
Mpsy_2847	AFV25046	response regulator receiver protein [Metha...	CDD	UniProt	Pfam	InterPro	
**REC-HisK**							
Mpsy_1340	AFV23548	multisensor signal transduction histidine ...	CDD	UniProt	Pfam	InterPro	
Mpsy_1428	AFV23636	PAS/PAC sensor signal transduction histidi...	CDD	UniProt	Pfam	InterPro	
Mpsy_2044	AFV24250	PAS/PAC sensor signal transduction histidi...	CDD	UniProt	Pfam	InterPro	
Mpsy_2480	AFV24684	PAS/PAC sensor signal transduction histidi...	CDD	UniProt	Pfam	InterPro	
Mpsy_3013	AFV25212	multisensor signal transduction histidine ...	CDD	UniProt	Pfam	InterPro	
**REC-CheB**							
Mpsy_2599	AFV24802	chemotaxis response regulator protein-glut...	CDD	UniProt	Pfam	InterPro	
**REC-PAS**							
Mpsy_0813	AFV23022	putative signal transduction protein [Meth...	CDD	UniProt	Pfam	InterPro	
Mpsy_2721	AFV24922	multisensor signal transduction histidine ...	CDD	UniProt	Pfam	InterPro	
**HTH-REC**							
Mpsy_1252	AFV23460	response regulator receiver protein [Metha...	CDD	UniProt	Pfam	InterPro	
**REC-MetOD1**							
Mpsy_2767	AFV24968	response regulator receiver [Methanolobus ...	CDD	UniProt	Pfam	InterPro	(REC-MetOD1)
**Other RRs**							
Mpsy_2343	AFV24547	response regulator receiver protein [Metha...	CDD	UniProt	Pfam	InterPro	(KaiC-REC)
Mpsy_2611	AFV24814	hypothetical protein Mpsy_2611 [Methanolob...	CDD	UniProt	Pfam	InterPro	(REC-PAS-PAS-DUF835)

Methanomethylovorans hollandica DSM 15978

HisK, no REC

Metho_0017	AGB48315	PAS domain S-box [Methanomethylovorans hol...	CDD	UniProt	Pfam	InterPro
Metho_0348	AGB48621	PAS domain S-box [Methanomethylovorans hol...	CDD	UniProt	Pfam	InterPro
Metho_0372	AGB48645	PAS domain S-box [Methanomethylovorans hol...	CDD	UniProt	Pfam	InterPro
Metho_0391	AGB48661	histidine kinase [Methanomethylovorans hol...	CDD	UniProt	Pfam	InterPro
Metho_1007	AGB49246	PAS domain S-box [Methanomethylovorans hol...	CDD	UniProt	Pfam	InterPro
Metho_1008	AGB49247	PAS domain S-box [Methanomethylovorans hol...	CDD	UniProt	Pfam	InterPro
Metho_1028	AGB49266	signal transduction histidine kinase [Meth...	CDD	UniProt	Pfam	InterPro
Metho_1103	AGB49338	PAS domain S-box [Methanomethylovorans hol...	CDD	UniProt	Pfam	InterPro
Metho_1265	AGB49493	PAS domain S-box [Methanomethylovorans hol...	CDD	UniProt	Pfam	InterPro
Metho_1298	AGB49521	PAS domain S-box [Methanomethylovorans hol...	CDD	UniProt	Pfam	InterPro
Metho_1431	AGB49638	chemotaxis protein histidine kinase-like p...	CDD	UniProt	Pfam	InterPro

REC only

Metho_0018	AGB48316	CheY-like receiver domain-containing prote...	CDD	UniProt	Pfam	InterPro
Metho_1029	AGB49267	CheY-like receiver domain-containing prote...	CDD	UniProt	Pfam	InterPro
Metho_1347	AGB49565	response regulator containing a CheY-like ...	CDD	UniProt	Pfam	InterPro
Metho_1429	AGB49636	chemotaxis response regulator containing a...	CDD	UniProt	Pfam	InterPro

REC-HisK

Metho_0316	AGB48590	PAS domain S-box [Methanomethylovorans hol...	CDD	UniProt	Pfam	InterPro
Metho_1090	AGB49325	PAS domain S-box [Methanomethylovorans hol...	CDD	UniProt	Pfam	InterPro

REC-CheB

Metho_1430	AGB49637	chemotaxis response regulator protein-glut...	CDD	UniProt	Pfam	InterPro

REC-PAS

Metho_1877	AGB50054	PAS domain S-box [Methanomethylovorans hol...	CDD	UniProt	Pfam	InterPro

Other RRs

Metho_0471	AGB48738	response regulator with CheY-like receiver...	CDD	UniProt	Pfam	InterPro	(KaiC-REC)

Methanoplanus limicola DSM 2279

HisK, no REC

Metlim_0040	EHQ34193	PAS/PAC sensor signal transduction histid...	CDD	UniProt	Pfam	InterPro
Metlim_0344	EHQ34485	PAS/PAC sensor signal transduction histid...	CDD	UniProt	Pfam	InterPro
Metlim_0721	EHQ34844	PAS/PAC sensor signal transduction histid...	CDD	UniProt	Pfam	InterPro
Metlim_0814	EHQ34936	multi-sensor signal transduction histidin...	CDD	UniProt	Pfam	InterPro
Metlim_0817	EHQ34939	PAS/PAC sensor signal transduction histid...	CDD	UniProt	Pfam	InterPro
Metlim_0899	EHQ35021	multi-sensor signal transduction histidin...	CDD	UniProt	Pfam	InterPro
Metlim_0909	EHQ35031	PAS/PAC sensor signal transduction histid...	CDD	UniProt	Pfam	InterPro
Metlim_0911	EHQ35033	histidine kinase [Methanoplanus limicola ...	CDD	UniProt	Pfam	InterPro
Metlim_0938	EHQ35060	histidine kinase [Methanoplanus limicola ...	CDD	UniProt	Pfam	InterPro
Metlim_1000	EHQ35122	PAS/PAC sensor signal transduction histid...	CDD	UniProt	Pfam	InterPro
Metlim_1148	EHQ35259	signal transduction histidine kinase [Met...	CDD	UniProt	Pfam	InterPro
Metlim_1168	EHQ35279	PAS/PAC sensor signal transduction histid...	CDD	UniProt	Pfam	InterPro
Metlim_1222	EHQ35332	integral membrane sensor signal transduct...	CDD	UniProt	Pfam	InterPro
Metlim_1317	EHQ35426	signal transduction histidine kinase [Met...	CDD	UniProt	Pfam	InterPro
Metlim_1319	EHQ35428	signal transduction histidine kinase [Met...	CDD	UniProt	Pfam	InterPro
Metlim_1478	EHQ35581	multi-sensor signal transduction histidin...	CDD	UniProt	Pfam	InterPro
Metlim_1502	EHQ35605	multi-sensor signal transduction histidin...	CDD	UniProt	Pfam	InterPro
Metlim_1519	EHQ35620	multi-sensor signal transduction histidin...	CDD	UniProt	Pfam	InterPro
Metlim_1710	EHQ35811	multi-sensor signal transduction histidin...	CDD	UniProt	Pfam	InterPro
Metlim_1992	EHQ36081	PAS/PAC sensor signal transduction histid...	CDD	UniProt	Pfam	InterPro
Metlim_2014	EHQ36100	multi-sensor signal transduction histidin...	CDD	UniProt	Pfam	InterPro
Metlim_2147	EHQ36222	ATP-binding region ATPase domain protein ...	CDD	UniProt	Pfam	InterPro
Metlim_2193	EHQ36258	integral membrane sensor signal transduct...	CDD	UniProt	Pfam	InterPro
Metlim_2233	EHQ36294	multi-sensor signal transduction histidin...	CDD	UniProt	Pfam	InterPro
Metlim_2479	EHQ36526	multi-sensor signal transduction histidin...	CDD	UniProt	Pfam	InterPro
Metlim_2630	EHQ36671	CheA signal transduction histidine kinase...	CDD	UniProt	Pfam	InterPro
Metlim_2717	EHQ36755	signal transduction histidine kinase [Met...	CDD	UniProt	Pfam	InterPro
Metlim_2961	EHQ36994	PAS/PAC sensor signal transduction histid...	CDD	UniProt	Pfam	InterPro
Metlim_2977	EHQ37009	signal transduction histidine kinase [Met...	CDD	UniProt	Pfam	InterPro
Metlim_3018	EHQ37050	integral membrane sensor signal transduct...	CDD	UniProt	Pfam	InterPro
Metlim_3059	EHQ37091	integral membrane sensor signal transduct...	CDD	UniProt	Pfam	InterPro

Hybrid HisK

Metlim_2017	EHQ36103	CheA signal transduction histidine kinase...	CDD	UniProt	Pfam	InterPro

HisKA, no HATPase

Metlim_0048	EHQ34201	PAS sensor protein [Methanoplanus limicol...	CDD	UniProt	Pfam	InterPro
Metlim_0685	EHQ34808	PAS sensor protein [Methanoplanus limicol...	CDD	UniProt	Pfam	InterPro
Metlim_1284	EHQ35393	PAS sensor protein [Methanoplanus limicol...	CDD	UniProt	Pfam	InterPro
Metlim_1482	EHQ35585	PAS sensor protein [Methanoplanus limicol...	CDD	UniProt	Pfam	InterPro
Metlim_1987	EHQ36076	putative PAS/PAC sensor protein [Methanop...	CDD	UniProt	Pfam	InterPro

REC only

Metlim_0900	EHQ35022	response regulator receiver protein [Metha...	CDD	UniProt	Pfam	InterPro
Metlim_0907	EHQ35029	response regulator receiver protein [Metha...	CDD	UniProt	Pfam	InterPro
Metlim_1521	EHQ35622	response regulator receiver protein [Metha...	CDD	UniProt	Pfam	InterPro
Metlim_2232	EHQ36293	response regulator receiver protein [Metha...	CDD	UniProt	Pfam	InterPro
Metlim_2583	EHQ36625	response regulator receiver protein [Metha...	CDD	UniProt	Pfam	InterPro
Metlim_2632	EHQ36673	response regulator receiver protein [Metha...	CDD	UniProt	Pfam	InterPro

REC-HisK
Metlim_0842 EHQ34964 putative PAS/PAC sensor protein [Methanopl... CDD UniProt Pfam InterPro
Metlim_1139 EHQ35250 signal transduction histidine kinase [Meth... CDD UniProt Pfam InterPro
Metlim_1204 EHQ35314 multi-sensor signal transduction histidine... CDD UniProt Pfam InterPro
Metlim_1473 EHQ35576 multi-sensor signal transduction histidin... CDD UniProt Pfam InterPro
Metlim_1781 EHQ35882 multi-sensor signal transduction histidin... CDD UniProt Pfam InterPro
Metlim_1993 EHQ36082 multi-sensor signal transduction histidin... CDD UniProt Pfam InterPro
Metlim_2015 EHQ36101 response regulator receiver sensor signal ... CDD UniProt Pfam InterPro
Metlim_2038 EHQ36123 multi-sensor signal transduction histidine... CDD UniProt Pfam InterPro
Metlim_2077 EHQ36162 multi-sensor signal transduction histidine... CDD UniProt Pfam InterPro
Metlim_2136 EHQ36211 multi-sensor signal transduction histidin... CDD UniProt Pfam InterPro
Metlim_2137 EHQ36212 multi-sensor signal transduction histidin... CDD UniProt Pfam InterPro
Metlim_2140 EHQ36215 multi-sensor signal transduction histidine... CDD UniProt Pfam InterPro
Metlim_2141 EHQ36216 putative PAS/PAC sensor protein [Methanopl... CDD UniProt Pfam InterPro
Metlim_2142 EHQ36217 multi-sensor signal transduction histidine... CDD UniProt Pfam InterPro
Metlim_2143 EHQ36218 putative PAS/PAC sensor protein [Methanopl... CDD UniProt Pfam InterPro
Metlim_2186 EHQ36254 response regulator receiver sensor signal ... CDD UniProt Pfam InterPro
Metlim_2231 EHQ36292 putative PAS/PAC sensor protein [Methanop... CDD UniProt Pfam InterPro
Metlim_2375 EHQ36426 multi-sensor signal transduction histidine... CDD UniProt Pfam InterPro
REC-CheB
Metlim_2631 EHQ36672 response regulator receiver protein [Metha... CDD UniProt Pfam InterPro
REC-PAS/GAF
Metlim_0533 EHQ34668 CheA signal transduction histidine kinase ... CDD UniProt Pfam InterPro
Metlim_0794 EHQ34917 putative PAS/PAC sensor protein [Methanopl... CDD UniProt Pfam InterPro
Metlim_1147 EHQ35258 putative PAS/PAC sensor protein [Methanopl... CDD UniProt Pfam InterPro
Metlim_1285 EHQ35394 putative PAS/PAC sensor protein [Methanopl... CDD UniProt Pfam InterPro
Metlim_2716 EHQ36754 response regulator receiver protein [Metha... CDD UniProt Pfam InterPro
Metlim_2495 EHQ36542 response regulator receiver modulated GAF ... CDD UniProt Pfam InterPro
Other RRs
Metlim_0426 EHQ34565 putative PAS/PAC sensor protein [Methanopl... CDD UniProt Pfam InterPro (REC-PAS-PAS-HisKA)
Metlim_1761 EHQ35862 response regulator receiver protein [Metha... CDD UniProt Pfam InterPro (REC-MetOD2)
Metlim_2029 EHQ36115 response regulator receiver protein [Metha... CDD UniProt Pfam InterPro (REC-MetOD2)
Metlim_2144 EHQ36219 response regulator receiver protein [Metha... CDD UniProt Pfam InterPro (REC-MetOD2)
Metlim_2230 EHQ36291 response regulator receiver protein [Metha... CDD UniProt Pfam InterPro (no archaeal hits)
Metlim_2231 EHQ36292 putative PAS/PAC sensor protein [Methanopl... CDD UniProt Pfam InterPro (REC-PAS-HisKA)
Metlim_3023 EHQ37055 putative PAS/PAC sensor protein [Methanopl... CDD UniProt Pfam InterPro (REC-PAS-xHisKA)
Metlim_3060 EHQ37092 response regulator receiver protein [Metha... CDD UniProt Pfam InterPro (REC-PAS-wHTH-wHTH-DUF1724)

Methanoregula boonei 6A8
HisK, no REC
Mboo_0295 ABS54817 PAS/PAC sensor signal transduction histidi... CDD UniProt Pfam InterPro
Mboo_0328 ABS54850 putative PAS/PAC sensor protein [Methanore... CDD UniProt Pfam InterPro
Mboo_0467 ABS54985 multi-sensor signal transduction histidine... CDD UniProt Pfam InterPro
Mboo_0782 ABS55300 multi-sensor signal transduction histidine... CDD UniProt Pfam InterPro
Mboo_0821 ABS55339 putative PAS/PAC sensor protein [Methanore... CDD UniProt Pfam InterPro
Mboo_0935 ABS55453 histidine kinase [Methanoregula boonei 6A8... CDD UniProt Pfam InterPro
Mboo_0936 ABS55454 signal transduction histidine kinase [Meth... CDD UniProt Pfam InterPro
Mboo_0999 ABS55517 multi-sensor signal transduction histidine... CDD UniProt Pfam InterPro
Mboo_1008 ABS55526 signal transduction histidine kinase [Meth... CDD UniProt Pfam InterPro
Mboo_1028 ABS55546 integral membrane sensor signal transducti... CDD UniProt Pfam InterPro
Mboo_1104 ABS55622 multi-sensor signal transduction histidine... CDD UniProt Pfam InterPro
Mboo_1112 ABS55630 multi-sensor signal transduction histidine... CDD UniProt Pfam InterPro
Mboo_1119 ABS55637 multi-sensor signal transduction histidine... CDD UniProt Pfam InterPro
Mboo_1140 ABS55658 multi-sensor signal transduction histidine... CDD UniProt Pfam InterPro
Mboo_1231 ABS55749 integral membrane sensor signal transducti... CDD UniProt Pfam InterPro
Mboo_1244 ABS55762 PAS/PAC sensor signal transduction histidi... CDD UniProt Pfam InterPro
Mboo_1250 ABS55768 PAS/PAC sensor signal transduction histidi... CDD UniProt Pfam InterPro
Mboo_1336 ABS55854 CheA signal transduction histidine kinase ... CDD UniProt Pfam InterPro
Mboo_1359 ABS55877 signal transduction histidine kinase [Meth... CDD UniProt Pfam InterPro
Mboo_1487 ABS56005 signal transduction histidine kinase [Meth... CDD UniProt Pfam InterPro
Mboo_1496 ABS56014 signal transduction histidine kinase [Meth... CDD UniProt Pfam InterPro
Mboo_1516 ABS56034 signal transduction histidine kinase [Meth... CDD UniProt Pfam InterPro
Mboo_1527 ABS56045 signal transduction histidine kinase [Meth... CDD UniProt Pfam InterPro
Mboo_1806 ABS56322 signal transduction histidine kinase [Meth... CDD UniProt Pfam InterPro
Mboo_1810 ABS56326 PAS/PAC sensor signal transduction histidi... CDD UniProt Pfam InterPro
Mboo_1979 ABS56493 multi-sensor signal transduction histidine... CDD UniProt Pfam InterPro
Mboo_2061 ABS56575 signal transduction histidine kinase regul... CDD UniProt Pfam InterPro
Mboo_2200 ABS56714 histidine kinase [Methanoregula boonei 6A8... CDD UniProt Pfam InterPro
Mboo_2408 ABS56922 signal transduction histidine kinase [Meth... CDD UniProt Pfam InterPro
Mboo_2409 ABS56923 integral membrane sensor signal transducti... CDD UniProt Pfam InterPro
HisKA, no HATPase
Mboo_1107 ABS55625 putative PAS/PAC sensor protein [Methanore... CDD UniProt Pfam InterPro
REC only
Mboo_0332 ABS54853 response regulator receiver protein [Metha... CDD UniProt Pfam InterPro
Mboo_0900 ABS55418 response regulator receiver protein [Metha... CDD UniProt Pfam InterPro

Mboo_0913	ABS55431	response regulator receiver protein [Metha...	CDD	UniProt	Pfam	InterPro	
Mboo_1023	ABS55541	response regulator receiver protein [Metha...	CDD	UniProt	Pfam	InterPro	
Mboo_1102	ABS55620	response regulator receiver protein [Metha...	CDD	UniProt	Pfam	InterPro	
Mboo_1120	ABS55638	response regulator receiver protein [Metha...	CDD	UniProt	Pfam	InterPro	
Mboo_1246	ABS55764	response regulator receiver protein [Metha...	CDD	UniProt	Pfam	InterPro	
Mboo_1338	ABS55856	response regulator receiver protein [Metha...	CDD	UniProt	Pfam	InterPro	
Mboo_1517	ABS56035	response regulator receiver protein [Metha...	CDD	UniProt	Pfam	InterPro	
Mboo_1697	ABS56214	response regulator receiver protein [Metha...	CDD	UniProt	Pfam	InterPro	
Mboo_1698	ABS56215	response regulator receiver protein [Metha...	CDD	UniProt	Pfam	InterPro	
Mboo_1811	ABS56327	response regulator receiver protein [Metha...	CDD	UniProt	Pfam	InterPro	
Mboo_1814	ABS56330	response regulator receiver protein [Metha...	CDD	UniProt	Pfam	InterPro	
Mboo_2092	ABS56606	response regulator receiver protein [Metha...	CDD	UniProt	Pfam	InterPro	
Mboo_2407	ABS56921	response regulator receiver protein [Metha...	CDD	UniProt	Pfam	InterPro	

FrzZ family (REC-REC)

Mboo_2154	ABS56668	response regulator receiver protein [Metha...	CDD	UniProt	Pfam	InterPro	

REC-HisK

Mboo_0161	ABS54684	multi-sensor signal transduction histidine...	CDD	UniProt	Pfam	InterPro	
Mboo_0274	ABS54796	multi-sensor signal transduction histidine...	CDD	UniProt	Pfam	InterPro	
Mboo_0292	ABS54814	multi-sensor signal transduction histidine...	CDD	UniProt	Pfam	InterPro	
Mboo_0309	ABS54831	multi-sensor signal transduction histidine...	CDD	UniProt	Pfam	InterPro	
Mboo_0331	ABS54852	multi-sensor signal transduction histidine...	CDD	UniProt	Pfam	InterPro	
Mboo_0338	ABS54859	multi-sensor signal transduction histidine...	CDD	UniProt	Pfam	InterPro	
Mboo_0339	ABS54860	multi-sensor signal transduction histidine...	CDD	UniProt	Pfam	InterPro	
Mboo_0402	ABS54922	multi-sensor signal transduction histidine...	CDD	UniProt	Pfam	InterPro	
Mboo_0459	ABS54977	multi-sensor signal transduction histidine...	CDD	UniProt	Pfam	InterPro	
Mboo_0640	ABS55158	multi-sensor signal transduction histidine...	CDD	UniProt	Pfam	InterPro	
Mboo_1121	ABS55639	multi-sensor signal transduction histidine...	CDD	UniProt	Pfam	InterPro	
Mboo_1242	ABS55760	multi-sensor signal transduction histidine...	CDD	UniProt	Pfam	InterPro	
Mboo_1360	ABS55878	signal transduction histidine kinase [Meth...	CDD	UniProt	Pfam	InterPro	
Mboo_1463	ABS55981	multi-sensor signal transduction histidine...	CDD	UniProt	Pfam	InterPro	
Mboo_1464	ABS55982	multi-sensor signal transduction histidine...	CDD	UniProt	Pfam	InterPro	
Mboo_1482	ABS56000	multi-sensor signal transduction histidine...	CDD	UniProt	Pfam	InterPro	
Mboo_1502	ABS56020	multi-sensor signal transduction histidine...	CDD	UniProt	Pfam	InterPro	
Mboo_1520	ABS56038	multi-sensor signal transduction histidine...	CDD	UniProt	Pfam	InterPro	
Mboo_1812	ABS56328	signal transduction histidine kinase [Meth...	CDD	UniProt	Pfam	InterPro	
Mboo_2107	ABS56621	multi-sensor signal transduction histidine...	CDD	UniProt	Pfam	InterPro	
Mboo_2110	ABS56624	multi-sensor signal transduction histidine...	CDD	UniProt	Pfam	InterPro	
Mboo_2366	ABS56880	multi-sensor signal transduction histidine...	CDD	UniProt	Pfam	InterPro	

REC-CheB

Mboo_1337	ABS55855	response regulator receiver protein [Metha...	CDD	UniProt	Pfam	InterPro	

REC-PAS/GAF

Mboo_1007	ABS55525	putative GAF sensor protein [Methanoregula...	CDD	UniProt	Pfam	InterPro	
Mboo_1497	ABS56015	putative PAS/PAC sensor protein [Methanore...	CDD	UniProt	Pfam	InterPro	
Mboo_1807	ABS56323	putative PAS/PAC sensor protein [Methanore...	CDD	UniProt	Pfam	InterPro	
Mboo_1808	ABS56324	putative PAS/PAC sensor protein [Methanore...	CDD	UniProt	Pfam	InterPro	
Mboo_1813	ABS56329	response regulator receiver protein [Metha...	CDD	UniProt	Pfam	InterPro	
Mboo_1912	ABS56427	response regulator receiver protein [Metha...	CDD	UniProt	Pfam	InterPro	

Other RRs

Mboo_0795	ABS55313	putative PAS/PAC sensor protein [Methanore...	CDD	UniProt	Pfam	InterPro	(REC-PAS-TPR)
Mboo_0887	ABS55405	response regulator receiver protein [Metha...	CDD	UniProt	Pfam	InterPro	(REC-Smc)
Mboo_0965	ABS55483	response regulator receiver protein [Metha...	CDD	UniProt	Pfam	InterPro	(MetOD3-REC-MetOD2)
Mboo_1245	ABS55763	putative PAS/PAC sensor protein [Methanore...	CDD	UniProt	Pfam	InterPro	(REC-PAS-GAF)
Mboo_1311	ABS55829	putative PAS/PAC sensor protein [Methanore...	CDD	UniProt	Pfam	InterPro	(REC-PAS-xHisKA)
Mboo_1358	ABS55876	putative PAS/PAC sensor protein [Methanore...	CDD	UniProt	Pfam	InterPro	(REC-PAS-TPR)
Mboo_1478	ABS55996	response regulator receiver protein [Metha...	CDD	UniProt	Pfam	InterPro	(MetOD3-REC-MetOD2)
Mboo_1488	ABS56006	response regulator receiver protein [Metha...	CDD	UniProt	Pfam	InterPro	(REC-xxx-HTH/PadR)
Mboo_1528	ABS56046	response regulator receiver protein [Metha...	CDD	UniProt	Pfam	InterPro	(REC-xxx-HTH/PadR)
Mboo_1609	ABS56126	response regulator receiver protein [Metha...	CDD	UniProt	Pfam	InterPro	(MetOD5-REC)
Mboo_1616	ABS56133	putative PAS/PAC sensor protein [Methanore...	CDD	UniProt	Pfam	InterPro	(REC-PAS-PAS-DisA)
Mboo_1936	ABS56451	response regulator receiver protein [Metha...	CDD	UniProt	Pfam	InterPro	(REC-MetOD2)
Mboo_1937	ABS56452	response regulator receiver protein [Metha...	CDD	UniProt	Pfam	InterPro	(REC-MetOD2)
Mboo_1949	ABS56464	putative PAS/PAC sensor protein [Methanorg...	CDD	UniProt	Pfam	InterPro	(REC-PAS-TPR)
Mboo_2032	ABS56546	response regulator receiver protein [Metha...	CDD	UniProt	Pfam	InterPro	(REC-TPR)
Mboo_2267	ABS56781	putative PAS/PAC sensor protein [Methanore...	CDD	UniProt	Pfam	InterPro	(REC-PAS-PAS-xxx)
Mboo_2336	ABS56850	response regulator receiver protein [Metha...	CDD	UniProt	Pfam	InterPro	(xxx_REC)
Mboo_2365	ABS56879	response regulator receiver protein [Metha...	CDD	UniProt	Pfam	InterPro	(REC-MetOD2)

Methanoregula formicica SMSP

HisK, no REC

Metfor_0128	AGB01213	PAS domain S-box [Methanoregula formicica...	CDD	UniProt	Pfam	InterPro
Metfor_0165	AGB01247	histidine kinase [Methanoregula formicica...	CDD	UniProt	Pfam	InterPro
Metfor_0385	AGB01459	PAS domain S-box [Methanoregula formicica...	CDD	UniProt	Pfam	InterPro
Metfor_0407	AGB01480	PAS domain S-box [Methanoregula formicica...	CDD	UniProt	Pfam	InterPro

Metfor_0409	AGB01482	periplasmic component of amino acid ABC-t...	CDD	UniProt	Pfam	InterPro	
Metfor_0465	AGB01537	PAS domain S-box [Methanoregula formicica...	CDD	UniProt	Pfam	InterPro	
Metfor_0520	AGB01590	histidine kinase [Methanoregula formicica...	CDD	UniProt	Pfam	InterPro	
Metfor_0557	AGB01620	PAS domain S-box [Methanoregula formicica...	CDD	UniProt	Pfam	InterPro	
Metfor_1106	AGB02154	bacteriophytochrome (light-regulated sign...	CDD	UniProt	Pfam	InterPro	
Metfor_1181	AGB02225	PAS domain S-box [Methanoregula formicica...	CDD	UniProt	Pfam	InterPro	
Metfor_1257	AGB02300	PAS domain S-box [Methanoregula formicica...	CDD	UniProt	Pfam	InterPro	
Metfor_1519	AGB02552	chemotaxis protein histidine kinase-like ...	CDD	UniProt	Pfam	InterPro	
Metfor_1687	AGB02714	bacteriophytochrome (light-regulated sign...	CDD	UniProt	Pfam	InterPro	
Metfor_1755	AGB02779	PAS domain S-box [Methanoregula formicica...	CDD	UniProt	Pfam	InterPro	
Metfor_1887	AGB02908	PAS domain S-box [Methanoregula formicica...	CDD	UniProt	Pfam	InterPro	
Metfor_1974	AGB02988	PAS domain S-box [Methanoregula formicica...	CDD	UniProt	Pfam	InterPro	
Metfor_2040	AGB03053	PAS domain S-box [Methanoregula formicica...	CDD	UniProt	Pfam	InterPro	
Metfor_2139	AGB03145	PAS domain S-box [Methanoregula formicica...	CDD	UniProt	Pfam	InterPro	
Metfor_2145	AGB03151	PAS domain S-box [Methanoregula formicica...	CDD	UniProt	Pfam	InterPro	
Metfor_2161	AGB03167	PAS domain S-box [Methanoregula formicica...	CDD	UniProt	Pfam	InterPro	
Metfor_2184	AGB03190	PAS domain S-box [Methanoregula formicica...	CDD	UniProt	Pfam	InterPro	
Metfor_2191	AGB03197	PAS domain S-box [Methanoregula formicica...	CDD	UniProt	Pfam	InterPro	
Metfor_2187	AGB03193	PAS domain S-box [Methanoregula formicica...	CDD	UniProt	Pfam	InterPro	
Metfor_2193	AGB03199	PAS domain S-box [Methanoregula formicica...	CDD	UniProt	Pfam	InterPro	
Metfor_2194	AGB03200	PAS domain S-box [Methanoregula formicica...	CDD	UniProt	Pfam	InterPro	
Metfor_2269	AGB03274	PAS domain S-box [Methanoregula formicica...	CDD	UniProt	Pfam	InterPro	
Metfor_2327	AGB03331	signal transduction histidine kinase [Met...	CDD	UniProt	Pfam	InterPro	
Metfor_2801	AGB03784	PAS domain S-box [Methanoregula formicica...	CDD	UniProt	Pfam	InterPro	
Metfor_2804	AGB03787	PAS domain S-box [Methanoregula formicica...	CDD	UniProt	Pfam	InterPro	
*Metfor_0342	AGB01419	histidine kinase,PAS domain-containing pr...	CDD	UniProt	Pfam	InterPro	
**Hybrid HisK**							
Metfor_1885	AGB02906	chemotaxis protein histidine kinase-like ...	CDD	UniProt	Pfam	InterPro	
**REC only**							
Metfor_0758	AGB01818	response regulator with CheY-like receiver...	CDD	UniProt	Pfam	InterPro	
Metfor_1517	AGB02550	response regulator with CheY-like receiver...	CDD	UniProt	Pfam	InterPro	
Metfor_2138	AGB03144	CheY-like receiver domain-containing prote...	CDD	UniProt	Pfam	InterPro	
Metfor_2143	AGB03149	response regulator with CheY-like receiver...	CDD	UniProt	Pfam	InterPro	
Metfor_2328	AGB03332	CheY-like receiver domain-containing prote...	CDD	UniProt	Pfam	InterPro	
**REC-HisK**							
Metfor_0001	AGB01089	PAS domain S-box [Methanoregula formicica ...	CDD	UniProt	Pfam	InterPro	
Metfor_0062	AGB01148	PAS domain S-box [Methanoregula formicica ...	CDD	UniProt	Pfam	InterPro	
Metfor_0164	AGB01246	PAS domain S-box [Methanoregula formicica ...	CDD	UniProt	Pfam	InterPro	
Metfor_0325	AGB01402	PAS domain S-box [Methanoregula formicica ...	CDD	UniProt	Pfam	InterPro	
Metfor_0384	AGB01458	PAS domain S-box [Methanoregula formicica ...	CDD	UniProt	Pfam	InterPro	
Metfor_0534	AGB01604	PAS domain S-box [Methanoregula formicica ...	CDD	UniProt	Pfam	InterPro	
Metfor_0945	AGB02000	PAS domain S-box [Methanoregula formicica ...	CDD	UniProt	Pfam	InterPro	
Metfor_1387	AGB02426	PAS domain S-box [Methanoregula formicica ...	CDD	UniProt	Pfam	InterPro	
Metfor_1886	AGB02907	response regulator containing a CheY-like ...	CDD	UniProt	Pfam	InterPro	
Metfor_1959	AGB02974	PAS domain S-box [Methanoregula formicica ...	CDD	UniProt	Pfam	InterPro	
Metfor_2072	AGB03081	response regulator containing a CheY-like ...	CDD	UniProt	Pfam	InterPro	
Metfor_2186	AGB03192	Response regulator receiver domain protein...	CDD	UniProt	Pfam	InterPro	
Metfor_2228	AGB03233	PAS domain S-box [Methanoregula formicica ...	CDD	UniProt	Pfam	InterPro	
Metfor_2240	AGB03245	PAS domain S-box [Methanoregula formicica ...	CDD	UniProt	Pfam	InterPro	
Metfor_2326	AGB03330	PAS domain S-box [Methanoregula formicica ...	CDD	UniProt	Pfam	InterPro	
Metfor_2329	AGB03333	bacteriophytochrome (light-regulated signa...	CDD	UniProt	Pfam	InterPro	
Metfor_2398	AGB03400	PAS domain S-box [Methanoregula formicica ...	CDD	UniProt	Pfam	InterPro	
Metfor_2747	AGB03732	signal transduction histidine kinase [Meth...	CDD	UniProt	Pfam	InterPro	
Metfor_2844	AGB03827	PAS domain S-box [Methanoregula formicica...	CDD	UniProt	Pfam	InterPro	
Metfor_2917	AGB03897	PAS domain S-box [Methanoregula formicica ...	CDD	UniProt	Pfam	InterPro	
**REC-CheB**							
Metfor_1518	AGB02551	response regulator with CheY-like receiver...	CDD	UniProt	Pfam	InterPro	
**REC-PAS/GAF**							
Metfor_0046	AGB01132	CheY-like receiver domain-containing prote...	CDD	UniProt	Pfam	InterPro	
Metfor_0405	AGB01478	PAS domain S-box [Methanoregula formicica ...	CDD	UniProt	Pfam	InterPro	
Metfor_1064	AGB02113	CheY-like receiver domain-containing prote...	CDD	UniProt	Pfam	InterPro	
Metfor_1100	AGB02148	PAS domain S-box [Methanoregula formicica ...	CDD	UniProt	Pfam	InterPro	
Metfor_1729	AGB02756	PAS domain S-box [Methanoregula formicica ...	CDD	UniProt	Pfam	InterPro	
Metfor_2195	AGB03201	CheY-like receiver domain-containing prote...	CDD	UniProt	Pfam	InterPro	
Metfor_2227	AGB03232	PAS domain S-box [Methanoregula formicica ...	CDD	UniProt	Pfam	InterPro	
Metfor_2717	AGB03702	PAS domain S-box [Methanoregula formicica ...	CDD	UniProt	Pfam	InterPro	
**REC-wHTH**							
Metfor_2800	AGB03783	response regulator with CheY-like receiver...	CDD	UniProt	Pfam	InterPro	
**Other RRs**							
Metfor_1023	AGB02074	response regulator with CheY-like receiver...	CDD	UniProt	Pfam	InterPro	(REC-MetOD2)
Metfor_1024	AGB02075	response regulator containing a CheY-like ...	CDD	UniProt	Pfam	InterPro	(REC-MetOD2)
Metfor_1444	AGB02479	response regulator containing a CheY-like ...	CDD	UniProt	Pfam	InterPro	(MetOD3-REC-MetOD2)
Metfor_1899	AGB02918	response regulator containing a CheY-like ...	CDD	UniProt	Pfam	InterPro	(MetOD5-REC)
Metfor_2262	AGB03267	response regulator with CheY-like receiver...	CDD	UniProt	Pfam	InterPro	(REC-Smc)

Methanosaeta concilii GP6

Hybrid HisK

MCON_0550	AEB67389	two-component hybrid sensor and regulator ...	CDD	UniProt	Pfam	InterPro	
MCON_1408	AEB68077	multi-sensor hybrid histidine kinase [Meth...	CDD	UniProt	Pfam	InterPro	
MCON_1632	AEB68259	signal transduction histidine-protein kina...	CDD	UniProt	Pfam	InterPro	
MCON_1639	AEB68265	multi-sensor hybrid histidine kinase [Meth...	CDD	UniProt	Pfam	InterPro	
MCON_2222	AEB68733	two-component hybrid sensor and regulator ...	CDD	UniProt	Pfam	InterPro	
MCON_2375	AEB68840	two-component hybrid sensor and regulator ...	CDD	UniProt	Pfam	InterPro	
MCON_3111	AEB69416	two-component hybrid sensor and regulator ...	CDD	UniProt	Pfam	InterPro	
MCON_3220	AEB69502	histidine-protein kinase [Methanosaeta con...	CDD	UniProt	Pfam	InterPro	
**REC only**							
MCON_0419	AEB67278	response regulator receiver domain protein...	CDD	UniProt	Pfam	InterPro	
MCON_1876	AEB68464	two-component hybrid sensor and regulator ...	CDD	UniProt	Pfam	InterPro	
MCON_2134	AEB68665	response regulator receiver domain protein...	CDD	UniProt	Pfam	InterPro	
**Other RRs**							
MCON_1506	AEB68159	response regulator domain with PAS sensor,...	CDD	UniProt	Pfam	InterPro	(REC-PAS-GAF-xxx)

Methanosaeta harundinacea 6Ac

**HisK, no REC**							
Mhar_0446	AET63831	Multi-sensor signal transduction histidini...	CDD	UniProt	Pfam	InterPro	
Mhar_1766	AET65124	PAS/PAC sensor signal transduction histid ...	CDD	UniProt	Pfam	InterPro	
**REC only**							
Mhar_0447	AET63832	FilR2, methanogenesis response regulator p...	CDD	UniProt	Pfam	InterPro	
Mhar_1520	AET64882	Response regulator receiver protein [Metha...	CDD	UniProt	Pfam	InterPro	
**REC-HisK**							
Mhar_0935	AET64306	Multi-sensor signal transduction histidine...	CDD	UniProt	Pfam	InterPro	
**REC-GAF-PAS**							
Mhar_2042	AET65398	Multi-sensor signal transduction histidine...	CDD	UniProt	Pfam	InterPro	
**Other RRs**							
Mhar_0445	AET63830	FliR1, transcriptional regulator of metano...	CDD	UniProt	Pfam	InterPro	(wHTH-DUF1724-REC)
Mhar_0169	AET63560	Response regulator domain with PAS sensor ...	CDD	UniProt	Pfam	InterPro	(REC-PAS-xGAF)

Methanosaeta thermophila PT

**HisK, no REC**							
Mthe_0065	ABK13867	PAS/PAC sensor signal transduction histidi...	CDD	UniProt	Pfam	InterPro	
Mthe_0630	ABK14421	multi-sensor signal transduction histidine...	CDD	UniProt	Pfam	InterPro	
Mthe_1493	ABK15265	PAS/PAC sensor signal transduction histidi...	CDD	UniProt	Pfam	InterPro	
Mthe_1517	ABK15289	integral membrane sensor signal transducti...	CDD	UniProt	Pfam	InterPro	
Mthe_1524	ABK15296	integral membrane sensor signal transducti...	CDD	UniProt	Pfam	InterPro	
Mthe_1531	ABK15303	integral membrane sensor signal transducti...	CDD	UniProt	Pfam	InterPro	
**Hybrid HisK**							
Mthe_0384	ABK14177	multi-sensor hybrid histidine kinase [Meth...	CDD	UniProt	Pfam	InterPro	
**REC only**							
Mthe_0064	ABK13866	response regulator receiver protein [Metha...	CDD	UniProt	Pfam	InterPro	
Mthe_0407	ABK14200	response regulator receiver protein [Metha...	CDD	UniProt	Pfam	InterPro	
Mthe_1248	ABK15027	response regulator receiver protein [Metha...	CDD	UniProt	Pfam	InterPro	
**Other RRs**							
Mthe_1109	ABK14892	response regulator receiver protein [Metha...	CDD	UniProt	Pfam	InterPro	(REC-HisKA)

Methanosalsum zhilinae DSM 4017

**HisK, no REC**						
Mzhil_0129	AEH60009	signal transduction histidine kinase [Meth...	CDD	UniProt	Pfam	InterPro
Mzhil_0133	AEH60013	signal transduction histidine kinase [Meth...	CDD	UniProt	Pfam	InterPro
Mzhil_0360	AEH60236	multi-sensor signal transduction histidine...	CDD	UniProt	Pfam	InterPro
Mzhil_0396	AEH60271	signal transduction histidine kinase [Meth...	CDD	UniProt	Pfam	InterPro
Mzhil_0572	AEH60439	signal transduction histidine kinase [Meth...	CDD	UniProt	Pfam	InterPro
Mzhil_0577	AEH60444	CheA signal transduction histidine kinase ...	CDD	UniProt	Pfam	InterPro
Mzhil_0608	AEH60475	signal transduction histidine kinase [Meth...	CDD	UniProt	Pfam	InterPro
Mzhil_0638	AEH60505	signal transduction histidine kinase [Meth...	CDD	UniProt	Pfam	InterPro
Mzhil_0670	AEH60537	multi-sensor signal transduction histidine...	CDD	UniProt	Pfam	InterPro
Mzhil_0839	AEH60701	signal transduction histidine kinase [Meth...	CDD	UniProt	Pfam	InterPro
Mzhil_0850	AEH60712	PAS/PAC sensor signal transduction histidi...	CDD	UniProt	Pfam	InterPro
Mzhil_0892	AEH60754	PAS/PAC sensor signal transduction histidi...	CDD	UniProt	Pfam	InterPro
Mzhil_0893	AEH60755	PAS/PAC sensor signal transduction histidi...	CDD	UniProt	Pfam	InterPro
Mzhil_0910	AEH60772	signal transduction histidine kinase [Meth...	CDD	UniProt	Pfam	InterPro
Mzhil_0965	AEH60823	integral membrane sensor signal transducti...	CDD	UniProt	Pfam	InterPro
Mzhil_1077	AEH60933	signal transduction histidine kinase [Meth...	CDD	UniProt	Pfam	InterPro
Mzhil_1153	AEH61009	signal transduction histidine kinase [Meth...	CDD	UniProt	Pfam	InterPro
Mzhil_1154	AEH61010	signal transduction histidine kinase [Meth...	CDD	UniProt	Pfam	InterPro
Mzhil_1180	AEH61036	multi-sensor signal transduction histidine...	CDD	UniProt	Pfam	InterPro
Mzhil_1319	AEH61169	signal transduction histidine kinase [Meth...	CDD	UniProt	Pfam	InterPro
Mzhil_1449	AEH61289	PAS/PAC sensor signal transduction histidi...	CDD	UniProt	Pfam	InterPro
Mzhil_1529	AEH61368	signal transduction histidine kinase [Meth...	CDD	UniProt	Pfam	InterPro
Mzhil_1552	AEH61391	signal transduction histidine kinase [Meth...	CDD	UniProt	Pfam	InterPro
Mzhil_1587	AEH61425	signal transduction histidine kinase [Meth...	CDD	UniProt	Pfam	InterPro
Mzhil_1625	AEH61461	integral membrane sensor signal transducti...	CDD	UniProt	Pfam	InterPro
Mzhil_1694	AEH61529	signal transduction histidine kinase [Meth...	CDD	UniProt	Pfam	InterPro

Mzhil_1793	AEH61628	PAS/PAC sensor signal transduction histidi...	CDD	UniProt	Pfam	InterPro	
Mzhil_1800	AEH61635	PAS/PAC sensor signal transduction histidi...	CDD	UniProt	Pfam	InterPro	
**REC only**							
Mzhil_0575	AEH60442	response regulator receiver protein [Metha...	CDD	UniProt	Pfam	InterPro	
Mzhil_0606	AEH60473	response regulator receiver protein [Metha...	CDD	UniProt	Pfam	InterPro	
Mzhil_0607	AEH60474	response regulator receiver protein [Metha...	CDD	UniProt	Pfam	InterPro	
Mzhil_1530	AEH61369	response regulator receiver protein [Metha...	CDD	UniProt	Pfam	InterPro	
Mzhil_1670	AEH61505	response regulator receiver protein [Metha...	CDD	UniProt	Pfam	InterPro	
**REC-CheB**							
Mzhil_0576	AEH60443	response regulator receiver modulated CheB...	CDD	UniProt	Pfam	InterPro	
**REC-PAS**							
Mzhil_0343	AEH60219	putative PAS/PAC sensor protein [Methanosa...	CDD	UniProt	Pfam	InterPro	
Mzhil_0637	AEH60504	putative PAS/PAC sensor protein [Methanosa...	CDD	UniProt	Pfam	InterPro	
**Other RRs**							
Mzhil_1671	AEH61506	response regulator receiver protein [Metha...	CDD	UniProt	Pfam	InterPro	(KaiC-REC)

Methanosarcina acetivorans C2A

**HisK, no REC**						
MA_0014	AAM03468	chemotaxis sensor histidine kinase [Methan...	CDD	UniProt	Pfam	InterPro
MA_0203	AAM03656	signal-transducing histidine kinase [Metha...	CDD	UniProt	Pfam	InterPro
MA_0490	AAM03934	sensory transduction histidine kinase [Met...	CDD	UniProt	Pfam	InterPro
MA_0551	AAM03995	sensory transduction histidine kinase [Met...	CDD	UniProt	Pfam	InterPro
MA_0552	AAM03996	sensory transduction histidine kinase [Met...	CDD	UniProt	Pfam	InterPro
MA_0619	AAM04062	sensory transduction histidine kinase [Met...	CDD	UniProt	Pfam	InterPro
MA_0620	AAM04063	sensory transduction histidine kinase [Met...	CDD	UniProt	Pfam	InterPro
MA_0758	AAM04198	sensory transduction histidine kinase [Met...	CDD	UniProt	Pfam	InterPro
MA_0759	AAM04199	sensory transduction histidine kinase [Met...	CDD	UniProt	Pfam	InterPro
MA_0777	AAM04216	signal-transducing histidine kinase [Metha...	CDD	UniProt	Pfam	InterPro
MA_0863	AAM04302	sensory transduction histidine kinase [Met...	CDD	UniProt	Pfam	InterPro
MA_0970	AAM04402	sensory transduction histidine kinase [Met...	CDD	UniProt	Pfam	InterPro
MA_1149	AAM04570	sensory transduction histidine kinase [Met...	CDD	UniProt	Pfam	InterPro
MA_1270	AAM04689	sensory transduction histidine kinase [Met...	CDD	UniProt	Pfam	InterPro
MA_1274	AAM04693	sensory transduction histidine kinase [Met...	CDD	UniProt	Pfam	InterPro
MA_1322	AAM04738	sensory transduction histidine kinase [Met...	CDD	UniProt	Pfam	InterPro
MA_1463	AAM04877	sensory transduction histidine kinase [Met...	CDD	UniProt	Pfam	InterPro
MA_1470	AAM04884	sensory transduction histidine kinase [Met...	CDD	UniProt	Pfam	InterPro
MA_1627	AAM05037	sensory transduction histidine kinase [Met...	CDD	UniProt	Pfam	InterPro
MA_1628	AAM05038	sensory transduction histidine kinase [Met...	CDD	UniProt	Pfam	InterPro
MA_1630	AAM05040	sensory transduction histidine kinase [Met...	CDD	UniProt	Pfam	InterPro
MA_1645	AAM05053	sensory transduction histidine kinase [Met...	CDD	UniProt	Pfam	InterPro
MA_1646	AAM05054	sensory transduction histidine kinase [Met...	CDD	UniProt	Pfam	InterPro
MA_1704	AAM05111	sensory transduction histidine kinase [Met...	CDD	UniProt	Pfam	InterPro
MA_1739	AAM05146	sensory transduction histidine kinase [Met...	CDD	UniProt	Pfam	InterPro
MA_1844	AAM05249	sensory transduction histidine kinase [Met...	CDD	UniProt	Pfam	InterPro
MA_1878	AAM05283	sensory transduction histidine kinase [Met...	CDD	UniProt	Pfam	InterPro
MA_1957	AAM05360	sensory transduction histidine kinase [Met...	CDD	UniProt	Pfam	InterPro
MA_1991	AAM05394	sensory transduction histidine kinase [Met...	CDD	UniProt	Pfam	InterPro
MA_2082	AAM05482	sensory transduction histidine kinase [Met...	CDD	UniProt	Pfam	InterPro
MA_2266	AAM05659	sensory transduction histidine kinase [Met...	CDD	UniProt	Pfam	InterPro
MA_2294	AAM05687	sensory transduction histidine kinase [Met...	CDD	UniProt	Pfam	InterPro
MA_2348	AAM05738	sensory transduction histidine kinase [Met...	CDD	UniProt	Pfam	InterPro
MA_2553	AAM05935	sensory transduction histidine kinase [Met...	CDD	UniProt	Pfam	InterPro
MA_2555	AAM05937	sensory transduction histidine kinase [Met...	CDD	UniProt	Pfam	InterPro
MA_2732	AAM06111	sensory transduction histidine kinase [Met...	CDD	UniProt	Pfam	InterPro
MA_2757	AAM06135	sensory transduction histidine kinase [Met...	CDD	UniProt	Pfam	InterPro
MA_2784	AAM06162	sensory transduction histidine kinase [Met...	CDD	UniProt	Pfam	InterPro
MA_2890	AAM06267	sensory transduction histidine kinase [Met...	CDD	UniProt	Pfam	InterPro
MA_3066	AAM06439	chemotaxis sensor histidine kinase [Methan...	CDD	UniProt	Pfam	InterPro
MA_3346	AAM06715	sensory transduction histidine kinase [Met...	CDD	UniProt	Pfam	InterPro
MA_3368	AAM06737	sensory transduction histidine kinase [Met...	CDD	UniProt	Pfam	InterPro
MA_3370	AAM06739	sensory transduction histidine kinase [Met...	CDD	UniProt	Pfam	InterPro
MA_3481	AAM06847	hypothetical protein MA_3481 [Methanosarci...	CDD	UniProt	Pfam	InterPro
MA_3543	AAM06905	sensory transduction histidine kinase [Met...	CDD	UniProt	Pfam	InterPro
MA_3962	AAM07313	sensory transduction histidine kinase [Met...	CDD	UniProt	Pfam	InterPro
MA_4026	AAM07375	sensory transduction histidine kinase [Met...	CDD	UniProt	Pfam	InterPro
MA_4561	AAM07900	sensory transduction histidine kinase [Met...	CDD	UniProt	Pfam	InterPro
**Hybrid HisK**						
MA_2013	AAM05416	sensory transduction histidine kinase [Met...	CDD	UniProt	Pfam	InterPro
MA_2256	AAM05650	sensory transduction histidine kinase [Met...	CDD	UniProt	Pfam	InterPro
MA_4377	AAM07719	sensory transduction histidine kinase [Met...	CDD	UniProt	Pfam	InterPro
**REC only**						
MA_0016	AAM03470	chemotaxis response regulator [Methanosarc...	CDD	UniProt	Pfam	InterPro
MA_0018	AAM03472	response regulator receiver [Methanosarcin...	CDD	UniProt	Pfam	InterPro
MA_1268	AAM04687	response regulator receiver [Methanosarcin...	CDD	UniProt	Pfam	InterPro
MA_1269	AAM04688	response regulator receiver [Methanosarcin...	CDD	UniProt	Pfam	InterPro
MA_1366	AAM04782	response regulator receiver [Methanosarcin...	CDD	UniProt	Pfam	InterPro
MA_1468	AAM04882	response regulator receiver [Methanosarcin...	CDD	UniProt	Pfam	InterPro
MA_1469	AAM04883	response regulator receiver [Methanosarcin...	CDD	UniProt	Pfam	InterPro

MA_2012	AAM05415	response regulator receiver [Methanosarcin...	CDD	UniProt	Pfam	InterPro	
MA_2861	AAM06240	response regulator receiver [Methanosarcin...	CDD	UniProt	Pfam	InterPro	
MA_3068	AAM06441	chemotaxis response regulator [Methanosarc...	CDD	UniProt	Pfam	InterPro	
MA_4376	AAM07718	response regulator receiver [Methanosarcin...	CDD	UniProt	Pfam	InterPro	
MA_4671	AAM05649	response regulator receiver [Methanosarcin...	CDD	UniProt	Pfam	InterPro	
**REC-HisK**							
MA_1267	AAM04686	sensory transduction histidine kinase [Met...	CDD	UniProt	Pfam	InterPro	
MA_3405	AAM06772	sensory transduction histidine kinase [Met...	CDD	UniProt	Pfam	InterPro	
**REC-CheB**							
MA_0015	AAM03469	chemotaxis protein-glutamate methylesteras...	CDD	UniProt	Pfam	InterPro	
MA_3067	AAM06440	chemotaxis protein-glutamate methylesteras...	CDD	UniProt	Pfam	InterPro	
**REC-MetOD1**							
MA_2445	AAM05831	response regulator receiver [Methanosarcin...	CDD	UniProt	Pfam	InterPro	(REC-MetOD1)

Methanosarcina barkeri str. fusaro

**HisK, no REC**							
Mbar_A0404	AAZ69387	hypothetical protein Mbar_A0404 [Methanosa...	CDD	UniProt	Pfam	InterPro	
Mbar_A0679	AAZ69658	sensory transduction histidine kinase [Met...	CDD	UniProt	Pfam	InterPro	
Mbar_A0815	AAZ69792	hypothetical protein Mbar_A0815 [Methanosa...	CDD	UniProt	Pfam	InterPro	
Mbar_A0984	AAZ69955	chemotaxis sensor histidine kinase [Methan...	CDD	UniProt	Pfam	InterPro	
Mbar_A1483	AAZ70438	sensory transduction histidine kinase [Met...	CDD	UniProt	Pfam	InterPro	
Mbar_A1535	AAZ70485	sensory transduction histidine kinase [Met...	CDD	UniProt	Pfam	InterPro	
Mbar_A1666	AAZ70610	sensory transduction histidine kinase [Met...	CDD	UniProt	Pfam	InterPro	
Mbar_A1687	AAZ70629	signal-transducing histidine kinase [Metha...	CDD	UniProt	Pfam	InterPro	
Mbar_A1944	AAZ70883	signal-transducing histidine kinase [Metha...	CDD	UniProt	Pfam	InterPro	
Mbar_A2184	AAZ71112	hypothetical protein Mbar_A2184 [Methanosa...	CDD	UniProt	Pfam	InterPro	
Mbar_A2362	AAZ71283	hypothetical protein Mbar_A2362 [Methanosa...	CDD	UniProt	Pfam	InterPro	
Mbar_A2552	AAZ71465	sensory transduction histidine kinase [Met...	CDD	UniProt	Pfam	InterPro	
Mbar_A2594	AAZ71504	hypothetical protein Mbar_A2594 [Methanosa...	CDD	UniProt	Pfam	InterPro	
Mbar_A2680	AAZ71584	sensory transduction histidine kinase [Met...	CDD	UniProt	Pfam	InterPro	
Mbar_A2691	AAZ71595	hypothetical protein Mbar_A2691 [Methanosa...	CDD	UniProt	Pfam	InterPro	
Mbar_A2876	AAZ71776	sensory transduction histidine kinase [Met...	CDD	UniProt	Pfam	InterPro	
Mbar_A2907	AAZ71805	sensory transduction histidine kinase [Met...	CDD	UniProt	Pfam	InterPro	
Mbar_A2935	AAZ71830	sensory transduction histidine kinase [Met...	CDD	UniProt	Pfam	InterPro	
Mbar_A3007	AAZ71901	two component system histidine kinase [Met...	CDD	UniProt	Pfam	InterPro	
Mbar_A3036	AAZ71928	sensory transduction histidine kinase [Met...	CDD	UniProt	Pfam	InterPro	
Mbar_A3037	AAZ71929	hypothetical protein Mbar_A3037 [Methanosa...	CDD	UniProt	Pfam	InterPro	
Mbar_A3250	AAZ72131	sensory transduction histidine kinase [Met...	CDD	UniProt	Pfam	InterPro	
Mbar_A3447	AAZ72320	hypothetical protein Mbar_A3447 [Methanosa...	CDD	UniProt	Pfam	InterPro	
Mbar_A3538	AAZ72407	hypothetical protein Mbar_A3538 [Methanosa...	CDD	UniProt	Pfam	InterPro	
**Hybrid HisK**							
Mbar_A1052	AAZ70020	sensory transduction histidine kinase [Met...	CDD	UniProt	Pfam	InterPro	
**REC only**							
Mbar_A0988	AAZ69958	response regulator receiver [Methanosarcin...	CDD	UniProt	Pfam	InterPro	
Mbar_A0986	AAZ69957	chemotaxis response regulator[Methanosarci...	CDD	UniProt	Pfam	InterPro	
Mbar_A1051	AAZ70019	response regulator receiver [Methanosarcin...	CDD	UniProt	Pfam	InterPro	
Mbar_A2388	AAZ71309	response regulator receiver [Methanosarcin...	CDD	UniProt	Pfam	InterPro	
Mbar_A2510	AAZ71423	transcriptional regulator [Methanosarcina ...	CDD	UniProt	Pfam	InterPro	
Mbar_A3248	AAZ72129	response regulator receiver [Methanosarcin...	CDD	UniProt	Pfam	InterPro	
Mbar_A3321	AAZ72199	chemotaxis protein [Methanosarcina barkeri...	CDD	UniProt	Pfam	InterPro	
Mbar_A3448	AAZ72321	putative transcriptional regulator [Methan...	CDD	UniProt	Pfam	InterPro	
**REC-HisK**							
Mbar_A3247	AAZ72128	sensory transduction histidine kinase [Met...	CDD	UniProt	Pfam	InterPro	
**REC-CheB**							
Mbar_A0985	AAZ69956	chemotaxis protein-glutamate methylesteras...	CDD	UniProt	Pfam	InterPro	
**wHTH-REC**							
Mbar_A2896	AAZ71794	transcriptional regulator [Methanosarcina ...	CDD	UniProt	Pfam	InterPro	(wHTH-REC)
**REC-MetOD1**							
Mbar_A3109	AAZ71996	response regulator receiver [Methanosarcin...	CDD	UniProt	Pfam	InterPro	(REC-MetOD1)

Methanosarcina horonobensis

**HisK, no REC**							
MSHOH_0263	AKB76746	sensory transduction histidine kinase [Met...	CDD	UniProt	Pfam	InterPro	
MSHOH_0448	AKB76931	sensory transduction histidine kinase [Met...	CDD	UniProt	Pfam	InterPro	
MSHOH_0506	AKB76989	sensory transduction histidine kinase [Met...	CDD	UniProt	Pfam	InterPro	
MSHOH_0621	AKB77104	sensory transduction histidine kinase [Met...	CDD	UniProt	Pfam	InterPro	
MSHOH_0624	AKB77107	sensory transduction histidine kinase [Met...	CDD	UniProt	Pfam	InterPro	
MSHOH_0639	AKB77122	sensory transduction histidine kinase [Met...	CDD	UniProt	Pfam	InterPro	
MSHOH_0998	AKB77481	sensory transduction histidine kinase [Met...	CDD	UniProt	Pfam	InterPro	
MSHOH_1036	AKB77519	Sensory transduction histidine kinase [Met...	CDD	UniProt	Pfam	InterPro	
MSHOH_1037	AKB77520	sensory transduction histidine kinase [Met...	CDD	UniProt	Pfam	InterPro	
MSHOH_1041	AKB77524	sensory transduction histidine kinase [Met...	CDD	UniProt	Pfam	InterPro	
MSHOH_1042	AKB77525	sensory transduction histidine kinase [Met...	CDD	UniProt	Pfam	InterPro	
MSHOH_1063	AKB77546	sensory transduction histidine kinase [Met...	CDD	UniProt	Pfam	InterPro	
MSHOH_1129	AKB77612	sensory transduction histidine kinase [Met...	CDD	UniProt	Pfam	InterPro	
MSHOH_1151	AKB77634	Signal transduction histidine kinase CheA ...	CDD	UniProt	Pfam	InterPro	
MSHOH_1339	AKB77822	sensory transduction histidine kinase [Met...	CDD	UniProt	Pfam	InterPro	

MSHOH_1364	AKB77847	sensory transduction histidine kinase [Met...	CDD	UniProt	Pfam	InterPro
MSHOH_1404	AKB77887	Sensory transduction histidine kinase [Met...	CDD	UniProt	Pfam	InterPro
MSHOH_1423	AKB77906	Sensory transduction histidine kinase [Met...	CDD	UniProt	Pfam	InterPro
MSHOH_1428	AKB77911	Sensory transduction histidine kinase [Met...	CDD	UniProt	Pfam	InterPro
MSHOH_1508	AKB77991	hypothetical protein MSHOH_1508 [Methanosa...	CDD	UniProt	Pfam	InterPro
MSHOH_1511	AKB77994	sensory transduction histidine kinase [Met...	CDD	UniProt	Pfam	InterPro
MSHOH_1522	AKB78005	sensory transduction histidine kinase [Met...	CDD	UniProt	Pfam	InterPro
MSHOH_1535	AKB78018	hypothetical protein MSHOH_1535 [Methanosa...	CDD	UniProt	Pfam	InterPro
MSHOH_1544	AKB78027	sensory transduction histidine kinase [Met...	CDD	UniProt	Pfam	InterPro
MSHOH_1630	AKB78113	sensory transduction histidine kinase [Met...	CDD	UniProt	Pfam	InterPro
MSHOH_1742	AKB78225	sensory transduction histidine kinase [Met...	CDD	UniProt	Pfam	InterPro
MSHOH_1743	AKB78226	sensory transduction histidine kinase [Met...	CDD	UniProt	Pfam	InterPro
MSHOH_1768	AKB78251	sensory transduction histidine kinase [Met...	CDD	UniProt	Pfam	InterPro
MSHOH_1801	AKB78284	diguanylate cyclase/phosphodiesterase (GGD...	CDD	UniProt	Pfam	InterPro
MSHOH_2086	AKB78569	Sensory transduction histidine kinase [Met...	CDD	UniProt	Pfam	InterPro
MSHOH_2227	AKB78710	Sensory transduction histidine kinase [Met...	CDD	UniProt	Pfam	InterPro
MSHOH_2295	AKB78778	sensory transduction histidine kinase [Met...	CDD	UniProt	Pfam	InterPro
MSHOH_2307	AKB78790	sensory transduction histidine kinase [Met...	CDD	UniProt	Pfam	InterPro
MSHOH_2332	AKB78815	sensory transduction histidine kinase [Met...	CDD	UniProt	Pfam	InterPro
MSHOH_2340	AKB78823	sensory transduction histidine kinase [Met...	CDD	UniProt	Pfam	InterPro
MSHOH_2356	AKB78839	sensory transduction histidine kinase [Met...	CDD	UniProt	Pfam	InterPro
MSHOH_2462	AKB78945	hypothetical protein MSHOH_2462 [Methanosa...	CDD	UniProt	Pfam	InterPro
MSHOH_2471	AKB78954	sensory transduction histidine kinase [Met...	CDD	UniProt	Pfam	InterPro
MSHOH_2501	AKB78984	sensory transduction histidine kinase [Met...	CDD	UniProt	Pfam	InterPro
MSHOH_2503	AKB78986	hypothetical protein MSHOH_2503 [Methanosa...	CDD	UniProt	Pfam	InterPro
MSHOH_2549	AKB79032	hypothetical protein MSHOH_2549 [Methanosa...	CDD	UniProt	Pfam	InterPro
MSHOH_2593	AKB79076	sensory transduction histidine kinase [Met...	CDD	UniProt	Pfam	InterPro
MSHOH_2603	AKB79086	sensory transduction histidine kinase [Met...	CDD	UniProt	Pfam	InterPro
MSHOH_2678	AKB79161	sensory transduction histidine kinase [Met...	CDD	UniProt	Pfam	InterPro
MSHOH_2695	AKB79178	sensory transduction histidine kinase [Met...	CDD	UniProt	Pfam	InterPro
MSHOH_2696	AKB79179	sensory transduction histidine kinase [Met...	CDD	UniProt	Pfam	InterPro
MSHOH_2747	AKB79230	sensory transduction histidine kinase [Met...	CDD	UniProt	Pfam	InterPro
MSHOH_2788	AKB79271	Sensory transduction histidine kinase [Met...	CDD	UniProt	Pfam	InterPro
MSHOH_2796	AKB79279	sensory transduction histidine kinase [Met...	CDD	UniProt	Pfam	InterPro
MSHOH_2797	AKB79280	sensory transduction histidine kinase [Met...	CDD	UniProt	Pfam	InterPro
MSHOH_3148	AKB79631	Two component system histidine kinase [Met...	CDD	UniProt	Pfam	InterPro
MSHOH_3253	AKB79736	Sensory transduction histidine kinase [Met...	CDD	UniProt	Pfam	InterPro
MSHOH_3361	AKB79844	sensory transduction histidine kinase [Met...	CDD	UniProt	Pfam	InterPro
MSHOH_3437	AKB79920	Signal-transducing histidine kinase [Metha...	CDD	UniProt	Pfam	InterPro
MSHOH_3458	AKB79941	sensory transduction histidine kinase [Met...	CDD	UniProt	Pfam	InterPro
MSHOH_3460	AKB79943	sensory transduction histidine kinase [Met...	CDD	UniProt	Pfam	InterPro
MSHOH_3602	AKB80085	sensory transduction histidine kinase [Met...	CDD	UniProt	Pfam	InterPro
MSHOH_3603	AKB80086	sensory transduction histidine kinase [Met...	CDD	UniProt	Pfam	InterPro
MSHOH_3607	AKB80090	sensory transduction histidine kinase [Met...	CDD	UniProt	Pfam	InterPro
MSHOH_3721	AKB80204	sensory transduction histidine kinase [Met...	CDD	UniProt	Pfam	InterPro
MSHOH_3733	AKB80216	sensory transduction histidine kinase [Met...	CDD	UniProt	Pfam	InterPro
MSHOH_3867	AKB80350	sensory transduction histidine kinase [Met...	CDD	UniProt	Pfam	InterPro
MSHOH_4086	AKB80569	Signal transduction histidine kinase CheA ...	CDD	UniProt	Pfam	InterPro
**Hybrid HisK**						
MSHOH_0295	AKB76778	Sensory transduction histidine kinase [Met...	CDD	UniProt	Pfam	InterPro
MSHOH_1278	AKB77761	hypothetical protein MSHOH_1278 [Methanosa...	CDD	UniProt	Pfam	InterPro
MSHOH_1413	AKB77896	hypothetical protein MSHOH_1413 [Methanosa...	CDD	UniProt	Pfam	InterPro
MSHOH_1756	AKB78239	sensory transduction histidine kinase [Met...	CDD	UniProt	Pfam	InterPro
**REC only**						
MSHOH_0296	AKB76779	Two-component response regulator [Methanos...	CDD	UniProt	Pfam	InterPro
MSHOH_1040	AKB77523	Two-component system response regulator [M...	CDD	UniProt	Pfam	InterPro
MSHOH_1128	AKB77611	Response regulator receiver [Methanosarcin...	CDD	UniProt	Pfam	InterPro
MSHOH_1149	AKB77632	Chemotaxis regulator - transmits chemorece...	CDD	UniProt	Pfam	InterPro
MSHOH_1168	AKB77651	Chemotaxis regulator - transmits chemorece...	CDD	UniProt	Pfam	InterPro
MSHOH_1279	AKB77762	two-component response regulator [Methanos...	CDD	UniProt	Pfam	InterPro
MSHOH_1412	AKB77895	response regulator receiver [Methanosarcin...	CDD	UniProt	Pfam	InterPro
MSHOH_1550	AKB78033	Response regulator receiver [Methanosarcin...	CDD	UniProt	Pfam	InterPro
MSHOH_2228	AKB78711	response regulator receiver [Methanosarcin...	CDD	UniProt	Pfam	InterPro
MSHOH_2229	AKB78712	response regulator receiver [Methanosarcin...	CDD	UniProt	Pfam	InterPro
MSHOH_2339	AKB78822	sensory transduction histidine kinase [Met...	CDD	UniProt	Pfam	InterPro
MSHOH_2341	AKB78824	response regulator receiver [Methanosarcin...	CDD	UniProt	Pfam	InterPro
MSHOH_2795	AKB79278	response regulator receiver [Methanosarcin...	CDD	UniProt	Pfam	InterPro
MSHOH_4082	AKB80565	Two-component response regulator [Methanos...	CDD	UniProt	Pfam	InterPro
MSHOH_4083	AKB80566	Chemotaxis regulator - transmits chemorece...	CDD	UniProt	Pfam	InterPro
MSHOH_1632	AKB78115	Two-component system response regulator [M...	CDD	UniProt	Pfam	InterPro
MSHOH_4084	AKB80567	Chemotaxis response regulator protein-glut...	CDD	UniProt	Pfam	InterPro
*MSHOH_1757	AKB78240	response regulator receiver [Methanosarcin...	CDD	UniProt	Pfam	InterPro
**REC-HisK**						
MSHOH_1039	AKB77522	Sensory transduction histidine kinase [Met...	CDD	UniProt	Pfam	InterPro
MSHOH_1633	AKB78116	sensory transduction histidine kinase [Met...	CDD	UniProt	Pfam	InterPro
MSHOH_2793	AKB79276	hypothetical protein MSHOH_2793 [Methanosa...	CDD	UniProt	Pfam	InterPro
**REC-CheB**						
MSHOH_1150	AKB77633	Chemotaxis response regulator protein-glut...	CDD	UniProt	Pfam	InterPro
**wHTH-REC**						

MSHOH_2970	AKB79453	Phosphate regulon transcriptional regulato...	CDD	UniProt	Pfam	InterPro	
**REC-MetOD1**							
MSHOH_2111	AKB78594	Response regulator receiver [Methanosarcin...	CDD	UniProt	Pfam	InterPro	(REC-MetOD1)

Methanosarcina lacustris

**HisK, no REC**							
MSLAZ_0269	AKB73530	sensory transduction histidine kinase [Met...	CDD	UniProt	Pfam	InterPro	
MSLAZ_0372	AKB73633	Signal-transducing histidine kinase [Metha...	CDD	UniProt	Pfam	InterPro	
MSLAZ_0583	AKB73844	sensory transduction histidine kinase [Met...	CDD	UniProt	Pfam	InterPro	
MSLAZ_0695	AKB73956	Two component system histidine kinase [Met...	CDD	UniProt	Pfam	InterPro	
MSLAZ_0772	AKB74033	sensory transduction histidine kinase [Met...	CDD	UniProt	Pfam	InterPro	
MSLAZ_0907	AKB74168	sensory transduction histidine kinase [Met...	CDD	UniProt	Pfam	InterPro	
MSLAZ_1507	AKB74768	hypothetical protein MSLAZ_1507 [Methanosa...	CDD	UniProt	Pfam	InterPro	
MSLAZ_1509	AKB74770	sensory transduction histidine kinase [Met...	CDD	UniProt	Pfam	InterPro	
MSLAZ_1663	AKB74924	Sensory transduction histidine kinase [Met...	CDD	UniProt	Pfam	InterPro	
MSLAZ_1665	AKB74926	Sensory transduction histidine kinase [Met...	CDD	UniProt	Pfam	InterPro	
MSLAZ_1814	AKB75075	hypothetical protein MSLAZ_1814 [Methanosa...	CDD	UniProt	Pfam	InterPro	
MSLAZ_1912	AKB75173	sensory transduction histidine kinase [Met...	CDD	UniProt	Pfam	InterPro	
MSLAZ_1992	AKB75253	hypothetical protein MSLAZ_1992 [Methanosa...	CDD	UniProt	Pfam	InterPro	
MSLAZ_2115	AKB75376	Sensory transduction histidine kinase [Met...	CDD	UniProt	Pfam	InterPro	
MSLAZ_2313	AKB75574	sensory transduction histidine kinase [Met...	CDD	UniProt	Pfam	InterPro	
MSLAZ_2602	AKB75863	sensory transduction histidine kinase [Met...	CDD	UniProt	Pfam	InterPro	
MSLAZ_3096	AKB76357	Signal transduction histidine kinase CheA ...	CDD	UniProt	Pfam	InterPro	
**Hybrid HisK**							
MSLAZ_2967	AKB76228	Sensory transduction histidine kinase [Met...	CDD	UniProt	Pfam	InterPro	
**REC only**							
MSLAZ_0906	AKB74167	response regulator receiver [Methanosarcin...	CDD	UniProt	Pfam	InterPro	
MSLAZ_1661	AKB74922	response regulator receiver [Methanosarcin...	CDD	UniProt	Pfam	InterPro	
MSLAZ_1662	AKB74923	response regulator receiver [Methanosarcin...	CDD	UniProt	Pfam	InterPro	
MSLAZ_1890	AKB75151	Response regulator receiver [Methanosarcin...	CDD	UniProt	Pfam	InterPro	
MSLAZ_2968	AKB76229	Two-component response regulator [Methanos...	CDD	UniProt	Pfam	InterPro	
MSLAZ_3098	AKB76359	Chemotaxis regulator - transmits chemorece...	CDD	UniProt	Pfam	InterPro	
MSLAZ_3099	AKB76360	Two-component response regulator [Methanos...	CDD	UniProt	Pfam	InterPro	
**REC-CheB**							
MSLAZ_3097	AKB76358	Chemotaxis response regulator protein-glut...	CDD	UniProt	Pfam	InterPro	
**wHTH-REC**							
MSLAZ_0950	AKB74211	two-component response regulator [Methanos...	CDD	UniProt	Pfam	InterPro	
**REC-MetOD1**							
MSLAZ_1145	AKB74406	Response regulator receiver [Methanosarcin...	CDD	UniProt	Pfam	InterPro	(REC-MetOD1)

Methanosarcina mazei Go1

**HisK, no REC**						
MM_0168	AAM29864	hypothetical sensory transduction histidin...	CDD	UniProt	Pfam	InterPro
MM_0169	AAM29865	hypothetical sensory transduction histidin...	CDD	UniProt	Pfam	InterPro
MM_0328	AAM30024	Chemotaxis protein [Methanosarcina mazei G...	CDD	UniProt	Pfam	InterPro
MM_0518	AAM30214	hypothetical sensory transduction histidin...	CDD	UniProt	Pfam	InterPro
MM_0889	AAM30585	hypothetical protein MM_0889 [Methanosarci...	CDD	UniProt	Pfam	InterPro
MM_0948	AAM30644	Sensory transduction histidine kinases [Me...	CDD	UniProt	Pfam	InterPro
MM_1093	AAM30789	hypothetical sensory transduction histidin...	CDD	UniProt	Pfam	InterPro
MM_1325	AAM31021	Chemotaxis protein [Methanosarcina mazei G...	CDD	UniProt	Pfam	InterPro
MM_1671	AAM31367	hypothetical sensory transduction histidin...	CDD	UniProt	Pfam	InterPro
MM_1777	AAM31473	hypothetical sensory transduction histidin...	CDD	UniProt	Pfam	InterPro
MM_1781	AAM31477	hypothetical sensory transduction histidin...	CDD	UniProt	Pfam	InterPro
MM_1915	AAM31611	hypothetical sensory transduction histidin...	CDD	UniProt	Pfam	InterPro
MM_1931	AAM31627	sensory transduction protein kinase [Metha...	CDD	UniProt	Pfam	InterPro
MM_2178	AAM31874	Two component system histidine kinase [Met...	CDD	UniProt	Pfam	InterPro
MM_2275	AAM31971	hypothetical sensory transduction histidin...	CDD	UniProt	Pfam	InterPro
MM_2276	AAM31972	hypothetical sensory transduction histidin...	CDD	UniProt	Pfam	InterPro
MM_2277	AAM31973	hypothetical sensory transduction histidin...	CDD	UniProt	Pfam	InterPro
MM_2435	AAM32131	hypothetical sensory transduction histidin...	CDD	UniProt	Pfam	InterPro
MM_2515	AAM32211	hypothetical sensory transduction histidin...	CDD	UniProt	Pfam	InterPro
MM_2629	AAM32325	hypothetical sensory transduction histidin...	CDD	UniProt	Pfam	InterPro
MM_2646	AAM32342	sensory transduction protein kinase [Metha...	CDD	UniProt	Pfam	InterPro
MM_2748	AAM32444	hypothetical sensory transduction histidin...	CDD	UniProt	Pfam	InterPro
MM_2773	AAM32469	Two component system histidine kinase [Met...	CDD	UniProt	Pfam	InterPro
MM_2886	AAM32582	hypothetical sensory transduction histidin...	CDD	UniProt	Pfam	InterPro
MM_2955	AAM32651	hypothetical sensory transduction histidin...	CDD	UniProt	Pfam	InterPro
MM_2965	AAM32661	hypothetical sensory transduction histidin...	CDD	UniProt	Pfam	InterPro
MM_2990	AAM32686	hypothetical sensory transduction histidin...	CDD	UniProt	Pfam	InterPro
MM_3099	AAM32795	Two component system histidine kinase [Met...	CDD	UniProt	Pfam	InterPro
MM_3101	AAM32797	Two component system histidine kinase [Met...	CDD	UniProt	Pfam	InterPro
MM_3158	AAM32854	hypothetical protein MM_3158 [Methanosarci...	CDD	UniProt	Pfam	InterPro
MM_3295	AAM32991	hypothetical sensory transduction histidin...	CDD	UniProt	Pfam	InterPro
**Hybrid HisK**						
MM_2881	AAM32577	Sensor histidine kinase/response regulator...	CDD	UniProt	Pfam	InterPro
MM_3205	AAM32901	Two component system histidine kinase [Met...	CDD	UniProt	Pfam	InterPro
**REC only**						

MM_0049	AAM29745	conserved protein [Methanosarcina mazei Go...	CDD	UniProt	Pfam	InterPro	
MM_0330	AAM30026	Chemotaxis protein [Methanosarcina mazei ...	CDD	UniProt	Pfam	InterPro	
MM_1068	AAM30764	Two-component response regulator [Methanos...	CDD	UniProt	Pfam	InterPro	
MM_1327	AAM31023	Chemotaxis protein [Methanosarcina mazei ...	CDD	UniProt	Pfam	InterPro	
MM_1328	AAM31024	Two-component response regulator [Methanos...	CDD	UniProt	Pfam	InterPro	
MM_2516	AAM32212	putative transcriptional regulator [Methan...	CDD	UniProt	Pfam	InterPro	
MM_2880	AAM32576	Response regulator [Methanosarcina mazei G...	CDD	UniProt	Pfam	InterPro	
MM_2953	AAM32649	transcriptional regulator [Methanosarcina ...	CDD	UniProt	Pfam	InterPro	
MM_2954	AAM32650	transcriptional regulator [Methanosarcina ...	CDD	UniProt	Pfam	InterPro	
MM_3206	AAM32902	Response regulator receiver domain [Methan...	CDD	UniProt	Pfam	InterPro	
**REC-HisK**							
MM_2518	AAM32214	Sensory transduction histidine kinases [Me...	CDD	UniProt	Pfam	InterPro	
**REC-CheB**							
MM_0329	AAM30025	protein-glutamate methylesterase [Methanos...	CDD	UniProt	Pfam	InterPro	
MM_1326	AAM31022	protein-glutamate methylesterase [Methanos...	CDD	UniProt	Pfam	InterPro	
**wHTH-REC**							
MM_2351	AAM32047	transcriptional regulator [Methanosarcina ...	CDD	UniProt	Pfam	InterPro	(wHTH-REC)
**REC-MetOD1**							
MM_3007	AAM32703	putative transcriptional regulator [Methan...	CDD	UniProt	Pfam	InterPro	(REC-MetOD1)

Methanosarcina siciliae C2J

MSSAC_0011	AKB34601	Sensory transduction histidine kinase [Met...	CDD	UniProt	Pfam	InterPro
MSSAC_0014	AKB34604	sensory transduction histidine kinase [Met...	CDD	UniProt	Pfam	InterPro
MSSAC_0177	AKB34767	sensory transduction histidine kinase [Met...	CDD	UniProt	Pfam	InterPro
MSSAC_0198	AKB34788	sensory transduction histidine kinase [Met...	CDD	UniProt	Pfam	InterPro
MSSAC_0426	AKB35016	sensory transduction histidine kinase [Met...	CDD	UniProt	Pfam	InterPro
MSSAC_0427	AKB35017	hypothetical protein MSSAC_0427 [Methanosa...	CDD	UniProt	Pfam	InterPro
MSSAC_0481	AKB35071	sensory transduction histidine kinase [Met...	CDD	UniProt	Pfam	InterPro
MSSAC_0559	AKB35149	sensory transduction histidine kinase [Met...	CDD	UniProt	Pfam	InterPro
MSSAC_0696	AKB35286	sensory transduction histidine kinase [Met...	CDD	UniProt	Pfam	InterPro
MSSAC_0698	AKB35288	sensory transduction histidine kinase [Met...	CDD	UniProt	Pfam	InterPro
MSSAC_0717	AKB35307	Signal-transducing histidine kinase [Metha...	CDD	UniProt	Pfam	InterPro
MSSAC_0796	AKB35386	hypothetical protein MSSAC_0796 [Methanosa...	CDD	UniProt	Pfam	InterPro
MSSAC_0903	AKB35493	sensory transduction histidine kinase [Met...	CDD	UniProt	Pfam	InterPro
MSSAC_1067	AKB35657	sensory transduction histidine kinase [Met...	CDD	UniProt	Pfam	InterPro
MSSAC_1068	AKB35658	sensory transduction histidine kinase [Met...	CDD	UniProt	Pfam	InterPro
MSSAC_1072	AKB35662	sensory transduction histidine kinase [Met...	CDD	UniProt	Pfam	InterPro
MSSAC_1239	AKB35829	Two component system histidine kinase [Met...	CDD	UniProt	Pfam	InterPro
MSSAC_1480	AKB36070	sensory transduction histidine kinase [Met...	CDD	UniProt	Pfam	InterPro
MSSAC_1602	AKB36192	sensory transduction histidine kinase [Met...	CDD	UniProt	Pfam	InterPro
MSSAC_1607	AKB36197	sensory transduction histidine kinase [Met...	CDD	UniProt	Pfam	InterPro
MSSAC_1609	AKB36199	sensory transduction histidine kinase [Met...	CDD	UniProt	Pfam	InterPro
MSSAC_1656	AKB36246	sensory transduction histidine kinase [Met...	CDD	UniProt	Pfam	InterPro
MSSAC_1658	AKB36248	sensory transduction histidine kinase [Met...	CDD	UniProt	Pfam	InterPro
MSSAC_1905	AKB36495	sensory transduction histidine kinase [Met...	CDD	UniProt	Pfam	InterPro
MSSAC_2044	AKB36634	sensory transduction histidine kinase [Met...	CDD	UniProt	Pfam	InterPro
MSSAC_2244	AKB36834	sensory transduction histidine kinase [Met...	CDD	UniProt	Pfam	InterPro
MSSAC_2263	AKB36853	sensory transduction histidine kinase [Met...	CDD	UniProt	Pfam	InterPro
MSSAC_2379	AKB36969	Sensory transduction histidine kinase [Met...	CDD	UniProt	Pfam	InterPro
MSSAC_2382	AKB36972	Sensory transduction histidine kinase [Met...	CDD	UniProt	Pfam	InterPro
MSSAC_2503	AKB37093	sensory transduction histidine kinase [Met...	CDD	UniProt	Pfam	InterPro
MSSAC_2562	AKB37152	hypothetical protein MSSAC_2562 [Methanosa...	CDD	UniProt	Pfam	InterPro
MSSAC_2585	AKB37175	sensory transduction histidine kinase [Met...	CDD	UniProt	Pfam	InterPro
MSSAC_2666	AKB37256	sensory transduction histidine kinase [Met...	CDD	UniProt	Pfam	InterPro
MSSAC_2668	AKB37258	sensory transduction histidine kinase [Met...	CDD	UniProt	Pfam	InterPro
MSSAC_2881	AKB37471	Sensory transduction histidine kinase [Met...	CDD	UniProt	Pfam	InterPro
MSSAC_2891	AKB37481	hypothetical protein MSSAC_2891 [Methanosa...	CDD	UniProt	Pfam	InterPro
MSSAC_2975	AKB37565	Signal transduction histidine kinase CheA ...	CDD	UniProt	Pfam	InterPro
MSSAC_3292	AKB37882	sensory transduction histidine kinase [Met...	CDD	UniProt	Pfam	InterPro
MSSAC_3700	AKB38290	Sensory transduction histidine kinase [Met...	CDD	UniProt	Pfam	InterPro
MSSAC_3755	AKB38345	sensory transduction histidine kinase [Met...	CDD	UniProt	Pfam	InterPro
MSSAC_4240	AKB38830	sensory transduction histidine kinase [Met...	CDD	UniProt	Pfam	InterPro
MSSAC_4337	AKB38927	Signal transduction histidine kinase CheA ...	CDD	UniProt	Pfam	InterPro
**Hybrid HisK**						
MSSAC_4062	AKB38652	Sensory transduction histidine kinase [Met...	CDD	UniProt	Pfam	InterPro
HisKA, no HATPase						
MSSAC_1776	AKB36366	Sensory transduction histidine kinase [Met...	CDD	UniProt	Pfam	InterPro
**REC only**						
MSSAC_1073	AKB35663	Two-component system response regulator [M...	CDD	UniProt	Pfam	InterPro
MSSAC_1074	AKB35664	Two-component system response regulator [M...	CDD	UniProt	Pfam	InterPro
MSSAC_1481	AKB36071	response regulator receiver [Methanosarcin...	CDD	UniProt	Pfam	InterPro
MSSAC_2383	AKB36973	response regulator receiver [Methanosarcin...	CDD	UniProt	Pfam	InterPro
MSSAC_2384	AKB36974	response regulator receiver [Methanosarcin...	CDD	UniProt	Pfam	InterPro
MSSAC_2852	AKB37442	Response regulator receiver [Methanosarcin...	CDD	UniProt	Pfam	InterPro
MSSAC_2973	AKB37563	Chemotaxis regulator - transmits chemorece...	CDD	UniProt	Pfam	InterPro
MSSAC_4061	AKB38651	Two-component response regulator [Methanos...	CDD	UniProt	Pfam	InterPro
MSSAC_4339	AKB38929	Chemotaxis regulator - transmits chemorece...	CDD	UniProt	Pfam	InterPro
MSSAC_4340	AKB38930	Two-component response regulator [Methanos...	CDD	UniProt	Pfam	InterPro

*MSSAC_2270 AKB36860 response regulator receiver [Methanosarcin... CDD UniProt Pfam InterPro
REC-HisK
MSSAC_1075 AKB35665 Sensory transduction histidine kinase [Met... CDD UniProt Pfam InterPro
MSSAC_2826 AKB37416 hypothetical protein MSSAC_2826 [Methanosa... CDD UniProt Pfam InterPro
REC-CheB
MSSAC_2974 AKB37564 Chemotaxis response regulator protein-glut... CDD UniProt Pfam InterPro
MSSAC_4338 AKB38928 Chemotaxis response regulator protein-glut... CDD UniProt Pfam InterPro
wHTH-REC
MSSAC_1372 AKB35962 response regulator receiver [Methanosarcin... CDD UniProt Pfam InterPro (wHTH-REC)
REC-MetOD1
MSSAC_2753 AKB37343 Response regulator receiver [Methanosarcin... CDD UniProt Pfam InterPro (REC-MetOD1)

Methanosarcina sp. 795
HisK, no REC
AAY43_03140 ALK04871 chemotaxis protein [Methanosarcina sp. 79... CDD UniProt Pfam InterPro
AAY43_05040 ALK05185 hypothetical protein AAY43_05040 [Methano... CDD UniProt Pfam InterPro
AAY43_05080 ALK05190 hypothetical protein AAY43_05080 [Methano... CDD UniProt Pfam InterPro
AAY43_07605 ALK05587 histidine kinase [Methanosarcina sp. 795]... CDD UniProt Pfam InterPro
AAY43_10675 ALK06067 ATPase [Methanosarcina sp. 795] ... CDD UniProt Pfam InterPro
AAY43_11175 ALK06146 ATPase [Methanosarcina sp. 795] ... CDD UniProt Pfam InterPro
AAY43_12015 ALK06271 histidine kinase [Methanosarcina sp. 795]... CDD UniProt Pfam InterPro
AAY43_00695 ALK06511 hypothetical protein AAY43_00695 [Methano... CDD UniProt Pfam InterPro
AAY43_01580 ALK06525 histidine kinase [Methanosarcina sp. 795]... CDD UniProt Pfam InterPro
AAY43_04720 ALK06595 hypothetical protein AAY43_04720 [Methano... CDD UniProt Pfam InterPro
AAY43_05095 ALK06604 ATPase [Methanosarcina sp. 795] ... CDD UniProt Pfam InterPro
AAY43_08800 ALK06679 hypothetical protein AAY43_08800 [Methano... CDD UniProt Pfam InterPro
AAY43_09950 ALK06704 histidine kinase [Methanosarcina sp. 795]... CDD UniProt Pfam InterPro
AAY43_10595 ALK06732 histidine kinase [Methanosarcina sp. 795]... CDD UniProt Pfam InterPro
AAY43_12700 ALK06779 hypothetical protein AAY43_12700 [Methano... CDD UniProt Pfam InterPro
REC only
AAY43_02695 ALK04804 chemotaxis protein CheY [Methanosarcina s... CDD UniProt Pfam InterPro
AAY43_03150 ALK04873 chemotaxis protein CheY [Methanosarcina s... CDD UniProt Pfam InterPro
AAY43_03155 ALK04874 response regulator receiver protein [Meth... CDD UniProt Pfam InterPro
AAY43_08795 ALK05775 response regulator [Methanosarcina sp. 79... CDD UniProt Pfam InterPro
AAY43_09105 ALK06687 chemotaxis protein CheY [Methanosarcina s... CDD UniProt Pfam InterPro
AAY43_11180 ALK06748 chemotaxis protein CheY [Methanosarcina s... CDD UniProt Pfam InterPro
AAY43_11185 ALK06147 chemotaxis protein CheY [Methanosarcina s... CDD UniProt Pfam InterPro
AAY43_11925 ALK06257 response regulator receiver protein [Meth... CDD UniProt Pfam InterPro
AAY43_12190 ALK06302 chemotaxis protein CheY [Methanosarcina s... CDD UniProt Pfam InterPro
AAY43_12695 ALK06378 chemotaxis protein CheY [Methanosarcina s... CDD UniProt Pfam InterPro
FrzZ family (REC-REC)
AAY43_02690 ALK04803 hypothetical protein AAY43_02690 [Methano... CDD UniProt Pfam InterPro (REC-REC)
REC-CheB
AAY43_03145 ALK04872 chemotaxis protein CheY [Methanosarcina s... CDD UniProt Pfam InterPro
wHTH-REC
AAY43_09385 ALK05869 response regulator receiver protein [Meth... CDD UniProt Pfam InterPro
REC-MetOD1
AAY43_09910 ALK05954 response regulator receiver protein [Meth... CDD UniProt Pfam InterPro (REC-MetOD1)

Methanosarcina sp. Kolksee
HisK, no REC
MSKOL_0340 AKB46117 sensory transduction histidine kinase [Met... CDD UniProt Pfam InterPro
MSKOL_0357 AKB46134 Signal-transducing histidine kinase [Metha... CDD UniProt Pfam InterPro
MSKOL_0564 AKB46341 Sensory transduction histidine kinase [Met... CDD UniProt Pfam InterPro
MSKOL_1045 AKB46822 Sensory transduction histidine kinase [Met... CDD UniProt Pfam InterPro
MSKOL_1121 AKB46898 sensory transduction histidine kinase [Met... CDD UniProt Pfam InterPro
MSKOL_1240 AKB47017 sensory transduction histidine kinase [Met... CDD UniProt Pfam InterPro
MSKOL_1260 AKB47037 Sensory transduction histidine kinase [Met... CDD UniProt Pfam InterPro
MSKOL_1422 AKB47199 sensory transduction histidine kinase [Met... CDD UniProt Pfam InterPro
MSKOL_1484 AKB47261 sensory transduction histidine kinase [Met... CDD UniProt Pfam InterPro
MSKOL_1522 AKB47299 sensory transduction histidine kinase [Met... CDD UniProt Pfam InterPro
MSKOL_1935 AKB47712 sensory transduction histidine kinase [Met... CDD UniProt Pfam InterPro
MSKOL_1981 AKB47758 sensory transduction histidine kinase [Met... CDD UniProt Pfam InterPro
MSKOL_2142 AKB47919 sensory transduction histidine kinase [Met... CDD UniProt Pfam InterPro
MSKOL_2550 AKB48327 sensory transduction histidine kinase [Met... CDD UniProt Pfam InterPro
MSKOL_2674 AKB48451 Sensory transduction histidine kinase [Met... CDD UniProt Pfam InterPro
MSKOL_2698 AKB48475 sensory transduction histidine kinase [Met... CDD UniProt Pfam InterPro
MSKOL_2796 AKB48573 sensory transduction histidine kinase [Met... CDD UniProt Pfam InterPro
MSKOL_3109 AKB48886 sensory transduction histidine kinase [Met... CDD UniProt Pfam InterPro
MSKOL_3208 AKB48985 Sensory transduction histidine kinase [Met... CDD UniProt Pfam InterPro
MSKOL_3471 AKB49248 Signal transduction histidine kinase CheA ... CDD UniProt Pfam InterPro
Hybrid HisK
MSKOL_0213 AKB45990 Sensory transduction histidine kinase [Met... CDD UniProt Pfam InterPro
REC only
MSKOL_0214 AKB45991 Two-component response regulator [Methanos... CDD UniProt Pfam InterPro
MSKOL_1042 AKB46819 response regulator receiver [Methanosarcin... CDD UniProt Pfam InterPro
MSKOL_1044 AKB46821 response regulator receiver [Methanosarcin... CDD UniProt Pfam InterPro

MSKOL_1521 AKB47298 Two-component system response regulator [M... CDD UniProt Pfam InterPro
MSKOL_1767 AKB47544 response regulator receiver [Methanosarcin... CDD UniProt Pfam InterPro
MSKOL_1807 AKB47584 response regulator receiver [Methanosarcin... CDD UniProt Pfam InterPro
MSKOL_2198 AKB47975 Chemotaxis regulator - transmits chemorece... CDD UniProt Pfam InterPro
MSKOL_2358 AKB48135 Response regulator receiver [Methanosarcin... CDD UniProt Pfam InterPro
MSKOL_2859 AKB48636 Chemotaxis regulator - transmits chemorece... CDD UniProt Pfam InterPro
MSKOL_3468 AKB49245 Two-component response regulator [Methanos... CDD UniProt Pfam InterPro
MSKOL_3469 AKB49246 Chemotaxis regulator - transmits chemorece... CDD UniProt Pfam InterPro

REC-HisK

MSKOL_1520 AKB47297 Sensory transduction histidine kinase [Met... CDD UniProt Pfam InterPro

REC-CheB

MSKOL_3470 AKB49247 Chemotaxis response regulator protein-glut... CDD UniProt Pfam InterPro

wHTH-REC

MSKOL_1760 AKB47537 response regulator receiver [Methanosarcin... CDD UniProt Pfam InterPro

REC-MetOD1

MSKOL_2267 AKB48044 Response regulator receiver [Methanosarcin... CDD UniProt Pfam InterPro (REC-MetOD1)

Methanosarcina sp. MTP4

HisK, no REC

MSMTP_0085 AKB23554 hypothetical protein MSMTP_0085 [Methanosa... CDD UniProt Pfam InterPro
MSMTP_0414 AKB23883 hypothetical protein MSMTP_0414 [Methanosa... CDD UniProt Pfam InterPro
MSMTP_0825 AKB24294 sensory transduction histidine kinase [Met... CDD UniProt Pfam InterPro
MSMTP_0939 AKB24408 sensory transduction histidine kinase [Met... CDD UniProt Pfam InterPro
MSMTP_0940 AKB24409 sensory transduction histidine kinase [Met... CDD UniProt Pfam InterPro
MSMTP_0942 AKB24411 sensory transduction histidine kinase [Met... CDD UniProt Pfam InterPro
MSMTP_0943 AKB24412 sensory transduction histidine kinase [Met... CDD UniProt Pfam InterPro
MSMTP_1396 AKB24865 sensory transduction histidine kinase [Met... CDD UniProt Pfam InterPro
MSMTP_1411 AKB24880 sensory transduction histidine kinase [Met... CDD UniProt Pfam InterPro
MSMTP_1460 AKB24929 sensory transduction histidine kinase [Met... CDD UniProt Pfam InterPro
MSMTP_1504 AKB24973 sensory transduction histidine kinase [Met... CDD UniProt Pfam InterPro
MSMTP_1620 AKB25089 hypothetical protein MSMTP_1620 [Methanosa... CDD UniProt Pfam InterPro
MSMTP_1623 AKB25092 Sensory transduction histidine kinase [Met... CDD UniProt Pfam InterPro
MSMTP_1695 AKB25164 sensory transduction histidine kinase [Met... CDD UniProt Pfam InterPro
MSMTP_1707 AKB25176 Sensory transduction histidine kinase [Met... CDD UniProt Pfam InterPro
MSMTP_1722 AKB25191 sensory transduction histidine kinase [Met... CDD UniProt Pfam InterPro
MSMTP_1802 AKB25271 hypothetical protein MSMTP_1802 [Methanosa... CDD UniProt Pfam InterPro
MSMTP_1803 AKB25272 sensory transduction histidine kinase [Met... CDD UniProt Pfam InterPro
MSMTP_1820 AKB25289 sensory transduction histidine kinase [Met... CDD UniProt Pfam InterPro
MSMTP_2016 AKB25485 sensory transduction histidine kinase [Met... CDD UniProt Pfam InterPro
MSMTP_2352 AKB25821 sensory transduction histidine kinase [Met... CDD UniProt Pfam InterPro
MSMTP_2594 AKB26063 hypothetical protein MSMTP_2594 [Methanosa... CDD UniProt Pfam InterPro
MSMTP_2661 AKB26130 Signal-transducing histidine kinase [Metha... CDD UniProt Pfam InterPro
MSMTP_2667 AKB26136 Signal-transducing histidine kinase [Metha... CDD UniProt Pfam InterPro
MSMTP_2678 AKB26147 sensory transduction histidine kinase [Met... CDD UniProt Pfam InterPro
MSMTP_3240 AKB26709 Signal transduction histidine kinase CheA ... CDD UniProt Pfam InterPro

Hybrid HisK

MSMTP_0269 AKB23738 sensory transduction histidine kinase [Met... CDD UniProt Pfam InterPro
MSMTP_1703 AKB25172 Two-component sensor histidine kinase PleC... CDD UniProt Pfam InterPro

REC only

MSMTP_0270 AKB23739 Two-component response regulator [Methano... CDD UniProt Pfam InterPro
MSMTP_0938 AKB24407 response regulator receiver [Methanosarci... CDD UniProt Pfam InterPro
MSMTP_0941 AKB24410 response regulator receiver [Methanosarci... CDD UniProt Pfam InterPro
MSMTP_1102 AKB24571 response regulator receiver [Methanosarci... CDD UniProt Pfam InterPro
MSMTP_1454 AKB24923 multi-sensor signal transduction histidin... CDD UniProt Pfam InterPro
MSMTP_1455 AKB24924 multi-sensor signal transduction histidin... CDD UniProt Pfam InterPro
MSMTP_1621 AKB25090 response regulator receiver [Methanosarci... CDD UniProt Pfam InterPro
MSMTP_1624 AKB25093 response regulator receiver [Methanosarci... CDD UniProt Pfam InterPro
MSMTP_1625 AKB25094 response regulator receiver [Methanosarci... CDD UniProt Pfam InterPro
MSMTP_1704 AKB25173 Two-component response regulator [Methano... CDD UniProt Pfam InterPro
MSMTP_2159 AKB25628 Pole remodelling regulatory diguanylate c... CDD UniProt Pfam InterPro
MSMTP_3237 AKB26706 response regulator receiver [Methanosarci... CDD UniProt Pfam InterPro
MSMTP_3238 AKB26707 Chemotaxis regulator - transmits chemorec... CDD UniProt Pfam InterPro
*MSMTP_1456 AKB24925 sensory transduction histidine kinase [Me... CDD UniProt Pfam InterPro

REC-HisK

MSMTP_1293 AKB24762 hypothetical protein MSMTP_1293 [Methanos... CDD UniProt Pfam InterPro

REC-CheB

MSMTP_3239 AKB26708 Chemotaxis response regulator protein-glu... CDD UniProt Pfam InterPro

REC-MetOD1

MSMTP_1866 AKB25335 Response regulator receiver [Methanosarci... CDD UniProt Pfam InterPro (REC-MetOD1

Methanosarcina sp. WH1

HisK, no REC

MSWH1_0255 AKB20526 sensory transduction histidine kinase [Met... CDD UniProt Pfam InterPro
MSWH1_1026 AKB21297 sensory transduction histidine kinase [Met... CDD UniProt Pfam InterPro
MSWH1_1081 AKB21352 sensory transduction histidine kinase [Met... CDD UniProt Pfam InterPro
MSWH1_1350 AKB21621 sensory transduction histidine kinase [Met... CDD UniProt Pfam InterPro
MSWH1_1353 AKB21624 sensory transduction histidine kinase [Met... CDD UniProt Pfam InterPro

MSWH1_1358	AKB21629	Sensory transduction histidine kinase [Met...	CDD	UniProt	Pfam	InterPro
MSWH1_1483	AKB21754	Sensory transduction histidine kinase [Met...	CDD	UniProt	Pfam	InterPro
MSWH1_1616	AKB21887	Sensory transduction histidine kinase [Met...	CDD	UniProt	Pfam	InterPro
MSWH1_1618	AKB21889	sensory transduction histidine kinase [Met...	CDD	UniProt	Pfam	InterPro
MSWH1_1636	AKB21907	sensory transduction histidine kinase [Met...	CDD	UniProt	Pfam	InterPro
MSWH1_1705	AKB21976	sensory transduction histidine kinase [Met...	CDD	UniProt	Pfam	InterPro
MSWH1_1972	AKB22243	sensory transduction histidine kinase [Met...	CDD	UniProt	Pfam	InterPro
MSWH1_1991	AKB22262	sensory transduction histidine kinase [Met...	CDD	UniProt	Pfam	InterPro
MSWH1_2005	AKB22276	sensory transduction histidine kinase [Met...	CDD	UniProt	Pfam	InterPro
MSWH1_2555	AKB22826	sensory transduction histidine kinase [Met...	CDD	UniProt	Pfam	InterPro
MSWH1_2628	AKB22899	Signal-transducing histidine kinase [Metha...	CDD	UniProt	Pfam	InterPro
MSWH1_2751	AKB23022	sensory transduction histidine kinase [Met...	CDD	UniProt	Pfam	InterPro
MSWH1_2761	AKB23032	sensory transduction histidine kinase [Met...	CDD	UniProt	Pfam	InterPro
MSWH1_2864	AKB23135	sensory transduction histidine kinase [Met...	CDD	UniProt	Pfam	InterPro
MSWH1_3188	AKB23459	Signal transduction histidine kinase CheA ...	CDD	UniProt	Pfam	InterPro

Hybrid HisK

MSWH1_1194	AKB21465	hypothetical protein MSWH1_1194 [Methanos...	CDD	UniProt	Pfam	InterPro
MSWH1_1671	AKB21942	sensory transduction histidine kinase [Me...	CDD	UniProt	Pfam	InterPro

HisKA, no HATPase

MSWH1_2372	AKB22643	Two component system histidine kinase [Met...	CDD	UniProt	Pfam	InterPro

REC only

MSWH1_0476	AKB20747	Two-component response regulator [Methano...	CDD	UniProt	Pfam	InterPro
MSWH1_1193	AKB21464	GGDEF/response regulator receiver domain ...	CDD	UniProt	Pfam	InterPro
MSWH1_1354	AKB21625	response regulator receiver [Methanosarci...	CDD	UniProt	Pfam	InterPro
MSWH1_1501	AKB21772	Response regulator receiver [Methanosarci...	CDD	UniProt	Pfam	InterPro
*MSWH1_1502	AKB21773	sensory transduction histidine kinase [Me...	CDD	UniProt	Pfam	InterPro
MSWH1_1572	AKB21843	Two-component response regulator [Methano...	CDD	UniProt	Pfam	InterPro
MSWH1_1614	AKB21885	response regulator receiver [Methanosarci...	CDD	UniProt	Pfam	InterPro
MSWH1_1615	AKB21886	response regulator receiver [Methanosarci...	CDD	UniProt	Pfam	InterPro
MSWH1_1672	AKB21943	response regulator receiver [Methanosarci...	CDD	UniProt	Pfam	InterPro
MSWH1_1673	AKB21944	Response regulator receiver [Methanosarci...	CDD	UniProt	Pfam	InterPro
MSWH1_3185	AKB23456	Two-component response regulator [Methano...	CDD	UniProt	Pfam	InterPro
MSWH1_3186	AKB23457	Chemotaxis regulator - transmits chemorec...	CDD	UniProt	Pfam	InterPro
*MSWH1_0475	AKB20746	sensory transduction histidine kinase [Me...	CDD	UniProt	Pfam	InterPro
*MSWH1_1574	AKB21845	sensory transduction histidine kinase [Me...	CDD	UniProt	Pfam	InterPro

REC-HisK

MSWH1_1869	AKB22140	hypothetical protein MSWH1_1869 [Methanos...	CDD	UniProt	Pfam	InterPro

REC-CheB

MSWH1_3187	AKB23458	Chemotaxis response regulator protein-glu...	CDD	UniProt	Pfam	InterPro

wHTH-REC

MSWH1_2295	AKB22566	Phosphate regulon transcriptional regulat...	CDD	UniProt	Pfam	InterPro

REC-MetOD1

MSWH1_1245	AKB21516	Response regulator receiver [Methanosarci...	CDD	UniProt	Pfam	InterPro	(REC-MetOD1

Methanosarcina sp. WWM596

MSWHS_0265	AKB17128	sensory transduction histidine kinase [Met...	CDD	UniProt	Pfam	InterPro
MSWHS_1097	AKB17960	sensory transduction histidine kinase [Met...	CDD	UniProt	Pfam	InterPro
MSWHS_1150	AKB18013	sensory transduction histidine kinase [Met...	CDD	UniProt	Pfam	InterPro
MSWHS_1439	AKB18302	sensory transduction histidine kinase [Met...	CDD	UniProt	Pfam	InterPro
MSWHS_1608	AKB18471	sensory transduction histidine kinase [Met...	CDD	UniProt	Pfam	InterPro
MSWHS_1664	AKB18527	sensory transduction histidine kinase [Met...	CDD	UniProt	Pfam	InterPro
MSWHS_1683	AKB18546	sensory transduction histidine kinase [Met...	CDD	UniProt	Pfam	InterPro
MSWHS_1685	AKB18548	Sensory transduction histidine kinase [Met...	CDD	UniProt	Pfam	InterPro
MSWHS_1848	AKB18711	Sensory transduction histidine kinase [Met...	CDD	UniProt	Pfam	InterPro
MSWHS_2128	AKB18991	sensory transduction histidine kinase [Met...	CDD	UniProt	Pfam	InterPro
MSWHS_2277	AKB19140	sensory transduction histidine kinase [Met...	CDD	UniProt	Pfam	InterPro
MSWHS_2287	AKB19150	sensory transduction histidine kinase [Met...	CDD	UniProt	Pfam	InterPro
MSWHS_2410	AKB19273	Signal-transducing histidine kinase [Metha...	CDD	UniProt	Pfam	InterPro
MSWHS_2483	AKB19346	sensory transduction histidine kinase [Met...	CDD	UniProt	Pfam	InterPro
MSWHS_3065	AKB19928	sensory transduction histidine kinase [Met...	CDD	UniProt	Pfam	InterPro
MSWHS_3079	AKB19942	sensory transduction histidine kinase [Met...	CDD	UniProt	Pfam	InterPro
MSWHS_3098	AKB19961	sensory transduction histidine kinase [Met...	CDD	UniProt	Pfam	InterPro
MSWHS_3189	AKB20052	sensory transduction histidine kinase [Met...	CDD	UniProt	Pfam	InterPro
MSWHS_3196	AKB20059	Sensory transduction histidine kinase [Met...	CDD	UniProt	Pfam	InterPro
MSWHS_3397	AKB20260	Signal transduction histidine kinase CheA ...	CDD	UniProt	Pfam	InterPro

Hybrid HisK

MSWHS_1269	AKB18132	hypothetical protein MSWHS_1269 [Methanosa...	CDD	UniProt	Pfam	InterPro
MSWHS_1628	AKB18491	sensory transduction histidine kinase [Met...	CDD	UniProt	Pfam	InterPro

HisKA, no HATPase

MSWHS_2744	AKB19607	Two component system histidine kinase [Met...	CDD	UniProt	Pfam	InterPro

REC only

MSWHS_0484	AKB17347	Two-component response regulator [Methanos...	CDD	UniProt	Pfam	InterPro
MSWHS_1268	AKB18131	GGDEF/response regulator receiver domain ...	CDD	UniProt	Pfam	InterPro
MSWHS_1626	AKB18489	Response regulator receiver [Methanosarcin...	CDD	UniProt	Pfam	InterPro
MSWHS_1627	AKB18490	response regulator receiver [Methanosarcin...	CDD	UniProt	Pfam	InterPro
MSWHS_1686	AKB18549	response regulator receiver [Methanosarcin...	CDD	UniProt	Pfam	InterPro
MSWHS_1687	AKB18550	response regulator receiver [Methanosarcin...	CDD	UniProt	Pfam	InterPro
MSWHS_1737	AKB18600	Two-component response regulator [Methanos...	CDD	UniProt	Pfam	InterPro

*MSWHS_1831	AKB18694	sensory transduction histidine kinase [Met...	CDD	UniProt	Pfam	InterPro	
MSWHS_1832	AKB18695	Response regulator receiver [Methanosarcin...	CDD	UniProt	Pfam	InterPro	
MSWHS_3190	AKB20053	response regulator receiver [Methanosarcin...	CDD	UniProt	Pfam	InterPro	
MSWHS_3394	AKB20257	Two-component response regulator [Methanos...	CDD	UniProt	Pfam	InterPro	
MSWHS_3395	AKB20258	Chemotaxis regulator - transmits chemorece...	CDD	UniProt	Pfam	InterPro	
*MSWHS_0483	AKB17346	sensory transduction histidine kinase [Met...	CDD	UniProt	Pfam	InterPro	
*MSWHS_1734	AKB18597	sensory transduction histidine kinase [Met...	CDD	UniProt	Pfam	InterPro	
**REC-HisK**							
MSWHS_1462	AKB18325	hypothetical protein MSWHS_1462 [Methanosa...	CDD	UniProt	Pfam	InterPro	
**REC-CheB**							
MSWHS_3396	AKB20259	Chemotaxis response regulator protein-glut...	CDD	UniProt	Pfam	InterPro	
**wHTH-REC**							
MSWHS_2780	AKB19643	Phosphate regulon transcriptional regulato...	CDD	UniProt	Pfam	InterPro	(wHTH-REC)
**REC-MetOD1**							
MSWHS_1321	AKB18184	Response regulator receiver [Methanosarcin...	CDD	UniProt	Pfam	InterPro	(REC-MetOD1)
**Other RRs**							
MSWHS_1735	AKB18598	sensory transduction histidine kinase [Met...	CDD	UniProt	Pfam	InterPro	(REC-xREC)
*MSWHS_3191	AKB20054	hypothetical protein MSWHS_3191 [Methanosa...	CDD	UniProt	Pfam	InterPro	

Methanosarcina thermophila CHTI-55

**HisK, no REC**							
MSTHC_0124	AKB14442	sensory transduction histidine kinase [Met...	CDD	UniProt	Pfam	InterPro	
MSTHC_0141	AKB14459	sensory transduction histidine kinase [Met...	CDD	UniProt	Pfam	InterPro	
MSTHC_0235	AKB14553	hypothetical protein MSTHC_0235 [Methanosa...	CDD	UniProt	Pfam	InterPro	
MSTHC_0272	AKB14590	sensory transduction histidine kinase [Met...	CDD	UniProt	Pfam	InterPro	
MSTHC_0274	AKB14592	sensory transduction histidine kinase [Met...	CDD	UniProt	Pfam	InterPro	
*MSTHC_0276	AKB14594	sensory transduction histidine kinase [Met...	CDD	UniProt	Pfam	InterPro	
MSTHC_0277	AKB14595	sensory transduction histidine kinase [Met...	CDD	UniProt	Pfam	InterPro	
MSTHC_0360	AKB14678	Sensory transduction histidine kinase [Met...	CDD	UniProt	Pfam	InterPro	
MSTHC_0502	AKB14820	sensory transduction histidine kinase [Met...	CDD	UniProt	Pfam	InterPro	
MSTHC_1085	AKB15403	sensory transduction histidine kinase [Met...	CDD	UniProt	Pfam	InterPro	
MSTHC_1088	AKB15406	sensory transduction histidine kinase [Met...	CDD	UniProt	Pfam	InterPro	
MSTHC_1096	AKB15414	sensory transduction histidine kinase [Met...	CDD	UniProt	Pfam	InterPro	
MSTHC_1155	AKB15473	Sensory transduction histidine kinase [Met...	CDD	UniProt	Pfam	InterPro	
MSTHC_1198	AKB15516	sensory transduction histidine kinase [Met...	CDD	UniProt	Pfam	InterPro	
*MSTHC_1199	AKB15517	sensory transduction histidine kinase [Met...	CDD	UniProt	Pfam	InterPro	
MSTHC_1457	AKB15775	Signal transduction histidine kinase CheA ...	CDD	UniProt	Pfam	InterPro	
MSTHC_1739	AKB16057	Sensory transduction histidine kinase [Met...	CDD	UniProt	Pfam	InterPro	
MSTHC_1916	AKB16234	sensory transduction histidine kinase [Met...	CDD	UniProt	Pfam	InterPro	
MSTHC_1918	AKB16236	sensory transduction histidine kinase [Met...	CDD	UniProt	Pfam	InterPro	
MSTHC_2084	AKB16402	Signal-transducing histidine kinase [Metha...	CDD	UniProt	Pfam	InterPro	
MSTHC_2312	AKB16630	sensory transduction histidine kinase [Met...	CDD	UniProt	Pfam	InterPro	
MSTHC_2537	AKB16855	sensory transduction histidine kinase [Met...	CDD	UniProt	Pfam	InterPro	
**Hybrid HisK**							
MSTHC_1534	AKB15852	Sensory transduction histidine kinase [Met...	CDD	UniProt	Pfam	InterPro	
**REC only**							
MSTHC_0236	AKB14554	response regulator receiver [Methanosarci...	CDD	UniProt	Pfam	InterPro	
MSTHC_0237	AKB14555	response regulator receiver [Methanosarci...	CDD	UniProt	Pfam	InterPro	
MSTHC_0341	AKB14659	Response regulator receiver [Methanosarci...	CDD	UniProt	Pfam	InterPro	
MSTHC_0396	AKB14714	Chemotaxis regulator - transmits chemorec...	CDD	UniProt	Pfam	InterPro	
MSTHC_0501	AKB14819	response regulator receiver [Methanosarci...	CDD	UniProt	Pfam	InterPro	
MSTHT_0940	AKB12698	response regulator receiver [Methanosarci...	CDD	UniProt	Pfam	InterPro	
MSTHC_1454	AKB15772	Two-component response regulator [Methano...	CDD	UniProt	Pfam	InterPro	
MSTHC_1455	AKB15773	Chemotaxis regulator - transmits chemorec...	CDD	UniProt	Pfam	InterPro	
MSTHC_1533	AKB15851	Two-component response regulator [Methano...	CDD	UniProt	Pfam	InterPro	
MSTHC_2311	AKB16629	Two-component system response regulator [...	CDD	UniProt	Pfam	InterPro	
MSTHC_2366	AKB16684	response regulator receiver [Methanosarci...	CDD	UniProt	Pfam	InterPro	
**REC-HisK**							
MSTHC_2310	AKB16628	sensory transduction histidine kinase [Me...	CDD	UniProt	Pfam	InterPro	
**REC-CheB**							
MSTHC_1456	AKB15774	Chemotaxis response regulator protein-glu...	CDD	UniProt	Pfam	InterPro	
**wHTH-REC**							
MSTHT_0853	AKB12611	response regulator receiver [Methanosarci...	CDD	UniProt	Pfam	InterPro	
MSTHC_2418	AKB16736	response regulator receiver [Methanosarci...	CDD	UniProt	Pfam	InterPro	
**REC-MetOD1**							
MSTHC_2530	AKB16848	Response regulator receiver [Methanosarci...	CDD	UniProt	Pfam	InterPro	(REC-MetOD1)

Methanosarcina vacuolata Z-761

**HisK, no REC**						
MSVAZ_0356	AKB42625	sensory transduction histidine kinase [Met...	CDD	UniProt	Pfam	InterPro
MSVAZ_0372	AKB42641	Signal-transducing histidine kinase [Metha...	CDD	UniProt	Pfam	InterPro
MSVAZ_0588	AKB42857	Sensory transduction histidine kinase [Met...	CDD	UniProt	Pfam	InterPro
MSVAZ_0705	AKB42974	sensory transduction histidine kinase [Met...	CDD	UniProt	Pfam	InterPro
MSVAZ_1090	AKB43359	Sensory transduction histidine kinase [Met...	CDD	UniProt	Pfam	InterPro
MSVAZ_1169	AKB43438	sensory transduction histidine kinase [Met...	CDD	UniProt	Pfam	InterPro
MSVAZ_1297	AKB43566	sensory transduction histidine kinase [Met...	CDD	UniProt	Pfam	InterPro
MSVAZ_1321	AKB43590	Sensory transduction histidine kinase [Met...	CDD	UniProt	Pfam	InterPro

MSVAZ_1478	AKB43747	sensory transduction histidine kinase [Met...	CDD	UniProt	Pfam	InterPro	
MSVAZ_1553	AKB43822	sensory transduction histidine kinase [Met...	CDD	UniProt	Pfam	InterPro	
MSVAZ_1591	AKB43860	sensory transduction histidine kinase [Met...	CDD	UniProt	Pfam	InterPro	
MSVAZ_1954	AKB44223	sensory transduction histidine kinase [Met...	CDD	UniProt	Pfam	InterPro	
MSVAZ_2000	AKB44269	sensory transduction histidine kinase [Met...	CDD	UniProt	Pfam	InterPro	
MSVAZ_2146	AKB44415	sensory transduction histidine kinase [Met...	CDD	UniProt	Pfam	InterPro	
MSVAZ_2540	AKB44809	sensory transduction histidine kinase [Met...	CDD	UniProt	Pfam	InterPro	
MSVAZ_2670	AKB44939	Sensory transduction histidine kinase [Met...	CDD	UniProt	Pfam	InterPro	
MSVAZ_2694	AKB44963	sensory transduction histidine kinase [Met...	CDD	UniProt	Pfam	InterPro	
MSVAZ_2797	AKB45066	sensory transduction histidine kinase [Met...	CDD	UniProt	Pfam	InterPro	
MSVAZ_3145	AKB45414	sensory transduction histidine kinase [Met...	CDD	UniProt	Pfam	InterPro	
MSVAZ_3244	AKB45513	Sensory transduction histidine kinase [Met...	CDD	UniProt	Pfam	InterPro	
MSVAZ_3503	AKB45772	Signal transduction histidine kinase CheA ...	CDD	UniProt	Pfam	InterPro	
**Hybrid HisK**							
MSVAZ_0219	AKB42488	Sensory transduction histidine kinase [Met...	CDD	UniProt	Pfam	InterPro	
**REC only**							
MSVAZ_0220	AKB42489	Two-component response regulator [Methano...	CDD	UniProt	Pfam	InterPro	
MSVAZ_1087	AKB43356	response regulator receiver [Methanosarci...	CDD	UniProt	Pfam	InterPro	
MSVAZ_1089	AKB43358	response regulator receiver [Methanosarci...	CDD	UniProt	Pfam	InterPro	
MSVAZ_1590	AKB43859	Two-component system response regulator [...	CDD	UniProt	Pfam	InterPro	
MSVAZ_1791	AKB44060	response regulator receiver [Methanosarci...	CDD	UniProt	Pfam	InterPro	
MSVAZ_1830	AKB44099	response regulator receiver [Methanosarci...	CDD	UniProt	Pfam	InterPro	
MSVAZ_2204	AKB44473	Chemotaxis regulator - transmits chemorec...	CDD	UniProt	Pfam	InterPro	
MSVAZ_2348	AKB44617	Response regulator receiver [Methanosarci...	CDD	UniProt	Pfam	InterPro	
MSVAZ_3499	AKB45768	Two-component response regulator [Methano...	CDD	UniProt	Pfam	InterPro	
MSVAZ_3500	AKB45769	Chemotaxis regulator - transmits chemorec...	CDD	UniProt	Pfam	InterPro	
MSVAZ_3502	AKB45771	Chemotaxis response regulator protein-glu...	CDD	UniProt	Pfam	InterPro	
**REC-HisK**							
MSVAZ_1589	AKB43858	Sensory transduction histidine kinase [Me...	CDD	UniProt	Pfam	InterPro	
**wHTH-REC**							
MSVAZ_1784	AKB44053	response regulator receiver [Methanosarci...	CDD	UniProt	Pfam	InterPro	(wHTH-REC)
**REC-MetOD1**							
MSVAZ_2260	AKB44529	Response regulator receiver [Methanosarci...	CDD	UniProt	Pfam	InterPro	(REC-MetOD1)

Methanosphaerula palustris E1-9c

**HisK, no REC**						
Mpal_0053	ACL15447	PAS/PAC sensor signal transduction histidi...	CDD	UniProt	Pfam	InterPro
Mpal_0058	ACL15452	PAS/PAC sensor signal transduction histidi...	CDD	UniProt	Pfam	InterPro
Mpal_0157	ACL15550	PAS/PAC sensor signal transduction histidi...	CDD	UniProt	Pfam	InterPro
Mpal_0273	ACL15656	PAS/PAC sensor signal transduction histidi...	CDD	UniProt	Pfam	InterPro
Mpal_0574	ACL15946	putative PAS/PAC sensor protein [Methanosp...	CDD	UniProt	Pfam	InterPro
Mpal_0667	ACL16035	PAS/PAC sensor signal transduction histidi...	CDD	UniProt	Pfam	InterPro
Mpal_0877	ACL16235	PAS/PAC sensor signal transduction histidi...	CDD	UniProt	Pfam	InterPro
Mpal_0908	ACL16262	multi-sensor signal transduction histidine...	CDD	UniProt	Pfam	InterPro
Mpal_0994	ACL16345	PAS/PAC sensor signal transduction histidi...	CDD	UniProt	Pfam	InterPro
Mpal_1055	ACL16402	multi-sensor signal transduction histidine...	CDD	UniProt	Pfam	InterPro
Mpal_1172	ACL16514	histidine kinase [Methanosphaerula palustr...	CDD	UniProt	Pfam	InterPro
Mpal_1307	ACL16641	multi-sensor signal transduction histidine...	CDD	UniProt	Pfam	InterPro
Mpal_1656	ACL16968	PAS/PAC sensor signal transduction histidi...	CDD	UniProt	Pfam	InterPro
Mpal_1660	ACL16972	PAS/PAC sensor signal transduction histidi...	CDD	UniProt	Pfam	InterPro
Mpal_1687	ACL16996	PAS/PAC sensor signal transduction histidi...	CDD	UniProt	Pfam	InterPro
Mpal_1776	ACL17083	signal transduction histidine kinase [Meth...	CDD	UniProt	Pfam	InterPro
Mpal_1856	ACL17161	CheA signal transduction histidine kinase ...	CDD	UniProt	Pfam	InterPro
Mpal_2002	ACL17305	PAS/PAC sensor signal transduction histidi...	CDD	UniProt	Pfam	InterPro
Mpal_2205	ACL17501	PAS/PAC sensor signal transduction histidi...	CDD	UniProt	Pfam	InterPro
Mpal_2213	ACL17508	PAS/PAC sensor signal transduction histidi...	CDD	UniProt	Pfam	InterPro
Mpal_2216	ACL17510	PAS/PAC sensor signal transduction histidi...	CDD	UniProt	Pfam	InterPro
Mpal_2222	ACL17515	histidine kinase [Methanosphaerula palustr...	CDD	UniProt	Pfam	InterPro
Mpal_2251	ACL17544	signal transduction histidine kinase, nitr...	CDD	UniProt	Pfam	InterPro
Mpal_2258	ACL17549	PAS/PAC sensor signal transduction histidi...	CDD	UniProt	Pfam	InterPro
Mpal_2259	ACL17550	PAS/PAC sensor signal transduction histidi...	CDD	UniProt	Pfam	InterPro
Mpal_2433	ACL17712	Osmosensitive K+ channel histidine kinase-...	CDD	UniProt	Pfam	InterPro
Mpal_2453	ACL17730	PAS/PAC sensor signal transduction histidi...	CDD	UniProt	Pfam	InterPro
Mpal_2461	ACL17738	PAS/PAC sensor signal transduction histidi...	CDD	UniProt	Pfam	InterPro
Mpal_2554	ACL17826	PAS/PAC sensor signal transduction histidi...	CDD	UniProt	Pfam	InterPro
Mpal_2588	ACL17858	PAS/PAC sensor signal transduction histidi...	CDD	UniProt	Pfam	InterPro
**Hybrid HisK**						
Mpal_1329	ACL16662	CheA signal transduction histidine kinase [...	CDD	UniProt	Pfam	InterPro
Mpal_1518	ACL16832	PAS/PAC sensor hybrid histidine kinase [Met...	CDD	UniProt	Pfam	InterPro
Mpal_1818	ACL17124	PAS/PAC sensor hybrid histidine kinase [Met...	CDD	UniProt	Pfam	InterPro
HisKA, no HATPase						
Mpal_2249	ACL17542	multi-sensor signal transduction histidine...	CDD	UniProt	Pfam	InterPro
**REC only**						
Mpal_1519	ACL16833	response regulator receiver protein [Metha...	CDD	UniProt	Pfam	InterPro
Mpal_1771	ACL17078	response regulator receiver protein [Metha...	CDD	UniProt	Pfam	InterPro
Mpal_1858	ACL17163	response regulator receiver protein [Metha...	CDD	UniProt	Pfam	InterPro
Mpal_2157	ACL17455	response regulator receiver protein [Metha...	CDD	UniProt	Pfam	InterPro
Mpal_2212	ACL17507	response regulator receiver protein [Metha...	CDD	UniProt	Pfam	InterPro

Mpal_2247	ACL17540	response regulator receiver protein [Metha...	CDD	UniProt	Pfam	InterPro	
Mpal_2268	ACL17557	response regulator receiver protein [Metha...	CDD	UniProt	Pfam	InterPro	
**REC-HisK**							
Mpal_0015	ACL15411	response regulator receiver protein [Metha...	CDD	UniProt	Pfam	InterPro	
Mpal_0047	ACL15442	signal transduction histidine kinase [Meth...	CDD	UniProt	Pfam	InterPro	
Mpal_0079	ACL15473	signal transduction histidine kinase [Meth...	CDD	UniProt	Pfam	InterPro	
Mpal_0170	ACL15563	multi-sensor signal transduction histidine...	CDD	UniProt	Pfam	InterPro	
Mpal_0266	ACL15650	multi-sensor signal transduction histidine...	CDD	UniProt	Pfam	InterPro	
Mpal_0715	ACL16081	multi-sensor signal transduction histidine...	CDD	UniProt	Pfam	InterPro	
Mpal_0932	ACL16284	multi-sensor signal transduction histidine...	CDD	UniProt	Pfam	InterPro	
Mpal_1070	ACL16416	multi-sensor signal transduction histidine...	CDD	UniProt	Pfam	InterPro	
Mpal_1167	ACL16509	multi-sensor signal transduction histidine...	CDD	UniProt	Pfam	InterPro	
Mpal_1293	ACL16627	putative PAS/PAC sensor protein [Methanosp...	CDD	UniProt	Pfam	InterPro	
Mpal_1331	ACL16664	multi-sensor signal transduction histidine...	CDD	UniProt	Pfam	InterPro	
Mpal_1514	ACL16828	multi-sensor signal transduction histidine...	CDD	UniProt	Pfam	InterPro	
Mpal_1520	ACL16834	multi-sensor signal transduction histidine...	CDD	UniProt	Pfam	InterPro	
Mpal_1525	ACL16839	multi-sensor signal transduction histidine...	CDD	UniProt	Pfam	InterPro	
Mpal_1640	ACL16952	multi-sensor signal transduction histidine...	CDD	UniProt	Pfam	InterPro	
Mpal_1755	ACL17062	multi-sensor signal transduction histidine...	CDD	UniProt	Pfam	InterPro	
Mpal_1773	ACL17080	response regulator receiver protein [Metha...	CDD	UniProt	Pfam	InterPro	
Mpal_1919	ACL17223	multi-sensor signal transduction histidine...	CDD	UniProt	Pfam	InterPro	
Mpal_2462	ACL17739	multi-sensor signal transduction histidine...	CDD	UniProt	Pfam	InterPro	
Mpal_2477	ACL17753	multi-sensor signal transduction histidine...	CDD	UniProt	Pfam	InterPro	
Mpal_2700	ACL17964	multi-sensor signal transduction histidine...	CDD	UniProt	Pfam	InterPro	
Mpal_2706	ACL17970	hypothetical protein Mpal_2706 [Methanosph...	CDD	UniProt	Pfam	InterPro	
**REC-CheB**							
Mpal_1330	ACL16663	response regulator receiver protein [Metha...	CDD	UniProt	Pfam	InterPro	
Mpal_1857	ACL17162	response regulator receiver protein [Metha...	CDD	UniProt	Pfam	InterPro	
**REC-PAS/GAF**							
Mpal_0048	ACL15443	putative PAS/PAC sensor protein [Methanosp...	CDD	UniProt	Pfam	InterPro	
Mpal_0141	ACL15534	putative PAS/PAC sensor protein [Methanosp...	CDD	UniProt	Pfam	InterPro	
Mpal_0573	ACL15945	response regulator receiver protein [Metha...	CDD	UniProt	Pfam	InterPro	
Mpal_0622	ACL15993	putative PAS/PAC sensor protein [Methanosp...	CDD	UniProt	Pfam	InterPro	
Mpal_1335	ACL16668	putative PAS/PAC sensor protein [Methanosp...	CDD	UniProt	Pfam	InterPro	
Mpal_1709	ACL17017	putative PAS/PAC sensor protein [Methanosp...	CDD	UniProt	Pfam	InterPro	
Mpal_2215	ACL17509	putative PAS/PAC sensor protein [Methanosp...	CDD	UniProt	Pfam	InterPro	
Mpal_2266	ACL17555	putative PAS/PAC sensor protein [Methanosp...	CDD	UniProt	Pfam	InterPro	
Mpal_2464	ACL17740	putative PAS/PAC sensor protein [Methanosp...	CDD	UniProt	Pfam	InterPro	
Mpal_2756	ACL18015	putative PAS/PAC sensor protein [Methanosp...	CDD	UniProt	Pfam	InterPro	
**Other RRs**							
Mpal_1470	ACL16792	response regulator receiver protein [Metha...	CDD	UniProt	Pfam	InterPro	(unique???)
Mpal_1772	ACL17079	methyl-accepting chemotaxis sensory transd...	CDD	UniProt	Pfam	InterPro	(REC-PAS-PAS-HAMP-MCPsignal)
Mpal_2156	ACL17454	response regulator receiver protein [Metha...	CDD	UniProt	Pfam	InterPro	(REC-MetOD2)

Methanospirillum hungatei JF-1

**HisK, no REC**						
Mhun_0005	ABD39786	PAS/PAC sensor signal transduction histidi...	CDD	UniProt	Pfam	InterPro
Mhun_0110	ABD39888	CheA signal transduction histidine kinases...	CDD	UniProt	Pfam	InterPro
Mhun_0170	ABD39946	ATP-binding region, ATPase-like protein [M...	CDD	UniProt	Pfam	InterPro
Mhun_0325	ABD40095	histidine kinase [Methanospirillum hungate...	CDD	UniProt	Pfam	InterPro
Mhun_0358	ABD40128	multi-sensor signal transduction histidine...	CDD	UniProt	Pfam	InterPro
Mhun_0463	ABD40225	putative signal transduction histidine kin...	CDD	UniProt	Pfam	InterPro
Mhun_0471	ABD40233	histidine kinase [Methanospirillum hungate...	CDD	UniProt	Pfam	InterPro
Mhun_0640	ABD40396	multi-sensor signal transduction histidine...	CDD	UniProt	Pfam	InterPro
Mhun_0700	ABD40453	multi-sensor signal transduction histidine...	CDD	UniProt	Pfam	InterPro
Mhun_0885	ABD40635	PAS/PAC sensor signal transduction histidi...	CDD	UniProt	Pfam	InterPro
Mhun_0925	ABD40675	histidine kinase [Methanospirillum hungate...	CDD	UniProt	Pfam	InterPro
Mhun_1199	ABD40946	multi-sensor signal transduction histidine...	CDD	UniProt	Pfam	InterPro
Mhun_1234	ABD40980	multi-sensor signal transduction histidine...	CDD	UniProt	Pfam	InterPro
Mhun_1287	ABD41030	multi-sensor signal transduction histidine...	CDD	UniProt	Pfam	InterPro
Mhun_1346	ABD41086	histidine kinase [Methanospirillum hungate...	CDD	UniProt	Pfam	InterPro
Mhun_1350	ABD41090	multi-sensor signal transduction histidine...	CDD	UniProt	Pfam	InterPro
Mhun_1352	ABD41092	multi-sensor signal transduction histidine...	CDD	UniProt	Pfam	InterPro
Mhun_1413	ABD41148	putative PAS/PAC sensor protein [Methanosp...	CDD	UniProt	Pfam	InterPro
Mhun_1414	ABD41149	signal transduction histidine kinase regul...	CDD	UniProt	Pfam	InterPro
Mhun_1417	ABD41152	multi-sensor signal transduction histidine...	CDD	UniProt	Pfam	InterPro
Mhun_1433	ABD41168	multi-sensor signal transduction histidine...	CDD	UniProt	Pfam	InterPro
Mhun_1435	ABD41170	periplasmic sensor signal transduction his...	CDD	UniProt	Pfam	InterPro
Mhun_1442	ABD41177	multi-sensor signal transduction histidine...	CDD	UniProt	Pfam	InterPro
Mhun_1563	ABD41294	multi-sensor signal transduction histidine...	CDD	UniProt	Pfam	InterPro
Mhun_1647	ABD41378	PAS/PAC sensor signal transduction histidi...	CDD	UniProt	Pfam	InterPro
Mhun_1933	ABD41644	multi-sensor signal transduction histidine...	CDD	UniProt	Pfam	InterPro
Mhun_1958	ABD41668	periplasmic ligand-binding sensor protein ...	CDD	UniProt	Pfam	InterPro
Mhun_1963	ABD41673	periplasmic sensor signal transduction his...	CDD	UniProt	Pfam	InterPro
Mhun_2024	ABD41733	PAS/PAC sensor signal transduction histidi...	CDD	UniProt	Pfam	InterPro
Mhun_2038	ABD41746	signal transduction histidine kinase regul...	CDD	UniProt	Pfam	InterPro
Mhun_2434	ABD42135	multi-sensor signal transduction histidine...	CDD	UniProt	Pfam	InterPro

Mhun_2640	ABD42337	histidine kinase [Methanospirillum hungate...	CDD	UniProt	Pfam	InterPro
Mhun_2642	ABD42339	multi-sensor signal transduction histidine...	CDD	UniProt	Pfam	InterPro
Mhun_2672	ABD42368	PAS/PAC sensor signal transduction histidi...	CDD	UniProt	Pfam	InterPro
Mhun_2676	ABD42372	histidine kinase [Methanospirillum hungate...	CDD	UniProt	Pfam	InterPro
Mhun_2683	ABD42378	PAS/PAC sensor signal transduction histidi...	CDD	UniProt	Pfam	InterPro
Mhun_2687	ABD42382	signal transduction histidine kinase [Meth...	CDD	UniProt	Pfam	InterPro
Mhun_2689	ABD42384	Cache sensor signal transduction histidine...	CDD	UniProt	Pfam	InterPro
Mhun_2693	ABD42388	PAS/PAC sensor signal transduction histidi...	CDD	UniProt	Pfam	InterPro
Mhun_2719	ABD42414	periplasmic sensor signal transduction his...	CDD	UniProt	Pfam	InterPro
Mhun_2775	ABD42470	ATP-binding region, ATPase-like protein [M...	CDD	UniProt	Pfam	InterPro
Mhun_2876	ABD42568	multi-sensor signal transduction histidine...	CDD	UniProt	Pfam	InterPro
Mhun_2965	ABD42653	histidine kinase [Methanospirillum hungate...	CDD	UniProt	Pfam	InterPro
Mhun_3167	ABD42849	multi-sensor signal transduction histidine...	CDD	UniProt	Pfam	InterPro
**Hybrid HisK**						
Mhun_0494	ABD40256	CheA signal transduction histidine kinases...	CDD	UniProt	Pfam	InterPro
Mhun_0989	ABD40739	CheA signal transduction histidine kinase ...	CDD	UniProt	Pfam	InterPro
HisKA, no HATPase						
Mhun_0006	ABD39787	putative PAS/PAC sensor protein [Methanosp...	CDD	UniProt	Pfam	InterPro
**REC only**						
Mhun_0108	ABD39886	Response regulator receiver domain protein...	CDD	UniProt	Pfam	InterPro
Mhun_0126	ABD39903	Response regulator receiver domain protein...	CDD	UniProt	Pfam	InterPro
Mhun_0159	ABD39935	Response regulator receiver domain protein...	CDD	UniProt	Pfam	InterPro
Mhun_0161	ABD39937	Response regulator receiver domain protein...	CDD	UniProt	Pfam	InterPro
Mhun_0315	ABD40085	Response regulator receiver domain protein...	CDD	UniProt	Pfam	InterPro
Mhun_0886	ABD40636	Response regulator receiver domain protein...	CDD	UniProt	Pfam	InterPro
Mhun_0953	ABD40703	Response regulator receiver domain protein...	CDD	UniProt	Pfam	InterPro
Mhun_1439	ABD41174	Response regulator receiver domain protein...	CDD	UniProt	Pfam	InterPro
Mhun_1643	ABD41374	Response regulator receiver domain protein...	CDD	UniProt	Pfam	InterPro
Mhun_1923	ABD41634	Response regulator receiver domain protein...	CDD	UniProt	Pfam	InterPro
Mhun_1941	ABD41652	Response regulator receiver domain protein...	CDD	UniProt	Pfam	InterPro
Mhun_1966	ABD41676	Response regulator receiver domain protein...	CDD	UniProt	Pfam	InterPro
Mhun_2031	ABD41739	Response regulator receiver domain protein...	CDD	UniProt	Pfam	InterPro
Mhun_2037	ABD41745	Response regulator receiver domain protein...	CDD	UniProt	Pfam	InterPro
Mhun_2576	ABD42273	Response regulator receiver domain protein...	CDD	UniProt	Pfam	InterPro
Mhun_2673	ABD42369	Response regulator receiver domain protein...	CDD	UniProt	Pfam	InterPro
Mhun_2684	ABD42379	Response regulator receiver domain protein...	CDD	UniProt	Pfam	InterPro
Mhun_2690	ABD42385	Response regulator receiver domain protein...	CDD	UniProt	Pfam	InterPro
Mhun_2877	ABD42569	Response regulator receiver domain protein...	CDD	UniProt	Pfam	InterPro
Mhun_2879	ABD42571	Response regulator receiver domain protein...	CDD	UniProt	Pfam	InterPro
Mhun_3035	ABD42722	Response regulator receiver domain protein...	CDD	UniProt	Pfam	InterPro
Mhun_3040	ABD42727	Response regulator receiver domain protein...	CDD	UniProt	Pfam	InterPro
Mhun_3041	ABD42728	Response regulator receiver domain protein...	CDD	UniProt	Pfam	InterPro
Mhun_3201	ABD42883	Response regulator receiver domain protein...	CDD	UniProt	Pfam	InterPro
**REC-HisK**						
Mhun_0004	ABD39785	multi-sensor signal transduction histidine...	CDD	UniProt	Pfam	InterPro
Mhun_0160	ABD39936	PAS/PAC sensor hybrid histidine kinase [Me...	CDD	UniProt	Pfam	InterPro
Mhun_0454	ABD40216	signal transduction histidine kinase [Meth...	CDD	UniProt	Pfam	InterPro
Mhun_0493	ABD40255	response regulator receiver sensor signal ...	CDD	UniProt	Pfam	InterPro
Mhun_0545	ABD40305	multi-sensor signal transduction histidine...	CDD	UniProt	Pfam	InterPro
Mhun_0549	ABD40309	multi-sensor signal transduction histidine...	CDD	UniProt	Pfam	InterPro
Mhun_0986	ABD40736	response regulator receiver sensor signal ...	CDD	UniProt	Pfam	InterPro
Mhun_0987	ABD40737	response regulator receiver sensor signal ...	CDD	UniProt	Pfam	InterPro
Mhun_1048	ABD40798	multi-sensor signal transduction histidine...	CDD	UniProt	Pfam	InterPro
Mhun_1208	ABD40955	multi-sensor signal transduction histidine...	CDD	UniProt	Pfam	InterPro
Mhun_1309	ABD41049	multi-sensor signal transduction histidine...	CDD	UniProt	Pfam	InterPro
Mhun_1349	ABD41089	multi-sensor signal transduction histidine...	CDD	UniProt	Pfam	InterPro
Mhun_1353	ABD41093	multi-sensor signal transduction histidine...	CDD	UniProt	Pfam	InterPro
Mhun_1354	ABD41094	multi-sensor signal transduction histidine...	CDD	UniProt	Pfam	InterPro
Mhun_1415	ABD41150	multi-sensor signal transduction histidine...	CDD	UniProt	Pfam	InterPro
Mhun_1419	ABD41154	multi-sensor signal transduction histidine...	CDD	UniProt	Pfam	InterPro
Mhun_1420	ABD41155	multi-sensor signal transduction histidine...	CDD	UniProt	Pfam	InterPro
Mhun_1481	ABD41215	multi-sensor signal transduction histidine...	CDD	UniProt	Pfam	InterPro
Mhun_1649	ABD41380	response regulator receiver sensor signal ...	CDD	UniProt	Pfam	InterPro
Mhun_1723	ABD41446	signal transduction histidine kinase [Meth...	CDD	UniProt	Pfam	InterPro
Mhun_1739	ABD41461	multi-sensor signal transduction histidine...	CDD	UniProt	Pfam	InterPro
Mhun_1792	ABD41513	multi-sensor signal transduction histidine...	CDD	UniProt	Pfam	InterPro
Mhun_1846	ABD41563	multi-sensor signal transduction histidine...	CDD	UniProt	Pfam	InterPro
Mhun_1847	ABD41564	multi-sensor signal transduction histidine...	CDD	UniProt	Pfam	InterPro
Mhun_1943	ABD41654	multi-sensor signal transduction histidine...	CDD	UniProt	Pfam	InterPro
Mhun_1962	ABD41672	multi-sensor signal transduction histidine...	CDD	UniProt	Pfam	InterPro
Mhun_2385	ABD42087	multi-sensor signal transduction histidine...	CDD	UniProt	Pfam	InterPro
Mhun_2636	ABD42333	signal transduction histidine kinase [Meth...	CDD	UniProt	Pfam	InterPro
Mhun_3038	ABD42725	signal transduction histidine kinase [Meth...	CDD	UniProt	Pfam	InterPro
Mhun_3039	ABD42726	response regulator receiver sensor signal ...	CDD	UniProt	Pfam	InterPro
Mhun_3202	ABD42884	response regulator receiver sensor signal ...	CDD	UniProt	Pfam	InterPro
**REC-CheB**						
Mhun_0109	ABD39887	response regulator receiver (CheY-like) mo...	CDD	UniProt	Pfam	InterPro
Mhun_0988	ABD40738	response regulator receiver (CheY-like) mo...	CDD	UniProt	Pfam	InterPro
**REC-PAS/GAF**						

```
Mhun_0118    ABD39895 putative PAS/PAC sensor protein [Methanosp...    CDD   UniProt   Pfam   InterPro
Mhun_0455    ABD40217 response regulator receiver domain protein...    CDD   UniProt   Pfam   InterPro
Mhun_0642    ABD40398 putative PAS/PAC sensor protein [Methanosp...    CDD   UniProt   Pfam   InterPro
Mhun_1026    ABD40776 putative PAS/PAC sensor protein [Methanosp...    CDD   UniProt   Pfam   InterPro
Mhun_1267    ABD41010 putative PAS/PAC sensor protein [Methanosp...    CDD   UniProt   Pfam   InterPro
Mhun_1438    ABD41173 putative PAS/PAC sensor protein [Methanosp...    CDD   UniProt   Pfam   InterPro
Mhun_2026    ABD41735 putative PAS/PAC sensor protein [Methanosp...    CDD   UniProt   Pfam   InterPro
Mhun_2671    ABD42367 response regulator receiver domain protein...    CDD   UniProt   Pfam   InterPro
Mhun_2967    ABD42655 putative PAS/PAC sensor protein [Methanosp...    CDD   UniProt   Pfam   InterPro
REC-PAS-PAS-MCPsignal
Mhun_2551    ABD42250 methyl-accepting chemotaxis sensory transd...    CDD   UniProt   Pfam   InterPro
Other RRs
Mhun_0264    ABD40035 response regulator receiver domain protein...    CDD   UniProt   Pfam   InterPro (no archaeal
hits)
Mhun_0840    ABD40592 response regulator receiver domain protein...    CDD   UniProt   Pfam   InterPro (no archaeal
hits)
Mhun_1200    ABD40947 response regulator receiver domain protein...    CDD   UniProt   Pfam   InterPro (no archaeal
hits)
Mhun_1290    ABD41033 response regulator receiver domain protein...    CDD   UniProt   Pfam   InterPro
Mhun_1440    ABD41175 putative PAS/PAC sensor protein [Methanosp...    CDD   UniProt   Pfam   InterPro (REC-PAS-PAS-
HisKA)
Mhun_1472    ABD41206 response regulator receiver domain protein...    CDD   UniProt   Pfam   InterPro (REC-MetOD2)
Mhun_1572    ABD41303 response regulator receiver domain protein...    CDD   UniProt   Pfam   InterPro
Mhun_1607    ABD41338 response regulator receiver domain protein...    CDD   UniProt   Pfam   InterPro
Mhun_1613    ABD41344 response regulator receiver domain protein...    CDD   UniProt   Pfam   InterPro (REC-MetOD2)
Mhun_1618    ABD41349 response regulator receiver domain protein...    CDD   UniProt   Pfam   InterPro (REC-MetOD2)
Mhun_1724    ABD41447 response regulator receiver domain protein...    CDD   UniProt   Pfam   InterPro
Mhun_2637    ABD42334 putative PAS/PAC sensor protein [Methanosp...    CDD   UniProt   Pfam   InterPro (REC-PAS-HisKA)
Mhun_2638    ABD42335 response regulator receiver domain protein...    CDD   UniProt   Pfam   InterPro
Mhun_2645    ABD42342 putative PAS/PAC sensor protein [Methanosp...    CDD   UniProt   Pfam   InterPro (REC-PAS-HisKA)
Mhun_2686    ABD42381 putative PAS/PAC sensor protein [Methanosp...    CDD   UniProt   Pfam   InterPro (REC-PAS-HisKA)
Mhun_2691    ABD42386 putative PAS/PAC sensor protein [Methanosp...    CDD   UniProt   Pfam   InterPro (REC-PAS-HisKA)
Mhun_2749    ABD42444 response regulator receiver domain protein...    CDD   UniProt   Pfam   InterPro (REC-HATPase)
Mhun_2968    ABD42656 putative PAS/PAC sensor protein [Methanosp...    CDD   UniProt   Pfam   InterPro (REC-PAS-HisKA)
Mhun_2986    ABD42673 putative PAS/PAC sensor protein [Methanosp...    CDD   UniProt   Pfam   InterPro (REC-PAS-PAS-
HisKA)
Mhun_3166    ABD42848 putative PAS/PAC sensor protein [Methanosp...    CDD   UniProt   Pfam   InterPro (REC-PAS-xxx)
```

Methanothermobacter marburgensis str. Marburg

```
HisK, no REC
MTBMA_c05730   ADL58168 predicted sensory transduction histidine...   CDD   UniProt   Pfam   InterPro
MTBMA_c06270   ADL58222 predicted signal transduction histidine ...   CDD   UniProt   Pfam   InterPro
MTBMA_c07310   ADL58326 predicted sensory transduction histidine...   CDD   UniProt   Pfam   InterPro
MTBMA_c08400   ADL58435 predicted sensory transduction histidine...   CDD   UniProt   Pfam   InterPro
MTBMA_c08790   ADL58474 predicted sensory transduction histidine...   CDD   UniProt   Pfam   InterPro
MTBMA_c10000   ADL58595 predicted sensory transduction histidine...   CDD   UniProt   Pfam   InterPro
MTBMA_c12200   ADL58810 predicted sensory transduction histidine...   CDD   UniProt   Pfam   InterPro
MTBMA_c13690   ADL58956 predicted sensory transduction histidine...   CDD   UniProt   Pfam   InterPro
MTBMA_c14790   ADL59058 predicted sensory transduction histidine...   CDD   UniProt   Pfam   InterPro
MTBMA_c15070   ADL59086 predicted signal transduction histidine ...   CDD   UniProt   Pfam   InterPro
MTBMA_c16470   ADL59226 predicted signal transduction histidine ...   CDD   UniProt   Pfam   InterPro
REC-HisK
MTBMA_c08420   ADL58437 predicted sensory transduction histidine...   CDD   UniProt   Pfam   InterPro
MTBMA_c08500   ADL58445 predicted sensory transduction histidine...   CDD   UniProt   Pfam   InterPro
MTBMA_c08550   ADL58450 predicted sensory transduction histidine...   CDD   UniProt   Pfam   InterPro
MTBMA_c12950   ADL58883 predicted sensory transduction histidine...   CDD   UniProt   Pfam   InterPro
MTBMA_c12960   ADL58884 predicted sensory transduction histidine...   CDD   UniProt   Pfam   InterPro
REC-PAS
MTBMA_c08360   ADL58431 predicted sensory transduction regulator...   CDD   UniProt   Pfam   InterPro
REC-PAS-GAF
MTBMA_c03410   ADL57942 predicted signal transduction protein [M...   CDD   UniProt   Pfam   InterPro
REC-BcsA
MTBMA_c09390   ADL58534 predicted sensory transduction regulator...   CDD   UniProt   Pfam   InterPro (REC-BcsA)
REC-MetOD1
MTBMA_c08430   ADL58438 predicted sensory transduction regulator...   CDD   UniProt   Pfam   InterPro (REC-MetOD1)
Other RRs
MTBMA_c01930   ADL57802 predicted signal transduction protein [M...   CDD   UniProt   Pfam   InterPro (REC-PAS-GAF-xxx)
```

Methanothermobacter sp. CaT2

```
HisK, no REC
MTCT_0088    BAM69337 sensory transduction histidine kinase [Me...    CDD   UniProt   Pfam   InterPro
MTCT_0141    BAM69390 sensory transduction histidine kinase [Me...    CDD   UniProt   Pfam   InterPro
MTCT_0244    BAM69493 sensory transduction histidine kinase [Me...    CDD   UniProt   Pfam   InterPro
MTCT_0300    BAM69549 sensory transduction histidine kinase [Me...    CDD   UniProt   Pfam   InterPro
MTCT_0304    BAM69553 sensory transduction histidine kinase [Me...    CDD   UniProt   Pfam   InterPro
MTCT_0384    BAM69633 sensory transduction histidine kinase [Me...    CDD   UniProt   Pfam   InterPro
MTCT_0404    BAM69653 sensory transduction histidine kinase [Me...    CDD   UniProt   Pfam   InterPro
```

MTCT_0548	BAM69797	sensory transduction histidine kinase [Me...	CDD	UniProt	Pfam	InterPro	
MTCT_0739	BAM69988	sensory transduction histidine kinase [Me...	CDD	UniProt	Pfam	InterPro	
MTCT_0895	BAM70144	sensory transduction histidine kinase [Me...	CDD	UniProt	Pfam	InterPro	
MTCT_0989	BAM70237	sensory transduction histidine kinase [Me...	CDD	UniProt	Pfam	InterPro	
MTCT_1151	BAM70399	sensory transduction histidine kinase [Me...	CDD	UniProt	Pfam	InterPro	
**REC only**							
MTCT_0385	BAM69634	sensory transduction regulatory protein [...	CDD	UniProt	Pfam	InterPro	
**REC-HisK**							
MTCT_0386	BAM69635	sensory transduction histidine kinase [Me...	CDD	UniProt	Pfam	InterPro	
MTCT_0395	BAM69644	sensory transduction regulatory protein [...	CDD	UniProt	Pfam	InterPro	
MTCT_0817	BAM70066	sensory transduction regulatory protein [...	CDD	UniProt	Pfam	InterPro	
MTCT_0818	BAM70067	sensory transduction histidine kinase [Me...	CDD	UniProt	Pfam	InterPro	
**REC-PAS/GAF**							
MTCT_1611	BAM70859	sensory transduction regulatory protein [...	CDD	UniProt	Pfam	InterPro	
MTCT_1466	BAM70714	truncated sensory transduction regulatory...	CDD	UniProt	Pfam	InterPro	
**REC-BcsA**							
MTCT_0476	BAM69725	sensory transduction regulatory protein [...	CDD	UniProt	Pfam	InterPro	(REC-BcsA)
**REC-MetOD1**							
MTCT_0380	BAM69629	sensory transduction regulatory protein [...	CDD	UniProt	Pfam	InterPro	(REC-PAS-MetOD1)
MTCT_0387	BAM69636	sensory transduction regulatory protein [...	CDD	UniProt	Pfam	InterPro	(REC-MetOD1)

Methanothermobacter thermautotrophicus str. Delta H

**HisK, no REC**							
MTH_123	AAB84629	sensory transduction histidine kinase [Met...	CDD	UniProt	Pfam	InterPro	
MTH_174	AAB84680	sensory transduction histidine kinase [Met...	CDD	UniProt	Pfam	InterPro	
MTH_292	AAB84798	sensory transduction histidine kinase [Met...	CDD	UniProt	Pfam	InterPro	
MTH_356	AAB84862	sensory transduction histidine kinase [Met...	CDD	UniProt	Pfam	InterPro	
MTH_360	AAB84866	sensory transduction histidine kinase [Met...	CDD	UniProt	Pfam	InterPro	
MTH_444	AAB84950	sensory transduction histidine kinase [Met...	CDD	UniProt	Pfam	InterPro	
MTH_459	AAB84965	sensory transduction histidine kinase [Met...	CDD	UniProt	Pfam	InterPro	
MTH_468	AAB84974	sensory transduction histidine kinase [Met...	CDD	UniProt	Pfam	InterPro	
MTH_619	AAB85125	sensory transduction histidine kinase [Met...	CDD	UniProt	Pfam	InterPro	
MTH_823	AAB85323	sensory transduction histidine kinase [Met...	CDD	UniProt	Pfam	InterPro	
MTH_985	AAB85482	sensory transduction histidine kinase [Met...	CDD	UniProt	Pfam	InterPro	
MTH_1124	AAB85613	sensory transduction histidine kinase [Met...	CDD	UniProt	Pfam	InterPro	
MTH_1260	AAB85749	sensory transduction histidine kinase [Met...	CDD	UniProt	Pfam	InterPro	
**REC only**							
MTH445	AAB84951	sensory transduction regulatory protein [M...	CDD	UniProt	Pfam	InterPro	
**REC-HisK**							
MTH_446	AAB84952	sensory transduction regulatory protein [M...	CDD	UniProt	Pfam	InterPro	
MTH_901	AAB85399	sensory transduction regulatory protein [M...	CDD	UniProt	Pfam	InterPro	
MTH_902	AAB85400	sensory transduction histidine kinase [Met...	CDD	UniProt	Pfam	InterPro	
**REC-PAS/GAF**							
MTH457	AAB84963	sensory transduction regulatory protein [M...	CDD	UniProt	Pfam	InterPro	
MTH1764	AAB86230	sensory transduction regulatory protein [M...	CDD	UniProt	Pfam	InterPro	
**REC-BcsA**							
MTH548	AAB85054	sensory transduction regulatory protein [M...	CDD	UniProt	Pfam	InterPro	(REC-BcsA)
*MTH549	AAB85055	sensory transduction regulatory protein [M...	CDD	UniProt	Pfam	InterPro	
**REC-MetOD1**							
MTH440	AAB84946	sensory transduction regulatory protein [M...	CDD	UniProt	Pfam	InterPro	(REC-PAS-MetOD1)
MTH447	AAB84953	sensory transduction regulatory protein [M...	CDD	UniProt	Pfam	InterPro	(REC-MetOD1)
**Other RRs**							
MTH1607	AAB86080	sensory transduction regulatory protein [M...	CDD	UniProt	Pfam	InterPro	(PAS-GAF-xxx)

Methanothermobacter wolfeii

**HisK, no REC**						
MWSIV6_0538	SCM56481	Sensory transduction histidine kinase {EC...	CDD	UniProt	Pfam	InterPro
MWSIV6_0590	SCM56579	putative sensor histidine kinase pdtaS [M...	CDD	UniProt	Pfam	InterPro
MWSIV6_0703	SCM56787	Sensory transduction histidine kinase {EC...	CDD	UniProt	Pfam	InterPro
MWSIV6_0748	SCM56863	Sensory transduction histidine kinase {EC...	CDD	UniProt	Pfam	InterPro
MWSIV6_0752	SCM56870	Sensory transduction histidine kinase {EC...	CDD	UniProt	Pfam	InterPro
MWSIV6_0761	SCM56887	putative sensor histidine kinase pdtaS [M...	CDD	UniProt	Pfam	InterPro
MWSIV6_0842	SCM57028	putative sensor histidine kinase pdtaS [M...	CDD	UniProt	Pfam	InterPro
MWSIV6_0847	SCM57037	Sensory transduction histidine kinase {EC...	CDD	UniProt	Pfam	InterPro
MWSIV6_0868	SCM57073	putative sensory transduction histidine k...	CDD	UniProt	Pfam	InterPro
MWSIV6_0994	SCM57299	putative sensor histidine kinase pdtaS [M...	CDD	UniProt	Pfam	InterPro
MWSIV6_1199	SCM57679	Sensor protein FixL [Methanothermobacter ...	CDD	UniProt	Pfam	InterPro
MWSIV6_1373	SCM57984	Methyl sulfide methyltransferase-associat...	CDD	UniProt	Pfam	InterPro
MWSIV6_1516	SCM58236	Sensory transduction histidine kinase {EC...	CDD	UniProt	Pfam	InterPro
*MWSIV6_1515	SCM58234	putative signal transduction histidine ki...	CDD	UniProt	Pfam	InterPro
MWSIV6_1652	SCM58475	Sensory transduction histidine kinase {EC...	CDD	UniProt	Pfam	InterPro
**REC only**						
MWSIV6_0829	SCM57006	putative methanogenesis regulatory protei...	CDD	UniProt	Pfam	InterPro
**REC-HisK**						
MWSIV6_0830	SCM57008	putative sensor histidine kinase pdtaS [M...	CDD	UniProt	Pfam	InterPro
MWSIV6_1274	SCM57813	putative transcriptional regulatory prote...	CDD	UniProt	Pfam	InterPro
MWSIV6_1275	SCM57815	Nodulation protein V [Methanothermobacter...	CDD	UniProt	Pfam	InterPro

REC-PAS/GAF
MWSIV6_0305 SCM56061 Acetoacetate metabolism regulatory protei... CDD UniProt Pfam InterPro
MWSIV6_0841 SCM57027 KDP operon transcriptional regulatory pro... CDD UniProt Pfam InterPro
REC-BcsA
MWSIV6_0915 SCM57154 putative glycosyltransferase YdaM [Methan... CDD UniProt Pfam InterPro (REC-BcsA)
REC-MetOD1
MWSIV6_0824 SCM56997 putative transcriptional regulatory prote... CDD UniProt Pfam InterPro (REC-PAS-MetOD1)
MWSIV6_0831 SCM57009 putative transcriptional regulatory prote... CDD UniProt Pfam InterPro (REC-MetOD1)
Other RRs
MWSIV6_0153 SCM55785 Sensory transduction regulatory protein [... CDD UniProt Pfam InterPro (REC-GAF-xxx)

Methanothermococcus okinawensis
HisK, no REC
Metok_1036 AEH07006 integral membrane sensor signal transducti... CDD UniProt Pfam InterPro
Metok_1419 AEH07384 CheA signal transduction histidine kinase ... CDD UniProt Pfam InterPro
REC only
Metok_1037 AEH07007 response regulator receiver protein [Metha... CDD UniProt Pfam InterPro
Metok_1412 AEH07377 response regulator receiver protein [Metha... CDD UniProt Pfam InterPro
REC-CheB
Metok_1420 AEH07385 response regulator receiver modulated Chem... CDD UniProt Pfam InterPro

---

Natrialba magadii ATCC 43099
HisK, no REC
Nmag_0168 ADD03760 sensor box histidine kinase [Natrialba mag... CDD UniProt Pfam InterPro
Nmag_0323 ADD03914 sensor box histidine kinase [Natrialba mag... CDD UniProt Pfam InterPro
Nmag_0435 ADD04023 histidine kinase [Natrialba magadii ATCC 4... CDD UniProt Pfam InterPro
Nmag_1296 ADD04876 histidine kinase [Natrialba magadii ATCC 4... CDD UniProt Pfam InterPro
Nmag_2062 ADD05632 sensor box histidine kinase [Natrialba mag... CDD UniProt Pfam InterPro
Nmag_2888 ADD06441 taxis sensor histidine kinase CheA [Natria... CDD UniProt Pfam InterPro
Nmag_3106 ADD06658 sensor box histidine kinase [Natrialba mag... CDD UniProt Pfam InterPro
Nmag_3149 ADD06701 taxis sensor histidine kinase CheA [Natria... CDD UniProt Pfam InterPro
Nmag_3188 ADD06738 histidine kinase [Natrialba magadii ATCC 4... CDD UniProt Pfam InterPro
Nmag_3216 ADD06766 sensor box histidine kinase [Natrialba mag... CDD UniProt Pfam InterPro
Nmag_3297 ADD06847 sensor box histidine kinase [Natrialba mag... CDD UniProt Pfam InterPro
Nmag_3535 ADD07085 sensor box histidine kinase [Natrialba mag... CDD UniProt Pfam InterPro
Nmag_3954 ADD07494 sensor box histidine kinase (plasmid) [Nat... CDD UniProt Pfam InterPro
Nmag_4064 ADD07590 sensor box histidine kinase (plasmid) [Nat... CDD UniProt Pfam InterPro
Nmag_4101 ADD07622 sensor box histidine kinase (plasmid) [Nat... CDD UniProt Pfam InterPro
Nmag_4114 ADD07633 histidine kinase (plasmid) [Natrialba maga... CDD UniProt Pfam InterPro
REC only
Nmag_0951 ADD04535 receiver box response regulator [Natrialba... CDD UniProt Pfam InterPro
Nmag_1095 ADD04679 receiver box response regulator [Natrialba... CDD UniProt Pfam InterPro
Nmag_1130 ADD04714 receiver box response regulator [Natrialba... CDD UniProt Pfam InterPro
Nmag_1797 ADD05371 receiver box response regulator [Natrialba... CDD UniProt Pfam InterPro
Nmag_2693 ADD06249 receiver box response regulator [Natrialba... CDD UniProt Pfam InterPro
Nmag_2877 ADD06430 response regulator CheY [Natrialba magadii... CDD UniProt Pfam InterPro
Nmag_3151 ADD06703 response regulator CheY [Natrialba magadii... CDD UniProt Pfam InterPro
Nmag_3379 ADD06929 receiver box response regulator [Natrialba... CDD UniProt Pfam InterPro
Nmag_3389 ADD06939 receiver box response regulator [Natrialba... CDD UniProt Pfam InterPro
Nmag_3679 ADD07225 receiver box response regulator (plasmid) ... CDD UniProt Pfam InterPro
REC-HisK
Nmag_2042 ADD05612 receiver/sensor box histidine kinase [Natr... CDD UniProt Pfam InterPro
Nmag_3740 ADD07282 receiver/sensor box histidine kinase (plas... CDD UniProt Pfam InterPro
REC-CheB
Nmag_2887 ADD06440 protein-glutamate methylesterase CheB [Nat... CDD UniProt Pfam InterPro
Nmag_3150 ADD06702 protein-glutamate methylesterase CheB [Nat... CDD UniProt Pfam InterPro
REC-HalX
Nmag_3536 ADD07086 receiver box response regulator [Natrialba... CDD UniProt Pfam InterPro
Nmag_3741 ADD07283 receiver box response regulator (plasmid) ... CDD UniProt Pfam InterPro
HlxR-REC
Nmag_2044 ADD05614 HxlR family transcription regulator / rece... CDD UniProt Pfam InterPro (wHTH-REC)
BAT
Nmag_1012 ADD04596 receiver/sensor box HTH-10 family transcri... CDD UniProt Pfam InterPro
Nmag_2147 ADD05716 receiver/bat box HTH-10 family transcripti... CDD UniProt Pfam InterPro
Nmag_2692 ADD06248 receiver/bat box HTH-10 family transcripti... CDD UniProt Pfam InterPro
Nmag_3054 ADD06606 sensor/bat box HTH-10 family transcription... CDD UniProt Pfam InterPro
Other RRs
Nmag_2043 ADD05613 receiver box response regulator [Natrialba... CDD UniProt Pfam InterPro (REC-xHisK)

Natrialbaceae archaeon JW/NM-HA 15
HisK, no REC
B1756_00280 ARS91605 histidine kinase [Natrialbaceae archaeon ... CDD UniProt Pfam InterPro
B1756_00460 ARS88377 hypothetical protein B1756_00460 [Natrial... CDD UniProt Pfam InterPro
B1756_02660 ARS88765 hypothetical protein B1756_02660 [Natrial... CDD UniProt Pfam InterPro
B1756_02685 ARS88770 chemotaxis protein CheA [Natrialbaceae ar... CDD UniProt Pfam InterPro
B1756_03155 ARS88850 hypothetical protein B1756_03155 [Natrial... CDD UniProt Pfam InterPro

B1756_03245	ARS88865	hypothetical protein B1756_03245 [Natrial...	CDD	UniProt	Pfam	InterPro	
B1756_03310	ARS88877	hypothetical protein B1756_03310 [Natrial...	CDD	UniProt	Pfam	InterPro	
B1756_04810	ARS89140	PAS domain-containing sensor histidine ki...	CDD	UniProt	Pfam	InterPro	
B1756_05205	ARS89202	histidine kinase [Natrialbaceae archaeon ...	CDD	UniProt	Pfam	InterPro	
B1756_05510	ARS91700	hypothetical protein B1756_05510 [Natrial...	CDD	UniProt	Pfam	InterPro	
B1756_06735	ARS89470	hypothetical protein B1756_06735 [Natrial...	CDD	UniProt	Pfam	InterPro	
B1756_08005	ARS89689	histidine kinase [Natrialbaceae archaeon ...	CDD	UniProt	Pfam	InterPro	
B1756_08185	ARS89720	hypothetical protein B1756_08185 [Natrial...	CDD	UniProt	Pfam	InterPro	
B1756_09130	ARS89881	hypothetical protein B1756_09130 [Natrial...	CDD	UniProt	Pfam	InterPro	
B1756_10135	ARS90050	PAS domain-containing sensor histidine ki...	CDD	UniProt	Pfam	InterPro	
B1756_11295	ARS90250	hypothetical protein B1756_11295 [Natrial...	CDD	UniProt	Pfam	InterPro	
B1756_13070	ARS90563	hypothetical protein B1756_13070 [Natrial...	CDD	UniProt	Pfam	InterPro	
B1756_14660	ARS90842	hypothetical protein B1756_14660 [Natrial...	CDD	UniProt	Pfam	InterPro	
B1756_14665	ARS90843	PAS domain-containing sensor histidine ki...	CDD	UniProt	Pfam	InterPro	
B1756_14930	ARS90894	hypothetical protein B1756_14930 [Natrial...	CDD	UniProt	Pfam	InterPro	
B1756_16740	ARS91211	hypothetical protein B1756_16740 [Natrial...	CDD	UniProt	Pfam	InterPro	
B1756_16745	ARS91212	hypothetical protein B1756_16745 [Natrial...	CDD	UniProt	Pfam	InterPro	
B1756_16750	ARS91213	hypothetical protein B1756_16750 [Natrial...	CDD	UniProt	Pfam	InterPro	
B1756_17185	ARS91283	hypothetical protein B1756_17185 [Natrial...	CDD	UniProt	Pfam	InterPro	
B1756_17710	ARS91374	hypothetical protein B1756_17710 [Natrial...	CDD	UniProt	Pfam	InterPro	
B1756_17815	ARS91392	hypothetical protein B1756_17815 [Natrial...	CDD	UniProt	Pfam	InterPro	
B1756_18170	ARS91456	hypothetical protein B1756_18170 [Natrial...	CDD	UniProt	Pfam	InterPro	
B1756_18270	ARS91475	histidine kinase [Natrialbaceae archaeon ...	CDD	UniProt	Pfam	InterPro	
B1756_18940	ARS91593	hypothetical protein B1756_18940 [Natrial...	CDD	UniProt	Pfam	InterPro	
**REC only**							
B1756_02720	ARS88777	response regulator [Natrialbaceae archaeo...	CDD	UniProt	Pfam	InterPro	
B1756_03235	ARS88863	response regulator [Natrialbaceae archaeo...	CDD	UniProt	Pfam	InterPro	
B1756_05620	ARS89274	response regulator [Natrialbaceae archaeo...	CDD	UniProt	Pfam	InterPro	
B1756_05735	ARS89293	response regulator [Natrialbaceae archaeo...	CDD	UniProt	Pfam	InterPro	
B1756_11110	ARS90219	response regulator [Natrialbaceae archaeo...	CDD	UniProt	Pfam	InterPro	
B1756_16040	ARS91093	response regulator [Natrialbaceae archaeo...	CDD	UniProt	Pfam	InterPro	
**REC-HisK**							
B1756_07945	ARS89679	hybrid sensor histidine kinase/response r...	CDD	UniProt	Pfam	InterPro	
B1756_10015	ARS90029	hybrid sensor histidine kinase/response r...	CDD	UniProt	Pfam	InterPro	
B1756_13745	ARS90686	hypothetical protein B1756_13745 [Natrial...	CDD	UniProt	Pfam	InterPro	
B1756_16090	ARS91893	hypothetical protein B1756_16090 [Natrial...	CDD	UniProt	Pfam	InterPro	
B1756_17180	ARS91282	hybrid sensor histidine kinase/response r...	CDD	UniProt	Pfam	InterPro	
B1756_17715	ARS91375	hypothetical protein B1756_17715 [Natrial...	CDD	UniProt	Pfam	InterPro	
B1756_18395	ARS91498	hypothetical protein B1756_18395 [Natrial...	CDD	UniProt	Pfam	InterPro	
**REC-CheB**							
B1756_02690	ARS88771	chemotaxis response regulator protein-glu...	CDD	UniProt	Pfam	InterPro	
**REC-GAF**							
B1756_04385	ARS89068	diguanylate cyclase [Natrialbaceae archae...	CDD	UniProt	Pfam	InterPro	
**REC-HalX**							
B1756_17720	ARS91376	hypothetical protein B1756_17720 [Natrial...	CDD	UniProt	Pfam	InterPro	
B1756_18265	ARS91474	DNA-binding protein [Natrialbaceae archae...	CDD	UniProt	Pfam	InterPro	
**wHTH-REC**							
B1756_04815	ARS89141	response regulator [Natrialbaceae archaeo...	CDD	UniProt	Pfam	InterPro	(wHTH-REC)
B1756_07935	ARS89677	HxlR family transcriptional regulator [Na...	CDD	UniProt	Pfam	InterPro	(wHTH-REC)
**BAT**							
B1756_04700	ARS91678	diguanylate cyclase [Natrialbaceae archae...	CDD	UniProt	Pfam	InterPro	
B1756_07405	ARS89584	diguanylate cyclase [Natrialbaceae archae...	CDD	UniProt	Pfam	InterPro	
**Other RRs**							
B1756_07940	ARS89678	hypothetical protein B1756_07940 [Natrial...	CDD	UniProt	Pfam	InterPro	(REC-xHisK)

Natrinema pellirubrum DSM 15624

HisK, no REC

Natpe_0091	AGB30037	PAS domain S-box [Natrinema pellirubrum DS...	CDD	UniProt	Pfam	InterPro
Natpe_0202	AGB30142	PAS domain S-box [Natrinema pellirubrum DS...	CDD	UniProt	Pfam	InterPro
Natpe_0405	AGB30336	PAS domain S-box [Natrinema pellirubrum DS...	CDD	UniProt	Pfam	InterPro
Natpe_1306	AGB31211	PAS domain S-box [Natrinema pellirubrum DS...	CDD	UniProt	Pfam	InterPro
Natpe_1362	AGB31266	histidine kinase [Natrinema pellirubrum DS...	CDD	UniProt	Pfam	InterPro
Natpe_1630	AGB31527	PAS domain S-box [Natrinema pellirubrum DS...	CDD	UniProt	Pfam	InterPro
Natpe_1774	AGB31663	histidine kinase [Natrinema pellirubrum DS...	CDD	UniProt	Pfam	InterPro
Natpe_2091	AGB31920	PAS domain S-box [Natrinema pellirubrum DS...	CDD	UniProt	Pfam	InterPro
Natpe_2130	AGB31959	bacteriophytochrome (light-regulated signa...	CDD	UniProt	Pfam	InterPro
Natpe_1778	AGB31667	histidine kinase [Natrinema pellirubrum DS...	CDD	UniProt	Pfam	InterPro
Natpe_2301	AGB32121	signal transduction histidine kinase [Natr...	CDD	UniProt	Pfam	InterPro
Natpe_2392	AGB32208	PAS domain S-box [Natrinema pellirubrum DS...	CDD	UniProt	Pfam	InterPro
Natpe_2522	AGB32334	signal transduction histidine kinase [Natr...	CDD	UniProt	Pfam	InterPro
Natpe_2613	AGB32421	PAS domain S-box [Natrinema pellirubrum DS...	CDD	UniProt	Pfam	InterPro
Natpe_2661	AGB32467	chemotaxis protein histidine kinase-like p...	CDD	UniProt	Pfam	InterPro
Natpe_3074	AGB32867	PAS domain S-box [Natrinema pellirubrum DS...	CDD	UniProt	Pfam	InterPro
Natpe_3111	AGB32904	PAS domain S-box [Natrinema pellirubrum DS...	CDD	UniProt	Pfam	InterPro
Natpe_3358	AGB33145	PAS domain S-box [Natrinema pellirubrum DS...	CDD	UniProt	Pfam	InterPro
Natpe_3397	AGB33183	PAS domain S-box [Natrinema pellirubrum DS...	CDD	UniProt	Pfam	InterPro
Natpe_3462	AGB33237	signal transduction histidine kinase [Natr...	CDD	UniProt	Pfam	InterPro
Natpe_3578	AGB33346	PAS domain S-box [Natrinema pellirubrum DS...	CDD	UniProt	Pfam	InterPro

Natpe_3603 AGB33370 signal transduction histidine kinase [Natr... CDD UniProt Pfam InterPro
REC only
Natpe_2663 AGB32469 response regulator containing CheY-like re... CDD UniProt Pfam InterPro
Natpe_2623 AGB32430 response regulator with CheY-like receiver... CDD UniProt Pfam InterPro
Natpe_2548 AGB32359 response regulator with CheY-like receiver... CDD UniProt Pfam InterPro
Natpe_3756 AGB33518 response regulator with CheY-like receiver... CDD UniProt Pfam InterPro
Natpe_3156 AGB32948 response regulator with CheY-like receiver... CDD UniProt Pfam InterPro
Natpe_2129 AGB31958 response regulator with CheY-like receiver... CDD UniProt Pfam InterPro
*Natpe_1740 AGB31629 hypothetical protein Natpe_1740 [Natrinema... CDD UniProt Pfam InterPro
*Natpe_1744 AGB31633 response regulator with CheY-like receiver... CDD UniProt Pfam InterPro
REC-HisK
Natpe_0289 AGB30223 PAS domain S-box [Natrinema pellirubrum DS... CDD UniProt Pfam InterPro
Natpe_0512 AGB30441 PAS domain S-box [Natrinema pellirubrum DS... CDD UniProt Pfam InterPro
Natpe_0611 AGB30536 PAS domain S-box [Natrinema pellirubrum DS... CDD UniProt Pfam InterPro
Natpe_1750 AGB31639 PAS domain S-box [Natrinema pellirubrum DS... CDD UniProt Pfam InterPro
Natpe_1893 AGB31753 PAS domain S-box [Natrinema pellirubrum DS... CDD UniProt Pfam InterPro
Natpe_2599 AGB32407 PAS domain S-box [Natrinema pellirubrum DS... CDD UniProt Pfam InterPro
Natpe_3144 AGB32936 PAS domain S-box [Natrinema pellirubrum DS... CDD UniProt Pfam InterPro
REC-CheB
Natpe_2662 AGB32468 chemotaxis response regulator containing a... CDD UniProt Pfam InterPro
REC-HalX
Natpe_1772 AGB31661 response regulator with CheY-like receiver... CDD UniProt Pfam InterPro
Natpe_2391 AGB32207 response regulator with CheY-like receiver... CDD UniProt Pfam InterPro
BAT
Natpe_1071 AGB30983 PAS domain S-box [Natrinema pellirubrum DS... CDD UniProt Pfam InterPro
Natpe_1885 AGB31748 PAS domain S-box [Natrinema pellirubrum DS... CDD UniProt Pfam InterPro
Natpe_3155 AGB32947 putative DNA binding protein [Natrinema pe... CDD UniProt Pfam InterPro
Natpe_3634 AGB33399 response regulator with CheY-like receiver... CDD UniProt Pfam InterPro

---

Natrinema sp. J7-2

HisK, no REC
NJ7G_0185 AFO55440 ATP-binding region ATPase domain protein [... CDD UniProt Pfam InterPro
NJ7G_0557 AFO55812 PAS sensor protein [Natrinema sp. J7-2] ... CDD UniProt Pfam InterPro
NJ7G_1164 AFO56411 multi-sensor signal transduction histidine... CDD UniProt Pfam InterPro
NJ7G_1393 AFO56640 PAS sensor protein [Natrinema sp. J7-2] ... CDD UniProt Pfam InterPro
NJ7G_2984 AFO58206 PAS sensor protein [Natrinema sp. J7-2] ... CDD UniProt Pfam InterPro
NJ7G_3067 AFO58288 PAS sensor protein [Natrinema sp. J7-2] ... CDD UniProt Pfam InterPro
NJ7G_3336 AFO58554 PAS sensor protein [Natrinema sp. J7-2] ... CDD UniProt Pfam InterPro
NJ7G_3488 AFO58705 putative signal-transducing histidine kina... CDD UniProt Pfam InterPro
NJ7G_3528 AFO58745 ATP-binding region ATPase domain protein [... CDD UniProt Pfam InterPro
NJ7G_3793 AFO59010 PAS sensor protein [Natrinema sp. J7-2] ... CDD UniProt Pfam InterPro
REC only
NJ7G_0324 AFO55577 response regulator receiver [Natrinema sp.... CDD UniProt Pfam InterPro
NJ7G_0885 AFO56134 response regulator receiver [Natrinema sp.... CDD UniProt Pfam InterPro
NJ7G_2773 AFO58001 PAS sensor protein [Natrinema sp. J7-2]...... CDD UniProt Pfam InterPro
NJ7G_3370 AFO58588 response regulator receiver protein [Natrin.. CDD UniProt Pfam InterPro
NJ7G_3530 AFO58747 response regulator receiver [Natrinema sp. .. CDD UniProt Pfam InterPro
NJ7G_3972 AFO59186 response regulator receiver protein [Natrin.. CDD UniProt Pfam InterPro
REC-HisK
NJ7G_2462 AFO57693 PAS sensor protein [Natrinema sp. J7-2]...... CDD UniProt Pfam InterPro
NJ7G_3303 AFO58522 PAS sensor protein [Natrinema sp. J7-2]...... CDD UniProt Pfam InterPro
NJ7G_3963 AFO59177 PAS sensor protein [Natrinema sp. J7-2] ... CDD UniProt Pfam InterPro
REC-CheB
NJ7G_3529 AFO58746 CheB methylesterase [Natrinema sp. J7-2]..... CDD UniProt Pfam InterPro
REC-HalX
NJ7G_3068 AFO58289 response regulator receiver protein [Natri... CDD UniProt Pfam InterPro
NJ7G_3487 AFO58706 HalX domain protein [Natrinema sp. J7-2]..... CDD UniProt Pfam InterPro
BAT
NJ7G_0213 AFO55468 Bacterio-opsin activator HTH domain protei... CDD UniProt Pfam InterPro
NJ7G_1601 AFO56845 PAS sensor protein [Natrinema sp. J7-2]...... CDD UniProt Pfam InterPro
NJ7G_3971 AFO59185 Bacterio-opsin activator HTH domain protei... CDD UniProt Pfam InterPro

---

Natronobacterium gregoryi SP2

HisK, no REC
Natgr_0515 AFZ71768 chemotaxis protein histidine kinase-like p... CDD UniProt Pfam InterPro
Natgr_0560 AFZ71810 PAS domain S-box [Natronobacterium gregory... CDD UniProt Pfam InterPro
Natgr_1954 AFZ73137 chemotaxis protein histidine kinase-like p... CDD UniProt Pfam InterPro
Natgr_2193 AFZ73371 PAS domain S-box [Natronobacterium gregory... CDD UniProt Pfam InterPro
Natgr_3215 AFZ74345 PAS domain S-box [Natronobacterium gregory... CDD UniProt Pfam InterPro
Natgr_3236 AFZ74365 PAS domain S-box [Natronobacterium gregory... CDD UniProt Pfam InterPro
REC only
Natgr_0517 AFZ71770 response regulator containing CheY-like re... CDD UniProt Pfam InterPro
Natgr_1049 AFZ72279 response regulator with CheY-like receiver... CDD UniProt Pfam InterPro
Natgr_1953 AFZ73136 response regulator containing a CheY-like ... CDD UniProt Pfam InterPro
Natgr_2829 AFZ73973 response regulator with CheY-like receiver... CDD UniProt Pfam InterPro
Natgr_3286 AFZ74411 CheY-like receiver domain-containing prote... CDD UniProt Pfam InterPro
Natgr_3323 AFZ74448 response regulator with CheY-like receiver... CDD UniProt Pfam InterPro

REC-HisK

Natgr_3398 AFZ74518 PAS domain S-box [Natronobacterium gregory... CDD UniProt Pfam InterPro

REC-CheB

Natgr_1955 AFZ73138 chemotaxis response regulator containing a... CDD UniProt Pfam InterPro
Natgr_0516 AFZ71769 chemotaxis response regulator containing a... CDD UniProt Pfam InterPro

REC-HalX

Natgr_3393 AFZ74513 response regulator with CheY-like receiver... CDD UniProt Pfam InterPro

wHTH-REC

Natgr_2194 AFZ73372 response regulator with CheY-like receiver... CDD UniProt Pfam InterPro (wHTH-REC)

BAT

Natgr_1050 AFZ72280 putative DNA binding protein [Natronobacte... CDD UniProt Pfam InterPro
Natgr_1657 AFZ72856 response regulator with CheY-like receiver... CDD UniProt Pfam InterPro
Natgr_3018 AFZ74152 PAS domain S-box [Natronobacterium gregory... CDD UniProt Pfam InterPro
Natgr_3339 AFZ74463 PAS domain S-box [Natronobacterium gregory... CDD UniProt Pfam InterPro

---

Natronococcus occultus SP4

HisK, no REC

Natoc_0064 AGB35946 bacteriophytochrome (light-regulated signa... CDD UniProt Pfam InterPro
Natoc_0123 AGB36004 bacteriophytochrome (light-regulated signa... CDD UniProt Pfam InterPro
Natoc_0362 AGB36227 PAS domain S-box [Natronococcus occultus S... CDD UniProt Pfam InterPro
Natoc_0482 AGB36345 PAS domain S-box [Natronococcus occultus S... CDD UniProt Pfam InterPro
Natoc_0527 AGB36389 PAS domain S-box [Natronococcus occultus S... CDD UniProt Pfam InterPro
Natoc_0531 AGB36393 PAS domain S-box [Natronococcus occultus S... CDD UniProt Pfam InterPro
Natoc_0766 AGB36623 PAS/PAC sensor hybrid histidine kinase [Na... CDD UniProt Pfam InterPro
Natoc_1623 AGB37428 PAS domain S-box [Natronococcus occultus S... CDD UniProt Pfam InterPro
Natoc_2617 AGB38379 PAS domain S-box [Natronococcus occultus S... CDD UniProt Pfam InterPro
Natoc_2674 AGB38436 PAS domain S-box [Natronococcus occultus S... CDD UniProt Pfam InterPro
Natoc_2836 AGB38595 bacteriophytochrome (light-regulated signa... CDD UniProt Pfam InterPro
Natoc_2872 AGB38630 PAS domain S-box [Natronococcus occultus S... CDD UniProt Pfam InterPro
Natoc_3096 AGB38839 PAS domain S-box [Natronococcus occultus S... CDD UniProt Pfam InterPro
Natoc_3124 AGB38865 PAS/PAC sensor signal transduction histidi... CDD UniProt Pfam InterPro
Natoc_3181 AGB38920 bacteriophytochrome (light-regulated signa.. CDD UniProt Pfam InterPro
Natoc_3348 AGB39081 PAS domain S-box [Natronococcus occultus S... CDD UniProt Pfam InterPro
Natoc_3377 AGB39109 PAS domain S-box [Natronococcus occultus S... CDD UniProt Pfam InterPro
Natoc_3594 AGB39312 PAS domain S-box [Natronococcus occultus S... CDD UniProt Pfam InterPro
Natoc_3640 AGB39356 PAS domain S-box [Natronococcus occultus S... CDD UniProt Pfam InterPro
Natoc_3719 AGB39434 bacteriophytochrome (light-regulated signa.. CDD UniProt Pfam InterPro
Natoc_3789 AGB39498 PAS domain S-box [Natronococcus occultus S... CDD UniProt Pfam InterPro
Natoc_3920 AGB39618 signal transduction histidine kinase [Natr... CDD UniProt Pfam InterPro
Natoc_4317 AGB40007 PAS domain S-box (plasmid) [Natronococcus ... CDD UniProt Pfam InterPro

REC only

Natoc_0500 AGB36362 response regulator with CheY-like receiver... CDD UniProt Pfam InterPro
Natoc_0549 AGB36411 response regulator containing a CheY-like ... CDD UniProt Pfam InterPro
Natoc_1460 AGB37271 response regulator containing a CheY-like ... CDD UniProt Pfam InterPro
Natoc_1794 AGB37592 response regulator with CheY-like receiver... CDD UniProt Pfam InterPro
Natoc_1795 AGB37593 CheY-like receiver domain-containing prote... CDD UniProt Pfam InterPro
Natoc_2082 AGB37868 response regulator with CheY-like receiver... CDD UniProt Pfam InterPro
Natoc_2174 AGB37956 response regulator with CheY-like receiver... CDD UniProt Pfam InterPro
Natoc_2257 AGB38035 response regulator with CheY-like receiver... CDD UniProt Pfam InterPro
Natoc_2423 AGB38198 response regulator with CheY-like receiver... CDD UniProt Pfam InterPro

REC-HisK

Natoc_0486 AGB36348 PAS domain S-box [Natronococcus occultus S... CDD UniProt Pfam InterPro
Natoc_2955 AGB38710 PAS domain S-box [Natronococcus occultus S... CDD UniProt Pfam InterPro

REC-HalX

Natoc_1303 AGB37125 response regulator with CheY-like receiver... CDD UniProt Pfam InterPro
Natoc_1629 AGB37434 response regulator with CheY-like receiver... CDD UniProt Pfam InterPro

BAT

Natoc_0262 AGB36137 putative DNA binding protein [Natronococcu... CDD UniProt Pfam InterPro
Natoc_0501 AGB36363 putative DNA binding protein [Natronococcu... CDD UniProt Pfam InterPro
Natoc_1930 AGB37720 PAS domain S-box [Natronococcus occultus S... CDD UniProt Pfam InterPro
Natoc_3414 AGB39146 PAS domain S-box [Natronococcus occultus S... CDD UniProt Pfam InterPro
Natoc_4318 AGB40008 response regulator with CheY-like receiver... CDD UniProt Pfam InterPro

---

Natronomonas moolapensis 8.8.11

HisK, no REC

Nmlp_1339 CCQ35547 histidine kinase [Natronomonas moolapensis... CDD UniProt Pfam InterPro
Nmlp_1567 CCQ35766 histidine kinase [Natronomonas moolapensis... CDD UniProt Pfam InterPro
Nmlp_1633 CCQ35828 sensor box histidine kinase [Natronomonas ... CDD UniProt Pfam InterPro
Nmlp_1636 CCQ35831 sensor box histidine kinase [Natronomonas ... CDD UniProt Pfam InterPro
Nmlp_1963 CCQ36148 sensor box histidine kinase [Natronomonas ... CDD UniProt Pfam InterPro
Nmlp_2197 CCQ36372 histidine kinase [Natronomonas moolapensis... CDD UniProt Pfam InterPro
Nmlp_2234 CCQ36406 sensor box histidine kinase [Natronomonas ... CDD UniProt Pfam InterPro
Nmlp_2331 CCQ36499 sensor box histidine kinase [Natronomonas ... CDD UniProt Pfam InterPro
Nmlp_2493 CCQ36659 sensor box histidine kinase [Natronomonas ... CDD UniProt Pfam InterPro
Nmlp_2654 CCQ36810 histidine kinase [Natronomonas moolapensis... CDD UniProt Pfam InterPro
Nmlp_2689 CCQ36843 taxis sensor histidine kinase CheA [Natron... CDD UniProt Pfam InterPro

Nmlp_2838	CCQ36988	sensor box histidine kinase [Natronomonas ...	CDD	UniProt	Pfam	InterPro	
Nmlp_2853	CCQ37003	sensor box histidine kinase [Natronomonas ...	CDD	UniProt	Pfam	InterPro	
Nmlp_2854	CCQ37004	sensor box histidine kinase [Natronomonas ...	CDD	UniProt	Pfam	InterPro	
Nmlp_2967	CCQ37110	sensor box histidine kinase [Natronomonas ...	CDD	UniProt	Pfam	InterPro	
Nmlp_3040	CCQ37183	histidine kinase [Natronomonas moolapensis...	CDD	UniProt	Pfam	InterPro	
Nmlp_3120	CCQ37262	histidine kinase [Natronomonas moolapensis...	CDD	UniProt	Pfam	InterPro	
Nmlp_3273	CCQ37407	sensor box histidine kinase [Natronomonas ...	CDD	UniProt	Pfam	InterPro	
Nmlp_3396	CCQ37525	sensor box histidine kinase [Natronomonas ...	CDD	UniProt	Pfam	InterPro	
Nmlp_3414	CCQ37542	sensor box histidine kinase [Natronomonas ...	CDD	UniProt	Pfam	InterPro	
Nmlp_3522	CCQ37647	sensor box histidine kinase [Natronomonas ...	CDD	UniProt	Pfam	InterPro	
Nmlp_3744	CCQ37858	sensor box histidine kinase [Natronomonas ...	CDD	UniProt	Pfam	InterPro	
Nmlp_3775	CCQ37888	histidine kinase [Natronomonas moolapensis...	CDD	UniProt	Pfam	InterPro	
Nmlp_3825	CCQ37937	sensor box histidine kinase [Natronomonas ...	CDD	UniProt	Pfam	InterPro	
**REC only**							
Nmlp_3163	CCQ37300	receiver box response regulator [Natronomo...	CDD	UniProt	Pfam	InterPro	
Nmlp_3199	CCQ37336	response regulator CheY [Natronomonas mool...	CDD	UniProt	Pfam	InterPro	
Nmlp_3895	CCQ38007	response regulator CheY [Natronomonas mool...	CDD	UniProt	Pfam	InterPro	
**REC-HisK**							
Nmlp_1112	CCQ35323	receiver/sensor box histidine kinase [Natr...	CDD	UniProt	Pfam	InterPro	
Nmlp_1632	CCQ35827	receiver/sensor box histidine kinase [Natr...	CDD	UniProt	Pfam	InterPro	
Nmlp_2277	CCQ36447	receiver/sensor box histidine kinase [Natr...	CDD	UniProt	Pfam	InterPro	
Nmlp_2278	CCQ36448	receiver box histidine kinase [Natronomona...	CDD	UniProt	Pfam	InterPro	
Nmlp_2339	CCQ36507	receiver/sensor box histidine kinase [Natr...	CDD	UniProt	Pfam	InterPro	
Nmlp_3142	CCQ37280	receiver/sensor box histidine kinase [Natr...	CDD	UniProt	Pfam	InterPro	
Nmlp_3362	CCQ37491	receiver/sensor box histidine kinase [Natr...	CDD	UniProt	Pfam	InterPro	
Nmlp_3385	CCQ37514	receiver/sensor box histidine kinase [Natr...	CDD	UniProt	Pfam	InterPro	
Nmlp_3506	CCQ37631	receiver/sensor box histidine kinase [Natr...	CDD	UniProt	Pfam	InterPro	
Nmlp_3933	CCQ38044	receiver/sensor box histidine kinase [Natr...	CDD	UniProt	Pfam	InterPro	
**REC-CheB**							
Nmlp_2688	CCQ36842	protein-glutamate methylesterase CheB [Nat...	CDD	UniProt	Pfam	InterPro	
**REC-PAS\GAF**							
Nmlp_2008	CCQ36192	receiver box response regulator [Natronomo...	CDD	UniProt	Pfam	InterPro	
Nmlp_3616	CCQ37735	receiver/sensor box protein [Natronomonas ...	CDD	UniProt	Pfam	InterPro	
**REC-HalX**							
Nmlp_1999	CCQ36183	receiver box response regulator [Natronomo...	CDD	UniProt	Pfam	InterPro	
Nmlp_2965	CCQ37108	receiver box response regulator [Natronomo...	CDD	UniProt	Pfam	InterPro	
Nmlp_3118	CCQ37260	receiver box response regulator [Natronomo...	CDD	UniProt	Pfam	InterPro	
Nmlp_3223	CCQ37360	receiver box response regulator [Natronomo...	CDD	UniProt	Pfam	InterPro	
**REC-wHTH**							
Nmlp_3226	CCQ37363	receiver box response regulator [Natronomo...	CDD	UniProt	Pfam	InterPro	(REC-wHTH)
**BAT**							
Nmlp_1294	CCQ35503	receiver/sensor/bat box HTH-10 family tran...	CDD	UniProt	Pfam	InterPro	
Nmlp_1964	CCQ36149	receiver/sensor/bat box HTH-10 family tran...	CDD	UniProt	Pfam	InterPro	
Nmlp_2653	CCQ36809	receiver/bat box HTH-10 family transcripti...	CDD	UniProt	Pfam	InterPro	
Nmlp_2966	CCQ37109	receiver box HTH-10 family transcription r...	CDD	UniProt	Pfam	InterPro	
**Other RRs**							
Nmlp_2279	CCQ36449	receiver box response regulator [Natronomo...	CDD	UniProt	Pfam	InterPro	(CheF-REC)

Natronomonas pharaonis DSM 2160

**HisK, no REC**						
NP_0142A	CAI48162	histidine kinase [Natronomonas pharaonis D...	CDD	UniProt	Pfam	InterPro
NP_0144A	CAI48163	sensor box histidine kinase [Natronomonas ...	CDD	UniProt	Pfam	InterPro
NP_0730A	CAI48456	histidine kinase [Natronomonas pharaonis D...	CDD	UniProt	Pfam	InterPro
NP_1154A	CAI48668	sensor box histidine kinase [Natronomonas ...	CDD	UniProt	Pfam	InterPro
NP_1622A	CAI48902	sensor box histidine kinase [Natronomonas ...	CDD	UniProt	Pfam	InterPro
NP_1640A	CAI48911	sensor box histidine kinase [Natronomonas ...	CDD	UniProt	Pfam	InterPro
NP_1804A	CAI48993	sensor box histidine kinase [Natronomonas ...	CDD	UniProt	Pfam	InterPro
NP_1850A	CAI49016	sensor box histidine kinase [Natronomonas ...	CDD	UniProt	Pfam	InterPro
NP_1882A	CAI49032	sensor box histidine kinase [Natronomonas ...	CDD	UniProt	Pfam	InterPro
NP_1912A	CAI49047	sensor box histidine kinase [Natronomonas ...	CDD	UniProt	Pfam	InterPro
NP_2172A	CAI49177	taxis sensor histidine kinase CheA [Natron...	CDD	UniProt	Pfam	InterPro
NP_2516A	CAI49349	sensor box histidine kinase [Natronomonas ...	CDD	UniProt	Pfam	InterPro
NP_2742A	CAI49462	receiver/sensor box histidine kinase [Natr...	CDD	UniProt	Pfam	InterPro
NP_3356A	CAI49769	sensor box histidine kinase [Natronomonas ...	CDD	UniProt	Pfam	InterPro
NP_3536A	CAI49859	sensor box histidine kinase [Natronomonas ...	CDD	UniProt	Pfam	InterPro
NP_3752A	CAI49967	sensor box histidine kinase [Natronomonas ...	CDD	UniProt	Pfam	InterPro
NP_4606A	CAI50394	sensor box histidine kinase [Natronomonas ...	CDD	UniProt	Pfam	InterPro
NP_4696A	CAI50439	sensor box histidine kinase [Natronomonas ...	CDD	UniProt	Pfam	InterPro
NP_5274A	CAI50728	sensor box histidine kinase [Natronomonas ...	CDD	UniProt	Pfam	InterPro
NP_6064A	CAI50812	sensor box histidine kinase (plasmid) [Nat...	CDD	UniProt	Pfam	InterPro
NP_6080A	CAI50820	sensor box histidine kinase (plasmid) [Nat...	CDD	UniProt	Pfam	InterPro
NP_0090A	CAI48136	sensor box histidine kinase [Natronomonas ...	CDD	UniProt	Pfam	InterPro
NP_1506A	CAI48844	sensor box histidine kinase [Natronomonas ...	CDD	UniProt	Pfam	InterPro
NP_1914A	CAI49048	sensor box histidine kinase [Natronomonas ...	CDD	UniProt	Pfam	InterPro
**REC only**						
NP0140A	CAI48161	receiver box response regulator [Natronomo...	CDD	UniProt	Pfam	InterPro
NP0516A	CAI48349	response regulator CheY [Natronomonas phar...	CDD	UniProt	Pfam	InterPro
NP0682A	CAI48432	receiver box response regulator [Natronomo...	CDD	UniProt	Pfam	InterPro

NP2102A	CAI49142	response regulator CheY [Natronomonas phar...	CDD	UniProt	Pfam	InterPro
**REC-HisK**						
NP0138A	CAI48160	receiver/sensor box histidine kinase [Natr...	CDD	UniProt	Pfam	InterPro
NP0188A	CAI48185	receiver/sensor box histidine kinase [Natr...	CDD	UniProt	Pfam	InterPro
NP0622A	CAI48402	receiver/sensor box histidine kinase [Natr...	CDD	UniProt	Pfam	InterPro
NP0626A	CAI48404	receiver/sensor box histidine kinase [Natr...	CDD	UniProt	Pfam	InterPro
NP0862A	CAI48522	receiver/sensor box histidine kinase [Natr...	CDD	UniProt	Pfam	InterPro
NP1912A	CAI49047	sensor box histidine kinase [Natronomonas ...	CDD	UniProt	Pfam	InterPro
NP2656A	CAI49419	receiver/sensor box histidine kinase [Natr...	CDD	UniProt	Pfam	InterPro
NP3458A	CAI49820	receiver box histidine kinase [Natronomona...	CDD	UniProt	Pfam	InterPro
NP4696A	CAI50439	sensor box histidine kinase [Natronomonas ...	CDD	UniProt	Pfam	InterPro
NP5120A	CAI50651	receiver/sensor box histidine kinase [Natr...	CDD	UniProt	Pfam	InterPro
NP6028A	CAI50794	receiver/sensor box histidine kinase [Natr...	CDD	UniProt	Pfam	InterPro
NP6202A	CAI50881	receiver/sensor box histidine kinase [Natr...	CDD	UniProt	Pfam	InterPro
**REC-CheB**						
NP2174A	CAI49178	protein-glutamate methylesterase CheB [Nat...	CDD	UniProt	Pfam	InterPro
**REC-GAF**						
NP1846A	CAI49014	receiver box response regulator [Natronomo...	CDD	UniProt	Pfam	InterPro
NP2716A	CAI49449	receiver box response regulator [Natronomo...	CDD	UniProt	Pfam	InterPro
**REC-HalX**						
NP0028A	CAI48105	receiver box response regulator [Natronomo...	CDD	UniProt	Pfam	InterPro
NP2906A	CAI49544	receiver box response regulator [Natronomo...	CDD	UniProt	Pfam	InterPro
**BAT**						
NP0654A	CAI48418	receiver/sensor/bat box HTH-10 family tran...	CDD	UniProt	Pfam	InterPro

Palaeococcus pacificus DY20341

**HisK, no REC**						
PAP_04520	AIF69315	hypothetical protein PAP_04520 [Palaeococc...	CDD	UniProt	Pfam	InterPro
**REC only**						
PAP_04530	AIF69317	chemotaxis protein CheY [Palaeococcus paci...	CDD	UniProt	Pfam	InterPro
**REC-CheB**						
PAP_04525	AIF69316	hypothetical protein PAP_04525 [Palaeococc...	CDD	UniProt	Pfam	InterPro

Pyrococcus abyssi GE5

**HisK, no REC**						
PAB1332	CAB50458	cheA chemotaxis histidine kinase [Pyrococc...	CDD	UniProt	Pfam	InterPro
**REC only**						
PAB1330	CAB50460	cheY chemotaxis response regulator [Pyroco...	CDD	UniProt	Pfam	InterPro
**REC-CheB**						
PAB1331	CAB50459	cheB protein-glutamate methylesterase [Pyr...	CDD	UniProt	Pfam	InterPro

Pyrococcus horikoshii OT3

**HisK, no REC**						
PH0484	BAA29572	766aa long hypothetical histidine kinase ...	CDD	UniProt	Pfam	InterPro
**REC only**						
PH0482	BAA29570	120aa long hypothetical chemotaxis protein...	CDD	UniProt	Pfam	InterPro
**REC-CheB**						
PH0483	BAA29571	368aa long hypothetical protein-glutamate ...	CDD	UniProt	Pfam	InterPro

Pyrococcus kukulkanii

**HisK, no REC**						
TQ32_01480	AMM53314	histidine kinase [Pyrococcus kukulkanii] ...	CDD	UniProt	Pfam	InterPro
**REC only**						
TQ32_01470	AMM53312	chemotaxis protein CheY [Pyrococcus kukulk...	CDD	UniProt	Pfam	InterPro
**REC-CheB**						
TQ32_01475	AMM53313	chemotaxis protein CheY [Pyrococcus kukulk...	CDD	UniProt	Pfam	InterPro

Pyrococcus sp. NA2

**HisK, no REC**						
PNA2_1134	AEC52050	histidine kinase (cheA) [Pyrococcus sp. NA...	CDD	UniProt	Pfam	InterPro
**REC only**						
PNA2_1132	AEC52048	chemotaxis response regulator [Pyrococcus ...	CDD	UniProt	Pfam	InterPro
**REC-CheB**						
PNA2_1133	AEC52049	chemotaxis-specific methylesterase [Pyroco...	CDD	UniProt	Pfam	InterPro

Pyrococcus sp. ST04

**HisK, no REC**						
Py04_0537	AFK22139	chemotaxis histidine kinase [Pyrococcus sp...	CDD	UniProt	Pfam	InterPro
**REC only**						
Py04_0535	AFK22137	chemotaxis response regulator [Pyrococcus ...	CDD	UniProt	Pfam	InterPro
**REC-CheB**						
Py04_0536	AFK22138	chemotaxis-specific methylesterase [Pyroco...	CDD	UniProt	Pfam	InterPro

Pyrococcus yayanosii CH1

HisK, no REC

PYCH_15450 AEH25211 chemotaxis histidine kinase [Pyrococcus ya... CDD UniProt Pfam InterPro

REC only

PYCH_15430 AEH25209 chemotaxis response regulator [Pyrococcus ... CDD UniProt Pfam InterPro

REC-CheB

PYCH_15440 AEH25210 chemotaxis-specific methylesterase [Pyroco... CDD UniProt Pfam InterPro

---

Salinarchaeum sp. Harcht-Bsk1

HisK, no REC

L593_00155 AGM99985 signal-transducing histidine kinase [Salin... CDD UniProt Pfam InterPro
L593_00245 AGN00004 chemotaxis protein CheA [Salinarchaeum sp.... CDD UniProt Pfam InterPro
L593_00745 AGN00104 PAS domain S-box [Salinarchaeum sp. Harcht... CDD UniProt Pfam InterPro
L593_01540 AGN00261 PAS/PAC sensor signal transduction histidi... CDD UniProt Pfam InterPro
L593_02555 AGN00462 histidine kinase [Salinarchaeum sp. Harcht... CDD UniProt Pfam InterPro
L593_02640 AGN00479 CheA signal transduction histidine kinase ... CDD UniProt Pfam InterPro
L593_03370 AGN00625 HTR-like protein [Salinarchaeum sp. Harcht... CDD UniProt Pfam InterPro
L593_03635 AGN00678 PAS/PAC sensor signal transduction histidi... CDD UniProt Pfam InterPro
L593_03980 AGN00747 signal-transducing histidine kinase [Salin... CDD UniProt Pfam InterPro
L593_04445 AGN00840 PAS/PAC sensor hybrid histidine kinase [Sa... CDD UniProt Pfam InterPro
L593_04515 AGN00854 histidine kinase [Salinarchaeum sp. Harcht... CDD UniProt Pfam InterPro
L593_04535 AGN00858 HTR-like protein [Salinarchaeum sp. Harcht... CDD UniProt Pfam InterPro
L593_04770 AGN00903 PAS/PAC sensor signal transduction histidi... CDD UniProt Pfam InterPro
L593_06165 AGN01182 redox sensing protein [Salinarchaeum sp. H... CDD UniProt Pfam InterPro
L593_08965 AGN01738 signal-transducing histidine kinase [Salin... CDD UniProt Pfam InterPro
L593_09630 AGN01871 GAF sensor signal transduction histidine k... CDD UniProt Pfam InterPro
L593_10955 AGN02136 PAS domain-containing protein [Salinarchae... CDD UniProt Pfam InterPro
L593_11925 AGN02328 light- and oxygen-sensing transcription re... CDD UniProt Pfam InterPro
L593_12315 AGN02402 histidine kinase [Salinarchaeum sp. Harcht... CDD UniProt Pfam InterPro
L593_13520 AGN02643 pas domain s-box [Salinarchaeum sp. Harcht... CDD UniProt Pfam InterPro
L593_14460 AGN02831 PAS domain S-box [Salinarchaeum sp. Harcht... CDD UniProt Pfam InterPro
L593_14480 AGN02835 PAS sensor protein [Salinarchaeum sp. Harc... CDD UniProt Pfam InterPro
L593_14520 AGN02843 putative signal-transducing histidine kina... CDD UniProt Pfam InterPro

REC only

L593_02335 AGN00418 response regulator containing chey-like re... CDD UniProt Pfam InterPro
L593_00255 AGN00006 response regulator containing CheY-like re... CDD UniProt Pfam InterPro

REC-HisK

L593_04230 AGN00797 multi-sensor signal transduction histidine... CDD UniProt Pfam InterPro
L593_05180 AGN00985 PAS/PAC sensor signal transduction histidi... CDD UniProt Pfam InterPro
L593_06385 AGN01224 Htr-like protein [Salinarchaeum sp. Harcht... CDD UniProt Pfam InterPro
L593_07045 AGN01356 sensor box histidine kinase [Salinarchaeum... CDD UniProt Pfam InterPro
L593_07370 AGN01419 histidine kinase [Salinarchaeum sp. Harcht... CDD UniProt Pfam InterPro
L593_10790 AGN02103 PAS/PAC sensor signal transduction histidi... CDD UniProt Pfam InterPro
L593_11575 AGN02258 HTR-like protein [Salinarchaeum sp. Harcht... CDD UniProt Pfam InterPro
L593_12465 AGN02432 PAS domain S-box [Salinarchaeum sp. Harcht... CDD UniProt Pfam InterPro
L593_14735 AGN02881 PAS/PAC sensor signal transduction histidi... CDD UniProt Pfam InterPro

REC-CheB

L593_00250 AGN00005 chemotaxis-specific methylesterase [Salina... CDD UniProt Pfam InterPro
*L593_02645 AGN00480 chemotaxis-specific methylesterase [Salina... CDD UniProt Pfam InterPro

REC-PAS

L593_03645 AGN00680 HTR-like protein [Salinarchaeum sp. Harcht... CDD UniProt Pfam InterPro

REC-HalX

L593_04530 AGN00857 response regulator receiver protein [Salin... CDD UniProt Pfam InterPro
L593_06565 AGN01260 response regulator with CheY-like receiver... CDD UniProt Pfam InterPro
L593_11525 AGN02248 response regulator with CheY-like receiver... CDD UniProt Pfam InterPro
L593_14935 AGN02921 HalX domain protein [Salinarchaeum sp. Har... CDD UniProt Pfam InterPro

Other RRs

L593_02140 AGN00379 response regulator receiver protein [Salin... CDD UniProt Pfam InterPro (REC-HalOD2)

Thermococcus barophilus

HisK, no REC

TBCH5v1_1428 ALM75345 chemotaxis histidine kinase [Thermococcu... CDD UniProt Pfam InterPro

REC only

TBCH5v1_1430 ALM75347 Chemotaxis protein CheY [Thermococcus ba... CDD UniProt Pfam InterPro

REC-CheB

TBCH5v1_1429 ALM75346 Chemotaxis response regulator protein-gl... CDD UniProt Pfam InterPro

---

Thermococcus eurythermalis

HisK, no REC

TEU_11690 AIU70934 histidine kinase [Thermococcus eurythermal... CDD UniProt Pfam InterPro

REC only

TEU_11680 AIU70932 chemotaxis protein CheY [Thermococcus eury... CDD UniProt Pfam InterPro

REC-CheB

TEU_11685 AIU70933 chemotaxis protein CheY [Thermococcus eury... CDD UniProt Pfam InterPro

---

Thermococcus gammatolerans EJ3

HisK, no REC

TGAM_1529 ACS34031 Chemotaxis histidine kinase (cheA) [Thermo... CDD UniProt Pfam InterPro

REC only

TGAM_1530 ACS34032 Chemotaxis response regulator (cheY) [Ther... CDD UniProt Pfam InterPro

---

Thermococcus guaymasensis DSM 11113

HisK, no REC

X802_00465 AJC70836 histidine kinase [Thermococcus guaymasensi... CDD UniProt Pfam InterPro

REC only

X802_00470 AJC70837 chemotaxis protein CheY [Thermococcus guay... CDD UniProt Pfam InterPro

---

Thermococcus kodakarensis KOD1

HisK, no REC

TK0635 BAD84824 chemotaxis histidine kinase, flame shift [... CDD UniProt Pfam InterPro

REC only

TK0632 BAD84821 chemotaxis response regulator [Thermococcu... CDD UniProt Pfam InterPro

REC-CheB

TK0633 BAD84822 chemotaxis protein-glutamate methylesteras... CDD UniProt Pfam InterPro

---

Thermococcus litoralis DSM 5473

HisK, no REC

OCC_10424 EHR77516 cheA chemotaxis histidine kinase [Thermoco... CDD UniProt Pfam InterPro

REC only

OCC_10414 EHR77514 chemotaxis response regulator [Thermococcu... CDD UniProt Pfam InterPro

REC-CheB

OCC_10419 EHR77515 chemotaxis-specific methylesterase [Thermo... CDD UniProt Pfam InterPro

---

Thermococcus nautili

HisK, no REC

BD01_1162 AHL22779 Chemotaxis protein histidine kinase-relate... CDD UniProt Pfam InterPro

REC only

BD01_1160 AHL22777 CheY-like receiver [Thermococcus nautili] ... CDD UniProt Pfam InterPro

REC-CheB

BD01_1161 AHL22778 Chemotaxis response regulator containing a... CDD UniProt Pfam InterPro

---

Thermococcus onnurineus NA1

HisK, no REC

TON_1198 ACJ16686 cheA chemotaxis histidine kinase [Thermoco... CDD UniProt Pfam InterPro

REC only

TON_1196 ACJ16684 chemotaxis response regulator [Thermococcu... CDD UniProt Pfam InterPro

REC-CheB

TON_1197 ACJ16685 cheB protein-glutamate methylesterase [The... CDD UniProt Pfam InterPro

---

Thermococcus sp. AM4

HisK, no REC

TAM4_1755 EEB74388 Signal transduction histidine kinase CheA ... CDD UniProt Pfam InterPro

REC only

TAM4_1512 EEB74145 chemotaxis response regulator protein-glut... CDD UniProt Pfam InterPro

---

Thermococcus peptonophilus JCM 9653

HisK, no REC

A0127_00765 AMQ17807 chemotaxis rotein CheA [Thermococcus pept... CDD UniProt Pfam InterPro

REC only

A0127_00755 AMQ17805 two-component system response regulator [... CDD UniProt Pfam InterPro

REC-CheB

A0127_00760 AMQ17806 chemotaxis response regulator protein-glu... CDD UniProt Pfam InterPro

---

Thermoplasmatales archaeon BRNA1

Other RRs

TALC_00527 AGI47527 Response regulator containing CheY-like re... CDD UniProt Pfam InterPro (REC-DUF835)

---

unclassified Euryarchaeota

uncultured marine group II euryarchaeote

REC only

MG2_0491 EHR76079 putative acetoacetate metabolism regulator... CDD UniProt Pfam InterPro

Other RRs

MG2_0171 EHR75759 putative Signal transduction response regu... CDD UniProt Pfam InterPro (low complexity)

MG2_0555 EHR76143 putative CheY-like superfamily protein wit... CDD UniProt Pfam InterPro (no archaeal hits)

Thaumarchaeota

Candidatus Nitrocosmicus oleophilus strain MY3

HisK, no REC

NMY3_00019 ALI34233 Sensor protein SrrB [Candidatus Nitrocosmi... CDD UniProt Pfam InterPro
NMY3_00141 ALI34355 Sensory/regulatory protein RpfC [Candidatu... CDD UniProt Pfam InterPro
NMY3_00302 ALI34516 Sensor histidine kinase TodS [Candidatus N... CDD UniProt Pfam InterPro
NMY3_00671 ALI34880 Sensor protein kinase WalK [Candidatus Nit... CDD UniProt Pfam InterPro
NMY3_00763 ALI34972 Sensory/regulatory protein RpfC [Candidatu... CDD UniProt Pfam InterPro
NMY3_00767 ALI34976 Sensor protein DivL [Candidatus Nitrocosmi... CDD UniProt Pfam InterPro
NMY3_00923 ALI35130 Signal transduction histidine-protein kina... CDD UniProt Pfam InterPro
NMY3_01369 ALI35573 Sensor protein SrrB [Candidatus Nitrocosmi... CDD UniProt Pfam InterPro
NMY3_01566 ALI35769 Phytochrome-like protein cph1 [Candidatus ... CDD UniProt Pfam InterPro
NMY3_01835 ALI36038 Non-motile and phage-resistance protein [C... CDD UniProt Pfam InterPro
NMY3_02176 ALI36376 Sensor protein SrrB [Candidatus Nitrocosmi... CDD UniProt Pfam InterPro
NMY3_02759 ALI36949 Non-motile and phage-resistance protein [C... CDD UniProt Pfam InterPro
NMY3_02849 ALI37038 Alkaline phosphatase synthesis sensor prot... CDD UniProt Pfam InterPro
NMY3_02960 ALI37147 Sensor protein SrrB [Candidatus Nitrocosmi... CDD UniProt Pfam InterPro
NMY3_02973 ALI37160 Alkaline phosphatase synthesis sensor prot... CDD UniProt Pfam InterPro
NMY3_02978 ALI37165 Sensor histidine kinase YycG [Candidatus N... CDD UniProt Pfam InterPro
NMY3_03253 ALI37438 Alkaline phosphatase synthesis sensor prot... CDD UniProt Pfam InterPro
NMY3_03423 ALI37606 Alkaline phosphatase synthesis sensor prot... CDD UniProt Pfam InterPro
NMY3_03614 ALI37796 Alkaline phosphatase synthesis sensor prot... CDD UniProt Pfam InterPro
NMY3_03758 ALI37939 Non-motile and phage-resistance protein [C... CDD UniProt Pfam InterPro
*NMY3_01774 ALI35977 Virulence sensor protein BvgS precursor [C... CDD UniProt Pfam InterPro
NMY3_01775 ALI35978 Sensor protein FixL [Candidatus Nitrocosmi... CDD UniProt Pfam InterPro

Hybrid HisK

NMY3_00894 ALI35103 Aerobic respiration control sensor protein... CDD UniProt Pfam InterPro

REC only

NMY3_00001 ALI34215 Response regulator SaeR [Candidatus Nitroc... CDD UniProt Pfam InterPro
NMY3_00010 ALI34224 C4-dicarboxylate transport transcriptional... CDD UniProt Pfam InterPro
NMY3_00095 ALI34309 Blue-light-activated protein [Candidatus N... CDD UniProt Pfam InterPro
NMY3_00311 ALI34525 Alkaline phosphatase synthesis transcripti... CDD UniProt Pfam InterPro
NMY3_00699 ALI34908 hypothetical protein NMY3_00699 [Candidatu... CDD UniProt Pfam InterPro
NMY3_00764 ALI34973 Response regulator MprA [Candidatus Nitroc... CDD UniProt Pfam InterPro
NMY3_00922 ALI35129 Response regulator ArlR [Candidatus Nitroc... CDD UniProt Pfam InterPro
NMY3_01534 ALI35737 Alkaline phosphatase synthesis transcripti... CDD UniProt Pfam InterPro
NMY3_01702 ALI35905 Sporulation initiation phosphotransferase ... CDD UniProt Pfam InterPro
NMY3_01845 ALI36048 Response regulator receiver domain protein... CDD UniProt Pfam InterPro
NMY3_02193 ALI36393 Sensory transduction protein regX3 [Candid... CDD UniProt Pfam InterPro
NMY3_02760 ALI36950 DNA-binding response regulator MtrA [Candi... CDD UniProt Pfam InterPro
NMY3_02970 ALI37157 Sensor histidine kinase RcsC [Candidatus N... CDD UniProt Pfam InterPro
NMY3_03013 ALI37200 C4-dicarboxylate transport transcriptional... CDD UniProt Pfam InterPro
NMY3_03255 ALI37440 Transcriptional regulatory protein ZraR [C... CDD UniProt Pfam InterPro
NMY3_03566 ALI37748 Chemotaxis protein CheY [Candidatus Nitroc... CDD UniProt Pfam InterPro

ACyc-REC

NMY3_00724 ALI34933 C4-dicarboxylate transport transcriptional... CDD UniProt Pfam InterPro
NMY3_00765 ALI34974 Transcriptional regulatory protein ZraR [C... CDD UniProt Pfam InterPro
NMY3_00775 ALI34984 C4-dicarboxylate transport transcriptional... CDD UniProt Pfam InterPro
NMY3_03427 ALI37610 Transcriptional regulatory protein CssR [C... CDD UniProt Pfam InterPro

REC-NTPase

NMY3_02059 ALI36260 hypothetical protein NMY3_02059 [Candidatu... CDD UniProt Pfam InterPro

Other RRs

NMY3_00895 ALI35104 Response regulator receiver domain protein... CDD UniProt Pfam InterPro (no archaeal hits)
NMY3_01832 ALI36035 Cell cycle response regulator CtrA [Candid... CDD UniProt Pfam InterPro (no archaeal hits)
NMY3_03599 ALI37781 Response regulator receiver domain protein... CDD UniProt Pfam InterPro (no archaeal hits)

Candidatus Nitrosoarchaeum limnia SFB1

HisK, no REC

Nlim_0130 EGG42944 Signal transduction histidine kinase [Cand... CDD UniProt Pfam InterPro
Nlim_0172 EGG42942 Signal transduction histidine kinase [Cand... CDD UniProt Pfam InterPro
Nlim_0457 EGG42645 Signal transduction histidine kinase [Cand... CDD UniProt Pfam InterPro
Nlim_0468 EGG42656 Signal transduction histidine kinase [Cand... CDD UniProt Pfam InterPro
Nlim_0477 EGG42665 Chemotaxis protein histidine kinase [Candi... CDD UniProt Pfam InterPro
Nlim_0502 EGG42617 Signal transduction histidine kinase [Cand... CDD UniProt Pfam InterPro
Nlim_0503 EGG42618 Signal transduction histidine kinase [Cand... CDD UniProt Pfam InterPro
Nlim_0706 EGG42525 Signal transduction histidine kinase [Cand... CDD UniProt Pfam InterPro
Nlim_0741 EGG42560 Signal transduction histidine kinase [Cand... CDD UniProt Pfam InterPro
Nlim_1060 EGG42045 Signal transduction histidine kinase [Cand... CDD UniProt Pfam InterPro
Nlim_1130 EGG42005 histidine kinase [Candidatus Nitrosoarchae... CDD UniProt Pfam InterPro

Nlim_1227	EGG41993	Signal transduction histidine kinase [Cand...	CDD	UniProt	Pfam	InterPro	
Nlim_1312	EGG41788	histidine kinase [Candidatus Nitrosoarchae...	CDD	UniProt	Pfam	InterPro	
Nlim_1675	EGG41515	Signal transduction histidine kinase [Cand...	CDD	UniProt	Pfam	InterPro	
Nlim_1676	EGG41516	Signal transduction histidine kinase [Cand...	CDD	UniProt	Pfam	InterPro	
Nlim_1743	EGG41451	histidine kinase [Candidatus Nitrosoarchae...	CDD	UniProt	Pfam	InterPro	
**REC only**							
Nlim_0096	EGG43046	CheY-like receiver [Candidatus Nitrosoarch...	CDD	UniProt	Pfam	InterPro	
Nlim_0228	EGG42918	response regulator receiver protein [Candi...	CDD	UniProt	Pfam	InterPro	
Nlim_0417	EGG42704	CheY-like receiver [Candidatus Nitrosoarch...	CDD	UniProt	Pfam	InterPro	
Nlim_0466	EGG42654	CheY-like receiver [Candidatus Nitrosoarch...	CDD	UniProt	Pfam	InterPro	
Nlim_0467	EGG42655	CheY-like receiver [Candidatus Nitrosoarch...	CDD	UniProt	Pfam	InterPro	
Nlim_0469	EGG42657	CheY-like receiver [Candidatus Nitrosoarch...	CDD	UniProt	Pfam	InterPro	
Nlim_0472	EGG42660	CheY-like receiver [Candidatus Nitrosoarch...	CDD	UniProt	Pfam	InterPro	
Nlim_0499	EGG42614	CheY-like receiver [Candidatus Nitrosoarch...	CDD	UniProt	Pfam	InterPro	
Nlim_0739	EGG42558	CheY-like receiver [Candidatus Nitrosoarch...	CDD	UniProt	Pfam	InterPro	
Nlim_0779	EGG42334	CheY-like receiver [Candidatus Nitrosoarch...	CDD	UniProt	Pfam	InterPro	
Nlim_0780	EGG42335	CheY-like receiver [Candidatus Nitrosoarch...	CDD	UniProt	Pfam	InterPro	
Nlim_0781	EGG42336	CheY-like receiver [Candidatus Nitrosoarch...	CDD	UniProt	Pfam	InterPro	
Nlim_0783	EGG42338	CheY-like receiver [Candidatus Nitrosoarch...	CDD	UniProt	Pfam	InterPro	
Nlim_0784	EGG42339	CheY-like receiver [Candidatus Nitrosoarch...	CDD	UniProt	Pfam	InterPro	
Nlim_0785	EGG42340	response regulator receiver protein [Candi...	CDD	UniProt	Pfam	InterPro	
Nlim_0839	EGG42193	Hypothetical protein Nlim_0839 [Candidatus...	CDD	UniProt	Pfam	InterPro	
Nlim_0841	EGG42195	CheY-like receiver [Candidatus Nitrosoarch...	CDD	UniProt	Pfam	InterPro	
Nlim_0842	EGG42196	CheY-like receiver [Candidatus Nitrosoarch...	CDD	UniProt	Pfam	InterPro	
Nlim_0847	EGG42201	Chemotaxis response regulator [Candidatus ...	CDD	UniProt	Pfam	InterPro	
Nlim_0862	EGG42216	CheY-like receiver [Candidatus Nitrosoarch...	CDD	UniProt	Pfam	InterPro	
Nlim_0894	EGG42248	Hypothetical protein Nlim_0894 [Candidatus...	CDD	UniProt	Pfam	InterPro	
Nlim_0912	EGG42266	CheY-like receiver [Candidatus Nitrosoarch...	CDD	UniProt	Pfam	InterPro	
Nlim_1129	EGG42004	response regulator receiver protein [Candi...	CDD	UniProt	Pfam	InterPro	
Nlim_1313	EGG41789	CheY-like receiver [Candidatus Nitrosoarch...	CDD	UniProt	Pfam	InterPro	
Nlim_1378	EGG41761	response regulator receiver protein [Candi...	CDD	UniProt	Pfam	InterPro	
Nlim_1744	EGG41452	response regulator receiver protein [Candi...	CDD	UniProt	Pfam	InterPro	
*Nlim_0460	EGG42648	Hypothetical protein Nlim_0460 [Candidatus...	CDD	UniProt	Pfam	InterPro	
*Nlim_0838	EGG42192	Hypothetical protein Nlim_0838 [Candidatus...	CDD	UniProt	Pfam	InterPro	
**TrmB-REC**							
Nlim_0171	EGG42941	response regulator receiver protein [Candi...	CDD	UniProt	Pfam	InterPro	
**REC-CheB**							
Nlim_0478	EGG42666	Chemotaxis response regulator [Candidatus ...	CDD	UniProt	Pfam	InterPro	
**REC-HisK**							
Nlim_0782	EGG42337	Signal transduction histidine kinase [Cand...	CDD	UniProt	Pfam	InterPro	
**TPR-REC**							
Nlim_0076	EGG43026	Hypothetical protein Nlim_0076 [Candidatus...	CDD	UniProt	Pfam	InterPro	(TPR-TPR-TPR-REC)
**Other RRs**							
Nlim_0427	EGG42714	CheY-like receiver [Candidatus Nitrosoarch...	CDD	UniProt	Pfam	InterPro	(REC-TackOD1)

Candidatus Nitrosopumilus adriaticus

**HisK, no REC**						
NADRNF5_0257	AJW69956	PAS/PAC sensor signal transduction histi...	CDD	UniProt	Pfam	InterPro
NADRNF5_0796	AJW70490	ATPase/histidine kinase/DNA gyrase B/HSP...	CDD	UniProt	Pfam	InterPro
NADRNF5_0803	AJW70497	chemotaxis sensor histidine kinase CheA ...	CDD	UniProt	Pfam	InterPro
NADRNF5_0830	AJW70524	Signal transduction histidine kinase [Ca...	CDD	UniProt	Pfam	InterPro
NADRNF5_0838	AJW70532	ATPase/histidine kinase/DNA gyrase B/HSP...	CDD	UniProt	Pfam	InterPro
NADRNF5_0876	AJW70570	ATPase/histidine kinase/DNA gyrase B/HSP...	CDD	UniProt	Pfam	InterPro
NADRNF5_0915	AJW70609	Signal transduction histidine kinase [Ca...	CDD	UniProt	Pfam	InterPro
NADRNF5_0932	AJW70626	PAS domain S-box [Candidatus Nitrosopumi...	CDD	UniProt	Pfam	InterPro
NADRNF5_1098	AJW70788	Histidine kinase [Candidatus Nitrosopumi...	CDD	UniProt	Pfam	InterPro
NADRNF5_1201	AJW70889	ATPase/histidine kinase/DNA gyrase B/HSP...	CDD	UniProt	Pfam	InterPro
NADRNF5_1294	AJW70980	putative Histidine kinase [Candidatus Ni...	CDD	UniProt	Pfam	InterPro
NADRNF5_1555	AJW71236	Signal transduction histidine kinase [Ca...	CDD	UniProt	Pfam	InterPro
NADRNF5_1608	AJW71289	Sensor protein [Candidatus Nitrosopumilu...	CDD	UniProt	Pfam	InterPro
NADRNF5_1610	AJW71291	putative Histidine kinase [Candidatus Ni...	CDD	UniProt	Pfam	InterPro
NADRNF5_1612	AJW71293	Histidine kinase [Candidatus Nitrosopumi...	CDD	UniProt	Pfam	InterPro
NADRNF5_1796	AJW71474	Histidine kinase [Candidatus Nitrosopumi...	CDD	UniProt	Pfam	InterPro
NADRNF5_1806	AJW71484	Histidine kinase [Candidatus Nitrosopumi...	CDD	UniProt	Pfam	InterPro
NADRNF5_1813	AJW71491	ATPase/histidine kinase/DNA gyrase B/HSP...	CDD	UniProt	Pfam	InterPro
NADRNF5_1815	AJW71493	putative Histidine kinase [Candidatus Ni...	CDD	UniProt	Pfam	InterPro
NADRNF5_1875	AJW71553	putative Histidine kinase [Candidatus Ni...	CDD	UniProt	Pfam	InterPro
NADRNF5_2062	AJW71736	Histidine kinase [Candidatus Nitrosopumi...	CDD	UniProt	Pfam	InterPro
NADRNF5_1631	AJW71311	Sensor protein ZraS [Candidatus Nitrosop...	CDD	UniProt	Pfam	InterPro
*NADRNF5_1632	AJW71312	membrane protein of unknown function [Ca...	CDD	UniProt	Pfam	InterPro
**REC only**						
NADRNF5_0878	AJW70572	putative chemotaxis response regulator r...	CDD	UniProt	Pfam	InterPro
NADRNF5_0520	AJW70216	putative chemotaxis response regulator r...	CDD	UniProt	Pfam	InterPro
NADRNF5_0800	AJW70494	putative chemotaxis response regulator r...	CDD	UniProt	Pfam	InterPro
NADRNF5_0760	AJW70454	putative chemotaxis response regulator r...	CDD	UniProt	Pfam	InterPro
NADRNF5_2087	AJW71760	putative chemotaxis response regulator r...	CDD	UniProt	Pfam	InterPro
NADRNF5_2089	AJW71762	putative chemotaxis response regulator r...	CDD	UniProt	Pfam	InterPro
NADRNF5_2061	AJW71735	Response regulator receiver domain prote...	CDD	UniProt	Pfam	InterPro

```
NADRNF5_1807  AJW71485 Response regulator receiver protein [Can...    CDD   UniProt   Pfam   InterPro
NADRNF5_1798  AJW71476 Response regulator receiver protein [Can...    CDD   UniProt   Pfam   InterPro
NADRNF5_1840  AJW71518 Response regulator receiver protein [Can...    CDD   UniProt   Pfam   InterPro
NADRNF5_0264  AJW69962 Response regulator receiver protein [Can...    CDD   UniProt   Pfam   InterPro
NADRNF5_0795  AJW70489 putative chemotaxis response regulator r...    CDD   UniProt   Pfam   InterPro
NADRNF5_1039  AJW70730 Response regulator receiver protein [Can...    CDD   UniProt   Pfam   InterPro
NADRNF5_0825  AJW70519 putative chemotaxis response regulator r...    CDD   UniProt   Pfam   InterPro
NADRNF5_0797  AJW70491 putative chemotaxis response regulator r...    CDD   UniProt   Pfam   InterPro
NADRNF5_0694  AJW70389 Response regulator receiver domain prote...    CDD   UniProt   Pfam   InterPro
NADRNF5_1404  AJW71089 putative chemotaxis response regulator r...    CDD   UniProt   Pfam   InterPro
NADRNF5_0397  AJW70093 hypothetical protein NADRNF5_0397 [Candi...    CDD   UniProt   Pfam   InterPro
NADRNF5_0794  AJW70488 putative chemotaxis response regulator r...    CDD   UniProt   Pfam   InterPro
NADRNF5_1609  AJW71290 Response regulator receiver protein [Can...    CDD   UniProt   Pfam   InterPro
NADRNF5_1887  AJW71565 putative chemotaxis response regulator r...    CDD   UniProt   Pfam   InterPro
NADRNF5_0258  AJW69957 response regulator receiver protein [Can...    CDD   UniProt   Pfam   InterPro
NADRNF5_0844  AJW70538 putative chemotaxis response regulator r...    CDD   UniProt   Pfam   InterPro
NADRNF5_0831  AJW70525 putative chemotaxis response regulator r...    CDD   UniProt   Pfam   InterPro
NADRNF5_1402  AJW71087 Response regulator receiver protein (mod...    CDD   UniProt   Pfam   InterPro
NADRNF5_1099  AJW70789 Response regulator receiver protein [Can...    CDD   UniProt   Pfam   InterPro
NADRNF5_1293  AJW70979 putative Response regulator receiver pro...    CDD   UniProt   Pfam   InterPro
NADRNF5_2088  AJW71761 chemotaxis response regulator protein-gl...    CDD   UniProt   Pfam   InterPro
REC-CheB
NADRNF5_0804  AJW70498 chemotaxis response regulator protein-gl...    CDD   UniProt   Pfam   InterPro
TrmB-REC
NADRNF5_1556  AJW71237 Response regulator receiver protein [Can...    CDD   UniProt   Pfam   InterPro
TPR-REC
NADRNF5_1661  AJW71341 Response regulator receiver and ANTAR do...    CDD   UniProt   Pfam   InterPro (TPR-TPR-TPR-REC)
Other RRs
NADRNF5_1202  AJW70890 putative chemotaxis response regulator r...    CDD   UniProt   Pfam   InterPro (unique)
NADRNF5_1240  AJW70928 Response regulator receiver domain prote...    CDD   UniProt   Pfam   InterPro (FeS domain)
NADRNF5_1333  AJW71019 Response regulator receiver domain prote...    CDD   UniProt   Pfam   InterPro (FeS domain)
```

Candidatus Nitrosopumilus koreensis AR1

```
HisK, no REC
NKOR_00745  AFS80067 histidine kinase [Candidatus Nitrosopumilu...    CDD   UniProt   Pfam   InterPro
NKOR_01745  AFS80255 histidine kinase [Candidatus Nitrosopumilu...    CDD   UniProt   Pfam   InterPro
NKOR_02260  AFS80352 histidine kinase [Candidatus Nitrosopumilu...    CDD   UniProt   Pfam   InterPro
NKOR_03025  AFS80499 PAS/PAC sensor signal transduction histidi...    CDD   UniProt   Pfam   InterPro
NKOR_04865  AFS80859 PAS/PAC sensor signal transduction histidi...    CDD   UniProt   Pfam   InterPro
NKOR_05185  AFS80923 histidine kinase [Candidatus Nitrosopumilu...    CDD   UniProt   Pfam   InterPro
NKOR_05930  AFS81069 histidine kinase [Candidatus Nitrosopumilu...    CDD   UniProt   Pfam   InterPro
*NKOR_05935 AFS81070 hypothetical protein NKOR_05935 [Candidatu...    CDD   UniProt   Pfam   InterPro
NKOR_05970  AFS81077 PAS/PAC sensor signal transduction histidi...    CDD   UniProt   Pfam   InterPro
NKOR_06145  AFS81110 histidine kinase [Candidatus Nitrosopumilu...    CDD   UniProt   Pfam   InterPro
NKOR_06360  AFS81151 integral membrane sensor signal transducti...    CDD   UniProt   Pfam   InterPro
NKOR_07465  AFS81354 PAS/PAC sensor signal transduction histidi...    CDD   UniProt   Pfam   InterPro
NKOR_08145  AFS81490 histidine kinase [Candidatus Nitrosopumilu...    CDD   UniProt   Pfam   InterPro
REC only
NKOR_00740  AFS80066 response regulator receiver protein [Candi...    CDD   UniProt   Pfam   InterPro
NKOR_01775  AFS80261 response regulator receiver protein [Candi...    CDD   UniProt   Pfam   InterPro
NKOR_01875  AFS80281 response regulator receiver protein [Candi...    CDD   UniProt   Pfam   InterPro
NKOR_02265  AFS80353 response regulator receiver protein [Candi...    CDD   UniProt   Pfam   InterPro
NKOR_03020  AFS80498 response regulator receiver modulated digu...    CDD   UniProt   Pfam   InterPro
NKOR_04860  AFS80858 protein MprA [Candidatus Nitrosopumilus ko...    CDD   UniProt   Pfam   InterPro
NKOR_05945  AFS81072 response regulator receiver protein [Candi...    CDD   UniProt   Pfam   InterPro
NKOR_05965  AFS81076 response regulator [Candidatus Nitrosopumi...    CDD   UniProt   Pfam   InterPro
NKOR_06350  AFS81149 two-component response regulator involved ...    CDD   UniProt   Pfam   InterPro
NKOR_07460  AFS81353 response regulator receiver protein [Candi...    CDD   UniProt   Pfam   InterPro
NKOR_08150  AFS81491 response regulator receiver protein [Candi...    CDD   UniProt   Pfam   InterPro
NKOR_08810  AFS81613 response regulator receiver protein [Candi...    CDD   UniProt   Pfam   InterPro
TPR-REC
NKOR_02705  AFS80435 response regulator receiver and ANTAR doma...    CDD   UniProt   Pfam   InterPro
Other RRs
NKOR_04960  AFS80878 histidine kinase [Candidatus Nitrosopumilu...    CDD   UniProt   Pfam   InterPro (NitrOD4-REC)
NKOR_04565  AFS80801 hypothetical protein NKOR_04565 [Candidatu...    CDD   UniProt   Pfam   InterPro (REC-TackOD1)
```

Candidatus Nitrosopumilus piranensis

```
HisK, no REC
NPIRD3C_1703  AJM92915 putative Histidine kinase [Candidatus Ni...    CDD   UniProt   Pfam   InterPro
NPIRD3C_1833  AJM93043 putative Histidine kinase [Candidatus Ni...    CDD   UniProt   Pfam   InterPro
REC only
NPIRD3C_1061  AJM92273 putative chemotaxis response regulator r...    CDD   UniProt   Pfam   InterPro
*NPIRD3C_0946 AJM92158 Sensor histidine kinase/response regulat...    CDD   UniProt   Pfam   InterPro
Other RRs
NPIRD3C_1834  AJM93044 hypothetical protein NPIRD3C_1834 [Candi...    CDD   UniProt   Pfam   InterPro (no archaeal
hits)
NPIRD3C_0940  AJM92152 conserved membrane protein of unknown fu...    CDD   UniProt   Pfam   InterPro (RhaT/EamA-REC)
```

Candidatus Nitrosopumilus sediminis

HisK, no REC

NSED_00425	AFS81897	histidine kinase [Candidatus Nitrosopumilu...	CDD	UniProt	Pfam	InterPro	
NSED_04350	AFS82676	histidine kinase [Candidatus Nitrosopumilu...	CDD	UniProt	Pfam	InterPro	
NSED_04820	AFS82768	integral membrane sensor signal transducti...	CDD	UniProt	Pfam	InterPro	
NSED_05770	AFS82956	histidine kinase [Candidatus Nitrosopumilu...	CDD	UniProt	Pfam	InterPro	
NSED_06180	AFS83038	integral membrane sensor signal transducti...	CDD	UniProt	Pfam	InterPro	
NSED_06525	AFS83105	histidine kinase [Candidatus Nitrosopumilu...	CDD	UniProt	Pfam	InterPro	

REC only

NSED_00420	AFS81896	two component sigma54 specific Fis family ...	CDD	UniProt	Pfam	InterPro	
NSED_04815	AFS82767	response regulator receiver protein [Candi...	CDD	UniProt	Pfam	InterPro	
NSED_05350	AFS82874	response regulator receiver protein [Candi...	CDD	UniProt	Pfam	InterPro	
NSED_06170	AFS83036	response regulator receiver protein [Candi...	CDD	UniProt	Pfam	InterPro	
NSED_08760	AFS83544	response regulator receiver protein [Candi...	CDD	UniProt	Pfam	InterPro	
*NSED_00495	AFS81911	response regulator receiver protein [Candi...	CDD	UniProt	Pfam	InterPro	

TPR-REC

NSED_02635	AFS82335	two-component response regulator KdpE [Can...	CDD	UniProt	Pfam	InterPro	(TPR-TPR-TPR-REC)

Other RRs

NSED_03545	AFS82515	response regulator receiver protein [Candi...	CDD	UniProt	Pfam	InterPro	(REC-NitrOD1)
NSED_04940	AFS82792	response regulator receiver protein [Candi...	CDD	UniProt	Pfam	InterPro	(REC-NitrOD1)
NSED_05360	AFS82876	response regulator receiver protein [Candi...	CDD	UniProt	Pfam	InterPro	(REC-NitrOD1)
NSED_05820	AFS82966	response regulator receiver protein [Candi...	CDD	UniProt	Pfam	InterPro	(REC-NitrOD1)

Candidatus Nitrososphaera evergladensis SR1

HisK, no REC

NTE_00160	AIF82242	signal transduction histidine kinase [Cand...	CDD	UniProt	Pfam	InterPro	
NTE_00201	AIF82283	histidine kinase [Candidatus Nitrososphaer...	CDD	UniProt	Pfam	InterPro	
NTE_00233	AIF82315	signal transduction histidine kinase [Cand...	CDD	UniProt	Pfam	InterPro	
NTE_00338	AIF82420	histidine kinase [Candidatus Nitrososphaer...	CDD	UniProt	Pfam	InterPro	
NTE_00370	AIF82452	signal transduction histidine kinase [Cand...	CDD	UniProt	Pfam	InterPro	
NTE_00468	AIF82550	signal transduction histidine kinase [Cand...	CDD	UniProt	Pfam	InterPro	
NTE_00852	AIF82928	PAS domain S-box [Candidatus Nitrososphaer...	CDD	UniProt	Pfam	InterPro	
NTE_01052	AIF83125	histidine kinase [Candidatus Nitrososphaer...	CDD	UniProt	Pfam	InterPro	
NTE_01439	AIF83503	signal transduction histidine kinase [Cand...	CDD	UniProt	Pfam	InterPro	
NTE_01471	AIF83535	signal transduction histidine kinase [Cand...	CDD	UniProt	Pfam	InterPro	
NTE_01492	AIF83555	histidine kinase [Candidatus Nitrososphaer...	CDD	UniProt	Pfam	InterPro	
NTE_01496	AIF83559	signal transduction histidine kinase [Cand...	CDD	UniProt	Pfam	InterPro	
NTE_01820	AIF83880	signal transduction histidine kinase [Cand...	CDD	UniProt	Pfam	InterPro	
NTE_01927	AIF83986	signal transduction histidine kinase [Cand...	CDD	UniProt	Pfam	InterPro	
NTE_02123	AIF84178	signal transduction histidine kinase [Cand...	CDD	UniProt	Pfam	InterPro	
NTE_02155	AIF84209	signal transduction histidine kinase [Cand...	CDD	UniProt	Pfam	InterPro	
NTE_02334	AIF84387	signal transduction histidine kinase [Cand...	CDD	UniProt	Pfam	InterPro	
NTE_02348	AIF84400	chemotaxis protein histidine kinase-like p...	CDD	UniProt	Pfam	InterPro	
NTE_02940	AIF84976	PAS domain S-box [Candidatus Nitrososphaer...	CDD	UniProt	Pfam	InterPro	

REC only

NTE_00090	AIF82174	response regulator with CheY-like receiver...	CDD	UniProt	Pfam	InterPro	
NTE_00162	AIF82244	response regulator with CheY-like receiver...	CDD	UniProt	Pfam	InterPro	
NTE_00195	AIF82277	response regulator with CheY-like receiver...	CDD	UniProt	Pfam	InterPro	
NTE_00356	AIF82438	response regulator with CheY-like receiver...	CDD	UniProt	Pfam	InterPro	
NTE_00851	AIF82927	response regulator with CheY-like receiver...	CDD	UniProt	Pfam	InterPro	
NTE_01320	AIF83388	response regulator with CheY-like receiver...	CDD	UniProt	Pfam	InterPro	
NTE_01491	AIF83554	response regulator with CheY-like receiver...	CDD	UniProt	Pfam	InterPro	
NTE_01926	AIF83985	response regulator with CheY-like receiver...	CDD	UniProt	Pfam	InterPro	
NTE_02124	AIF84179	response regulator with CheY-like receiver...	CDD	UniProt	Pfam	InterPro	
NTE_02333	AIF84386	response regulator with CheY-like receiver...	CDD	UniProt	Pfam	InterPro	
NTE_02346	AIF84398	response regulator containing CheY-like re...	CDD	UniProt	Pfam	InterPro	
NTE_02941	AIF84977	response regulator with CheY-like receiver...	CDD	UniProt	Pfam	InterPro	
*NTE_00211	AIF82293	hypothetical protein NTE_00211 [Candidatus...	CDD	UniProt	Pfam	InterPro	

REC-CheB

NTE_02347	AIF84399	chemotaxis response regulator containing a...	CDD	UniProt	Pfam	InterPro	

ACyc-REC

NTE_01493	AIF83556	response regulator with CheY-like receiver...	CDD	UniProt	Pfam	InterPro	

wHTH-REC

NTE_01494	AIF83557	response regulator with CheY-like receiver...	CDD	UniProt	Pfam	InterPro	(wHTH-REC)

Other RRs

NTE_00171	AIF82253	response regulator containing CheY-like re...	CDD	UniProt	Pfam	InterPro	(REC-NitrOD2)
NTE_00257	AIF82339	response regulator with CheY-like receiver...	CDD	UniProt	Pfam	InterPro	(REC-TackOD1)
NTE_00367	AIF82449	response regulator with CheY-like receiver...	CDD	UniProt	Pfam	InterPro	(REC-NitrOD2)
NTE_01443	AIF83507	response regulator with CheY-like receiver...	CDD	UniProt	Pfam	InterPro	(NitrOD3-REC)

Candidatus Nitrososphaera gargensis Ga9.2

HisK, no REC

Ngar_c08540	AFU57796	putative signal transduction histidine ki...	CDD	UniProt	Pfam	InterPro
Ngar_c08680	AFU57810	signal transduction histidine kinase, wit...	CDD	UniProt	Pfam	InterPro
Ngar_c08700	AFU57812	signal transduction histidine kinase, wit...	CDD	UniProt	Pfam	InterPro

Ngar_c08930	AFU57835	signal transduction histidine kinase, wit...	CDD	UniProt	Pfam	InterPro	
Ngar_c09690	AFU57911	signal transduction histidine kinase, wit...	CDD	UniProt	Pfam	InterPro	
Ngar_c09970	AFU57939	putative signal transduction histidine ki...	CDD	UniProt	Pfam	InterPro	
Ngar_c10180	AFU57960	chemotactic sensor histidine kinase CheA ...	CDD	UniProt	Pfam	InterPro	
Ngar_c12530	AFU58193	putative signal transduction histidine ki...	CDD	UniProt	Pfam	InterPro	
Ngar_c21530	AFU59083	signal transduction histidine kinase, wit...	CDD	UniProt	Pfam	InterPro	
Ngar_c22010	AFU59131	signal transduction histidine kinase, wit...	CDD	UniProt	Pfam	InterPro	
Ngar_c22090	AFU59139	putative signal transduction histidine ki...	CDD	UniProt	Pfam	InterPro	
Ngar_c24800	AFU59402	signal transduction histidine kinase, wit...	CDD	UniProt	Pfam	InterPro	
Ngar_c24860	AFU59408	putative signal transduction histidine ki...	CDD	UniProt	Pfam	InterPro	
Ngar_c27670	AFU59688	signal transduction histidine kinase, wit...	CDD	UniProt	Pfam	InterPro	
Ngar_c28690	AFU59788	putative signal transduction histidine ki...	CDD	UniProt	Pfam	InterPro	
Ngar_c31260	AFU60042	putative signal transduction histidine ki...	CDD	UniProt	Pfam	InterPro	
Ngar_c32750	AFU60190	signal transduction histidine kinase, wit...	CDD	UniProt	Pfam	InterPro	
Ngar_c33320	AFU60247	putative signal transduction histidine ki...	CDD	UniProt	Pfam	InterPro	
Ngar_c33500	AFU60265	putative signal transduction histidine ki...	CDD	UniProt	Pfam	InterPro	
**REC only**							
Ngar_c00050	AFU56955	putative signal transduction response reg...	CDD	UniProt	Pfam	InterPro	
Ngar_c02520	AFU57200	putative signal transduction response reg...	CDD	UniProt	Pfam	InterPro	
Ngar_c02740	AFU57222	putative signal transduction response reg...	CDD	UniProt	Pfam	InterPro	
Ngar_c08410	AFU57783	putative signal transduction response reg...	CDD	UniProt	Pfam	InterPro	
Ngar_c08520	AFU57794	putative signal transduction response reg...	CDD	UniProt	Pfam	InterPro	
Ngar_c08880	AFU57830	putative two component transcriptional re...	CDD	UniProt	Pfam	InterPro	
Ngar_c10200	AFU57962	chemotaxis response regulator CheY [Candi...	CDD	UniProt	Pfam	InterPro	
Ngar_c10570	AFU57999	putative signal transduction response reg...	CDD	UniProt	Pfam	InterPro	
Ngar_c10640	AFU58006	putative signal transduction response reg...	CDD	UniProt	Pfam	InterPro	
Ngar_c13050	AFU58243	putative signal transduction response reg...	CDD	UniProt	Pfam	InterPro	
Ngar_c13380	AFU58276	putative signal transduction response reg...	CDD	UniProt	Pfam	InterPro	
Ngar_c14130	AFU58349	putative signal transduction response reg...	CDD	UniProt	Pfam	InterPro	
Ngar_c16500	AFU58583	response regulatory domain-containing pro...	CDD	UniProt	Pfam	InterPro	
Ngar_c22000	AFU59130	putative response regulator receiver prot...	CDD	UniProt	Pfam	InterPro	
Ngar_c22510	AFU59181	hypothetical protein Ngar_c22510 [Candida...	CDD	UniProt	Pfam	InterPro	
Ngar_c24870	AFU59409	putative signal transduction response reg...	CDD	UniProt	Pfam	InterPro	
Ngar_c25990	AFU59521	putative signal transduction response reg...	CDD	UniProt	Pfam	InterPro	
Ngar_c26590	AFU59580	putative signal transduction response reg...	CDD	UniProt	Pfam	InterPro	
Ngar_c27650	AFU59686	hypothetical protein Ngar_c27650 [Candida...	CDD	UniProt	Pfam	InterPro	
Ngar_c28680	AFU59787	hypothetical protein Ngar_c28680 [Candida...	CDD	UniProt	Pfam	InterPro	
Ngar_c28750	AFU59794	putative signal transduction response reg...	CDD	UniProt	Pfam	InterPro	
Ngar_c28870	AFU59806	putative signal transduction response reg...	CDD	UniProt	Pfam	InterPro	
Ngar_c28890	AFU59808	putative signal transduction response reg...	CDD	UniProt	Pfam	InterPro	
Ngar_c33330	AFU60248	putative signal transduction response reg...	CDD	UniProt	Pfam	InterPro	
Ngar_c33950	AFU60310	putative signal transduction response reg...	CDD	UniProt	Pfam	InterPro	
*Ngar_c04310	AFU57378	hypothetical protein Ngar_c04310 [Candida...	CDD	UniProt	Pfam	InterPro	
*Ngar_c04320	AFU57379	signal receiver domain-containing protein...	CDD	UniProt	Pfam	InterPro	
*Ngar_c08390	AFU57781	putative response regulator receiver prot...	CDD	UniProt	Pfam	InterPro	
*Ngar_c11730	AFU58113	putative short signal transduction respon...	CDD	UniProt	Pfam	InterPro	
*Ngar_c12540	AFU58194	putative short signal transduction respon...	CDD	UniProt	Pfam	InterPro	
*Ngar_c18900	AFU58822	response regulatory domain-containing pro...	CDD	UniProt	Pfam	InterPro	
**REC-CheB**							
Ngar_c10190	AFU57961	chemotaxis response regulator methylester...	CDD	UniProt	Pfam	InterPro	
**Other RRs**							
Ngar_c08720	AFU57814	putative signal transduction response reg...	CDD	UniProt	Pfam	InterPro	(NitrOD3-REC)
Ngar_c21520	AFU59082	putative signal transduction response reg...	CDD	UniProt	Pfam	InterPro	
Ngar_c21780	AFU59108	hypothetical protein Ngar_c21780 [Candida...	CDD	UniProt	Pfam	InterPro	(MEDS-REC)
Ngar_c27680	AFU59689	putative signal transduction response reg...	CDD	UniProt	Pfam	InterPro	(Ser-rich-REC)
Ngar_c33150	AFU60230	putative signal transduction response reg...	CDD	UniProt	Pfam	InterPro	(Ser-rich-REC)
*Ngar_c10040	AFU57946	putative response regulator receiver [Can...	CDD	UniProt	Pfam	InterPro	
*Ngar_c33200	AFU60235	putative response regulator receiver [Can...	CDD	UniProt	Pfam	InterPro	

Candidatus Nitrosotalea devanaterra

HisK, no REC

NDEV_0432	CUR51197	putative Histidine kinase [Candidatus Nitr...	CDD	UniProt	Pfam	InterPro
NDEV_0725	CUR51490	putative Histidine kinase [Candidatus Nitr...	CDD	UniProt	Pfam	InterPro
NDEV_0741	CUR51506	ATPase/histidine kinase/DNA gyrase B/HSP90...	CDD	UniProt	Pfam	InterPro
NDEV_0999	CUR51764	Chemotaxis histidine kinase CheA [Candidat...	CDD	UniProt	Pfam	InterPro
NDEV_1007	CUR51772	putative Histidine kinase [Candidatus Nitr...	CDD	UniProt	Pfam	InterPro
NDEV_1010	CUR51775	putative Histidine kinase [Candidatus Nitr...	CDD	UniProt	Pfam	InterPro
NDEV_1385	CUR52150	putative Histidine kinase [Candidatus Nitr...	CDD	UniProt	Pfam	InterPro
NDEV_1408	CUR52173	Histidine kinase [Candidatus Nitrosotalea ...	CDD	UniProt	Pfam	InterPro
NDEV_1419	CUR52184	PAS domain S-box (modular protein) [Candid...	CDD	UniProt	Pfam	InterPro
NDEV_1454	CUR52219	Putative PAS/PAC sensor histidine kinase [...	CDD	UniProt	Pfam	InterPro
NDEV_1455	CUR52220	K+ channel signal transduction histidine k...	CDD	UniProt	Pfam	InterPro
NDEV_1499	CUR52264	Signal transduction histidine kinase, with...	CDD	UniProt	Pfam	InterPro
NDEV_1508	CUR52273	putative Histidine kinase [Candidatus Nitr...	CDD	UniProt	Pfam	InterPro
NDEV_1531	CUR52296	putative signal transduction histidine kin...	CDD	UniProt	Pfam	InterPro
NDEV_1535	CUR52300	putative Histidine kinase [Candidatus Nitr...	CDD	UniProt	Pfam	InterPro
NDEV_1571	CUR52333	putative Histidine kinase [Candidatus Nitr...	CDD	UniProt	Pfam	InterPro
NDEV_1706	CUR52468	Signal transduction histidine kinase [Cand...	CDD	UniProt	Pfam	InterPro

NDEV_1717 CUR52479 Signal transduction histidine kinase [Cand... CDD UniProt Pfam InterPro
NDEV_1773 CUR52535 putative Histidine kinase [Candidatus Nitr... CDD UniProt Pfam InterPro

REC only

NDEV_0249 CUR51014 CheY-like receiver [Candidatus Nitrosotale... CDD UniProt Pfam InterPro
NDEV_0433 CUR51198 CheY-like receiver [Candidatus Nitrosotale... CDD UniProt Pfam InterPro
NDEV_0649 CUR51414 CheY-like receiver [Candidatus Nitrosotale... CDD UniProt Pfam InterPro
NDEV_0739 CUR51504 Chemotaxis protein CheY [Candidatus Nitros... CDD UniProt Pfam InterPro
NDEV_0981 CUR51746 CheY-like receiver [Candidatus Nitrosotale... CDD UniProt Pfam InterPro
NDEV_0997 CUR51762 Chemotaxis response regulator CheY [Candid... CDD UniProt Pfam InterPro
NDEV_1002 CUR51767 Protein CcdB [Candidatus Nitrosotalea deva... CDD UniProt Pfam InterPro
NDEV_1009 CUR51774 Putative chemotaxis response regulator Che... CDD UniProt Pfam InterPro
NDEV_1011 CUR51776 CheY-like receiver [Candidatus Nitrosotale... CDD UniProt Pfam InterPro
NDEV_1012 CUR51777 CheY-like receiver [Candidatus Nitrosotale... CDD UniProt Pfam InterPro
NDEV_1015 CUR51780 CheY-like receiver [Candidatus Nitrosotale... CDD UniProt Pfam InterPro
NDEV_1076 CUR51841 CheY-like receiver [Candidatus Nitrosotale... CDD UniProt Pfam InterPro
NDEV_1395 CUR52160 CheY-like receiver [Candidatus Nitrosotale... CDD UniProt Pfam InterPro
NDEV_1422 CUR52187 CheY-like receiver [Candidatus Nitrosotale... CDD UniProt Pfam InterPro
NDEV_1457 CUR52222 K+ channel signal transduction response re... CDD UniProt Pfam InterPro
NDEV_1500 CUR52265 putative signal transduction response regu... CDD UniProt Pfam InterPro

REC-CheB

NDEV_0998 CUR51763 Chemotaxis response regulator protein-glut... CDD UniProt Pfam InterPro

TPR-REC

NDEV_0516 CUR51281 conserved protein of unknown function [Can... CDD UniProt Pfam InterPro (TPR-TPR-TPR-REC)

Other RRs

NDEV_0738 CUR51503 conserved protein of unknown function [Can... CDD UniProt Pfam InterPro (REC-TackOD1)

Candidatus Nitrosotenuis cloacae strain SAT1

HisK, no REC

SU86_000030 AJZ76515 hypothetical protein SU86_000030 [Candida... CDD UniProt Pfam InterPro
SU86_000945 AJZ75188 histidine kinase [Candidatus Nitrosotenui... CDD UniProt Pfam InterPro
SU86_001160 AJZ75220 hypothetical protein SU86_001160 [Candida... CDD UniProt Pfam InterPro
SU86_001735 AJZ76557 hypothetical protein SU86_001735 [Candida... CDD UniProt Pfam InterPro
SU86_001905 AJZ76565 hypothetical protein SU86_001905 [Candida... CDD UniProt Pfam InterPro
SU86_008670 AJZ76744 hypothetical protein SU86_008670 [Candida... CDD UniProt Pfam InterPro

REC only

SU86_002750 AJZ76586 hypothetical protein SU86_002750 [Candida... CDD UniProt Pfam InterPro
SU86_005490 AJZ75909 chemotaxis protein CheY [Candidatus Nitro... CDD UniProt Pfam InterPro
SU86_002825 AJZ75489 hypothetical protein SU86_002825 [Candida... CDD UniProt Pfam InterPro
SU86_002745 AJZ75477 chemotaxis protein CheY [Candidatus Nitro... CDD UniProt Pfam InterPro
SU86_002475 AJZ75439 hypothetical protein SU86_002475 [Candida... CDD UniProt Pfam InterPro
SU86_001625 AJZ75300 hypothetical protein SU86_001625 [Candida... CDD UniProt Pfam InterPro
SU86_001485 AJZ75276 chemotaxis protein CheY [Candidatus Nitro... CDD UniProt Pfam InterPro
SU86_000955 AJZ75189 chemotaxis protein CheY [Candidatus Nitro... CDD UniProt Pfam InterPro
SU86_000455 AJZ75113 histidine kinase [Candidatus Nitrosotenui... CDD UniProt Pfam InterPro
SU86_000325 AJZ75092 hypothetical protein SU86_000325 [Candida... CDD UniProt Pfam InterPro
SU86_001310 AJZ75247 hypothetical protein SU86_001310 [Candida... CDD UniProt Pfam InterPro
SU86_002590 AJZ75453 hypothetical protein SU86_002590 [Candida... CDD UniProt Pfam InterPro

REC-CheB

SU86_005520 AJZ76668 chemotaxis protein [Candidatus Nitrosoten... CDD UniProt Pfam InterPro

TPR-REC

SU86_003460 AJZ75585 hypothetical protein SU86_003460 [Candida... CDD UniProt Pfam InterPro (TPR-TPR-TPR-REC)

Other RRs

SU86_000960 AJZ75190 hypothetical protein SU86_000960 [Candida... CDD UniProt Pfam InterPro (REC-TackOD1)

Nitrosopumilus maritimus SCM1

HisK, no REC

Nmar_0189 ABX12085 histidine kinase [Nitrosopumilus maritimus... CDD UniProt Pfam InterPro
Nmar_0249 ABX12145 histidine kinase [Nitrosopumilus maritimus... CDD UniProt Pfam InterPro
Nmar_0372 ABX12268 histidine kinase [Nitrosopumilus maritimus... CDD UniProt Pfam InterPro
Nmar_0460 ABX12356 histidine kinase [Nitrosopumilus maritimus... CDD UniProt Pfam InterPro
Nmar_0556 ABX12452 histidine kinase [Nitrosopumilus maritimus... CDD UniProt Pfam InterPro
Nmar_0656 ABX12552 histidine kinase [Nitrosopumilus maritimus... CDD UniProt Pfam InterPro
Nmar_0920 ABX12816 histidine kinase [Nitrosopumilus maritimus... CDD UniProt Pfam InterPro
Nmar_1076 ABX12972 putative sensor with HAMP domain [Nitrosop... CDD UniProt Pfam InterPro
Nmar_1207 ABX13103 histidine kinase [Nitrosopumilus maritimus... CDD UniProt Pfam InterPro
Nmar_1247 ABX13143 histidine kinase [Nitrosopumilus maritimus... CDD UniProt Pfam InterPro
Nmar_1299 ABX13195 PAS/PAC sensor signal transduction histidi... CDD UniProt Pfam InterPro
Nmar_1440 ABX13336 histidine kinase [Nitrosopumilus maritimus... CDD UniProt Pfam InterPro

REC only

Nmar_0251 ABX12147 response regulator receiver protein [Nitro... CDD UniProt Pfam InterPro
Nmar_0376 ABX12272 response regulator receiver protein [Nitro... CDD UniProt Pfam InterPro
Nmar_0452 ABX12348 response regulator receiver protein [Nitro... CDD UniProt Pfam InterPro
Nmar_0465 ABX12361 response regulator receiver protein [Nitro... CDD UniProt Pfam InterPro
Nmar_1045 ABX12941 response regulator receiver protein [Nitro... CDD UniProt Pfam InterPro
Nmar_1202 ABX13098 response regulator receiver protein [Nitro... CDD UniProt Pfam InterPro
Nmar_1206 ABX13102 response regulator receiver protein [Nitro... CDD UniProt Pfam InterPro
Nmar_1248 ABX13144 response regulator receiver protein [Nitro... CDD UniProt Pfam InterPro

Nmar_1619	ABX13515	response regulator receiver protein [Nitro...	CDD	UniProt	Pfam	InterPro	
**Other RRs**							
Nmar_0707	ABX12603	response regulator receiver protein [Nitro...	CDD	UniProt	Pfam	InterPro	(REC-NitrOD5)

Nitrososphaera viennensis EN76

**HisK, no REC**							
NVIE_001040	AIC14285	putative membrane associated signal trans...	CDD	UniProt	Pfam	InterPro	
NVIE_001570	AIC14342	putative membrane associated signal trans...	CDD	UniProt	Pfam	InterPro	
NVIE_005590	AIC14759	putative membrane associated signal trans...	CDD	UniProt	Pfam	InterPro	
NVIE_007400	AIC14950	putative membrane associated signal trans...	CDD	UniProt	Pfam	InterPro	
NVIE_009870	AIC15214	chemotactic sensor histidine kinase CheA ...	CDD	UniProt	Pfam	InterPro	
NVIE_015250	AIC15771	putative signal transduction histidine ki...	CDD	UniProt	Pfam	InterPro	
NVIE_021310	AIC16393	putative membrane associated signal trans...	CDD	UniProt	Pfam	InterPro	
NVIE_021340	AIC16396	putative membrane associated signal trans...	CDD	UniProt	Pfam	InterPro	
NVIE_026350	AIC16904	putative membrane associated signal trans...	CDD	UniProt	Pfam	InterPro	
NVIE_030060	AIC17283	putative signal transduction histidine ki...	CDD	UniProt	Pfam	InterPro	
**REC only**							
NVIE_015260	AIC15772	putative signal transduction response reg...	CDD	UniProt	Pfam	InterPro	
NVIE_005580	AIC14758	putative CheY-like signal transduction re...	CDD	UniProt	Pfam	InterPro	
NVIE_007410	AIC14952	putative signal transduction response reg...	CDD	UniProt	Pfam	InterPro	
NVIE_026340	AIC16903	putative signal transduction response reg...	CDD	UniProt	Pfam	InterPro	
NVIE_009850	AIC15212	chemotaxis response regulator CheY [Nitro...	CDD	UniProt	Pfam	InterPro	
NVIE_021320	AIC16394	putative Polar-differentiation response r...	CDD	UniProt	Pfam	InterPro	
NVIE_021330	AIC16395	putative signal transduction response reg...	CDD	UniProt	Pfam	InterPro	
NVIE_020450	AIC16303	putative signal transduction response reg...	CDD	UniProt	Pfam	InterPro	
**REC-CheB**							
NVIE_009860	AIC15213	Chemotaxis response regulator protein [Ni...	CDD	UniProt	Pfam	InterPro	
**wHTH-REC**							
NVIE_030040	AIC17281	putative signal transduction response reg...	CDD	UniProt	Pfam	InterPro	(wHTH-REC)
**Other RRs**							
NVIE_0132	AIC14313	putative signal transduction response reg...	CDD	UniProt	Pfam	InterPro	(NitrOD3-REC)

Please send corrections, questions and comments to Michael Galperin
