# Supplementary material for: Phyletic Distribution and Lineage-Specific Domain Architectures of Archaeal Two-Component Signal Transduction Systems
Source: J Bacteriol. 2018 Mar 12;200(7):e00681-17. doi: 10.1128/JB.00681-17 (PMC5847659; doi:10.1128/JB.00681-17)
Supplement: Supplemental material [file JB.00681-17_zjb999094653s3.pdf]

Table S3. Histidine kinases and response regulators encoded in unfinished genomes of Archaea

Asgard group
Candidatus Lokiarchaeota

. Lokiarchaeum sp. GC14_75	65 HKs, 27 RRs
. Candidatus Lokiarchaeota archaeon CR_4	26 HKs, 14 RRs
**Candidatus Heimdallarchaeota**	
. Candidatus Heimdallarchaeota archaeon AB_125	7 HKs, 4 RRs
. Candidatus Heimdallarchaeota archaeon LC_2	15 HKs, 23 RRs
. Candidatus Heimdallarchaeota archaeon LC_3	31 HKs, 46 RRs
**Candidatus Odinarchaeota**	
. Candidatus Odinarchaeota archaeon LCB_4	0 hits
**Candidatus Thorarchaeota**	
. Candidatus Thorarchaeota archaeon AB_25	10 HKs, 6 RRs
. Candidatus Thorarchaeota archaeon SMTZ-45	4 HKs, 2 RRs
. Candidatus Thorarchaeota archaeon SMTZ1-45	8 HKs, 6 RRs
. Candidatus Thorarchaeota archaeon SMTZ1-83	14 HKs, 5 RRs
**DPANN group**	
**Candidatus Aenigmarchaeota**	
. Candidatus Aenigmarchaeota archaeon CG1_02_38_14	1 HK, 3 RRs
**Candidatus Micrarchaeota**	
. Candidatus Micrarchaeota archaeon CG1_02_55_22	1 HK, 1 RR
. Candidatus Micrarchaeota archaeon RBG_16_49_10	1 HK, 1 RR
. Candidatus Micrarchaeum acidiphilum ARMAN-1	1 HK, 1 RR
. Candidatus Micrarchaeum acidiphilum ARMAN-2	2 HKs, 1 RR
. Candidatus Micrarchaeum sp. AZ1	0 HKs, 1 RR
**Candidatus Nanohaloarchaeota**	
. Candidatus Nanosalina sp. J07AB43	1 HK, 3 RRs
. Candidatus Nanosalinarum sp. J07AB56	0 HKs, 1 RRs
**Candidatus Pacearchaeota**	
. Candidatus Pacearchaeota archaeon RBG_13_36_9	3 HKs, 6 RRs
. Candidatus Pacearchaeota archaeon RBG_16_35_8	0 HKs, 1 RR
**Candidatus Woesearchaeota**	
. Candidatus Woesearchaeota archaeon CG1_02_57_44	3 HKs, 1 RR
**TACK group**	
**Candidatus Bathyarchaeota**	
. Candidatus Bathyarchaeota archaeon B26-1	0 HKs, 1 RR
. Candidatus Bathyarchaeota archaeon B26-2	0 HKs, 2 RRs
. Candidatus Bathyarchaeota archaeon B63	0 HKs, 1 RR
. Candidatus Bathyarchaeota archaeon RBG_13_38_9	1 HK, 7 RRs
. Candidatus Bathyarchaeota archaeon RBG_13_46_16b	3 HKs, 5 RRs
. Candidatus Bathyarchaeota archaeon RBG_13_52_12	4 HKs, 8 RRs
. Candidatus Bathyarchaeota archaeon RBG_13_60_20	1 HKs, 6 RRs
. Candidatus Bathyarchaeota archaeon RBG_16_48_13	1 HKs, 2 RRs
. Candidatus Bathyarchaeota archaeon RBG_16_57_9	2 HKs, 6 RRs
**unclassified Crenarchaeota**	
. Crenarchaeota archaeon 13_1_20CM_2_51_8	0 HKs, 6 RRs
. Crenarchaeota archaeon 13_1_20CM_2_53_14	1 HK, 1 RR
. miscellaneous Crenarchaeota group archaeon SMTZ-80	0 HKs, 4 RRs
. miscellaneous Crenarchaeota group archaeon SMTZ1-55	5 HKs, 3 RRs
. miscellaneous Crenarchaeota group-6 archaeon AD8-1	1 HK, 1 RR

Asgard group
Candidatus Lokiarchaeota
Lokiarchaeum sp. GC14_75

HisK, no REC

Lokiarch_00070	KKK46579	Signal transduction histidine kinase [...	CDD	UniProt	Pfam	InterPro
Lokiarch_00460	KKK46537	Signal transduction histidine kinase [...	CDD	UniProt	Pfam	InterPro
Lokiarch_00870	KKK46428	Signal transduction histidine kinase [...	CDD	UniProt	Pfam	InterPro
Lokiarch_01610	KKK46402	Signal transduction histidine kinase [...	CDD	UniProt	Pfam	InterPro
Lokiarch_01950	KKK46363	Signal transduction histidine kinase [...	CDD	UniProt	Pfam	InterPro
Lokiarch_02340	KKK46292	histidine kinase, partial [Lokiarchaeu...	CDD	UniProt	Pfam	InterPro
Lokiarch_02370	KKK46295	histidine kinase [Lokiarchaeum sp. GC1...	CDD	UniProt	Pfam	InterPro
Lokiarch_02430	KKK46301	Signal transduction histidine kinase [...	CDD	UniProt	Pfam	InterPro
Lokiarch_03510	KKK46181	Signal transduction histidine kinase [...	CDD	UniProt	Pfam	InterPro
Lokiarch_03590	KKK46173	Signal transduction histidine kinase [...	CDD	UniProt	Pfam	InterPro
Lokiarch_04650	KKK46050	Signal transduction histidine kinase, ...	CDD	UniProt	Pfam	InterPro
Lokiarch_05040	KKK46003	Signal transduction histidine kinase [...	CDD	UniProt	Pfam	InterPro
Lokiarch_05060	KKK46005	Signal transduction histidine kinase [...	CDD	UniProt	Pfam	InterPro
Lokiarch_05100	KKK46009	Signal transduction histidine kinase [...	CDD	UniProt	Pfam	InterPro
Lokiarch_05410	KKK45954	Signal transduction histidine kinase [...	CDD	UniProt	Pfam	InterPro
Lokiarch_05430	KKK45956	Signal transduction histidine kinase [...	CDD	UniProt	Pfam	InterPro
Lokiarch_07230	KKK45741	Signal transduction histidine kinase [...	CDD	UniProt	Pfam	InterPro
Lokiarch_07910	KKK45664	Signal transduction histidine kinase [...	CDD	UniProt	Pfam	InterPro

Lokiarch_08320	KKK45605	Signal transduction histidine kinase, ...	CDD	UniProt	Pfam	InterPro
Lokiarch_08370	KKK45609	Signal transduction histidine kinase [...	CDD	UniProt	Pfam	InterPro
Lokiarch_09070	KKK45532	Signal transduction histidine kinase [...	CDD	UniProt	Pfam	InterPro
Lokiarch_09590	KKK45479	Signal transduction histidine kinase [...	CDD	UniProt	Pfam	InterPro
Lokiarch_12330	KKK45149	Signal transduction histidine kinase [...	CDD	UniProt	Pfam	InterPro
Lokiarch_12590	KKK45111	Signal transduction histidine kinase [...	CDD	UniProt	Pfam	InterPro
Lokiarch_14070	KKK44935	Signal transduction histidine kinase [...	CDD	UniProt	Pfam	InterPro
Lokiarch_14080	KKK44936	Signal transduction histidine kinase, ...	CDD	UniProt	Pfam	InterPro
Lokiarch_15000	KKK44816	Signal transduction histidine kinase, ...	CDD	UniProt	Pfam	InterPro
Lokiarch_18040	KKK44457	Signal transduction histidine kinase, ...	CDD	UniProt	Pfam	InterPro
Lokiarch_18550	KKK44408	Signal transduction histidine kinase [...	CDD	UniProt	Pfam	InterPro
Lokiarch_18860	KKK44364	Signal transduction histidine kinase [...	CDD	UniProt	Pfam	InterPro
Lokiarch_19330	KKK44301	Signal transduction histidine kinase [...	CDD	UniProt	Pfam	InterPro
Lokiarch_20000	KKK44257	Signal transduction histidine kinase [...	CDD	UniProt	Pfam	InterPro
Lokiarch_22330	KKK43953	Signal transduction histidine kinase [...	CDD	UniProt	Pfam	InterPro
Lokiarch_23340	KKK43867	Signal transduction histidine kinase [...	CDD	UniProt	Pfam	InterPro
Lokiarch_23480	KKK43829	Signal transduction histidine kinase [...	CDD	UniProt	Pfam	InterPro
Lokiarch_23540	KKK43835	Signal transduction histidine kinase [...	CDD	UniProt	Pfam	InterPro
Lokiarch_23560	KKK43837	Signal transduction histidine kinase [...	CDD	UniProt	Pfam	InterPro
Lokiarch_25620	KKK43640	Signal transduction histidine kinase, ...	CDD	UniProt	Pfam	InterPro
Lokiarch_25750	KKK43591	Signal transduction histidine kinase [...	CDD	UniProt	Pfam	InterPro
Lokiarch_28080	KKK43304	Signal transduction histidine kinase, ...	CDD	UniProt	Pfam	InterPro
Lokiarch_28410	KKK43246	Signal transduction histidine kinase [...	CDD	UniProt	Pfam	InterPro
Lokiarch_28650	KKK43236	Signal transduction histidine kinase [...	CDD	UniProt	Pfam	InterPro
Lokiarch_28900	KKK43226	Signal transduction histidine kinase [...	CDD	UniProt	Pfam	InterPro
Lokiarch_29610	KKK43131	Signal transduction histidine kinase [...	CDD	UniProt	Pfam	InterPro
Lokiarch_31540	KKK42889	Signal transduction histidine kinase [...	CDD	UniProt	Pfam	InterPro
Lokiarch_32010	KKK42779	Signal transduction histidine kinase, ...	CDD	UniProt	Pfam	InterPro
Lokiarch_32380	KKK42815	Chemotaxis protein CheA [Lokiarchaeum ...	CDD	UniProt	Pfam	InterPro
Lokiarch_37990	KKK42045	Signal transduction histidine kinase [...	CDD	UniProt	Pfam	InterPro
Lokiarch_39210	KKK41896	Signal transduction histidine kinase [...	CDD	UniProt	Pfam	InterPro
Lokiarch_39440	KKK41869	Signal transduction histidine kinase [...	CDD	UniProt	Pfam	InterPro
Lokiarch_41490	KKK41609	Signal transduction histidine kinase [...	CDD	UniProt	Pfam	InterPro
Lokiarch_41560	KKK41616	Signal transduction histidine kinase [...	CDD	UniProt	Pfam	InterPro
Lokiarch_42190	KKK41530	Signal transduction histidine kinase [...	CDD	UniProt	Pfam	InterPro
Lokiarch_43180	KKK41384	Signal transduction histidine kinase, ...	CDD	UniProt	Pfam	InterPro
Lokiarch_43190	KKK41385	Signal transduction histidine kinase [...	CDD	UniProt	Pfam	InterPro
Lokiarch_43950	KKK41336	Signal transduction histidine kinase [...	CDD	UniProt	Pfam	InterPro
Lokiarch_45820	KKK41094	Signal transduction histidine kinase [...	CDD	UniProt	Pfam	InterPro
Lokiarch_45830	KKK41095	Signal transduction histidine kinase [...	CDD	UniProt	Pfam	InterPro
Lokiarch_48050	KKK40829	Signal transduction histidine kinase [...	CDD	UniProt	Pfam	InterPro
Lokiarch_49130	KKK40714	Signal transduction histidine kinase, ...	CDD	UniProt	Pfam	InterPro
Lokiarch_50120	KKK40577	Signal transduction histidine kinase [...	CDD	UniProt	Pfam	InterPro
Lokiarch_51420	KKK40441	Signal transduction histidine kinase [...	CDD	UniProt	Pfam	InterPro
Lokiarch_51540	KKK40437	Signal transduction histidine kinase [...	CDD	UniProt	Pfam	InterPro
Lokiarch_52410	KKK40347	Signal transduction histidine kinase [...	CDD	UniProt	Pfam	InterPro
Lokiarch_53490	KKK40193	Signal transduction histidine kinase [...	CDD	UniProt	Pfam	InterPro

REC only

Lokiarch_00080	KKK46580	Signal transduction response regulator...	CDD	UniProt	Pfam	InterPro
Lokiarch_00470	KKK46538	Signal transduction response regulator...	CDD	UniProt	Pfam	InterPro
Lokiarch_02270	KKK46321	Signal transduction response regulator...	CDD	UniProt	Pfam	InterPro
Lokiarch_04340	KKK46073	Signal transduction response regulator...	CDD	UniProt	Pfam	InterPro
Lokiarch_05420	KKK45955	Signal transduction response regulator...	CDD	UniProt	Pfam	InterPro
Lokiarch_08380	KKK45610	Signal transduction response regulator...	CDD	UniProt	Pfam	InterPro
Lokiarch_12580	KKK45110	Signal transduction response regulator...	CDD	UniProt	Pfam	InterPro
Lokiarch_15070	KKK44823	Signal transduction response regulator...	CDD	UniProt	Pfam	InterPro
Lokiarch_20950	KKK44134	Signal transduction response regulator...	CDD	UniProt	Pfam	InterPro
Lokiarch_22830	KKK43941	Signal transduction response regulator...	CDD	UniProt	Pfam	InterPro
Lokiarch_24680	KKK43712	Signal transduction response regulator...	CDD	UniProt	Pfam	InterPro
Lokiarch_32370	KKK42814	Chemotaxis protein CheY [Lokiarchaeum ...	CDD	UniProt	Pfam	InterPro
Lokiarch_32880	KKK42744	Signal transduction response regulator...	CDD	UniProt	Pfam	InterPro
Lokiarch_36940	KKK42174	Signal transduction response regulator...	CDD	UniProt	Pfam	InterPro
Lokiarch_37290	KKK42141	Signal transduction response regulator...	CDD	UniProt	Pfam	InterPro
Lokiarch_37560	KKK42103	Signal transduction response regulator...	CDD	UniProt	Pfam	InterPro
Lokiarch_39220	KKK41897	Signal transduction response regulator...	CDD	UniProt	Pfam	InterPro
Lokiarch_43970	KKK41338	Signal transduction response regulator...	CDD	UniProt	Pfam	InterPro
*Lokiarch_42390	KKK41515	Signal transduction response regulator...	CDD	UniProt	Pfam	InterPro

REC-HisK

Lokiarch_29550	KKK43125	Signal transduction histidine kinase [...	CDD	UniProt	Pfam	InterPro

REC-CheB

Lokiarch_16240	KKK44677	Signal transduction response regulator...	CDD	UniProt	Pfam	InterPro
Lokiarch_32440	KKK42821	Signal transduction response regulator...	CDD	UniProt	Pfam	InterPro

Other RRs

Lokiarch_05110 KKK46010 hypothetical protein Lokiarch_05110 [L... CDD UniProt Pfam InterPro (RibbonHH-HisKA-REC)

Lokiarch_06650 KKK45814 Signal transduction response regulator... CDD UniProt Pfam InterPro (???-HisKA-REC)

Lokiarch_07240 KKK45742 Signal transduction response regulator... CDD UniProt Pfam InterPro (RibbonHH-HisKA-REC)

Lokiarch_13560	KKK44996	Signal transduction response regulator...	CDD	UniProt	Pfam	InterPro	only in Loki, Heimdall
Lokiarch_20350	KKK44156	Signal transduction response regulator...	CDD	UniProt	Pfam	InterPro	(RibbonHH-HisKA-REC)
Lokiarch_20360	KKK44157	Signal transduction response regulator...	CDD	UniProt	Pfam	InterPro	(RibbonHH-HisKA-REC)
Lokiarch_49480	KKK40668	Signal transduction response regulator...	CDD	UniProt	Pfam	InterPro	unique

---

Candidatus Lokiarchaeota archaeon CR_4

HisK, no REC

RBG13Loki_0330	OLS16050	multi-sensor signal transduction histi...	CDD	UniProt	Pfam	InterPro	
RBG13Loki_0480	OLS15889	sensory box protein [Candidatus Lokiar...	CDD	UniProt	Pfam	InterPro	
RBG13Loki_0482	OLS15891	PAS domain S-box [Candidatus Lokiarcha...	CDD	UniProt	Pfam	InterPro	
RBG13Loki_0546	OLS15812	CheA signal transduction histidine kin...	CDD	UniProt	Pfam	InterPro	
RBG13Loki_0550	OLS15816	hypothetical protein RBG13Loki_0550 [C...	CDD	UniProt	Pfam	InterPro	
RBG13Loki_0800	OLS15572	hybrid sensory histidine kinase BarA [...	CDD	UniProt	Pfam	InterPro	
RBG13Loki_1041	OLS15336	integral membrane sensor signal transd...	CDD	UniProt	Pfam	InterPro	
RBG13Loki_1711	OLS14669	phosphate sensor histidine kinase, HAM...	CDD	UniProt	Pfam	InterPro	
RBG13Loki_1732	OLS14630	integral membrane sensor hybrid histid...	CDD	UniProt	Pfam	InterPro	
RBG13Loki_1752	OLS14650	PAS/PAC sensor signal transduction his...	CDD	UniProt	Pfam	InterPro	
RBG13Loki_1838	OLS14555	PAS domain S-box [Candidatus Lokiarcha...	CDD	UniProt	Pfam	InterPro	
RBG13Loki_2731	OLS13665	sensory transduction histidine kinase ...	CDD	UniProt	Pfam	InterPro	
RBG13Loki_2732	OLS13666	putative signal transduction histidine...	CDD	UniProt	Pfam	InterPro	
RBG13Loki_3134	OLS13260	integral membrane sensor signal transd...	CDD	UniProt	Pfam	InterPro	
RBG13Loki_3498	OLS12863	signal transduction histidine kinase [...	CDD	UniProt	Pfam	InterPro	
RBG13Loki_3963	OLS12427	sensory box protein [Candidatus Lokiar...	CDD	UniProt	Pfam	InterPro	
RBG13Loki_3964	OLS12428	PAS domain S-box [Candidatus Lokiarcha...	CDD	UniProt	Pfam	InterPro	
RBG13Loki_4103	OLS12280	sensory transduction histidine kinase ...	CDD	UniProt	Pfam	InterPro	
RBG13Loki_4106	OLS12283	multi-sensor signal transduction histi...	CDD	UniProt	Pfam	InterPro	
RBG13Loki_4112	OLS12289	PAS/PAC sensor signal transduction his...	CDD	UniProt	Pfam	InterPro	
RBG13Loki_4114	OLS12291	PAS/PAC sensor signal transduction his...	CDD	UniProt	Pfam	InterPro	
RBG13Loki_4117	OLS12294	two component system histidine kinase ...	CDD	UniProt	Pfam	InterPro	
RBG13Loki_4207	OLS12141	PAS/PAC sensor signal transduction his...	CDD	UniProt	Pfam	InterPro	
RBG13Loki_4368	OLS12024	PAS/PAC sensor signal transduction his...	CDD	UniProt	Pfam	InterPro	
RBG13Loki_4422	OLS11967	sensor histidine kinase protein [Candi...	CDD	UniProt	Pfam	InterPro	

Other HisK

RBG13Loki_2516	OLS13839	sensory transduction histidine kinase ...	CDD	UniProt	Pfam	InterPro	

REC only

RBG13Loki_1918	OLS14433	response regulator receiver protein [C...	CDD	UniProt	Pfam	InterPro	
RBG13Loki_4056	OLS12406	response regulator receiver protein [C...	CDD	UniProt	Pfam	InterPro	
RBG13Loki_1919	OLS14434	response regulator receiver protein [C...	CDD	UniProt	Pfam	InterPro	
RBG13Loki_2133	OLS14257	putative response regulator (CheY-like...	CDD	UniProt	Pfam	InterPro	
RBG13Loki_4057	OLS12407	response regulator receiver protein [C...	CDD	UniProt	Pfam	InterPro	
RBG13Loki_0147	OLS16236	two-component hybrid sensor and regula...	CDD	UniProt	Pfam	InterPro	
RBG13Loki_0548	OLS15814	response regulator receiver protein [C...	CDD	UniProt	Pfam	InterPro	
RBG13Loki_4290	OLS12092	component of chemotactic signal transd...	CDD	UniProt	Pfam	InterPro	
RBG13Loki_1689	OLS14688	response regulator receiver [Candidatu...	CDD	UniProt	Pfam	InterPro	

REC-HisK

RBG13Loki_1733	OLS14631	multisensor signal transduction histid...	CDD	UniProt	Pfam	InterPro	

REC-CheB

RBG13Loki_0547	OLS15813	response regulator receiver modulated ...	CDD	UniProt	Pfam	InterPro	

REC-PAS

RBG13Loki_4101	OLS12278	putative signaling protein [Candidatus...	CDD	UniProt	Pfam	InterPro	

Other RRs

RBG13Loki_1688	OLS14687	putative PAS/PAC sensor protein [Candi...	CDD	UniProt	Pfam	InterPro	(REC-PAS-wHTH)
RBG13Loki_1585	OLS14826	transcriptional regulatory protein [Ca...	CDD	UniProt	Pfam	InterPro	

Candidatus Heimdallarchaeota

Candidatus Heimdallarchaeota archaeon AB_125

HisK, no REC

HeimAB125_14200	OLS31916	Alkaline phosphatase synthesis sensor...	CDD	UniProt	Pfam	InterPro	
HeimAB125_16760	OLS31219	Sensor histidine kinase TodS [Candida...	CDD	UniProt	Pfam	InterPro	
HeimAB125_20900	OLS29990	Alginate biosynthesis sensor protein ...	CDD	UniProt	Pfam	InterPro	
HeimAB125_17880	OLS30870	hypothetical protein HeimAB125_17880,...	CDD	UniProt	Pfam	InterPro	
HeimAB125_11490	OLS32185	hypothetical protein HeimAB125_11490 ...	CDD	UniProt	Pfam	InterPro	
HeimAB125_20520	OLS30112	hypothetical protein HeimAB125_20520,...	CDD	UniProt	Pfam	InterPro	

Hybrid HisK

HeimAB125_12030	OLS32128	Blue-light-activated protein [Candida...	CDD	UniProt	Pfam	InterPro	

REC only

HeimAB125_14190	OLS31915	Chemotaxis protein CheY [Candidatus H...	CDD	UniProt	Pfam	InterPro	
HeimAB125_22250	OLS29525	Transcriptional regulatory protein Yy...	CDD	UniProt	Pfam	InterPro	

Other RRs

HeimAB125_05440	OLS32769	hypothetical protein HeimAB125_05440 ...	CDD	UniProt	Pfam	InterPro	(REC-PPDK)

Candidatus Heimdallarchaeota archaeon LC_2

HisK, no REC
HeimC2_15810 OLS26337 Nitrogen regulation protein NR(II), part... CDD UniProt Pfam InterPro
HeimC2_01450 OLS29297 Sensor protein ZraS [Candidatus Heimdall... CDD UniProt Pfam InterPro
HeimC2_34180 OLS21200 Sensor protein kinase WalK [Candidatus H... CDD UniProt Pfam InterPro
HeimC2_19620 OLS25286 hypothetical protein HeimC2_19620, parti... CDD UniProt Pfam InterPro
HeimC2_45130 OLS17060 Chemotaxis protein CheA [Candidatus Heim... CDD UniProt Pfam InterPro
HeimC2_12630 OLS27153 Chemotaxis protein CheA [Candidatus Heim... CDD UniProt Pfam InterPro
Hybrid HisK
HeimC2_01230 OLS29276 Blue-light-activated protein [Candidatus... CDD UniProt Pfam InterPro (PAS-PAS-HisK-REC)
HeimC2_08310 OLS28176 Sporulation kinase E [Candidatus Heimdal... CDD UniProt Pfam InterPro
HeimC2_23060 OLS24360 Blue-light-activated protein [Candidatus... CDD UniProt Pfam InterPro (PAS-PAS-HisK-REC)
HeimC2_36150 OLS20714 Blue-light-activated protein [Candidatus... CDD UniProt Pfam InterPro (CHASE-HisK-REC)
HeimC2_45040 OLS17051 Blue-light-activated protein [Candidatus... CDD UniProt Pfam InterPro (PAS-PAS-HisK-REC)
HeimC2_45180 OLS17065 Blue-light-activated protein [Candidatus... CDD UniProt Pfam InterPro (PAS-PAS-HisK-REC)
HeimC2_45200 OLS17067 Sensor protein FixL [Candidatus Heimdall... CDD UniProt Pfam InterPro
HeimC2_45220 OLS17069 Blue-light-activated protein [Candidatus... CDD UniProt Pfam InterPro
HeimC2_45230 OLS17070 Sensor protein ZraS [Candidatus Heimdall... CDD UniProt Pfam InterPro
REC only
HeimC2_27350 OLS23182 Chemotaxis protein CheY [Candidatus Heim... CDD UniProt Pfam InterPro
HeimC2_44360 OLS18434 Chemotaxis protein CheY [Candidatus Heim... CDD UniProt Pfam InterPro
HeimC2_32290 OLS21770 Chemotaxis protein CheY [Candidatus Heim... CDD UniProt Pfam InterPro
HeimC2_45120 OLS17059 Chemotaxis protein CheY [Candidatus Heim... CDD UniProt Pfam InterPro
HeimC2_44340 OLS18432 Chemotaxis protein CheY [Candidatus Heim... CDD UniProt Pfam InterPro
HeimC2_41320 OLS19452 hypothetical protein HeimC2_41320 [Candi... CDD UniProt Pfam InterPro
REC-CheB
HeimC2_12600 OLS27150 Chemotaxis response regulator protein-gl... CDD UniProt Pfam InterPro
HeimC2_45160 OLS17063 Chemotaxis response regulator protein-gl... CDD UniProt Pfam InterPro
Other RRs
HeimC2_01350 OLS29287 KDP operon transcriptional regulatory pr... CDD UniProt Pfam InterPro
HeimC2_04740 OLS28744 hypothetical protein HeimC2_04740 [Candi... CDD UniProt Pfam InterPro
HeimC2_10410 OLS27845 hypothetical protein HeimC2_10410 [Candi... CDD UniProt Pfam InterPro
HeimC2_10910 OLS27643 hypothetical protein HeimC2_10910 [Candi... CDD UniProt Pfam InterPro
HeimC2_11830 OLS27346 hypothetical protein HeimC2_11830 [Candi... CDD UniProt Pfam InterPro (REC-PPDK)
HeimC2_12440 OLS27252 Chemotaxis response regulator protein-gl... CDD UniProt Pfam InterPro
HeimC2_12450 OLS27253 Transcriptional regulatory protein SrrA ... CDD UniProt Pfam InterPro
HeimC2_15800 OLS26336 Thioredoxin reductase [Candidatus Heimda... CDD UniProt Pfam InterPro (REC-Thioredoxin reductase)
HeimC2_16010 OLS26189 hypothetical protein HeimC2_16010 [Candi... CDD UniProt Pfam InterPro
HeimC2_24580 OLS23943 Chemotaxis protein CheY [Candidatus Heim... CDD UniProt Pfam InterPro
HeimC2_25020 OLS23763 hypothetical protein HeimC2_25020 [Candi... CDD UniProt Pfam InterPro
HeimC2_26340 OLS23430 Chemotaxis protein CheY [Candidatus Heim... CDD UniProt Pfam InterPro
HeimC2_30070 OLS22308 hypothetical protein HeimC2_30070 [Candi... CDD UniProt Pfam InterPro
HeimC2_34170 OLS21199 hypothetical protein HeimC2_34170 [Candi... CDD UniProt Pfam InterPro
HeimC2_45210 OLS17068 Hydrogenase transcriptional regulatory p... CDD UniProt Pfam InterPro

---

Candidatus Heimdallarchaeota archaeon LC_3
HisK, no REC
HeimC3_02430 OLS27666 Adaptive-response sensory-kinase SasA [C... CDD UniProt Pfam InterPro
HeimC3_04220 OLS27423 putative sensor histidine kinase TcrY [C... CDD UniProt Pfam InterPro
HeimC3_05810 OLS27114 Phytochrome-like protein cph1 [Candidatu... CDD UniProt Pfam InterPro
HeimC3_06810 OLS26789 Osmolarity sensor protein EnvZ [Candidat... CDD UniProt Pfam InterPro
HeimC3_08490 OLS26530 putative sensor histidine kinase TcrY [C... CDD UniProt Pfam InterPro
HeimC3_08940 OLS26575 Sensor protein KdpD [Candidatus Heimdall... CDD UniProt Pfam InterPro
HeimC3_09130 OLS26363 Sensor histidine kinase TmoS [Candidatus... CDD UniProt Pfam InterPro
HeimC3_13240 OLS25745 putative sensor histidine kinase TcrY [C... CDD UniProt Pfam InterPro
HeimC3_13930 OLS25645 Sporulation kinase E [Candidatus Heimdal... CDD UniProt Pfam InterPro
HeimC3_14280 OLS25680 Adaptive-response sensory-kinase SasA [C... CDD UniProt Pfam InterPro
HeimC3_16290 OLS25185 Sensor protein KdpD [Candidatus Heimdall... CDD UniProt Pfam InterPro
HeimC3_16410 OLS25197 Alkaline phosphatase synthesis sensor pr... CDD UniProt Pfam InterPro
HeimC3_19410 OLS24511 Globin-coupled histidine kinase [Candida... CDD UniProt Pfam InterPro
HeimC3_19630 OLS24533 Sensor protein ZraS [Candidatus Heimdall... CDD UniProt Pfam InterPro
HeimC3_20510 OLS24333 Alkaline phosphatase synthesis sensor pr... CDD UniProt Pfam InterPro
HeimC3_22890 OLS23817 Sensor protein ZraS [Candidatus Heimdall... CDD UniProt Pfam InterPro
HeimC3_22910 OLS23819 Sensor protein ZraS [Candidatus Heimdall... CDD UniProt Pfam InterPro
HeimC3_24300 OLS23618 putative sensor histidine kinase TcrY [C... CDD UniProt Pfam InterPro
HeimC3_25250 OLS23361 Alkaline phosphatase synthesis sensor pr... CDD UniProt Pfam InterPro
HeimC3_34540 OLS21607 Alkaline phosphatase synthesis sensor pr... CDD UniProt Pfam InterPro
HeimC3_34830 OLS21574 Alkaline phosphatase synthesis sensor pr... CDD UniProt Pfam InterPro
HeimC3_37340 OLS21087 Alkaline phosphatase synthesis sensor pr... CDD UniProt Pfam InterPro
HeimC3_38260 OLS20915 Sensor protein ZraS [Candidatus Heimdall... CDD UniProt Pfam InterPro
HeimC3_39970 OLS20539 Sensor protein KdpD [Candidatus Heimdall... CDD UniProt Pfam InterPro
HeimC3_42490 OLS20038 Sensor protein ZraS [Candidatus Heimdall... CDD UniProt Pfam InterPro
HeimC3_44250 OLS19662 Nitrogen fixation regulatory protein [Ca... CDD UniProt Pfam InterPro
HeimC3_49540 OLS18429 hypothetical protein HeimC3_49540, parti... CDD UniProt Pfam InterPro

HeimC3_52020 OLS16692 Cell-division control histidine kinase P... CDD UniProt Pfam InterPro
HeimC3_55280 OLS16294 Chemotaxis protein CheA, partial [Candid... CDD UniProt Pfam InterPro
HeimC3_55320 OLS16298 Chemotaxis protein CheA [Candidatus Heim... CDD UniProt Pfam InterPro

Hybrid HisK

HeimC3_40090 OLS20551 Blue-light-activated protein [Candidatus... CDD UniProt Pfam InterPro

REC only

HeimC3_04930 OLS27026 hypothetical protein HeimC3_04930 [Candi... CDD UniProt Pfam InterPro
HeimC3_05030 OLS27036 Regulator of RpoS [Candidatus Heimdallar... CDD UniProt Pfam InterPro
HeimC3_07500 OLS26648 Transcriptional regulatory protein ZraR ... CDD UniProt Pfam InterPro
HeimC3_10590 OLS26086 Response regulator SaeR [Candidatus Heim... CDD UniProt Pfam InterPro
HeimC3_16650 OLS25035 Cyclic di-GMP phosphodiesterase response... CDD UniProt Pfam InterPro
HeimC3_22080 OLS23983 Regulator of RpoS [Candidatus Heimdallar... CDD UniProt Pfam InterPro
HeimC3_24850 OLS23410 Regulator of RpoS [Candidatus Heimdallar... CDD UniProt Pfam InterPro
HeimC3_25280 OLS23364 Signal transduction histidine-protein ki... CDD UniProt Pfam InterPro
HeimC3_34560 OLS21609 Chemotaxis protein CheY [Candidatus Heim... CDD UniProt Pfam InterPro
HeimC3_34820 OLS21573 Response regulator MprA, partial [Candid... CDD UniProt Pfam InterPro
HeimC3_37350 OLS21088 Response regulator MprA, partial [Candid... CDD UniProt Pfam InterPro
HeimC3_47210 OLS19145 hypothetical protein HeimC3_47210 [Candi... CDD UniProt Pfam InterPro
HeimC3_47220 OLS19146 Transcriptional regulatory protein ZraR ... CDD UniProt Pfam InterPro
HeimC3_47990 OLS18861 hypothetical protein HeimC3_47990 [Candi... CDD UniProt Pfam InterPro
HeimC3_55290 OLS16295 Chemotaxis protein CheY [Candidatus Heim... CDD UniProt Pfam InterPro
*HeimC3_10920 OLS26119 hypothetical protein HeimC3_10920 [Candi... CDD UniProt Pfam InterPro

REC-HisK

HeimC3_05100 OLS27043 Sporulation kinase A [Candidatus Heimdal... CDD UniProt Pfam InterPro
HeimC3_06660 OLS26909 Sensor protein FixL [Candidatus Heimdall... CDD UniProt Pfam InterPro
HeimC3_07560 OLS26654 Sporulation kinase A [Candidatus Heimdal... CDD UniProt Pfam InterPro
HeimC3_08590 OLS26540 Sporulation kinase E [Candidatus Heimdal... CDD UniProt Pfam InterPro
HeimC3_16370 OLS25193 Response regulator SaeR [Candidatus Heim... CDD UniProt Pfam InterPro
HeimC3_24200 OLS23608 Sporulation kinase E [Candidatus Heimdal... CDD UniProt Pfam InterPro
HeimC3_36920 OLS21133 Sensor protein ZraS [Candidatus Heimdall... CDD UniProt Pfam InterPro
HeimC3_38320 OLS20921 Response regulator ArlR [Candidatus Heim... CDD UniProt Pfam InterPro

REC-PAS

HeimC3_08160 OLS26714 Response regulator SaeR [Candidatus Heim... CDD UniProt Pfam InterPro
HeimC3_08350 OLS26733 Sensor histidine kinase TmoS [Candidatus... CDD UniProt Pfam InterPro
HeimC3_08410 OLS26739 Stage 0 sporulation protein A [Candidatu... CDD UniProt Pfam InterPro
HeimC3_16300 OLS25186 Cyclic di-GMP phosphodiesterase response... CDD UniProt Pfam InterPro
HeimC3_19170 OLS24487 Response regulator SaeR [Candidatus Heim... CDD UniProt Pfam InterPro
HeimC3_29710 OLS22697 Response regulator MprA [Candidatus Heim... CDD UniProt Pfam InterPro
HeimC3_35910 OLS21346 Transcriptional regulatory protein TcrA ... CDD UniProt Pfam InterPro
HeimC3_36900 OLS21131 Sensor histidine kinase TmoS [Candidatus... CDD UniProt Pfam InterPro
HeimC3_39980 OLS20540 Cyclic di-GMP phosphodiesterase response... CDD UniProt Pfam InterPro
HeimC3_42540 OLS20043 Stage 0 sporulation protein A [Candidatu... CDD UniProt Pfam InterPro
HeimC3_55370 OLS16303 Nitrogen fixation regulatory protein [Ca... CDD UniProt Pfam InterPro

Other RRs

HeimC3_34530 OLS21606 Alkaline phosphatase synthesis transcrip... CDD UniProt Pfam InterPro
HeimC3_20500 OLS24332 Alkaline phosphatase synthesis transcrip... CDD UniProt Pfam InterPro
HeimC3_16400 OLS25196 Alkaline phosphatase synthesis transcrip... CDD UniProt Pfam InterPro
HeimC3_25240 OLS23360 Alkaline phosphatase synthesis transcrip... CDD UniProt Pfam InterPro
HeimC3_46250 OLS19303 Response regulator SaeR [Candidatus Heim... CDD UniProt Pfam InterPro (wHTH-PAS-REC-PAS)
HeimC3_04650 OLS27294 Response regulator SaeR [Candidatus Heim... CDD UniProt Pfam InterPro
HeimC3_38270 OLS20916 Hydrogenase transcriptional regulatory p... CDD UniProt Pfam InterPro
HeimC3_22900 OLS23818 Hydrogenase transcriptional regulatory p... CDD UniProt Pfam InterPro
HeimC3_09140 OLS26364 Thioredoxin reductase [Candidatus Heimda... CDD UniProt Pfam InterPro (REC-Thioredoxin reductase)
HeimC3_22750 OLS23803 Transforming protein p29 precursor [Cand... CDD UniProt Pfam InterPro (REC-GTPase_XXX)
HeimC3_22760 OLS23804 DNA-binding response regulator MtrA [Can... CDD UniProt Pfam InterPro (REC-GTPase_XXX)
HeimC3_22790 OLS23807 hypothetical protein HeimC3_22790 [Candi... CDD UniProt Pfam InterPro (REC-PPDK)

Candidatus Thorarchaeota

Candidatus Thorarchaeota archaeon AB_25

HisK, no REC

ThorAB25_12070 OLS30105 hypothetical protein ThorAB25_12070, p... CDD UniProt Pfam InterPro
ThorAB25_13800 OLS29848 Sensor protein FixL [Candidatus Thorar... CDD UniProt Pfam InterPro
ThorAB25_14840 OLS29699 Histidine protein kinase SaeS [Candida... CDD UniProt Pfam InterPro
ThorAB25_18290 OLS28591 Sporulation kinase A [Candidatus Thora... CDD UniProt Pfam InterPro
ThorAB25_18300 OLS28592 Swarming motility regulation sensor pr... CDD UniProt Pfam InterPro
ThorAB25_24240 OLS23754 Sensor histidine kinase DpiB [Candidat... CDD UniProt Pfam InterPro
ThorAB25_25390 OLS22577 Autoinducer 2 sensor kinase/phosphatas... CDD UniProt Pfam InterPro
ThorAB25_26240 OLS21915 Sporulation kinase A [Candidatus Thora... CDD UniProt Pfam InterPro
ThorAB25_28470 OLS20297 hypothetical protein ThorAB25_28470, p... CDD UniProt Pfam InterPro
ThorAB25_28990 OLS19832 Signal-transduction histidine kinase s... CDD UniProt Pfam InterPro

REC only

ThorAB25_07510 OLS30773 Response regulator protein VraR [Candi... CDD UniProt Pfam InterPro
ThorAB25_11630 OLS30166 Chemotaxis protein CheY [Candidatus Th... CDD UniProt Pfam InterPro
ThorAB25_11640 OLS30167 putative transcriptional regulatory pr... CDD UniProt Pfam InterPro
ThorAB25_11660 OLS30169 Alkaline phosphatase synthesis transcr... CDD UniProt Pfam InterPro

REC-HisK
ThorAB25_17150 OLS29206 Sporulation kinase E [Candidatus Thora... CDD UniProt Pfam InterPro
REC-PAS\GAF
ThorAB25_18330 OLS28595 Stage 0 sporulation protein A [Candida... CDD UniProt Pfam InterPro

---

Candidatus Thorarchaeota
Candidatus Thorarchaeota archaeon SMTZ-45
HisK, no REC
AM326_00720 KXH72882 hypothetical protein AM326_00720 [Candida... CDD UniProt Pfam InterPro
AM326_06830 KXH76655 hypothetical protein AM326_06830 [Candida... CDD UniProt Pfam InterPro
AM326_07950 KXH76036 hypothetical protein AM326_07950 [Candida... CDD UniProt Pfam InterPro
AM326_01840 KXH77035 hypothetical protein AM326_01840 [Candida... CDD UniProt Pfam InterPro
REC only
AM326_03580 KXH72432 hypothetical protein AM326_03580 [Candida... CDD UniProt Pfam InterPro

---

Candidatus Thorarchaeota archaeon SMTZ1-45
HisK, no REC
AM325_12800 KXH74302 hypothetical protein AM325_12800 [Candida... CDD UniProt Pfam InterPro
AM325_13570 KXH73682 hypothetical protein AM325_13570 [Candida... CDD UniProt Pfam InterPro
AM325_13650 KXH73644 hypothetical protein AM325_13650 [Candida... CDD UniProt Pfam InterPro
AM325_13670 KXH73648 hypothetical protein AM325_13670 [Candida... CDD UniProt Pfam InterPro
AM325_13725 KXH73581 hypothetical protein AM325_13725 [Candida... CDD UniProt Pfam InterPro
AM325_13730 KXH73582 hypothetical protein AM325_13730 [Candida... CDD UniProt Pfam InterPro
AM325_14985 KXH71395 hypothetical protein AM325_14985 [Candida... CDD UniProt Pfam InterPro
AM325_16040 KXH75682 hypothetical protein AM325_16040 [Candida... CDD UniProt Pfam InterPro
REC only
AM325_08215 KXH73176 hypothetical protein AM325_08215 [Candida... CDD UniProt Pfam InterPro
AM325_08210 KXH73193 hypothetical protein AM325_08210 [Candida... CDD UniProt Pfam InterPro
AM325_06800 KXH73889 hypothetical protein AM325_06800 [Candida... CDD UniProt Pfam InterPro
*AM325_08225 KXH73178 hypothetical protein AM325_08225 [Candida... CDD UniProt Pfam InterPro
REC-HisK
AM325_03235 KXH70391 hypothetical protein AM325_03235 [Candida... CDD UniProt Pfam InterPro
REC-GAF-PAS
AM325_13850 KXH73528 hypothetical protein AM325_13850 [Candida... CDD UniProt Pfam InterPro
Other RRs
AM325_16070 KXH75686 hypothetical protein AM325_16070 [Candida... CDD UniProt Pfam InterPro REC-HisKA

Candidatus Thorarchaeota archaeon SMTZ1-83
HisK, no REC
AM324_00720 KXH72757 hypothetical protein AM324_00720 [Candida... CDD UniProt Pfam InterPro
AM324_01610 KXH70125 hypothetical protein AM324_01610 [Candida... CDD UniProt Pfam InterPro
AM324_01875 KXH69850 hypothetical protein AM324_01875 [Candida... CDD UniProt Pfam InterPro
AM324_02020 KXH69727 hypothetical protein AM324_02020 [Candida... CDD UniProt Pfam InterPro
AM324_03460 KXH77605 hypothetical protein AM324_03460, partial... CDD UniProt Pfam InterPro
AM324_07590 KXH72273 hypothetical protein AM324_07590 [Candida... CDD UniProt Pfam InterPro
AM324_10450 KXH69774 hypothetical protein AM324_10450 [Candida... CDD UniProt Pfam InterPro
AM324_10920 KXH78072 hypothetical protein AM324_10920 [Candida... CDD UniProt Pfam InterPro
AM324_10940 KXH78076 hypothetical protein AM324_10940 [Candida... CDD UniProt Pfam InterPro
AM324_10955 KXH78079 hypothetical protein AM324_10955 [Candida... CDD UniProt Pfam InterPro
AM324_10960 KXH78080 hypothetical protein AM324_10960 [Candida... CDD UniProt Pfam InterPro
AM324_14600 KXH76103 hypothetical protein AM324_14600 [Candida... CDD UniProt Pfam InterPro
AM324_14700 KXH76058 hypothetical protein AM324_14700 [Candida... CDD UniProt Pfam InterPro
AM324_15385 KXH75146 hypothetical protein AM324_15385 [Candida... CDD UniProt Pfam InterPro
REC only
AM324_05925 KXH74275 hypothetical protein AM324_05925 [Candida... CDD UniProt Pfam InterPro
AM324_05930 KXH74276 hypothetical protein AM324_05930 [Candida... CDD UniProt Pfam InterPro
AM324_10965 KXH78081 hypothetical protein AM324_10965 [Candida... CDD UniProt Pfam InterPro
AM324_10585 KXH78171 hypothetical protein AM324_10585 [Candida... CDD UniProt Pfam InterPro
NtrC family (REC-AAA-Fis)
AM324_01870 KXH69849 Fis family transcriptional regulator [Can... CDD UniProt Pfam InterPro

---

DPANN group
Candidatus Aenigmarchaeota
Candidatus Aenigmarchaeota archaeon CG1_02_38_14
HisK, no REC
AUJ50_03965 OIN86203 hypothetical protein AUJ50_03965 [Candida... CDD UniProt Pfam InterPro
REC only
AUJ50_00645 OIN88531 hypothetical protein AUJ50_00645 [Candida... CDD UniProt Pfam InterPro
AUJ50_03955 OIN86201 hypothetical protein AUJ50_03955 [Candida... CDD UniProt Pfam InterPro
AUJ50_03960 OIN86202 hypothetical protein AUJ50_03960 [Candida... CDD UniProt Pfam InterPro

---

Candidatus Micrarchaeota

Candidatus Micrarchaeota archaeon CG1_02_55_22

HisK, no REC

AUJ14_02250 OI026197 hypothetical protein AUJ14_02250 [Candida... CDD UniProt Pfam InterPro

Other RRs

AUJ14_05545 OI024744 hypothetical protein AUJ14_05545 [Candida... CDD UniProt Pfam InterPro

---

Candidatus Micrarchaeota archaeon RBG_16_49_10

REC only

A3K63_00125 OGI15767 hypothetical protein A3K63_00125 [Candida... CDD UniProt Pfam InterPro

---

Candidatus Micrarchaeum acidiphilum ARMAN-1

HisK, no REC

BK997_01990 OJI07769 hypothetical protein BK997_01990 [Candida... CDD UniProt Pfam InterPro

REC-GGDEF

BK997_02000 OJI07771 hypothetical protein BK997_02000 [Candida... CDD UniProt Pfam InterPro (REC-GGDEF)

Candidatus Micrarchaeum acidiphilum ARMAN-2

HisK, no REC

UNLARM2_0160 EET90488 Signal transduction histidine kinase reg... CDD UniProt Pfam InterPro
UNLARM2_0237 EET90383 PAS/PAC sensor signal transduction histi... CDD UniProt Pfam InterPro

REC-GGDEF

UNLARM2_0239 EET90385 response regulator receiver protein [Can... CDD UniProt Pfam InterPro (wHTH-REC-GGDEF)

Candidatus Micrarchaeum sp. AZ1

REC-GGDEF

JJ59_05330 OJT93977 hypothetical protein JJ59_05330 [Candidatu... CDD UniProt Pfam InterPro (REC-GGDEF)

Candidatus Nanohaloarchaeota

Candidatus Nanosalina sp. J07AB43

HisK, no REC

J07AB43_06780 EGQ42691 putative histidine kinase [Candidatus N... CDD UniProt Pfam InterPro

REC-HisK

J07AB43_10260 EGQ43037 PAS sensor histidine kinase [Candidatus... CDD UniProt Pfam InterPro

REC-PAS

J07AB43_06220 EGQ43744 response regulator [Candidatus Nanosali... CDD UniProt Pfam InterPro

wHTH-REC

J07AB43_10250 EGQ43036 putative transcriptional regulator [Can... CDD UniProt Pfam InterPro

---

Candidatus Nanosalinarum sp. J07AB56

REC only

J07AB56_11580 EGQ40429 hypothetical protein J07AB56_11580 [Ca... CDD UniProt Pfam InterPro

---

Candidatus Pacearchaeota

Candidatus Pacearchaeota archaeon RBG_13_36_9

HisK, no REC

A3K73_04080 OGJ15728 hypothetical protein A3K73_04080, partial... CDD UniProt Pfam InterPro
A3K73_05640 OGJ21307 hypothetical protein A3K73_05640 [Candida... CDD UniProt Pfam InterPro
A3K73_05595 OGJ21298 hypothetical protein A3K73_05595 [Candida... CDD UniProt Pfam InterPro

REC only

A3K73_05570 OGJ21293 hypothetical protein A3K73_05570 [Candida... CDD UniProt Pfam InterPro
A3K73_05590 OGJ21297 hypothetical protein A3K73_05590 [Candida... CDD UniProt Pfam InterPro
A3K73_05635 OGJ21306 hypothetical protein A3K73_05635 [Candida... CDD UniProt Pfam InterPro
A3K73_05620 OGJ21303 hypothetical protein A3K73_05620 [Candida... CDD UniProt Pfam InterPro

Other RRs

A3K73_00520 OGJ16906 hypothetical protein A3K73_00520 [Candida... CDD UniProt Pfam InterPro
A3K73_04085 OGJ15729 hypothetical protein A3K73_04085 [Candida... CDD UniProt Pfam InterPro

---

Candidatus Pacearchaeota archaeon RBG_16_35_8

REC only

A3K62_00590 OGJ12735 hypothetical protein A3K62_00590 [Candida... CDD UniProt Pfam InterPro

---

Candidatus Woesearchaeota

Candidatus Woesearchaeota archaeon CG1_02_57_44

HisK, no REC

AUJ68_00545 OI066483 hypothetical protein AUJ68_00545 [Candida... CDD UniProt Pfam InterPro
AUJ68_00560 OI066486 hypothetical protein AUJ68_00560 [Candida... CDD UniProt Pfam InterPro

AUJ68_04715 OIO64828 hypothetical protein AUJ68_04715 [Candida... CDD UniProt Pfam InterPro
REC only
AUJ68_00555 OIO66485 hypothetical protein AUJ68_00555 [Candida... CDD UniProt Pfam InterPro

---

TACK group
Candidatus Bathyarchaeota
Candidatus Bathyarchaeota archaeon B26-1
REC only
AYL31_009600 KYH39473 response regulator receiver domain-conta... CDD UniProt Pfam InterPro

---

Candidatus Bathyarchaeota archaeon B26-2
REC only
AYL32_010350 KYH40679 response regulator receiver domain-conta... CDD UniProt Pfam InterPro
AYL32_011520 KYH40474 response regulator receiver domain-conta... CDD UniProt Pfam InterPro

---

Candidatus Bathyarchaeota archaeon B63
REC only
AYL33_000980 KYH42750 response regulator receiver domain-conta... CDD UniProt Pfam InterPro

---

Candidatus Bathyarchaeota archaeon RBG_13_38_9
HisK, no REC
A3K80_03685 OGD53009 hypothetical protein A3K80_03685 [Candida... CDD UniProt Pfam InterPro
REC only
A3K80_03790 OGD53025 response regulator [Candidatus Bathyarcha... CDD UniProt Pfam InterPro
A3K80_03830 OGD53028 hypothetical protein A3K80_03830 [Candida... CDD UniProt Pfam InterPro
REC-HisK
A3K80_03775 OGD53022 hypothetical protein A3K80_03775 [Candida... CDD UniProt Pfam InterPro
Other RRs
A3K80_03680 OGD53008 hypothetical protein A3K80_03680 [Candida... CDD UniProt Pfam InterPro
A3K80_03780 OGD53023 hypothetical protein A3K80_03780 [Candida... CDD UniProt Pfam InterPro
A3K80_03765 OGD53020 hypothetical protein A3K80_03765 [Candida... CDD UniProt Pfam InterPro
A3K80_03770 OGD53021 hypothetical protein A3K80_03770 [Candida... CDD UniProt Pfam InterPro

---

Candidatus Bathyarchaeota archaeon RBG_13_46_16b
HisK, no REC
A3K79_04075 OGD45073 hypothetical protein A3K79_04075 [Candida... CDD UniProt Pfam InterPro
A3K79_05095 OGD47194 hypothetical protein A3K79_05095, partial... CDD UniProt Pfam InterPro
A3K79_06350 OGD47042 hypothetical protein A3K79_06350 [Candida... CDD UniProt Pfam InterPro
REC only
A3K79_00270 OGD47957 hypothetical protein A3K79_00270 [Candida... CDD UniProt Pfam InterPro
A3K79_01200 OGD45118 hypothetical protein A3K79_01200 [Candida... CDD UniProt Pfam InterPro
A3K79_02080 OGD47072 hypothetical protein A3K79_02080 [Candida... CDD UniProt Pfam InterPro
REC-HisK
A3K79_00265 OGD47956 hypothetical protein A3K79_00265 [Candida... CDD UniProt Pfam InterPro
REC-PAS
A3K79_07615 OGD44168 hypothetical protein A3K79_07615, partial... CDD UniProt Pfam InterPro

---

Candidatus Bathyarchaeota archaeon RBG_13_52_12
HisK, no REC
A3K78_02615 OGD55262 hypothetical protein A3K78_02615 [Candida... CDD UniProt Pfam InterPro
A3K78_06220 OGD56148 hypothetical protein A3K78_06220, partial... CDD UniProt Pfam InterPro
A3K78_09135 OGD60431 hypothetical protein A3K78_09135 [Candida... CDD UniProt Pfam InterPro
A3K78_10795 OGD58987 hypothetical protein A3K78_10795 [Candida... CDD UniProt Pfam InterPro
REC only
A3K78_02610 OGD55264 hypothetical protein A3K78_02610 [Candida... CDD UniProt Pfam InterPro
A3K78_05125 OGD56561 two-component system response regulator [... CDD UniProt Pfam InterPro
A3K78_06255 OGD56153 hypothetical protein A3K78_06255 [Candida... CDD UniProt Pfam InterPro
A3K78_06265 OGD56155 hypothetical protein A3K78_06265 [Candida... CDD UniProt Pfam InterPro
A3K78_10790 OGD58986 hypothetical protein A3K78_10790 [Candida... CDD UniProt Pfam InterPro
Other RRs
A3K78_02605 OGD55261 hypothetical protein A3K78_02605 [Candida... CDD UniProt Pfam InterPro
A3K78_05120 OGD56550 hypothetical protein A3K78_05120 [Candida... CDD UniProt Pfam InterPro
A3K78_09140 OGD60432 hypothetical protein A3K78_09140 [Candida... CDD UniProt Pfam InterPro

---

Candidatus Bathyarchaeota archaeon RBG_13_60_20
HisK, no REC
A3K81_01540 OGD55865 hypothetical protein A3K81_01540 [Candida... CDD UniProt Pfam InterPro
REC only
A3K81_04645 OGD53355 hypothetical protein A3K81_04645 [Candida... CDD UniProt Pfam InterPro
REC-HisK
A3K81_06585 OGD54085 hypothetical protein A3K81_06585 [Candida... CDD UniProt Pfam InterPro

A3K81_00905 OGD54583 hypothetical protein A3K81_00905 [Candida... CDD UniProt Pfam InterPro
A3K81_02575 OGD53329 hypothetical protein A3K81_02575 [Candida... CDD UniProt Pfam InterPro
Other RRs
A3K81_01690 OGD55697 hypothetical protein A3K81_01690 [Candida... CDD UniProt Pfam InterPro
A3K81_03960 OGD52326 hypothetical protein A3K81_03960 [Candida... CDD UniProt Pfam InterPro

---

Candidatus Bathyarchaeota archaeon RBG_16_48_13
HisK, no REC
A3K70_04505 OGD45808 hypothetical protein A3K70_04505 [Candida... CDD UniProt Pfam InterPro
REC only
A3K70_00955 OGD45656 hypothetical protein A3K70_00955 [Candida... CDD UniProt Pfam InterPro
REC-HisK
A3K70_00960 OGD45657 hypothetical protein A3K70_00960 [Candida... CDD UniProt Pfam InterPro

---

Candidatus Bathyarchaeota archaeon RBG_16_57_9
HisK, no REC
A3K69_03215 OGD44555 hypothetical protein A3K69_03215 [Candida... CDD UniProt Pfam InterPro
A3K69_03770 OGD45604 hypothetical protein A3K69_03770 [Candida... CDD UniProt Pfam InterPro
REC only
A3K69_02765 OGD45447 hypothetical protein A3K69_02765 [Candida... CDD UniProt Pfam InterPro
A3K69_00135 OGD44459 hypothetical protein A3K69_00135 [Candida... CDD UniProt Pfam InterPro
REC-HisK
A3K69_01050 OGD46118 hypothetical protein A3K69_01050 [Candida... CDD UniProt Pfam InterPro
Other RRs
A3K69_06655 OGD44705 hypothetical protein A3K69_06655 [Candida... CDD UniProt Pfam InterPro
A3K69_03970 OGD48311 hypothetical protein A3K69_03970 [Candida... CDD UniProt Pfam InterPro
A3K69_05730 OGD48668 hypothetical protein A3K69_05730 [Candida... CDD UniProt Pfam InterPro

---

---

unclassified Crenarchaeota
Crenarchaeota archaeon 13_1_20CM_2_51_8
REC only
AUF79_04595 OLE91433 hypothetical protein AUF79_04595 [Crenarc... CDD UniProt Pfam InterPro
AUF79_17120 OLE84948 hypothetical protein AUF79_17120 [Crenarc... CDD UniProt Pfam InterPro
AUF79_01930 OLE91858 hypothetical protein AUF79_01930 [Crenarc... CDD UniProt Pfam InterPro
REC-wHTH
AUF79_12530 OLE89065 hypothetical protein AUF79_12530 [Crenarc... CDD UniProt Pfam InterPro (REC-LuxR)
AUF79_11280 OLE89891 DNA-binding response regulator [Crenarcha... CDD UniProt Pfam InterPro (REC-LuxR)
AUF79_11870 OLE89516 hypothetical protein AUF79_11870 [Crenarc... CDD UniProt Pfam InterPro (REC-LuxR)

Crenarchaeota archaeon 13_1_20CM_2_53_14
HisK, no REC
AUG17_00415 OLE59984 hypothetical protein AUG17_00415 [Crenarc... CDD UniProt Pfam InterPro
REC only
AUG17_00410 OLE59983 hypothetical protein AUG17_00410 [Crenarc... CDD UniProt Pfam InterPro

---

miscellaneous Crenarchaeota group archaeon SMTZ-80
HisK, no REC
AC481_01535 KON28410 hypothetical protein AC481_01535, partial... CDD UniProt Pfam InterPro
AC481_01710 KON28442 hypothetical protein AC481_01710 [miscell... CDD UniProt Pfam InterPro
AC481_05500 KON27185 hypothetical protein AC481_05500 [miscell... CDD UniProt Pfam InterPro
AC481_05740 KON27109 hypothetical protein AC481_05740 [miscell... CDD UniProt Pfam InterPro
AC481_07300 KON26384 histidine kinase, partial [miscellaneous ... CDD UniProt Pfam InterPro
REC only
AC481_04280 KON27604 hypothetical protein AC481_04280 [miscell... CDD UniProt Pfam InterPro
NtrC family (REC-AAA-Fis)
AC481_01540 KON28411 hypothetical protein AC481_01540 [miscell... CDD UniProt Pfam InterPro (REC-AAA-Fis)
AC481_01705 KON28441 hypothetical protein AC481_01705 [miscell... CDD UniProt Pfam InterPro (REC-AAA-Fis)
Other RRs
AC481_01605 KON28423 response regulator [miscellaneous Crenarc... CDD UniProt Pfam InterPro (REC-PglZ)

---

miscellaneous Crenarchaeota group archaeon SMTZ1-55
REC only
*AC480_06220 KON26173 hypothetical protein AC480_06220, partial... CDD UniProt Pfam InterPro
Frz family (REC-REC)
AC480_06215 KON26172 hypothetical protein AC480_06215 [miscell... CDD UniProt Pfam InterPro (REC-REC)
NtrC family (REC-AAA-Fis)
AC480_00075 KON30915 hypothetical protein AC480_00075 [miscell... CDD UniProt Pfam InterPro (REC-AAA-Fis)

---

miscellaneous Crenarchaeota group-6 archaeon AD8-1
HisK, no REC
AC479_03670 KON33644 hypothetical protein AC479_03670 [miscell... CDD UniProt Pfam InterPro

REC-HisK

AC479_01620 KON34176 hypothetical protein AC479_01620 [miscell... CDD UniProt Pfam InterPro

---

Please send corrections, questions and comments to Michael Galperin
